# Protecting group free radical C–H trifluoromethylation of peptides†
†Electronic supplementary information (ESI) available. See DOI: 10.1039/c8sc00368h


**DOI:** 10.1039/c8sc00368h

**Published:** 2018-04-10

**Authors:** Naoko Ichiishi, John P. Caldwell, Melissa Lin, Wendy Zhong, Xiaohong Zhu, Eric Streckfuss, Hai-Young Kim, Craig A. Parish, Shane W. Krska

**Affiliations:** a Chemistry Capabilities and Screening , Merck Sharp & Dohme Corp. , Kenilworth , New Jersey 07033 , USA . Email: shane_krska@merck.com; b Kenilworth Discovery Chemistry , Merck Sharp & Dohme Corp. , Kenilworth , New Jersey 07033 , USA; c Analytical Research & Development , Merck Sharp & Dohme Corp. , Rahway , NJ , USA; d West Point Discovery Chemistry , Merck Sharp & Dohme Corp. , West Point , Pennsylvania 19486 , USA; e Discovery Chemistry Modalities , Merck Sharp & Dohme Corp. , Kenilworth , New Jersey 07033 , USA . Email: craig_parish@merck.com

## Abstract

Two radical-based approaches have been developed to effect the trifluoromethylation of aryl C–H bonds in native peptides either using stoichiometric oxidant or visible light photoredox catalysis. The reported methods are able to derivatize tyrosine and tryptophan sidechains under biocompatible conditions, and a number of examples are reported involving fully unprotected peptides with up to 51 amino acids. The development of this chemistry adds to the growing array of chemical methods for selectively modifying amino acid residues in the context of complex peptides. The direct incorporation of trifluoromethyl groups into biopolymers enables the study of a range of biological and biochemical systems, and preliminary results indicate this method can be extended to the incorporation of other fluoroalkyl groups for bioconjugation applications.

## Abstract 2

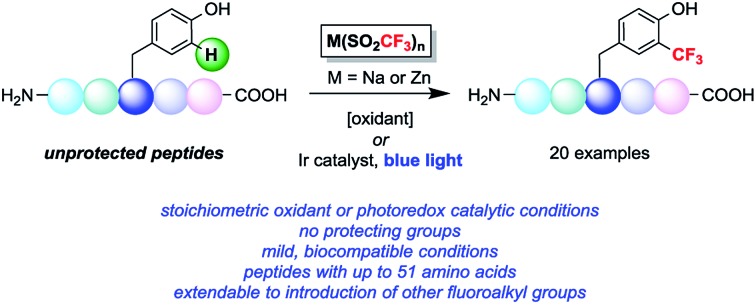
Two radical-based approaches enable the efficient trifluoromethylation of aromatic sidechains in fully unprotected peptides under mild, biocompatible conditions.

## Introduction

The landscape of molecular modalities employed in drug discovery has shifted dramatically in the last decade as peptides and proteins have taken on a more prominent role as therapeutics due to their exquisite target potency and selectivity.^1^ This growing importance of biomolecules has spurred the chemistry community to develop new biocompatible reactions for bioconjugation and/or post-translational modification.^2^ Peptide-based chemical probes have proven particularly useful in elucidating pharmacological mechanism of action, facilitating drug design and answering fundamental questions in biology. Among these, fluorinated peptides and proteins have enabled ^19^F-observed bio-NMR spectroscopy, which is a superb technique for studying protein structure, dynamics and protein–ligand interactions. This is due to the sensitivity and highly responsive nature of the ^19^F chemical shift to local environment.^3^,^4^ While fluorine-containing biomolecules are rarely found in nature, the *de novo* expression of proteins in the presence of ^19^F-labeled amino acids can be used to generate these tools. Alternatively, the direct modification of nucleophilic sidechains, such as cysteine thiols or lysine amines, with ^19^F-bearing electrophiles can be employed; however, such techniques have the potential to alter the inherent structure or function of a native biomolecule through changing the local polarity or disrupting key hydrogen-bonding interactions.^5^ Hence, a chemical method that directly introduces small, minimally perturbing fluorine-bearing substituents onto a native peptide or protein of interest without affecting polar sidechains would be a valuable tool for generating ^19^F-labeled biomolecules without compromising their overall structure and function and avoiding the need for *de novo* biosynthesis.

Late-stage C–H bond functionalization has recently gained prominence as a valuable approach to expand synthetic access to drug-like molecules and address many common problems encountered in drug discovery.^6^ Despite the many advances achieved in the utilization of C–H functionalization with small molecules, examples of applying such chemistries to peptides and proteins are relatively rare and often require protecting groups and/or harsh reaction conditions that limit the scope of peptide substrates.^7^

**Fig. 1:**
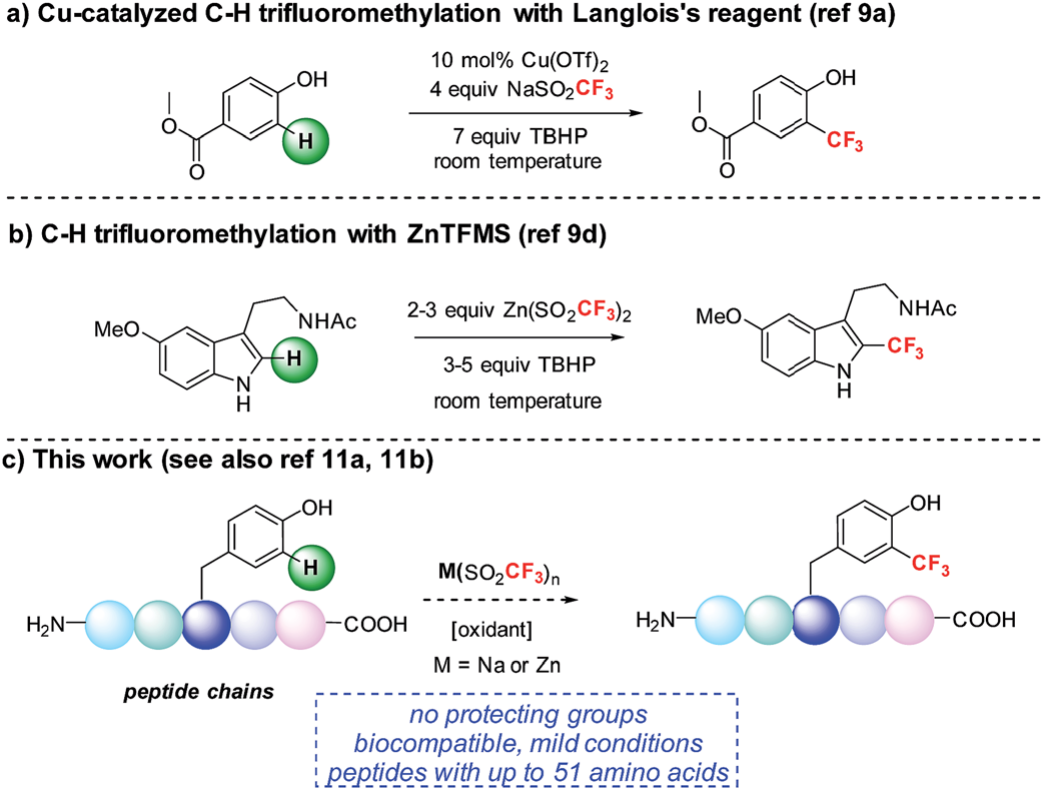
Radical trifluoromethylation of aromatic residues relevant to peptide functionalization.

We became interested in developing methods for the introduction of perfluoroalkyl groups into unprotected peptides and proteins *via* C–H functionalization under mild, biocompatible conditions to enable the generation of probes for ^19^F-bio-NMR spectroscopy and other applications.^8^ The addition of trifluoromethyl radicals to electron-rich aromatic and heterocyclic compounds has proven to be a successful approach for C–H trifluoromethylation (Fig. 1).^9^ In a seminal report, Langlois demonstrated the radical C–H trifluoromethylation of phenol using NaSO_2_CF_3_ and Cu(OTf)_2_ (Fig. 1a).9a,b Thereafter, Baran and coworkers reported the use of Zn(SO_2_CF_3_)_2_ (ZnTFMS) as a trifluoromethyl radical source, successfully trifluoromethylating electron-rich heterocycles found in complex drug molecules and natural products (Fig. 1b).9*e* Despite the utility of these methods, to date they have not been applied extensively to late-stage trifluoromethylation of peptide substrates.^10^,^11^ Herein, we demonstrate the radical-based trifluoromethylation of aromatic amino acid residues in peptides using trifluoromethyl sulfinate salts (Fig. 1c). The mild, biocompatible conditions identified in this study enable efficient trifluoromethyl incorporation into complex peptides without the need for protecting groups.

## Results and discussion

Among the four naturally occurring aromatic amino acids (Trp, Phe, Tyr and His), we reasoned that tyrosine functionalization would have the broadest applicability for peptide derivatization because of its high natural abundance and prevalence on the surfaces of proteins.^12^ Accordingly, we began our studies by examining the radical trifluoromethylation of a derivatized tyrosine analog, Ac-Tyr-NHMe (**1**), which we considered a reasonable model for tyrosine present in polypeptide chains (Table 1). Using Baran's ZnTFMS reagent and *t*-BuOOH (TBHP) as oxidant^13^ in a 2.5 : 1 DMSO/H_2_O mixture, the reaction afforded a mixture of mono-CF_3_ tyrosine **2** and bis-CF_3_ tyrosine **3** in 23% overall yield (entry 1). Further optimization of the reaction conditions led to the observation that addition of acid to the reaction mixture better solubilized all reagents at room temperature, thus enhancing the conversion to products (entry 2). Importantly, complete removal of DMSO from the reaction mixture was found to give enhanced product yields (entry 3). Further exploration of the effect of pH (entries 4–8) showed that a number of commonly utilized buffer systems could be employed, but pH 2.2 gave the optimal conditions for overall yield and mass balance.^14^,^15^

**Table 1:** Optimization of trifluoromethylation of Ac-Tyr-NHMe with stoichiometric oxidant

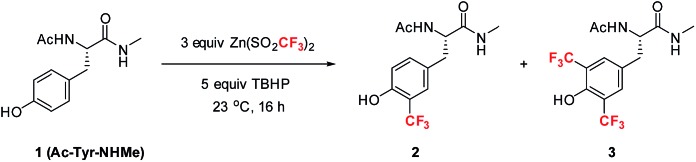			
Entry	Solvent	pH	Yield (**2** : **3**)^*a*^
1	DMSO : H_2_O (2.5 : 1)	8.9	23 (22 : 1)
2	DMSO : 10% AcOH (aq) (2.5 : 1)	4.4	40 (31 : 9)
3	10% AcOH (aq)	2.2	63 (41 : 22)
4	20 mM Na citrate (aq)	4.0	66 (56 : 10)
5	160 mM NaOAc	5.5	57 (55 : 2)
6	MES buffer	6.0	8 (8 : <1)
7	Tris buffer	7.5	32 (32 : <1)
8	30 mM NaOH/borax	10	53 (48 : 5)

^*a*^Yield was determined by ^19^F-NMR spectroscopy using α,α,α-trifluorotoluene as an internal standard. Each entry is an average of at least two data points. MES = 2-(*N*-morpholino)ethanesulfonic acid.

**Fig. 2:**
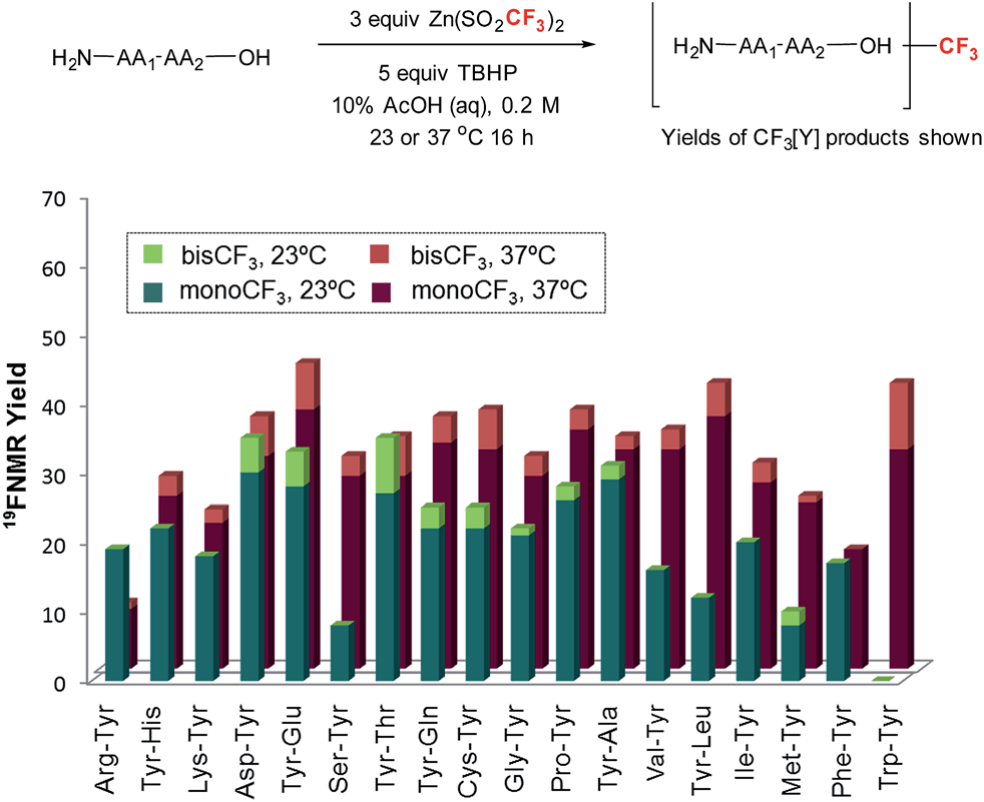
Amino acid compatibility of trifluoromethylation of tyrosine-containing dipeptides^a,b,c,d,e^ [^a^conditions: substrate (0.03 mmol), Zn(SO_2_CF_3_)_2_ (3 equiv.), TBHP (5 equiv.) in 10% AcOH (aq) buffer (0.2 M) for 16 h at 23 or 37 °C. Yield was determined by ^19^F-NMR spectroscopy using α,α,α-trifluorotoluene as an internal standard. Each entry is an average of at least two data points. ^b^Trifluoromethylation occurred at the 2-position of Trp indole in Trp–Tyr. ^c^Tyr–Thr–NH_2_ was used. ^d^Trifluoromethylated Cys–Tyr was obtained as the corresponding disulfide dimer. ^e^The thioether sidechain in trifluoromethylated Met–Tyr was oxidized].

With these reaction conditions in hand, we were interested in probing the selectivity and functional group compatibility of the trifluoromethylation reaction on unprotected peptide substrates. Thus, we applied the TBHP/ZnTFMS system to a series of tyrosine-containing dipeptides at two different temperatures (23 and 37 °C; Fig. 2). At room temperature, a number of the dipeptides, especially those with hydrophobic sidechains, exhibited poor solubility in the aqueous reaction medium, and hence gave poor yields. Overall, functional group compatibility was good and yields up to 35% could be obtained in some cases.^16^ Heating the reaction to physiological temperature (37 °C) resulted in increased reactivity for the majority of the dipeptides examined, most likely due to increased solubility, providing overall higher product yields (35–48%).

Although the TFMS/TBHP conditions were competent for the trifluoromethylation of tyrosine in unprotected dipeptides, further attempts to optimize the system were unsuccessful. Detailed GC-MS and NMR analyses identified side products such as tyrosine dimers and fluoroform that limited mass balance.^17^,^18^ These observations led us to evaluate other approaches that could better control the rate of formation of trifluoromethyl radicals. Baran, Blackmond and coworkers observed that running radical alkylation reactions without stirring gave improved yields,9*e* as did the use of electrochemistry for metering in oxidizing equivalents.^19^

**Fig. 3:**
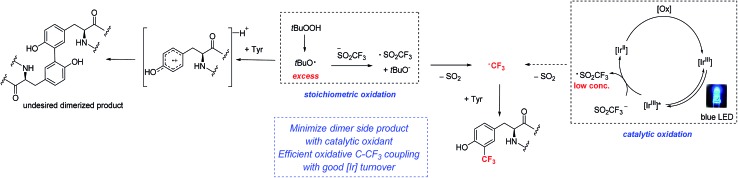
Comparison of stoichiometric and photoredox catalytic approaches to radical trifluoromethylation of peptides.

Along these lines, we hypothesized that if photoredox catalysis could be used to control the generation of oxidizing equivalents, we might be able to limit the formation of tyrosine radical that undergoes dimerization, and simultaneously reinforce the formation of the desired C–CF_3_ bond (Fig. 3).9e,^20^ Trifluoromethane sulfinate salts are known to possess readily accessible oxidation potentials (*E*_ox_ = 1.05 V *vs.* SCE, CF_3_SO_2_K).^21^ Thus, by choosing the appropriate photocatalyst, a system more compatible with sensitive peptide functionality might be accessible.

The investigation of a new photoredox protocol for this reaction began by evaluating 22 photoredox catalysts with **1** as substrate and 8 equivalents of either sodium or zinc trifluoromethane sulfinate salts.^18^ The use of Zn(SO_2_CF_3_)_2_ led to <10% combined yield of **2** and **3** in the presence of all tested photocatalysts. In contrast, NaSO_2_CF_3_ in combination with Ir[dF(CF_3_)ppy]_2_(dtbbpy)(PF_6_) (**4**) as photocatalyst gave 51% yield of product **2** with very little formation of the bis-trifluoromethylated product **3** (Table 2, entry 1).^22^ Other photoredox catalysts with similarly strong oxidizing potentials showed lower activity (entries 2, 3). Additional optimization with **4** showed that a number of solvents and solvent mixtures were compatible with this reaction (entries 4–7). For applications to polypeptide and protein substrates, the tolerance of the catalytic conditions to mixed aqueous systems is essential. Notably, conducting the photoredox trifluoromethylation of **2** in a 1 : 1 mixture of CH_3_CN : 10% AcOH (aq) gave 78% yield of the mono-CF_3_ product **2** (entry 5). Further optimization of pH showed that pH 2.2 gave the best yield, similar to the results obtained previously with stoichiometric oxidant.^18^ Other organic co-solvents such as ethyl acetate and acetone gave similarly high levels of productive reaction (entries 6, 7). Since the Ir catalyst was not water soluble, it was not possible to conduct the photoredox trifluoromethylation under fully aqueous conditions. Overall, the higher yields seen with the photoredox catalytic conditions supported our original hypothesis that slowly introducing oxidizing equivalents would improve the mass balance of this reaction.

**Table 2:** Development of photoredox catalytic trifluoromethylation conditions^*a*^

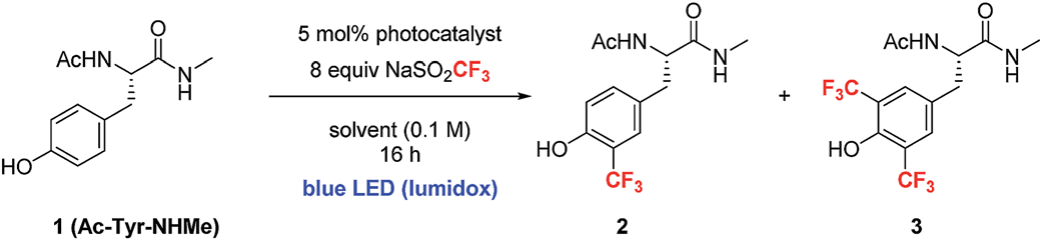			
Entry	Photocatalyst^*b*^	Solvent	Yield (**2** : **3**)^*c*^
1	Ir[dFCF_3_ppy] (**4**)	DMSO	51 (51 : <1)
2	*t*Bu-acr-*N*-Ph	DMSO	8 (8 : <1)
3	Ir[ppy]	DMSO	7 (7 : <1)
4	Ir[dFCF_3_ppy] (**4**)	CH_3_CN	84 (63 : 21)
5		CH_3_CN : 10% AcOH (aq)^*d*^	78 (78 : <1)
6		EtOAc : 10% AcOH (aq)^*d*^	78 (43 : 35)
7		Acetone : 10% AcOH (aq)^*d*^	76 (62 : 14)

^*a*^Conditions: **1** (0.01 mmol), photocatalyst (0.5 μmol, 5 mol%), NaSO_2_CF_3_ (0.08 mmol, 8 equiv.) in indicated solvent (0.1 M) for 16 hours at ambient temperature (27–32 °C due to the heat generation from blue LED) using 96-well plate as a reaction apparatus.

^*b*^Photocatalysts: Ir[dFCF_3_ppy] = Ir[{dFCF_3_ppy}_2_(dtbpy)](PF_6_); *t*Bu-acr-*N*-Ph = (*t*Bu-acridinium-*N*-Ph)(ClO_4_); Ir[ppy] = Ir[dtbpy(ppy)_2_](PF_6_).

^*c*^Yield was determined by ^19^F-NMR spectroscopy using α,α,α-trifluorotoluene as an internal standard. Each entry is an average of at least two data points.

^*d*^1 : 1 (v/v) solvent ratio.

In order to extend the photoredox catalytic conditions to more complex polypeptides, we next examined the trifluoromethylation of an unprotected dipeptide of aspartic acid and tyrosine (Asp–Tyr, **5**). In this series of experiments, we systematically varied the amount of photocatalyst and sulfinate salt and determined the effect on the combined yield of mono- and bis-trifluoromethylated Asp–Tyr products **5A** and **5B**, respectively (Table 3). The use of 15 mol% Ir catalyst and 20 equivalents of sulfinate salt gave the highest overall conversion (90%). Attempts to further boost yield using exogenous terminal oxidants were not successful, resulting in complex product mixtures.^18^

**Table 3:** Optimization studies of photoredox catalyzed trifluoromethylation of dipeptide^*a*^

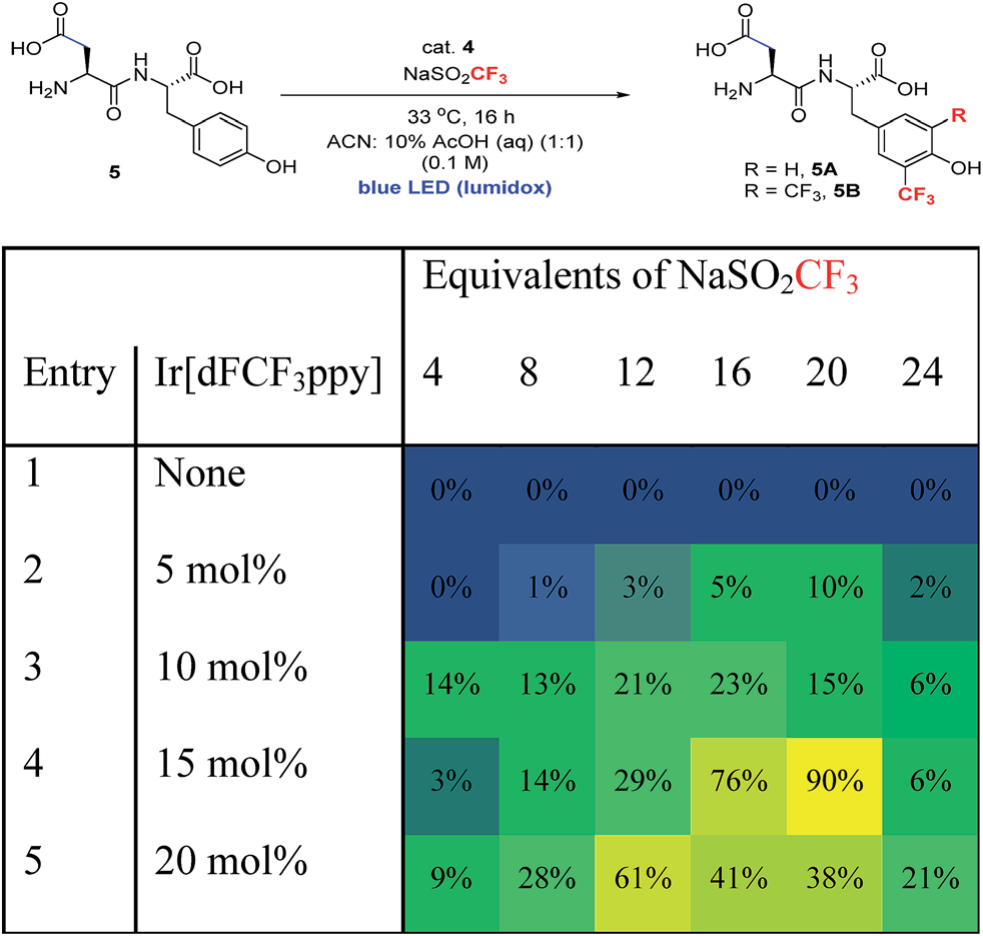

^*a*^Product % is the total yield of two products **5A** and **5B** (the average of two reactions), using biphenyl as an internal standard. Condition: **5** (0.01 mmol), cat. **4** (0–20 mol%), NaSO_2_CF_3_ (4–24 equivalents) in CH_3_CN : 10% AcOH (aq) mixture (0.1 M).

During the course of our study, it became apparent that the choice of the correct light source was critical for the optimal outcome of these reactions. When scaling up the photocatalytic trifluoromethylation of **5**, we first utilized a standard Kessil lamp (40W, blue LED) as a light source, giving a combined 39% yield of products **5A** and **5B**. Attempting to address the discrepancy between this result and that obtained in the smaller scale optimization screen that utilized the Lumidox 24-LED array as a light source, we repeated the reaction using the MSD photoreactor that has recently been reported to give enhanced rates of reaction for a number of photoredox catalytic processes.^23^ Using the MSD photoreactor, we were able to achieve a 61% combined yield of trifluoromethylated dipeptides (**5A** : **5B** = 46% : 15%), nearly double that seen with the less intense light source.

Having now developed two distinct sets of trifluoromethylation conditions, one employing stoichiometric TBHP oxidant in purely aqueous solvent and the other utilizing photoredox catalysis in mixed organic/aqueous solvent, we sought to understand the reaction scope with more complex peptides (Table 4). Thus, a series of polypeptides were subjected to both ZnTFMS/TBHP (condition X) and photoredox catalytic (condition Y) procedures. It is important to note that all peptides were examined in their native state without protecting groups. All trifluoromethylated products were isolated and characterized by NMR spectroscopy and LC-MS/MS spectrometry.^24^

**Table 4:** Scope of trifluoromethylation of unprotected peptides^*a*^

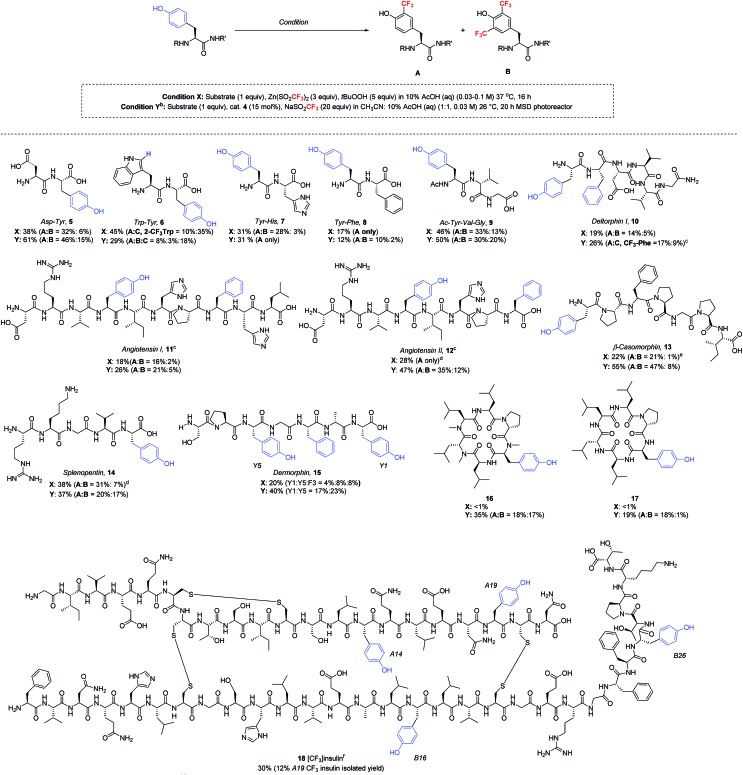

^*a*^Solution yields were determined by ^19^F-NMR using α,α,α-trifluorotoluene as internal standard in DMSO. Each entry is an average of at least two data points.

^*b*^MSD photoreactor setting: fan 4300 rpm, 470 nm, 1400 rpm (stir rate), 100% light intensity.

^*c*^Trifluoromethylation was also observed at the Phe group, yielding a mixture of regioisomers.

^*d*^Performed in pH 4 (20 mM) sodium citrate buffer.

^*e*^Performed in pH 6 MES buffer.

^*f*^Sodium trifluoromethane sulfinate (27 equiv.) was used in DMSO : 10% AcOH (aq) (3 : 2) solvent mixture.

In the absence of protecting groups, these polypeptides generally showed greater yields of trifluoromethylated products under the photoredox catalytic conditions. Lipophilic di- and tripeptides smoothly underwent trifluoromethylation under both conditions, with photocatalysis showing slightly superior yields in most cases (**5–9**). In the case of the Trp–Tyr dipeptide, in addition to the expected tyrosine trifluoromethylation products **6A/B**, the trifluoromethyl radical reacted readily with the Trp sidechain, installing the CF_3_ group at the 2-position of the indole core (3.5 : 1 = Trp : Tyr for condition X, 1.6 : 1 for condition Y). In contrast, Tyr showed superior reactivity over His and Phe in each respective dipeptide (**7** and **8**) under optimized conditions. Longer peptide chains such as deltorphin I (**10**), angiotensin I (**11**), angiotensin II (**12**) and β-casomorphin (**13**), when subjected to the trifluoromethylation conditions, gave selective modification at tyrosine, regardless of whether the Tyr residue was located at the peptide N-terminus (**10**, **13**) or at an internal position (**11**, **12**). In line with previous results, the photoredox catalytic conditions gave equivalent or superior results to the stoichiometric oxidant. Notably, the trifluoromethylation of these more complex natural products showed tolerance to His and Arg sidechains, although some modification was seen at Phe sidechains in **10–12**, and **15**.^25^ In the case of angiotensin II, conducting the reaction at pH 4 gave better stability of the Arg sidechain, which was found to be somewhat sensitive to acid. Similar to the angiotensin peptides, splenopentin (**14**) readily reacted at the C-terminal tyrosine at pH 4 to form mono-trifluoromethylated product in good yield. Further, in addition to the Arg residue, this peptide also contains a nucleophilic Lys sidechain that was well tolerated in the reaction. Dermorphin (**15**), which contains one phenylalanine and two tyrosine residues, was trifluoromethylated under stoichiometric oxidant conditions X equally at both the C-terminal (Y1) and internal (Y5) positions, along with 8% product arising from trifluoromethylation of phenylalanine. Under photocatalytic conditions, the C-terminal and internal tyrosines also exhibited similar reactivity, although the yields were somewhat higher.

Cyclic peptides have generated increasing interest as potential therapeutics due to their improved drug-like properties, including cell permeability, compared to linear peptides.^26^ Two cyclic peptides **16** and **17** recently reported by the Lokey laboratory^27^ were tested as substrates for the trifluoromethylation conditions. Importantly, both of these lipophilic cyclic peptides exhibited reactivity under photoredox catalytic conditions, while the initially optimized TFMS/TBHP conditions did not produce the desired trifluoromethylated products in significant yield (<1%). This demonstrates once again the advantage of having access to these two complementary sets of trifluoromethylation conditions for complex peptide functionalization.

As a final demonstration of the versatility of this transformation, we examined the trifluoromethylation of recombinant human insulin, which consists of two peptide chains, the 21 amino acid A chain and the 30 amino acid B chain, linked by three disulfide bonds. The peptide sequence of insulin includes four tyrosine residues (A14, A19, B16, and B26), two histidine residues (B5 and B10), three phenylalanine residues (B1, B24, and B25) and no tryptophan residues. Given the fact that insulin often forms insoluble fibrils upon vigorous agitation, and the photoredox catalytic conditions require stirring to disperse the acetonitrile and aqueous layers, we decided to proceed with the TBHP/TFMS conditions in the absence of stirring. Under these conditions, insulin was successfully trifluoromethylated at each of the four tyrosine residues with approximately 30% overall conversion as estimated by HPLC. Minor amounts of bis- and tris-CF_3_ insulin adducts were detected by LC-MS, but no reaction with histidine or phenylalanine was observed. The insulin modified at tyrosine A19 (**18**) was isolated in 12% yield by preparative HPLC. The isolated tyrosine product **18** was characterized by LC-MS/MS after DTT cleavage of the disulfide linkages.^18^

**Fig. 4:**
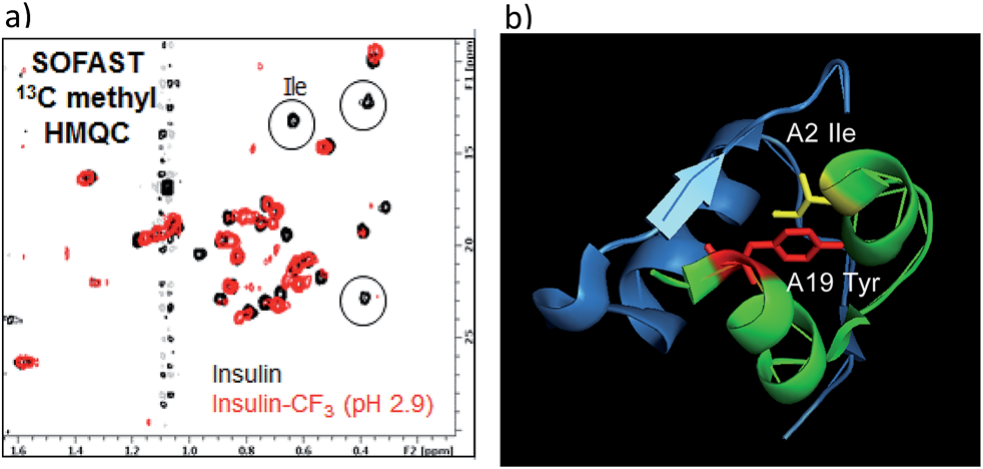
(a) Comparison of SOFAST ^13^C methyl HMQC NMR spectra of native unmodified insulin (black) and CF_3_-Tyr-A19 insulin **18** (red); (b) ribbon diagram of insulin showing proximity of Tyr-A19 and Ile-A2.

To verify whether the tyrosine A19 trifluoromethylation perturbed the structure or function of insulin, product **18** was compared to native insulin in both binding and functional assays (**18**/native insulin: IC_50_ = 1.4 nM/0.8 nM; EC_50_ = 2.2 nM/0.4 nM).^18^ Both the binding affinity and functional activity of **18** supported the conclusion that the A19-CF_3_-tyrosine–insulin maintained its structural integrity as compared with unmodified, native insulin. To further support this observation, SOFAST ^13^C methyl HMQC NMR spectroscopic analysis was conducted on both **18** and native unmodified insulin.^18^ Overlay of the 2D spectra showed a good correlation between unmodified and modified insulin samples, indicating retention of the overall solution structure. Interestingly, the insulin isoleucine A2 methyl peaks were not observed in the HMQC spectrum of **18** (Fig. 4). Since isoleucine A2 is in close proximity to tyrosine A19, we reasoned that the CF_3_-labeling on Tyr-A19 possibly induced a small change in the local conformation, altering the chemical shifts of the isoleucine methyl groups.^28^ Finally, comparison of ^19^F-NMR traces of **18** and the mixed fraction obtained by semi-preparative HPLC containing the other three CF_3_-Tyr insulin regioisomers showed well-resolved ^19^F-NMR signals for each CF_3_-modified insulin regioisomer.^18^ This observation validated the notion that CF_3_-labeling of aromatic sidechains provides a sensitive ^19^F-NMR spectroscopic probe of the local environment in complex peptides while being minimally perturbing to the overall structure of the biomacromolecule.

**Fig. 5:**
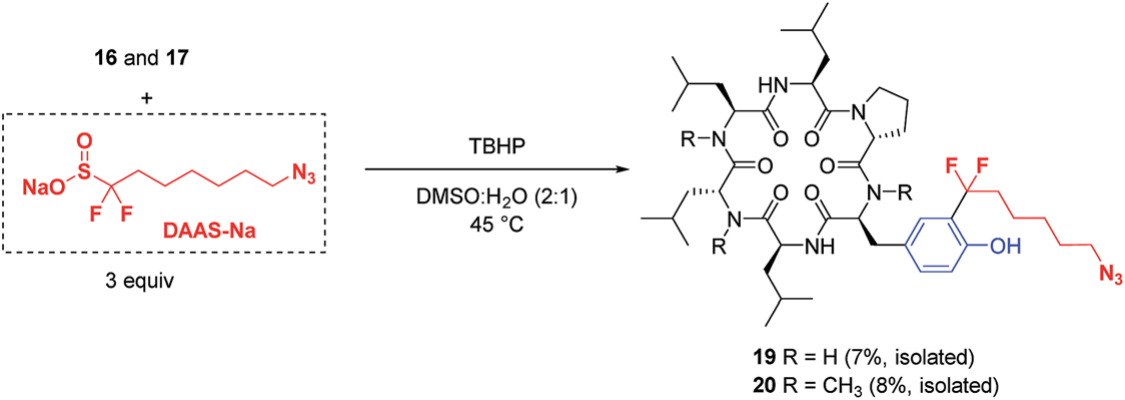
Installation of azidoalkyl groups through radical tyrosine C–H alkylation^a^ [^a^substrate (0.10 mmol), ZnCl_2_ (1.5 equiv., **16** only), DAAS-Na (3 equiv.), TBHP (5 equiv.), DMSO : H_2_O (2 : 1) at 45 °C, 20 h].

The potential applications of these mild, biocompatible radical aromatic C–H functionalization conditions go beyond CF_3_-installation. In preliminary studies, replacement of ZnTFMS with sodium azidofluoroalkylsulfinate DAAS-Na^29^ under the TBHP conditions with minimal optimization successfully installed the azidofluoroalkyl substituent on cyclic peptides **16** and **17** through reaction at the *ortho* C–H of tyrosine (Fig. 5). The corresponding azide-tagged cyclic peptides were obtained in modest isolated yields after semi-preparative HPLC.^30^ This result suggests that other functionalized fluoroalkylsulfinate precursors could be used for bioconjugation applications through radical C–H alkylation of aromatic sidechain residues.

## Conclusions

Two distinct methods have been developed that effect the radical C–H trifluoromethylation of aromatic residues in native peptides, one based on the use of stoichiometric oxidant and the other on photoredox catalysis. These mild, biocompatible conditions have enabled the direct modification of challenging peptide substrates in good yields, and demonstrated compatibility with other peptide sidechain functional groups. The results obtained with trifluoromethylated insulin suggest that this labeling approach is minimally perturbing to the overall structure of biomolecules and could be a valuable way to install spectroscopic handles into complex peptides and proteins. The extension of these approaches to larger proteins and to the installation of other tags valuable for biochemical and chemical biology studies is currently underway.

## Experimental

### General procedure for C–H trifluoromethylation of peptides with TFMS/TBHP

The peptide substrate (1 equiv.) and zinc trifluoromethanesulfinate (3 equiv.) were added to a 4 mL vial with a septum cap and brought into a N_2_ filled glovebox. Degassed 10% acetic acid in distilled water (final concentration 0.2 M solution) was added into the vial. To this solution was added TBHP (5 equiv.) in a dropwise manner, and this mixture was allowed to stand at ambient temperature for 24 h without stirring. Reactions were monitored at 220 nm by LC-MS for completion. NMR yields were determined using ^19^F-NMR with α,α,α-trifluorotoluene as an internal standard. The mixture was diluted with acetonitrile and the sample was lyophilized to remove solvents. The resultant solid was reconstituted in DMSO and purified by semi-preparative reversed-phase HPLC. Isolated compounds were characterized by NMR spectroscopy and either HRMS or LC-MS/MS spectrometry.

### General procedure for C–H trifluoromethylation of peptides with NaSO_2_CF_3_/[Ir(dFCF_3_ ppy)_2_(dtbpy)](PF_6_)

The peptide substrate (0.05–0.2 mmol, 1 equiv.), sodium trifluoromethanesulfinate (20 equiv.) and [Ir(dFCF_3_ppy)_2_(dtbpy)](PF_6_) (0.15 equiv.) were added to a 4 mL vial equipped with a stir bar, sealed with a septum cap and brought into a N_2_ filled glovebox. Degassed 10% acetic acid in distilled water : acetonitrile in 1 : 1 ratio (final concentration 0.1 M solution with respect to the substrate) was added to the vial. The vial was taken out of the glovebox and the reaction was irradiated in the MSD photoreactor^23^ at ambient temperature for 24 h. Reactions were worked up and analyzed as described in the previous example.

## Conflicts of interest

There are no conflicts to declare.

## Supplementary Material

Supplementary information
