# Supplementary material for: Protecting group free radical C–H trifluoromethylation of peptides
Source: Chem Sci. 2018 Apr 10;9(17):4168–75. doi: 10.1039/c8sc00368h (PMC5941281; doi:10.1039/c8sc00368h)

Electronic Supplementary Material (ESI) for Chemical Science.
This journal is © The Royal Society of Chemistry 2018

Protecting Group Free Radical C-H Trifluoromethylation of Peptides

Naoko Ichiishi[a], John P. Caldwell[b], Melissa Lin[c], Wendy Zhong[c], Xiaohong Zhu[b], Eric Streckfuss[d], Hai-Young Kim[c], Craig A. Parish[e,*], Shane W. Krska[a,*]

[a]*Chemistry Capabilities and Screening, MSD Sharp & Dohme Corp., Kenilworth, New Jersey 07033, United States.*
[b]*Kenilworth Discovery Chemistry, MSD Sharp & Dohme Corp., Kenilworth, New Jersey 07033, United States.*
[c]*Analytical Research & Development, MSD Sharp & Dohme Corp., Rahway, NJ, United States.*
[d]*West Point Discovery Chemistry, MSD Sharp & Dohme Corp., West Point, Pennsylvania 19486, United States*
[e]*Discovery Chemistry Modalities, MSD Sharp & Dohme Corp., Kenilworth, New Jersey 07033, United States*

Table of Contents

I.	General Procedures	S-2
II.	Materials	S-4
III.	Experimental Details for Table 1	S-5
IV.	Additional Optimization and Mechanistic Studies	S-5
V.	Experimental Procedure for Photoredox Catalysis	S-8
VI.	Photocatalyst Screening Using High Throughput Experimentation Method	S-8
VII.	Solvent Screen for Photocatalytic System	S-10
VIII.	Terminal Oxidant Screen	S-12
IX.	Met Residue Oxidation under TBHP/$Zn(SO_2CF_3)_2$ Condition (Condition X & Y)	S-13
X.	Synthesis of Trifluoromethylated Products: Procedures and Characterization	S-18
	a. Synthesis of trifluoromethylated amino acids	S-18
	b. Synthesis of trifluoromethylated peptides	S-25
	c. Synthesis of azido-containing cyclic peptides	S-50
XI.	Insulin Assays	S-52
XII.	References	S-53
XIII.	Spectroscopic Data for Characterized Compounds	S-54

I. General Procedures

Unless otherwise noted, all reactions were conducted under inert atmosphere (N_2). Reactions were monitored using a Waters Acquity UPLC system with UV detection at 220, 254 or 280 nm and a low resolution electrospray ionization mode (ESI) mass spectrometer. Yields were obtained through ^{19}F-NMR using α,α,α-trifluorotoluene (Aldrich) as an internal standard.

NMR Spectroscopy. All NMR spectra were recorded on a Bruker spectrometer (500 MHz) at ambient temperature. ^{1}H-NMR chemical shifts are reported as δ in units of parts per million (ppm) relative to methanol-d_4 (δ 3.31, quintet) or dimethylsulfoxide-d_6 (δ 2.50, quintet). Multiplicities are reported as follows: s (singlet), d (doublet), t (triplet), dd (doublet of doublets), br (broad) or m (multiplet). Coupling constants are reported as a *J* value in Hertz (Hz). The number of protons (*n*) for a given resonance is indicated as *n*H and is based on spectral integration values. ^{13}C-NMR chemical shifts are reported as δ in units of parts per million (ppm) relative to methanol-d_4 (δ 49.2, septet) or dimethylsulfoxide-d_6 (δ 39.5, septet). ^{19}F-NMR chemical shifts are reported as δ in units of parts per million (ppm).

Semi-preparative HPLC. Semi-preparative reverse-phase HPLC of the modified amino acids (**2, 3, S1-S9**) was performed using a Waters SunFire C18 preparative column (19 x 100 mm). Gradients for each amino acid product are detailed in the experimental procedures along with LCMS traces of final products. Semi-preparative reverse phase HPLC of peptides (**5-9, 16-17**) was accomplished using a Gilson HPLC equipped with a Waters SunFire Prep C18 stationary phase. Semi-preparative reverse-phase HPLC of the modified peptides (**10-14, S10-S23**) was accomplished using a Charged Surface Hybrid (CSH) stationary phase (Waters) and H_2O:acetonitrile with 0.16% TFA gradient mobile phase with the gradients for each peptide detailed in the experimental procedures.

High resolution mass spectrometry measurements:

For compounds **2, 3, S1-S9, 5A-9A:**

High-resolution MS for small molecules (i.e., compound **2, 3, S1-S9, 5A-9A**) was obtained on a Waters Synapt G1 with an ESI source, equipped with Waters UPLC CORTECS C18+ (100 x 2.1 mm, 1.6 μm particle size) at a flow rate of 0.3 mL/min using gradiant flow: 40-99% 0.1% formic acid (FA) in acetonitrile/0.1% FA in water over 7 minutes (column temperature = 50°C).

High resolution mass spectrometry/mass spectrometry measurements:

For compounds **11A , 12A S14-17, S20-23**:

High resolution LC/MS/MS analysis was performed on a Waters Xevo G2-XS Q-TOF mass spectrometer (Waters, Milford, MA) coupled to an I-Class Waters Acquity UPLC system, operated with electrospray ionization in positive-ion mode. The tandem MS/MS analysis of all the compounds was performed on the same Waters Xevo G2-XS MS instrument in the MS/MS mode. The collision energy was 15 eV for all analytes and standards. Calibration was achieved using sodium formate, and leucine enkephalin (m/z of 556.2772 $[M+H]^+$) was used as the lock mass for accurate mass measurements. LC gradient conditions are described as below: the gradient started at 0% B, and linearly increased to 100% B in 15 min at a flow rate of 200 µL/min. The sample was eluted using a Water's Acquity™ UPLC® BEH C18, 2.1 x 100 mm column with 1.7 µm particle size(column temperature = 30 °C). Mobile phase compositions were: solvent A: 0.1% FA in H_2O, solvent B: 0.1% FA in CH_3CN. The sample was dissolved in 0.1% formic acid in 1/1 H_2O/CH_3CN at a concentration of 0.2 mg/mL with an injection volume of 2 µL.

For compounds **10A, S10, S11, S12, S13, S18, 13A, 13B, S19**:

LC/MS/MS experiments were performed using a Thermo LTQ™-Orbitrap mass spec and a Water's Acquity™ UPLC® system. The high resolution LC/MS spectra were acquired in FTMS mode using an Orbitrap instrument at a resolution of 30,000 (at m/z 400). The electrospray ionization needle was held at 4.5 kV, and a nitrogen sheath gas and a nitrogen auxiliary gas were used to stabilize the spray. The heated capillary was set at 275 °C. All MS/MS experiments were conducted in the linear ion trap. Helium was introduced into the ion trap to improve the trapping efficiency and also to serve as the collision gas for CID. The operational pressure after introducing helium was $\sim 2 \times 10^{-5}$ Pa in the linear ion trap.

LC gradient conditions are described as below: The gradient started at 5% B and was heldfor 0.5 min, before increasing to 99% B in 9.5 min at a flow rate of 300 µL/min (column temperature = 30 °C). Mobile phase compositions were: solvent A: 0.1% FA in H_2O, solvent B: 0.1% FA in CH_3CN. The sample was eluted using a Water's Acquity™ UPLC® BEH C18, 2.1 x 150 mm column with 1.7 µm particle size. Modified peptides were dissolved in H_2O at the concentration of 1 mg/mL with an injection volume of 2 µL.

For modified CF_3[Y] insulin:

DTT was used to break all disulfide bonds. The procedure was: 5 μL of 1 M DTT in water was added to 50 μL of 1 mg/ml of the intact protein sample in H_2O. The solution was incubated for 30 min at 60 °C. The reaction mixtures were injected directly into the LC/MS system. The sample was diluted 100x in 50/50 0.1% FA H_2O/CH_3CN and directly infused into the mass spectrometer *via* a TriVersa NanoMate robot (Advion, Inc., Ithaca, NY, USA).

MS/MS experiments were carried out using a 9.4-T solariX qQq-Fourier transform ion cyclotron resonance (ICR) mass spectrometer (Bruker Daltonics, Billerica, MA). Argon was used as the collision gas. Each spectrum contains 2 M data points and was generated by summing over 10-100 individual spectra depending on the signal quality. All spectra were analyzed using the DataAnalysis software (Bruker Daltonics, Billerica, MA).

II. Materials

Unless otherwise noted all materials were obtained from commercial suppliers and used without further purification. Anhydrous organic solvents were purchased from Sigma Aldrich in SureSeal™ bottles. Concentrated acetic acid was purchased from Sigma Aldrich and degassed before further use. All *N*-acyl amino acid amides, deltorphin I (**10**), angiotensin I (**11**), angiotensin II (**12**), β-casomorphin (**13**), splenopentin (**14**), and dermorphin (**15**) were purchased from Bachem. Cyclic peptides **16** and **17** were prepared by BioPeptek. Zinc trifluoromethylsulfinate, α,α,α-trifluorotoluene, d_4-HOAc and all pH buffers were purchased from Sigma Aldrich. Sodium trifluoromethylsulfinate was purchased from Oakwood Products. Recombinant human insulin was prepared by Diosynth. 2-Hydroperoxy-2-methylpropane (TBHP, trade name Luperox® TBH70X as a 70 weight% in water) was purchased from Alfa Aesar. Deuterated solvents D_2O, d_6-DMSO, and d_4-methanol were purchased from Cambridge Isotope Laboratories. PVDF membrane filters were obtained from Millipore. Lumidox™ blue LED arrays for a 24- and 96 well-plate reactors were purchased from Analytical Sales and Services, Inc. The MSD photoreactor was fabricated in house; detailed information about the instrument has been published previously.[1]

III. Experimental Details for Table 1

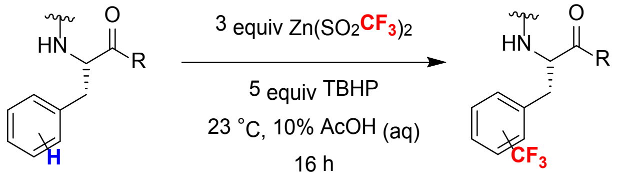

General Procedure. The substrate (1 equiv) and zinc trifluoromethanesulfinate (3 equiv) were added to a 4 mL vial with a septum cap and brought into an N_2-filled glove box. Degassed 10% acetic acid in distilled water (final concentration 0.2 M) was added into the vial. To this solution was added TBHP (5 equiv) in a dropwise manner, and this mixture was allowed to stand at 23 °C for 16 h. The reactions were monitored at 220 nm and 280 nm by LC/MS. Solution yields were determined using ^{19}F-NMR spectroscopy with α,α,α-trifluorotoluene as an internal standard. The mixture was then diluted with acetonitrile, transferred to a separate flask, and the solvents were removed by lyophilization. The resulting solid was reconstituted in DMSO and purified by semi-preparative reverse phase HPLC. Fractions were collected and solvents were removed by lyophilization.

IV. Additional Optimization and Mechanistic Studies

a. Solvent Screen

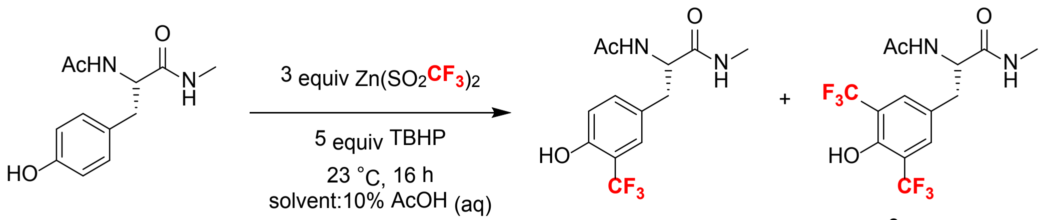

1 2 3

Entry	Solvent	^{19}F-NMR (%) 2+3	^{19}F-NMR (%) 2	^{19}F-NMR (%) 3
1	H_2O	19	4	15
2	10% AcOH (aq)	63	41	22
3	DMSO:10% AcOH (aq)	49	41	8
4	DCM: 10% AcOH (aq)	36	22	14
5	Acetone: 10% AcOH (aq)	61	27	24
6	DMSO:10% AcOH (aq) (1:1)	40	9	31
7	DMSO:10% AcOH (aq) (1:3)	61	26	35

b. Slow Addition of TBHP

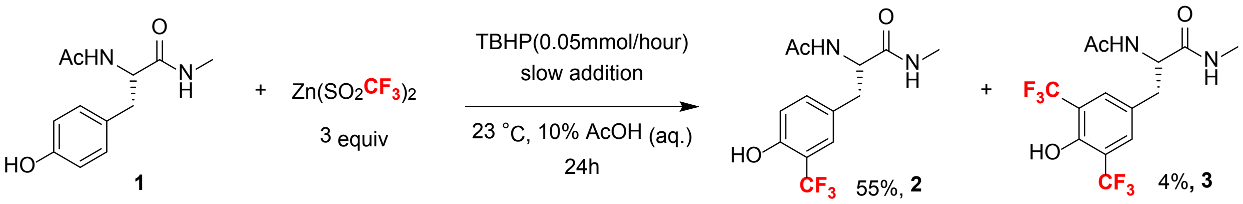

(S)-2-Acetamido-3-(4-hydroxyphenyl)-*N*-methylpropanamide (24 mg, 0.1 mmol) (**1**) and zinc trifluoromethansulfinate (133 mg 0.3 mmol, 3 equiv) were charged in a 4 mL vial. The mixture was transferred into a N_2-filled glove box and 206 μL of 10% AcOH (aq) was added. To a separate 4 mL vial, a 70% solution of TBHP in water (137 μL, 1.0 mmol, 5 equiv) was diluted with 492 μL of 10% AcOH and withdrawn into a 1-mL plastic syringe. The vial was sealed and these mixtures were removed from the glove box and the TBHP solution was added to the reaction at a rate of 0.05 mmol/hour using a syringe infusion pump (KD Scientific). The resultant reaction mixture was analyzed by ^{19}F-NMR using CF_3Ph in d_6-DMSO as an internal standard. By this method the yield of product **2** was determined to be 55% along with 4% of bis(trifluoromethylated) product **3**.

c. Observation of Fluoroform

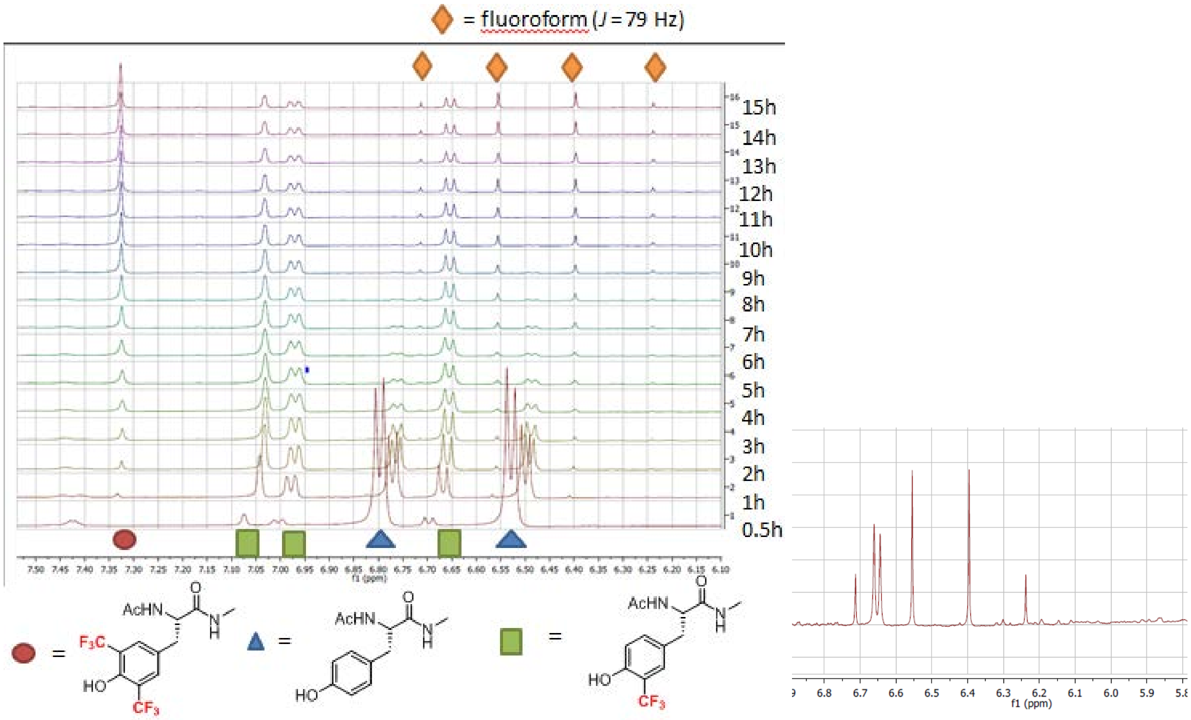

(S)-2-Acetamido-3-(4-hydroxyphenyl)-*N*-methylpropanamide (Ac-Tyr-NHMe, 28 mg, 0.12 mmol) (**1**) and zinc trifluoromethansulfinate (160 mg 0.36 mmol, 3 equiv) were charged to a J-Young NMR tube. In a N_2-filled glove box 600 µL of 10% AcOH in D_2O was added, followed by TBHP (84 µl, 0.6 mmol, 5 equiv) via syringe. The NMR tube was sealed with an air-tight screw cap and was taken out from the glove box. The reaction was monitored by ^{1}H-NMR (Bruker, 500 MHz, delay time = 20 seconds, 64 scans) every hour for 15 h. The formation of fluoroform (*J* = 79 Hz) was observed over 15 h by ^{1}H-NMR. Baran and coworkers previously reported the formation of fluoroform under similar reaction conditions and its observation by ^{19}F-NMR.[2]

V. Experimental Procedure for Photoredox Catalysis

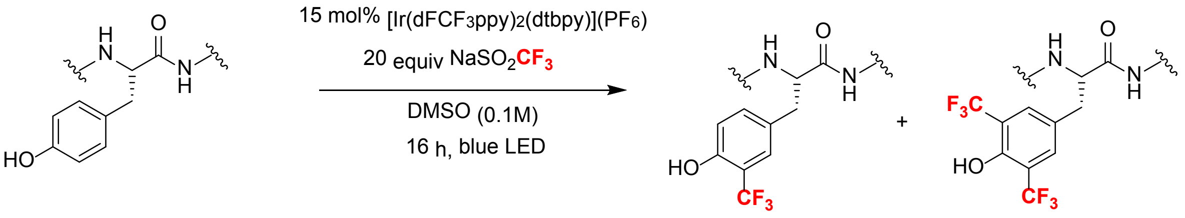

General Procedure: A substrate (0.05-0.2 mmol, 1 equiv), sodium trifluoromethanesulfinate (20 equiv) and [Ir(dFCF$_3$ppy)$_2$(dtbpy)](PF$_6$) **4** (0.15 equiv) were added to a 4 mL vial equipped with a stir bar and sealed with a septum cap and brought into a N_2-filled glove box. A 1:1 mixture of degassed 10% acetic acid in distilled water:acetonitrile (final concentration 0.1 M solution with respect to tyrosine) was added into the vial. The vial was removed from the N_2-filled glove box, the reaction was irradiated with the MSD photoreactor[1], and the mixture was allowed to stand at ambient temperature for 16 h. The reactions were monitored at either 254 or 280 nm by LC-MS. NMR yields were determined using ^{19}F-NMR with α,α,α-trifluorotoluene as an internal standard. The mixture was diluted with 1:1 acetonitrile:water and the sample was lyophilized to remove all solvents. The resultant solid was reconstituted in DMSO and purified by semi-preparative reverse phase HPLC. The isolated peaks were characterized by HRMS-LC/MS/MS, and by ^{1}H, ^{19}F, and ^{13}C-NMR (^{13}C-NMR are available for compounds isolated in sufficient quantity).

VI. Photocatalyst Screening using High Throughput Experimentation Method

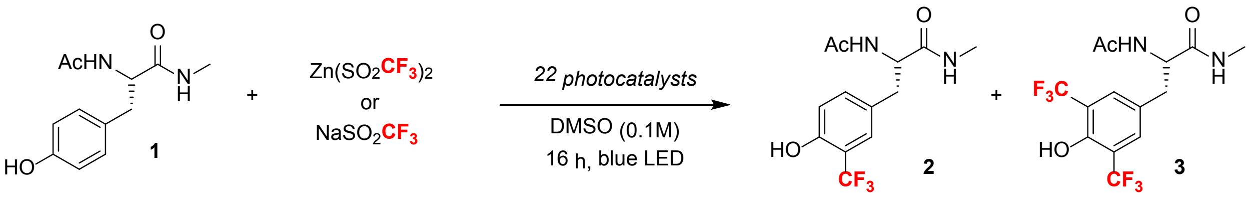

All photocatalysts (0.5 μmol, 5 mol%) were pre-dispensed in 750 μL clear glass shell vials (Analytical Services; catalog# 84001) equipped with a parylene-coated stir bar (V&P Scientific; catalog# VP711-1). A total of 48 vials were placed in a 96-well photoredox reaction plate (Analytical Sales and Services, catalog# 96973). Stock solutions of Ac-Tyr-NHMe in DMSO (0.2M) and trifluoromethylsulfinate salts in DMSO (0.8M) were prepared. To the pre-loaded vials, Ac-Tyr-NHMe (50 μL, 10 μmol, 1 equiv) and corresponding sulfinate solutions (100 μL, 80 μmol, 8 equiv) were added via multichannel pipettes in a N_2-filled glove box. The plate was sealed tightly, placed in a tumble stirrer with the Lumidox 96-well Blue LED array (Analytical sales & Services; catalog # LUM96B) in between the plate and stirrer, and irradiated for 16h (30 mA). The plate was quenched with 500 μL of quenching solution (0.02 M 4,4'-di-*tert*-butylbiphenyl in DMSO:CH_3CN:AcOH = 7:2.5:0.5) and stirred for 5 minutes. To a 2 mL UPLC

wellplate, 700 µL of CH_3CN was added to each well and a 10 µL aliquot of each quenched crude reaction mixture was added to the corresponding location. The samples were analyzed by LC-MS using 4,4'-di-*tert*-butylbiphenyl as an internal standard. Solution yields of **2** and **3** are shown under the corresponding photocatalyst.

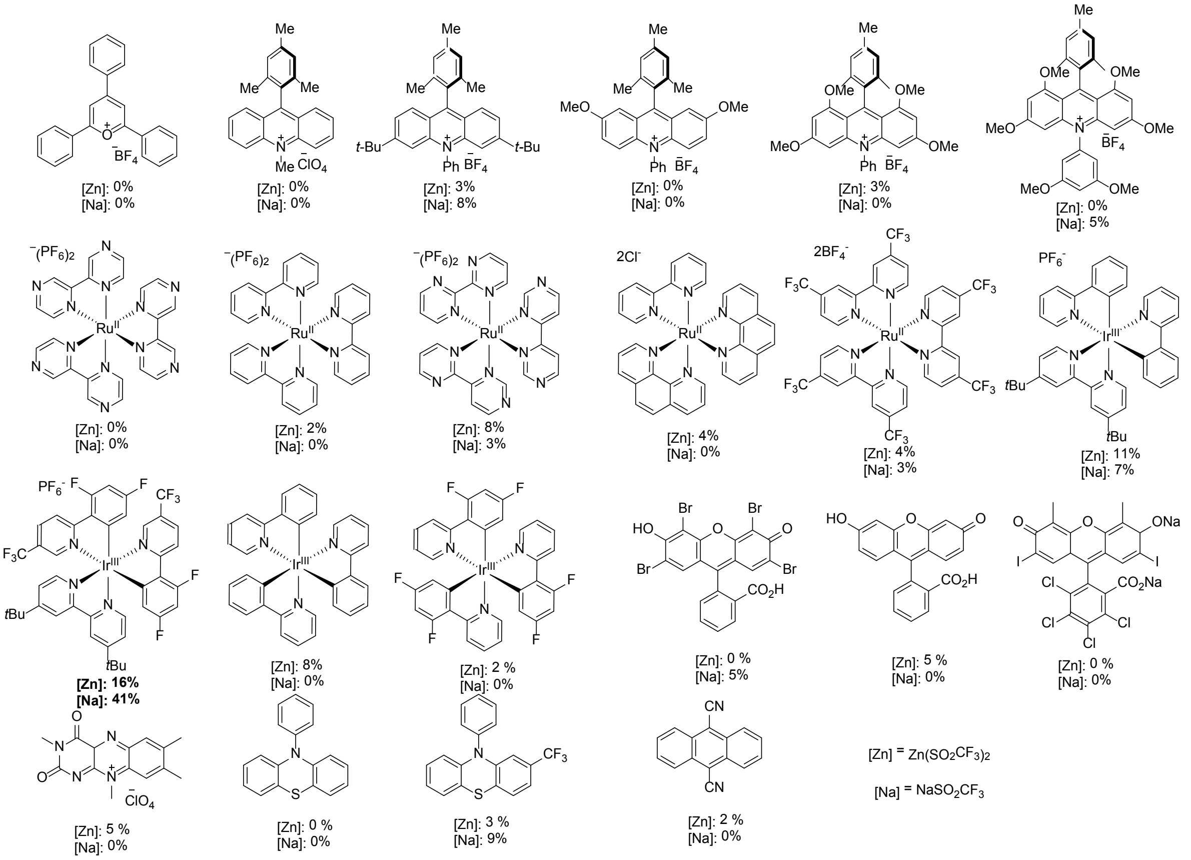

VII. Solvent Screen for Photocatalytic System

a. Organic solvent screen

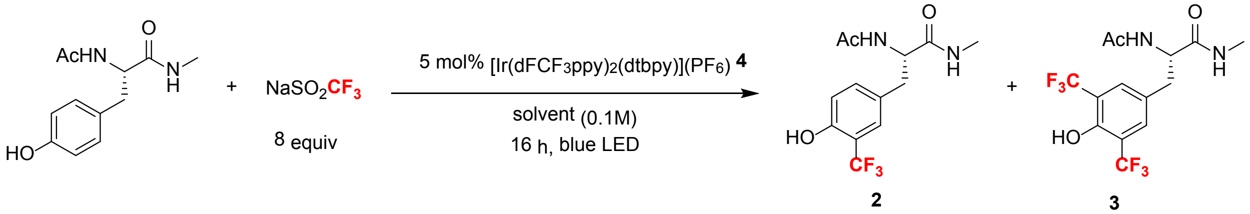

To 750 μL clear glass shell vials, 100 μL of [Ir(dFCF$_3$ppy)$_2$(dtbpy)](PF$_6$) (cat. **4**, 5 mM in CH_3CN, 0.5 μmol, 5 mol%) were placed in a 96-well photoredox reaction plate. Stock solutions of Ac-Tyr-NHMe in MeOH (0.2 M) and trifluoromethylsulfinate salts in MeOH (0.8 M) were prepared. To the reaction vials Ac-Tyr-NHMe (50 μL, 10 μmol, 1 equiv) and corresponding sulfinate solutions (100 μL, 80 μmol, 8 equiv) were added via multichannel pipettes in a N_2-filled glove box. Solvents were evaporated via Genevac. Each vial in the plate was reconstituted to a total of 100 μL with selected solvents (0.1 M, see graphs below), sealed tightly and placed in a tumble stirrer with the Lumidox 96-well Blue LED array in between the plate and stirrer and irradiated for 16 h (30 mA). The plate was quenched with 500 μL of quenching solution (0.02 M 4,4'-di-*tert*-butylbiphenyl in DMSO:CH_3CN:AcOH = 7:2.5:0.5) and stirred for 5 minutes. To a 2 mL UPLC wellplate, 700 μL of CH_3CN was added to each well and a 10 μL aliquot of each quenched crude reaction mixture was added to the corresponding location. The samples were analyzed by ^{19}F-NMR to determine solution yields.

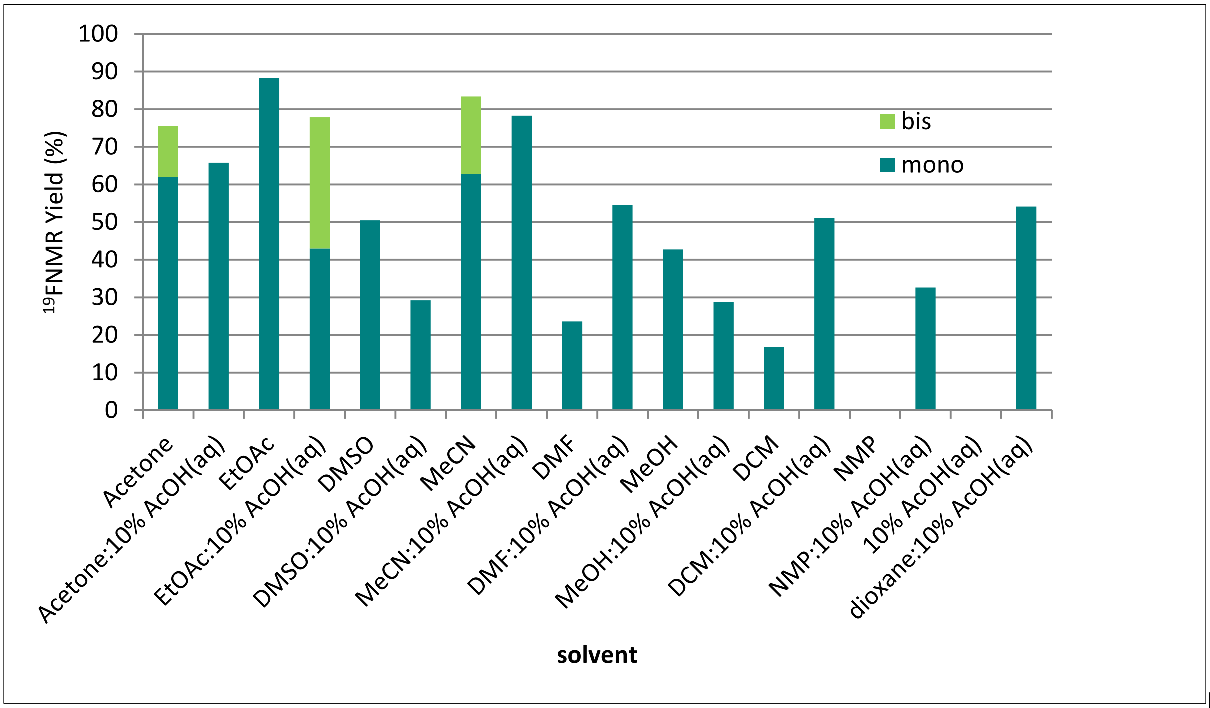

bis compound **3**, mono = compound **2**

b. Aqueous buffer screen

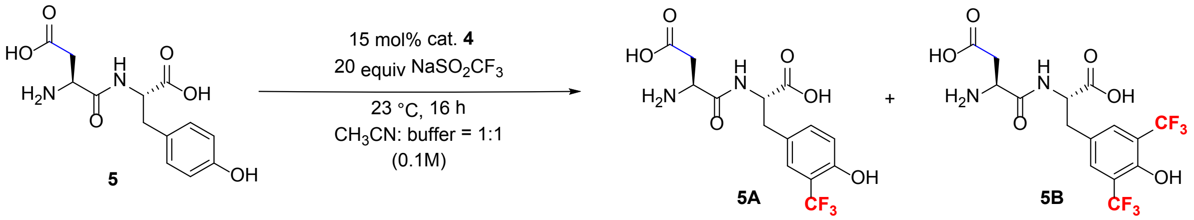

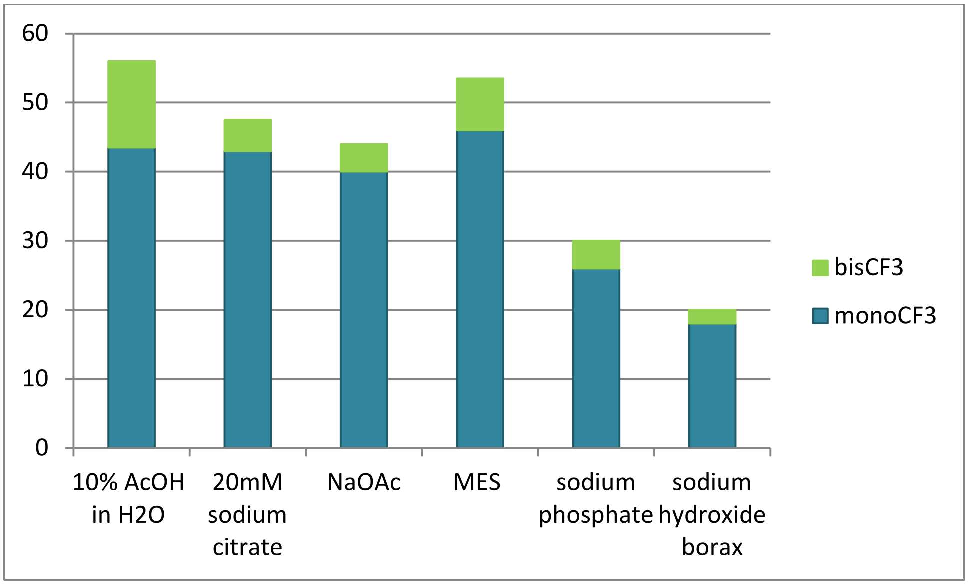

bis CF_3 = compound **5B**, monoCF_3 = compound **5A**

pH	Buffer
2.2	10% AcOH in H_2O
4.0	20 mM sodium citrate (aq)
5.5	160 mM NaOAc
6.0	0.1 M 2-(*N*-Morpholino acid)ethanesulfonic hemisodium salt (MES)
7.0	100 mM sodium phosphate
10	30 mM NaOH and borax

VIII. Terminal Oxidant Screen

In a N_2-filled glove box, **5** (0.05 mmol, 15.0mg), photocatalyst (15 mol%) and $NaSO_2CF_3$ (156 mg, 20 equiv) were added to a 4 mL singleton vial with a magnetic stir bar. A 1:1 mixture of acetonitrile:10% AcOH (aq), followed the selected oxidant (1 equiv), were added and the vial was sealed with a septum cap. The reaction was stirred and irradiated with blue LED (400 nm, ~3W/reaction) for 16 h. The samples were analyzed by ^{19}F-NMR to determine solution yields. Based on the proposed mechanism, we hypothesized that the use of a terminal oxidant may result in an acceleration of the oxidation of Ir(II) catalyst to Ir(III), thus rendering an increase in the overall rate of reaction. There are examples described in the literature where addition of terminal oxidants enhanced overall product yields.[3] However, in this case, stoichiometric oxidants generally led to lower overall product yields, perhaps due to side-reactions with the unprotected peptide functional groups.

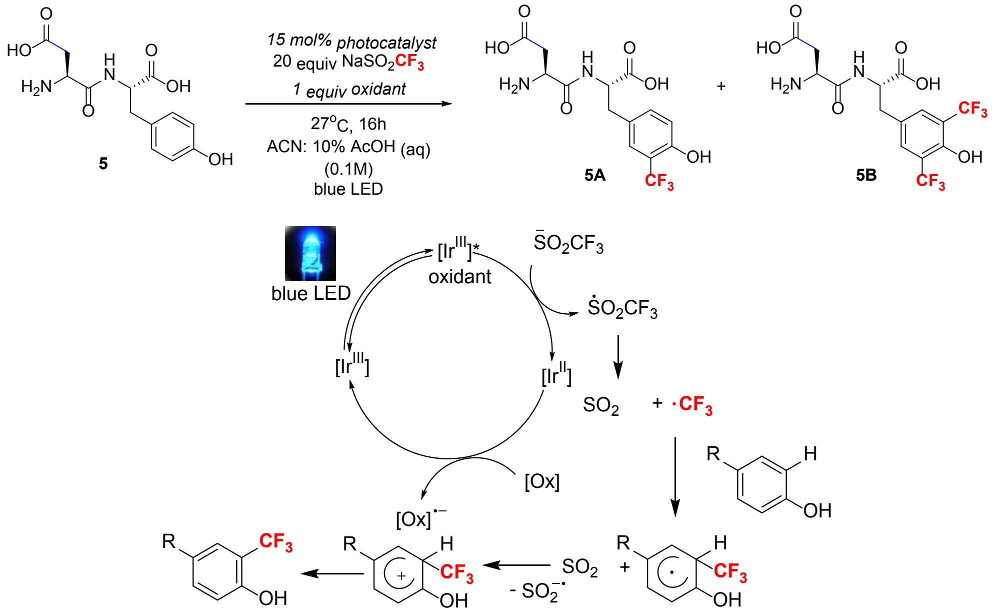

Oxidant	$[Ir(ppy)_2(dtbpy)](PF_6)$		$[Ir(dFCF_3ppy)_2(dtbpy)](PF_6)$ (4)	
	5A	5B	5A	5B
$(NH_4)_2S_2O_8$	20 %	3%	11 %	4%
Nitrobenzene	Not determined	Not determined	30%	6%
*t*BuOOH	26%	4%	15%	6%
Air[a]	23%	4%	21%	13%
None	42%	4%	46%	15%

[a]Reactions were set up in ambient atmosphere without purging with N_2.

IX. Stability of Met Residue under Trifluoromethylation Conditions (Condition X & Y)

Condition X: Under these conditions, methionine in NH_2-Met-Tyr-OH was oxidized to the corresponding sulfoxide in the presence of stoichiometric TBHP. In addition, trifluoromethylation occurred as expected and afforded the yields shown below.

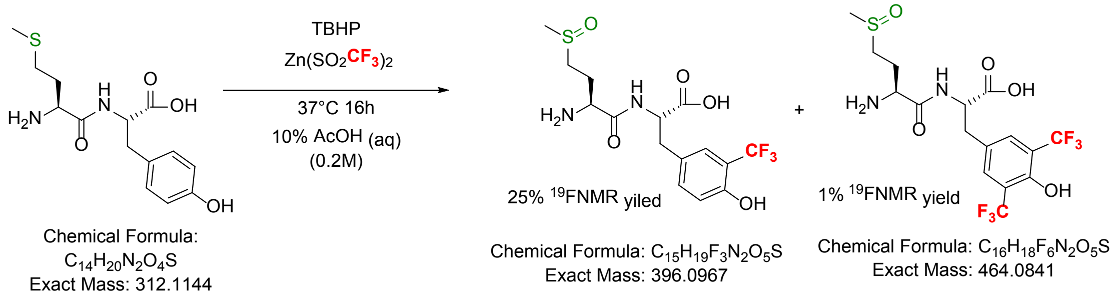

LC-MS Trace

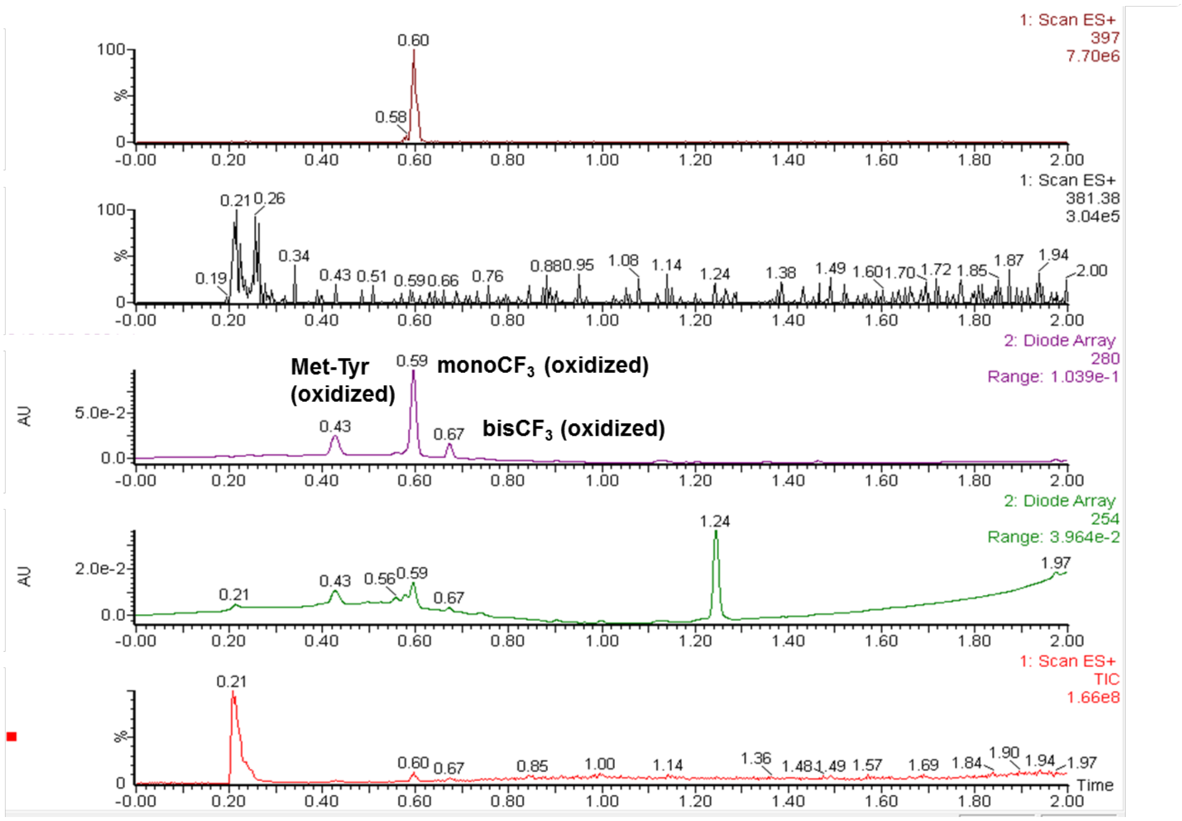

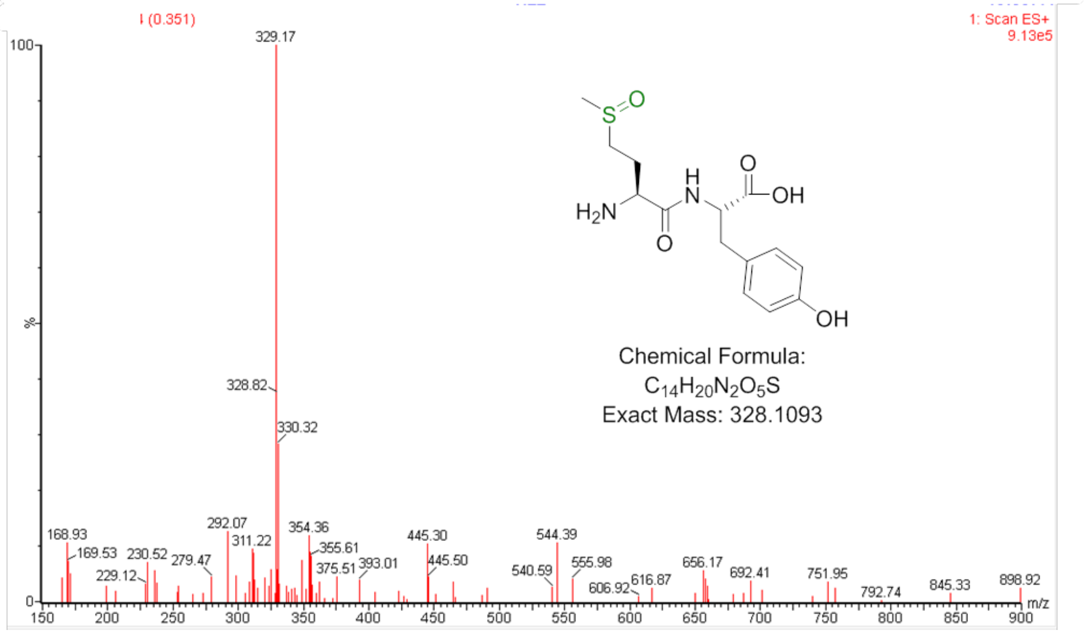
(0.351)
1: Scan ES+
9.13e5
100
%
0
329.17
328.82
330.32
168.93
169.53
230.52
229.12
279.47
292.07
311.22
354.36
355.61
375.51
393.01
445.30
445.50
540.59
544.39
555.98
606.92
616.87
656.17
692.41
751.95
792.74
845.33
898.92
m/z
150
200
250
300
350
400
450
500
550
600
650
700
750
800
850
900
S
O
H2N
H
N
O
OH
O
OH
Chemical Formula:
C14H20N2O5S
Exact Mass: 328.1093

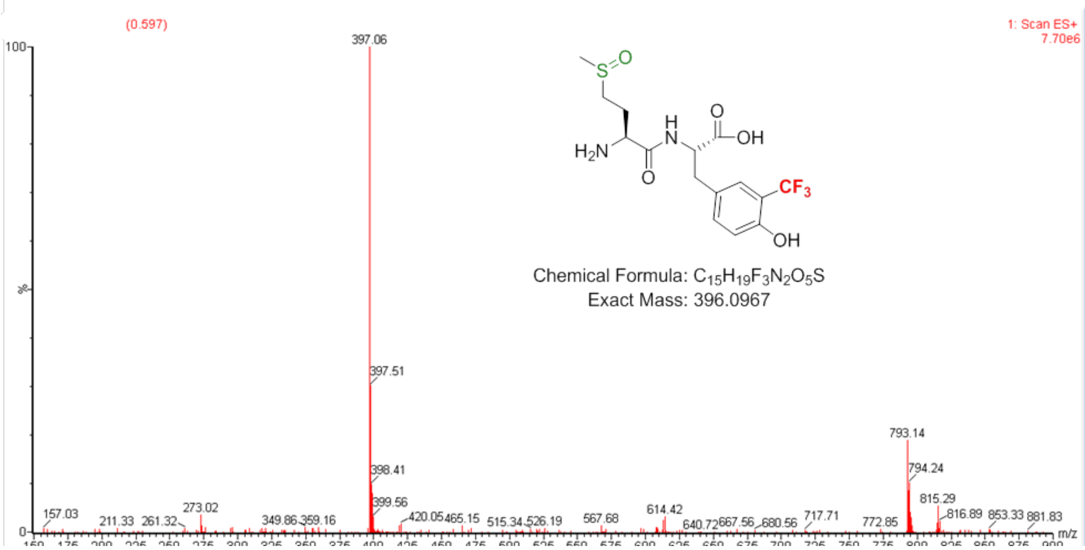
(0.597)
1: Scan ES+
7.70e6
100
%
0
397.06
397.51
398.41
399.56
157.03
211.33
261.32
273.02
349.86
359.16
420.05
465.15
515.34
526.19
567.68
614.42
640.72
667.56
680.56
717.71
772.85
793.14
794.24
815.29
816.89
853.33
881.83
m/z
S
O
H2N
H
N
O
OH
O
CF3
OH
Chemical Formula: C15H19F3N2O5S
Exact Mass: 396.0967

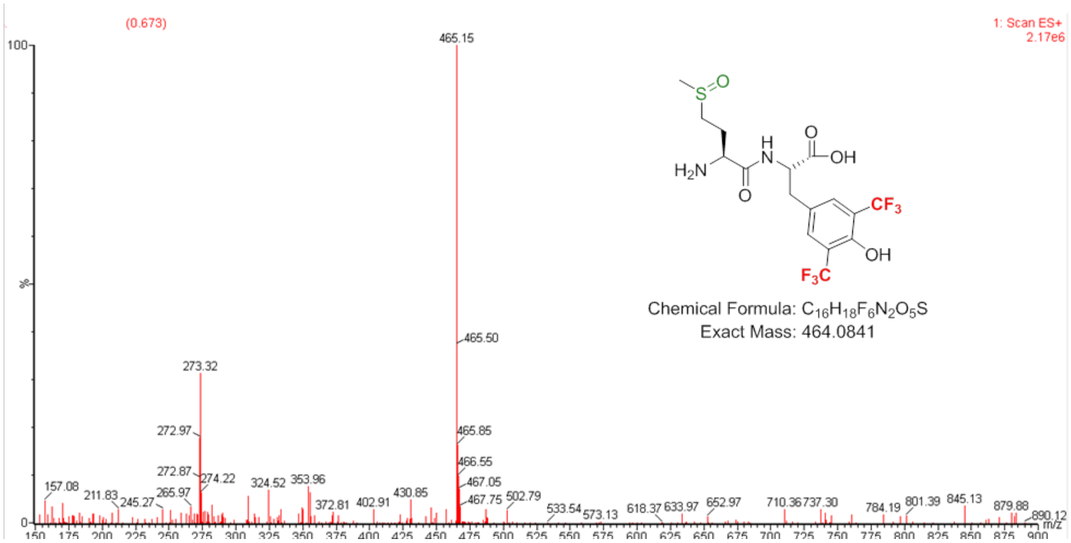

Condition Y: Conversely, under photoredox conditions, oxidation of the methionine thioether in NH_2-Met-Tyr-OH was observed to only a minor extent and the desired products were afforded in yields that were analogous for other dipeptide substrates.

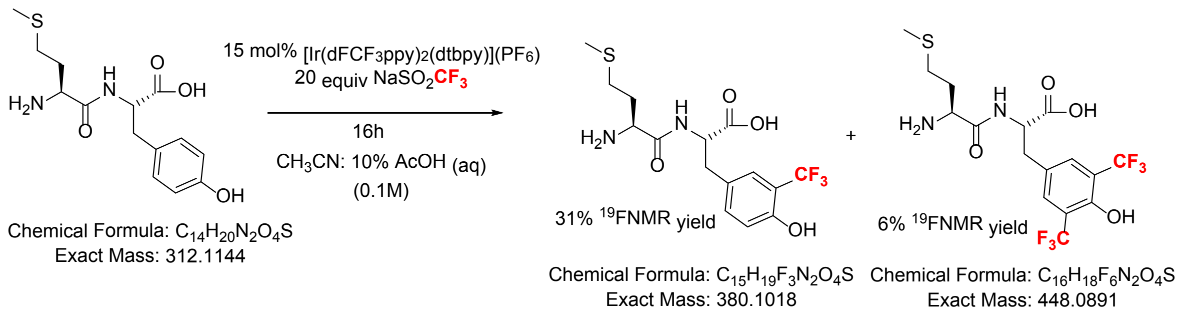

LC-MS Trace

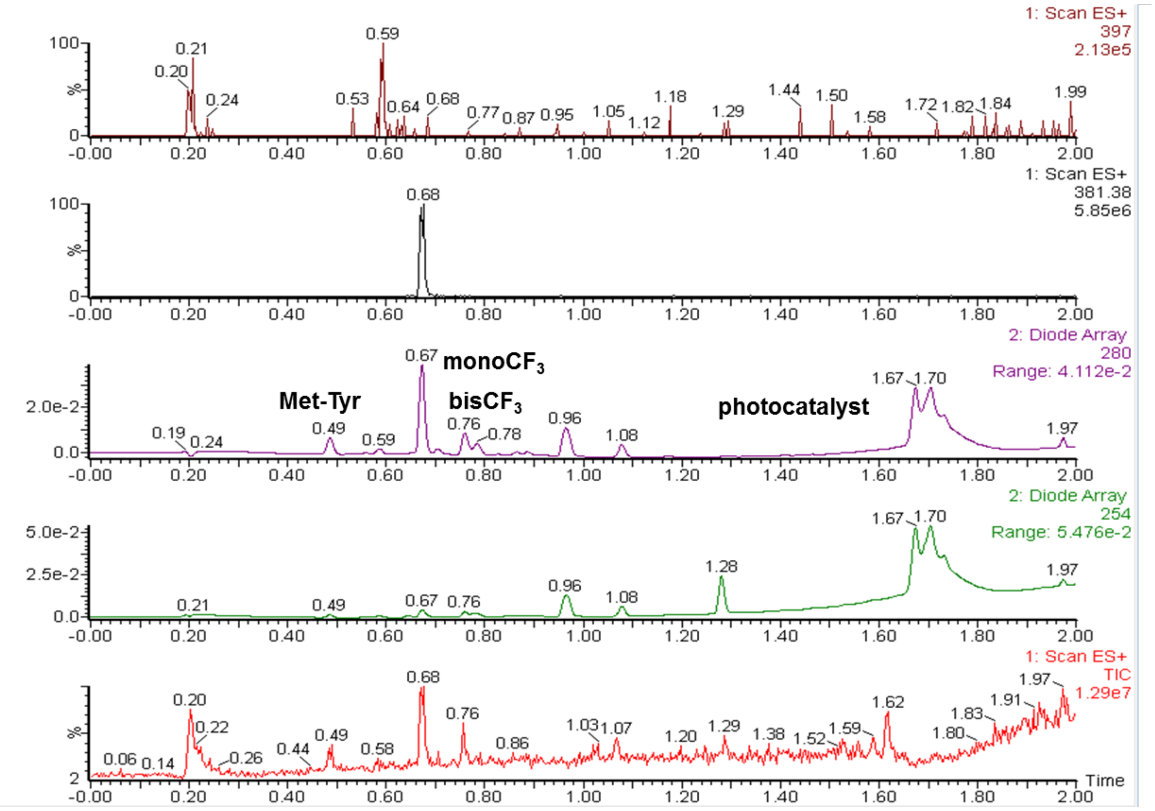

1: Scan ES+
397
2.13e5
1: Scan ES+
381.38
5.85e6
2: Diode Array
280
Range: 4.112e-2
Met-Tyr
monoCF3
bisCF3
photocatalyst
2: Diode Array
254
Range: 5.476e-2
1: Scan ES+
TIC
1.29e7
Time

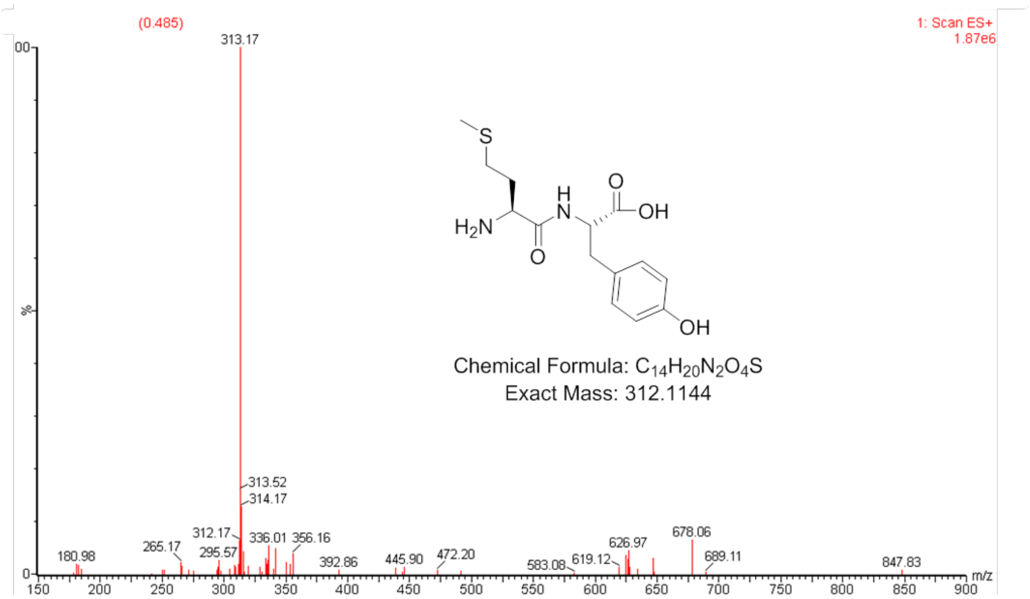

(0.485)
1: Scan ES+
1.87e6
313.17
m/z
Chemical Formula: C14H20N2O4S
Exact Mass: 312.1144

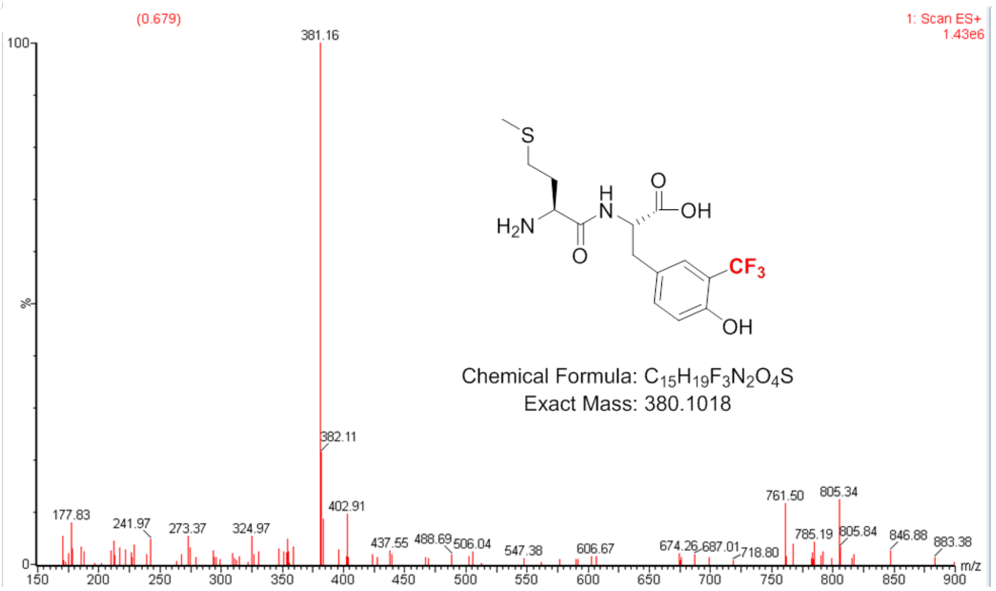
(0.679)
381.16
1: Scan ES+
1.43e6
100
%
0
H2N
S
H
N
O
OH
O
CF3
OH
Chemical Formula: C15H19F3N2O4S
Exact Mass: 380.1018
382.11
402.91
177.83
241.97
273.37
324.97
437.55
488.69
506.04
547.38
606.67
674.26
687.01
718.80
761.50
805.34
785.19
805.84
846.88
883.38
m/z
150
200
250
300
350
400
450
500
550
600
650
700
750
800
850
900

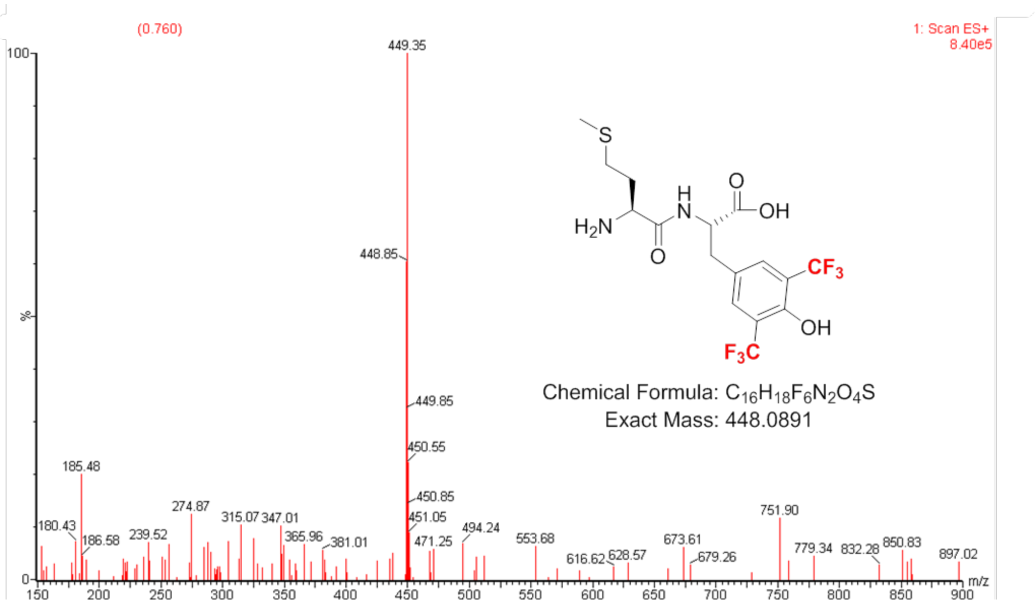
(0.760)
449.35
1: Scan ES+
8.40e5
100
%
0
H2N
S
H
N
O
OH
O
CF3
OH
F3C
Chemical Formula: C16H18F6N2O4S
Exact Mass: 448.0891
448.85
449.85
450.55
450.85
451.05
471.25
185.48
180.43
186.58
239.52
274.87
315.07
347.01
365.96
381.01
494.24
553.68
616.62
628.57
673.61
679.26
751.90
779.34
832.28
850.83
897.02
m/z
150
200
250
300
350
400
450
500
550
600
650
700
750
800
850
900

X. Synthesis of Trifluoromethylated Products and Characterizations

a. Simple Aromatic Amino Acids

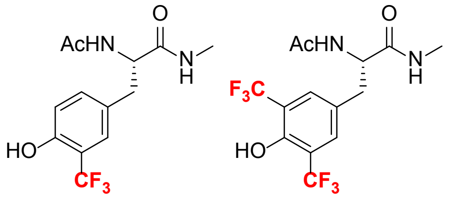

2 **3**

*(S)-2-acetamido-3-(4-hydroxy-3-(trifluoromethyl)phenyl)-N-methylpropanamide (**2**) and (S)-2-acetamido-3-(4-hydroxy-3,5-bis(trifluoromethyl)phenyl)-N-methylpropanamide (**3**):* (S)-2-acetamido-3-(4-hydroxyphenyl)-*N*-methylpropanamide (47.3 mg, 0.2 mmol) (**1**) and zinc trifluoromethansulfinate (199 mg, 0.6 mmol, 3 equiv) were charged in a 4 mL vial. The mixture was transferred into a N_2-filled glove box and 1 mL of 10% AcOH (aq) (0.2 M) was added. To this solution was added a 70% solution of TBHP in water (137 µL, 1.0 mmol, 5 equiv) in a dropwise manner. The vial was sealed and the mixture was allowed to stand at 23 °C for 16 h. The reaction mixture was lyophilized and reconstituted into DMSO. The resulting solution was purified by preparative HPLC purified using a Waters SunFire C18 preparative column (19 x 100 mm) with an 8 min run time (25 mL/min flow) and a gradient of 90:10 to 55:45 water:acetonitrile with 0.16% TFA. The fractions containing the desired products were combined and lyophilized under reduced pressure to afford 13.1 mg (21%) of mono-trifluoromethylated product **2** and 22.6 mg (29.7%) of bis-trifluormethylated product **3**.

2: ^{1}H-NMR (600 MHz, methanol-d_4) δ 7.35 (d, *J* = 2.0 Hz, 1H), 7.24 (dd, *J* = 8.4, 2.0 Hz, 1H), 6.85 (d, *J* = 8.4 Hz, 1H), 4.46 (dd, *J* = 8.4, 6.4 Hz, 1H), 3.03 (dd, *J* = 13.8, 6.4 Hz, 1H), 2.79 (dd, *J* = 13.8, 8.4 Hz, 1H), 2.67 (s, 3H), 1.90 (s, 3H). ^{19}F-NMR (564 MHz, methanol-d_4) δ -63.68; ^{13}C-NMR (150 MHz, methanol-d_4) δ 174.11, 173.29, 156.09, 135.16, 129.13, 128.53 (q, J = 5.0 Hz), 125.56 (q, J = 271.6 Hz), 117.89, 117.83 (q, *J* = 30.3 Hz), 56.51, 38.17, 26.40, 22.54. HRMS (ESI) $[M+H]^+$ calculated for $C_{13}H_{16}F_3N_2O_3^+$ 305.1108, found 305.1098.

3: ^{1}H-NMR (600 MHz, methanol-d_4) δ 7.65 (s, 2H), 4.51 (dd, J = 8.9, 6.0 Hz, 1H), 3.11 (dd, J = 13.9, 6.0 Hz, 1H), 2.87 (dd, J = 13.9, 8.9 Hz, 1H), 2.68 (s, 3H), 1.90 (s, 3H). ^{19}F-NMR (564 MHz, methanol-d_4) δ -63.06. ^{13}C-NMR (150 MHz, methanol-d_4) δ 173.69, 173.26, 153.52, 132.57 (q, J = 4.9 Hz, 2C) 130.88, 124.9 (q, J = 272.3 Hz, 2C) 122.13, 121.53 (q, J = 30.4 Hz, 2C), 56.08, 38.00, 26.39, 22.50. HRMS (ESI) $[M+H]^+$ calculated for $C_{14}H_{15}F_6N_2O_3^+$ 373.0981; found 373.0922.

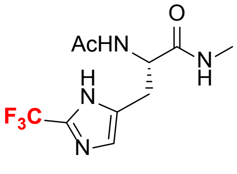

S1

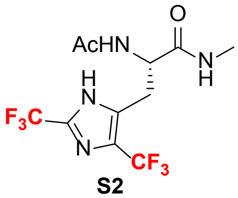

S2

*(S)-2-acetamido-N-methyl-3-(2-(trifluoromethyl)-1H-imidazol-5-yl)propanamide (**S1**) and (S)-2-acetamido-3-(2,4-bis(trifluoromethyl)-1H-imidazol-5-yl)-N-methylpropanamide (**S2**):* To a 1 mL vial containing *(S)*-2-acetamido-3-(*1H*-imidazol-5-yl)-*N*-methylpropanamide (50.00 mg, 0.238 mmol) and zinc trifluoromethansulfinate (237 mg, 0.713 mmol, 3 equiv) was added a solution of acetic acid (100 µL) and water (900 µL). This mixture was transferred into a N_2-filled glove box. To this solution was added a 70% solution of TBHP in water (163 µL, 1.189 mmol, 5 equiv) in a dropwise manner. The vial was sealed and the mixture was allowed to stand at 23 ºC for 16 h. The reaction mixture was lyophilized and reconstituted into DMSO. The resulting solution was purified by preparative HPLC purified using a Waters SunFire C18 preparative column (19 x 100 mm) with a 15 min run time (50 mL/min flow) and a gradient of 95:5 to 65:35 water:acetonitrile with 0.16% TFA. The fractions containing the desired products were combined and lyophilized under reduced pressure to afford 14.2 mg (21%) of mono-trifluoromethylated product **S1** and 1.8 mg (2%) of bis-trifluoromethylated product **S2**.

S1: ^{1}H-NMR (500 MHz, methanol-d_4) δ 7.02 (s, 1H), 4.57 (dd, J = 8.7, 5.7 Hz, 1H), 3.10 (dd, J = 15.0, 5.7 Hz, 1H), 2.92 (dd, J = 15.0, 8.7 Hz, 1H), 2.70 (s, 3H), 1.94 (s, 3H). ^{19}F-NMR (471 MHz, methanol-*d_4*) δ -64.90. ^{13}C-NMR (150 MHz, methanol-d_4) δ 172.27, 171.86, 135.74, 134.83, 119.93, 116.65 (*J* = 267 Hz), 53.17, 28.86, 24.92, 21.05. HRMS (ESI) $[M+H]^+$ calculated for $C_{10}H_{14}F_3N_4O_2^+$ 279.1069; found 279.1077.

S2: ^{1}H NMR (500 MHz, methanol-d_4) δ 4.64 (dd, *J* = 8.2, 6.2 Hz, 1H), 3.33 (dd, *J* = 14.8, 8.2 Hz, 1H), 3.09 (dd, *J* = 14.8, 8.2 Hz, 1H), 2.73 (s, 3H), 1.94 (s, 3H). ^{19}F-NMR (471 MHz, methanol-*d_4*) δ -61.66, -65.48. ^{13}C-NMR was not obtained due to an insufficient amount of **S2**. HRMS (ESI) $[M+H]^+$ calculated for $C_{11}H_{13}F_6N_4O_2^+$ 347.0943; found 347.0931.

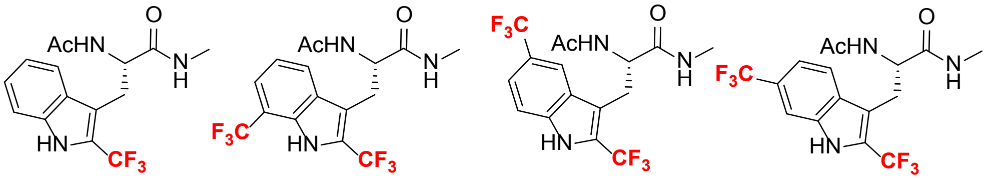

S3 S4 S5 S6

(S)-2-Acetamido-N-methyl-3-(2-(trifluoromethyl)-1H-indol-3-yl)propanamide *(**S3**), and isomers of (S)*-2-acetamido-3-(bis(trifluoromethyl)-1H-indol-3-yl)-N-methylpropanamide **S4-6***:* To a 1 mL

vial containing *(S)*-2-acetamido-3-(*1H*-indol-3-yl)-*N*-methylpropanamide (51.90 mg, 0.200 mmol) and zinc trifluoromethanesulfinate (199 mg, 0.6 mmol, 3 equiv) was added a solution of acetic acid (200 µL) and water (1800 µL). This mixture was transferred into a N_2-filled glove box. To this solution was added a 70% solution of TBHP in water (137 µL, 1.00 mmol, 5 equiv) in a dropwise manner. The vial was sealed and the mixture was allowed to stand at 23 °C for 16 h. The reaction mixture was lyophilized and reconstituted into DMSO. The resulting solution was purified by preparative HPLC purified using a Waters SunFire C18 preparative column (19 x 100 mm) with a 15 min run time (50 mL/min flow) and a gradient of 95:5 to 65:35 water:acetonitrile with 0.16% TFA. The fractions containing the desired products were combined and lyophilized under reduced pressure to afford 15.0 mg (23%) of mono-trifluoromethylated product **S3** and a total of 10.8 mg (10%) of bis-trifluoromethylated isomers **S4** and a mixture of **S5** and **S6** (chemical shifts are reported separately for each isomer).

S3: ^{1}H-NMR (500 MHz, methanol-*d*$_4$) δ ^{1}H NMR (600 MHz, methanol-d$_4$) δ 7.71 (d, J = 8.1 Hz, 1H), 7.40 (d, *J* = 8.3 Hz, 1H), 7.25 (t, *J* = 7.8 Hz, 1H), 7.12 (t, *J* = 7.7 Hz, 1H), 4.57 (t, *J* = 7.7 Hz , 1H), 3.40 (dd *J* = 14.5, 7.4 Hz, 1H), 3.19 (dd, *J* = 14.5, 7.4, Hz, 1H), 2.56 (s, 3H), 1.87 (s, 3H). ^{13}C-NMR (150 MHz, methanol-d$_4$) δ 173.72, 172.82, 137.37, 128.60, 125.42, 123.7 (q, *J* = 269 Hz),123.6 (q, *J* = 36.4 Hz) 121.16, 121.13, 113.33, 113.04, 55.87, 28.02, 26.33, 22.51.^{19}F-NMR (471 MHz, methanol-*d*$_4$) δ -59.16. HRMS (ESI) [M+H]$^+$ calculated for $C_{15}H_{17}F_3N_3O_2^+$ 328.1273; found 328.1272.

S4: ^{1}H-NMR (500 MHz, methanol-*d*$_4$) δ 8.01 (d, *J* = 8.0 Hz, 1H), 7.59 (d, *J* = 7.8 Hz, 1H), 7.27 (t, *J* = 7.8 Hz, 1H), 4.58 (t, *J* = 7.4 Hz, 1H), 3.44 (dd, *J* = 14.3, 7.4 Hz, 1H), 3.23 (dd, *J* = 14.3 Hz, 7.4 Hz, 1H), 2.57 (s, 3H), 1.87 (s, 3H). ^{13}C-NMR (150 MHz, methanol-d$_4$) δ 172.3, 171.4, 130.9, 128.8, 125.2, 124.5, 124.0, 121.7, 119.4, 114.0, 113.1, 55.87, 28.02, 26.33, 22.51.^{19}F-NMR (471 MHz, methanol-*d*$_4$) δ -59.08, -63.05. HRMS (ESI) [M+H]$^+$ calculated for $C_{16}H_{16}F_6N_3O_2^+$ 396.1147; found 396.1150.

S5+S6 for **S5:** ^{1}H-NMR (500 MHz, methanol-*d*$_4$) δ 8.10 (s, 1H), 7.55 (d, *J* =8.6 Hz, 1H), 7.52 (d, *J* = 8.6 Hz, 1H), 4.59 (m, 1H), 3.42 (dd, *J* = 14.0, 6.7 Hz 1H), 3.22 (dd, *J* = 14.0, 6.7 Hz, 1H), 2.57 (s, 3H), 1.87 (s, 3H). for **S6** ^{1}H-NMR (500 MHz, methanol-*d*$_4$) δ 7.90 (d, *J* = 8.6 Hz, 1H), 7.72 (s, 1H), 7.38 (d, *J* = 8.6 Hz, 1H), 4.59 (m, 1H), 3.42 (m, 1H), 3.22 (m, 1H), 2.57 (s, 3H), 1.87 (s, 3H). ^{13}C-NMR was not available due to limited quantities of the mixture. ^{19}F-NMR (471 MHz, methanol-*d*$_4$) δ -59.64, -59.76, -61.88, -62.67. HRMS (ESI) [M+H]$^+$ calculated for $C_{16}H_{16}F_6N_3O_2^+$ 396.1147; found 396.1146.

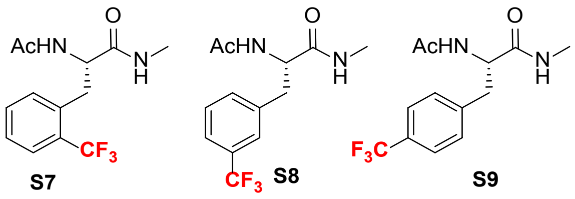

*(S)-2-Acetamido-N-methyl-3-(2-(trifluoromethyl)-1H-indol-3-yl)propanamide (**S7**) and isomers of (S)-2-acetamido-3-(2,7-bis(trifluoromethyl)-1H-indol-3-yl)-N-methylpropanamide **S8** and **S9**:* To a 1 mL vial containing *(S)*-2-acetamido-*N*-methyl-3-phenylpropanamide (50.0 mg, 0.227 mmol) and zinc trifluoromethansulfinate (226 mg, 0.681 mmol, 3 equiv) was added a solution of acetic acid (100 µL) and water (900 µL). This mixture was transferred into a N_2-filled glove box. To this solution was added a 70% solution of TBHP in water (155 µL, 1.14 mmol, 5 equiv) in a dropwise manner. The vial was sealed and the mixture was allowed to stand at 23 °C for 16 h. The reaction mixture was lyophilized and reconstituted into DMSO. The resulting solution was purified by preparative HPLC purified using a Waters SunFire C18 preparative column (19 x 100 mm) with a 15 min run time (50 mL/min flow) and a gradient of 95:5 to 65:35 water:acetonitrile with 0.16% TFA. The fractions containing the desired products were combined and lyophilized under reduced pressure to afford 0.5 mg (0.8%) of *ortho*-trifluoromethylated product **S7** and 1.8 mg (2.4%) of an inseparable mixture of *meta*- **S8** and *para*- **S9** trifluormethylated isomers.

S7: ^{1}H-NMR (500 MHz, methanol-d_4) δ 8.27 (b, 1H), 7.88 (b, 1H), 7.66 (d, *J* = 7.8 Hz, 1H), 7.52 (t, *J* = 7.8 Hz, 1H), 7.43 (m, 2H), 4.69 - 4.45 (m, 1H), 3.31 (dd, *J* = 14.6, 8.5 Hz, 1H), 3.14 - 2.91 (dd, *J* = 14.6, 8.5 Hz, 1H), 2.73 (d, *J* = 4.6 Hz, 3H), 1.90 (s, 3H). ^{13}C-NMR was not obtained due to an insufficient amount of isolated **S7.** ^{19}F-NMR (471 MHz, methanol- d_4) δ -64.1. HRMS (ESI) $[M+H]^+$ calculated for $C_{13}H_{16}F_3N_2O_2^+$ 289.1164; found 289.1169.

Mixture of **S8** and **S9**: **major** ^{1}H-NMR (500 MHz, methanol-d_4) δ 8.23, (b, *J* = 4.7 Hz , 1H), 7.95, (b, 1H), 7.55 - 7.44 (m, 4H), 4.54 (m, 1H), 3.18 (dd, *J* = 14.0, 6.0 Hz, 1H), 2.93 (dd, *J* = 14.0, 6.0 Hz, 1H), 2.67 (d, *J* = 7.6 Hz, 3H), 1.89 (s, 3H). **minor** ^{1}H-NMR (500 MHz, methanol-d_4) δ 8.23, (d, *J* = 4.7 Hz , 1H), 7.95, (b, 1H), 7.57 (d, *J* = 8.1 Hz, 2H), 7.42 (d, *J* = 8.1 Hz, 1H), 4.54 (m, 1H), 3.18 (dd, *J* = 13.7, 6.0 Hz, 1H), 2.93 (dd, *J* = 13.7, 9.1 Hz, 1H), 2.67 (d, *J* = 4.7 Hz, 3H), 1.89 (s, 3H).
major+minor: ^{13}C-NMR was not obtained due to an insufficient amount of isolated **S8** and **S9.** ^{19}F-NMR (471 MHz, methanol-d_4) δ -63.96, -64.08. HRMS (ESI) $[M+H]^+$ calculated for $C_{13}H_{16}F_3N_2O_2^+$ 289.1164; found 289.1175.

b. Synthesis of trifluoromethylated peptides. **Please note:** reaction yields reported in Table 4 of the manuscript were determined by ^{19}F NMR. In the procedures given below, isolated yields are reported after preparative HPLC. In addition, in some cases reaction conditions in the preparative examples listed below differ slightly from those reported in Table 4.

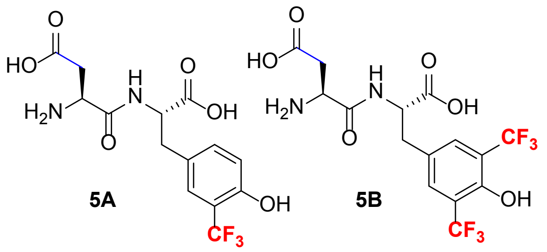

5A **5B**

*CF_3[Y]-Asp-Tyr (**5A**) and* ***(****$(CF_3)_2$[Y]-Asp-Tyr (**5B**)**):*** To a 4 mL vial containing Asp-Tyr (**5**) (30.5 mg, 0.103 mmol) and zinc trifluoromethanesulfinate (102 mg, 0.309 mmol, 3 equiv) was added 520 µL solution of 10% acetic acid in water (final concentration = 0.2 M). This mixture was transferred into a N_2-filled glove box. To this solution was added a 70% solution of TBHP in water (70 µL, 0.510 mmol, 5 equiv) in a dropwise manner. The vial was sealed and the mixture was allowed to stand at 37 ºC for 16 h. The reaction mixture was lyophilized and reconstituted into 1 mL DMSO. The resulting solution was purified using a SunFire Prep C18 OBD column (30 x 150 mm) with 20 min run time (30 mL/min flow) and a gradient of 10-100% acetonitrile/water with 0.05% TFA. The fractions containing the desired products were combined and lyophilized under reduced pressure to afford 11 mg (29%) of mono-trifluoromethylated product **5A** and 3.3 mg (7.3%) of bis-trifluoromethylated product **5B**.

5A: ^{1}H-NMR (500 MHz, methanol-d_4) δ 7.40 (s, 1H), 7.32 (d, J = 8.4 Hz, 1H), 6.90 (d, J = 8.4 Hz, 1H), 4.66 (dd, J = 9.7, 4.9 Hz, 1H), 4.13 (dd, J = 9.7, 3.7 Hz, 1H), 3.26 – 3.15 (m, 1H), 3.07 – 2.92 (m, 2H), 2.77 (dd, J = 18.0, 9.7 Hz, 1H). ^{13}C-NMR (125 MHz, methanol-d_4) δ 172.13, 171.60, 168.06, 152.03, 131.04, 131.00, 128.97, 124.46, 122.30, 120.61 (d, J = 30.4 Hz), 53.93, 49.62, 35.53, 34.79. ^{19}F-NMR (471 MHz, methanol-d_4) δ -63.75. HRMS (ESI) $[M+H]^+$ calculated for $C_{14}H_{16}F_3N_2O_6^+$: 365.0882; found 365.0954.

5B: ^{1}H-NMR (500 MHz, methanol-d_4) δ 7.70 (s, 2H), 4.71 (dd, J = 8.8, 3.7 Hz, 1H), 4.14 (dd, J = 8.8, 3.7 Hz, 1H), 3.38 – 3.22 (m, 1H), 3.15 – 2.95 (m, 2H), 2.80 (m, 1H). ^{13}C-NMR was not available due to an insufficient quantity of isolated trifluoromethylated product. ^{19}F-NMR (471 MHz, methanol-d_4) δ -63.14. HRMS (ESI) $[M+H]^+$ calculated for $C_{15}H_{15}F_6N_2O_6^+$: 433.0756; found 433.0826.

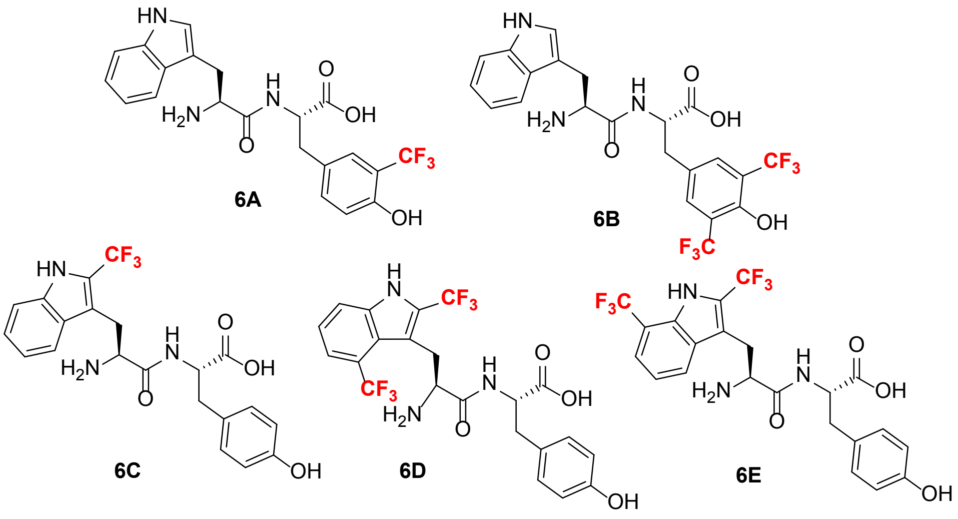

6A 6B

6C 6D 6E

*CF_3[Y]-Trp-Tyr (**6A-B**) and CF_3[W]-Trp-Tyr (**6C-E**):* To an 8 mL vial containing Trp-Tyr (**6**) (100 mg, 0.272 mmol) and zinc trifluoromethanesulfinate (296 mg, 0.816 mmol, 3 equiv) was added 2700 µL of 10% acetic acid in water (final concentration 0.1 M). This mixture was transferred into a N_2-filled glove box. To this solution was added a 70% solution of TBHP in water (186 µL, 1.360 mmol, 5 equiv) in a dropwise manner. The vial was sealed and the mixture was allowed to stand at 37 ºC for 16 h. The reaction mixture was lyophilized and reconstituted into 3 mL DMSO. The resulting solution was purified using a SunFire Prep C18 OBD column (30 x 150 mm) with 20 min run time (30 mL/min flow) and a gradient of 0-60% acetonitrile/water with 0.05% TFA. The fractions containing the desired products were combined and lyophilized under reduced pressure to afford 40 mg (34%) of mono-trifluoromethylated product **6C** and 5.1 mg (4%) bis-trifluoromethylated product **6D** and 9.5 mg (6%) of bis-trifluoromethylated product **6E** (in 92% purity). Compound **6A** was observed by ^{19}F-NMR (see the manuscript for yields (10%, -61.5ppm in d_6-DMSO), but it was inseparable from the mixture for full characterization (no **6B** was formed).

6C ^{1}H-NMR (500 MHz, methanol-*d_4*) δ 7.75 (d, *J* = 8.2 Hz, 1H), 7.47 (d, *J* = 8.2 Hz, 1H), 7.33 (dd, *J* = 7.8, 6.9 Hz, 1H), 7.18 (dd, *J* = 7.8, 6.9 Hz 1H), 7.07 – 6.93 (m, 2H), 6.68 (d, *J* = 8.3 Hz, 2H), 4.50 (t, *J* = 6.4 Hz, 1H), 4.4 (t, J = 2H), 3.45 (m, 1H); 3.4 (m, 1H); 3.1 (m, 1H) and 3.29 (m, 1H). ^{13}C-NMR (125 MHz, methanol-*d_4*) δ 172.79, 168.68, 156.03, 136.94, 129.83, 127.43, 126.88, 124.43, 121.52, 118.90, 117.68, 114.89, 111.22, 106.38, 54.39, 53.26, 36.05, 27.40. ^{19}F-NMR (471 MHz, methanol-*d_4*) δ -59.00. HRMS (ESI) $[M+H]^+$ calculated for $C_{21}H_{21}F_3N_3O_4^+$ 436.1479; found 436.1460.

6D ^{1}H-NMR (500 MHz, methanol-*d_4*) δ 7.69 (d, *J* = 8.2 Hz, 1H), 7.48 (d, *J* = 8.2 Hz, 1H), 7.44 (s, 1H), 7.26 (t, *J* = 8.4 Hz, 1H), 7.03 (d, *J* = 8.4 Hz, 2H), 6.70 (d, *J* = 8.4 Hz, 2H), 4.63 (dd, *J* = 8.4,

5.6 Hz, 1H), 4.16 (m, 1H), 3.50 (m, 1H), 3.35 (m, 1H), 3.17 (m, 1H) 2.88 (m, 1H). ^{13}C-NMR (125 MHz, methanol-d_4) δ 172.48, 168.60, 161.44, 156.05, 138.28, 129.89, 129.74, 127.27, 120.24, 120.05, 117.31, 117.26, 116.00, 114.89, 106.11, 54.46, 53.21, 29.27, 22.81. ^{19}F-NMR (471 MHz, ,methanol-d_4) δ -58.57. HRMS (ESI) $[M+H]^+$ calculated for $C_{22}H_{20}F_6N_3O_4^+$ 504.1353; found 504.1324.

6E ^{1}H-NMR (500 MHz, methanol-d_4) δ 7.96 (d, *J* = 8.0 Hz, 1H), 7.47 (d, *J* = 8.0 Hz, 1H), 7.41 – 7.36 (s, 1H), 7.22 (dd, *J* = 8.0, 7.4 Hz, 1H), 7.08 (dd, *J* = 8.0, Hz, 2H), 6.72 (d, *J* = 8.0 Hz, 2H), 4.71 – 4.61 (m, 1H), 4.14 (m, 1H), 3.47 (dt, *J* = 9.7, 4.9 Hz, 1H), 3.31 – 3.13 (m, 2H), 2.88(m, 1H). ^{19}F-NMR (471 MHz, methanol-d_4) δ -58.90. HRMS (ESI) $[M+H]^+$ calculated for $C_{22}H_{20}F_6N_3O_4^+$ 504.1353; found 504.1213.

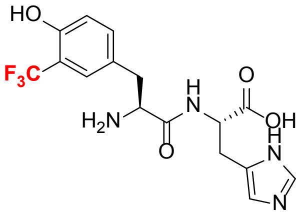

7A

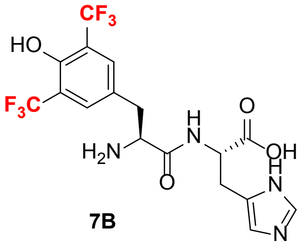

7B

*CF_3[Y]-Tyr-His (**7A**) and $(CF_3)_2$[Y]-Tyr-His (**7B**)* To an 8 mL vial containing Tyr-His (**7**) (100 mg, 0.314 mmol) and zinc trifluoromethanesulfinate (312 mg, 0.942 mmol, 3 equiv) was added 3100 µL of 10% acetic acid in water (final concentration 0.1 M). This mixture was transferred into a N_2-filled glove box. To this solution was added a 70% solution of TBHP in water (215 µL, 1.523 mmol, 5 equiv) in a dropwise manner. The vial was sealed and the mixture was allowed to stand at 37 ºC for 16 h. The reaction mixture was lyophilized and reconstituted into 3 mL DMSO. The resulting solution was purified using a SunFire Prep C18 OBD column (30 x 150 mm) with 20 min run time (30 mL/min flow) and a gradient of 0-50% acetonitrile/water with 0.05% TFA. The fractions containing the desired products were combined and lyophilized under reduced pressure to afford 30 mg (25%) of mono-trifluoromethylated product **7A** and 9.4 mg (7%) of bis-trifluoromethylated product **7B**.

7A ^{1}H-NMR (500 MHz, methanol-d_4) δ 8.80 (s, 1H), 7.46 (s, 1H), 7.37 (s, 1H), 7.35 (s, 1H), 6.94 (d, *J* = 8.4 Hz, 1H), 4.82 (dd, *J* = 7.6, 5.9 Hz, 2H), 4.18 (dd, *J* = 7.6, 5.9 Hz, 1H), 3.35 (dd, *J* = 14.8, 9.7 Hz, 2H), 3.28 – 3.18 (m, 3H), 3.05 (dd, *J* = 14.8, 9.7 Hz, 1H). ^{13}C-NMR (125 MHz, methanol-d_4) δ 171.08, 168.40, 155.45, 134.14, 133.61, 129.45, 127.57, 125.0, 124.19, 121.0, 117.07, 116.98, 54.09, 51.68, 35.96, 26.45. ^{19}F-NMR (471 MHz, methanol-d_4) δ -63.81. HRMS (ESI) $[M+H]^+$ calculated for $C_{16}H_{18}F_3N_4O_4^+$ 387.1202; found 387.1275.

7B ^{1}H-NMR (500 MHz, methanol-d_4) δ 8.81 (s, 1H), 7.75 (s, 2H), 7.32 (s, 1H), 4.88 – 4.81 (m, 1H), 4.27 – 4.21 (m, 1H), 3.37 (dd, *J* = 15.5, 5.1 Hz, 1H), 3.35 – 3.13 (m, 4H). ^{13}C-NMR (125 MHz, methanol-d_4) δ 170.93, 167.96, 152.94, 133.66, 131.65, 129.45, 125.63, 124.38, 122.21,

120.85 (q, J = 30.6 Hz), 117.03, 53.91, 51.45, 35.83, 26.54. ^{19}F-NMR (471 MHz, methanol-d_4) δ -63.22. HRMS (ESI) $[M+H]^+$ calculated for $C_{17}H_{17}F_6N_4O_4^+$ 455.1149; found 455.1151.

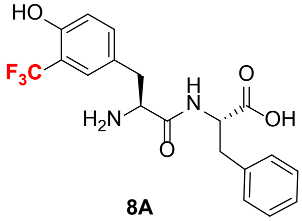

8A

*CF_3[Y]-Tyr-Phe (**8A**):* To an 8 mL vial containing Tyr-Phe (**8**) (100 mg, 0.305 mmol) and zinc trifluoromethanesulfinate (303 mg, 0.914 mmol, 3 equiv) was added 3050 µL of 10% acetic acid in water (final concentration 0.1 M). This mixture was transferred into a N_2-filled glove box. To this solution was added a 70% solution of TBHP in water (210 µL, 1.523 mmol, 5 equiv) in a dropwise manner. The vial was sealed and the mixture was allowed to stand at 37 ºC for 16 h. The reaction mixture was lyophilized and reconstituted into 3 mL DMSO. The resulting solution was purified using a SunFire Prep C18 OBD column (30 x 150 mm) with a 20 min run time (30 mL/min flow) and a gradient of 10-100% acetonitrile/water with 0.05% TFA. The fractions containing the desired products were combined and lyophilized under reduced pressure to afford 26.4 mg (21%) of mono-trifluoromethylated product **8A.**

^{1}H-NMR (500 MHz, methanol-d_4) δ 7.49 (s, 1H), 7.29 (m, 5H), 6.94 (d, J = 8.4 Hz, 1H), 4.75 (dd, J = 8.8, 5.1 Hz, 1H), 4.03 (dd, J = 8.8, 5.1 Hz, 1H), 3.26 (dd, J = 14.4, 8.8 Hz, 2H), 3.02 (dd, J = 14.4, 8.8 Hz, 2H).^{13}C-NMR (125 MHz, methanol-d_4) δ 172.53, 168.0, 156.90, 155.49, 138.30, 136.77, 134.13, 130.24, 128.82, 128.17, 127.57, 126.54, 124.30, 117.03, 115.48, 54.41, 53.93, 36.86, 36.07. ^{19}F-NMR (471 MHz, methanol-d_4) δ -63.86. HRMS (ESI) $[M+H]^+$ calculated for $C_{19}H_{20}F_3N_2O_4^+$ 397.1370; found 397.1372.

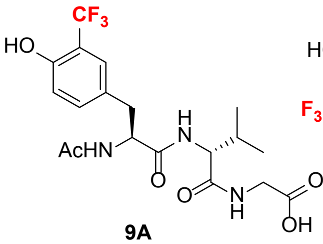

9A

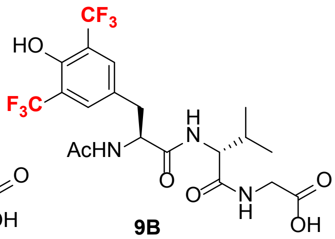

9B

*CF_3[Y]-Ac-Tyr-Val-Gly (**9A**) and $(CF_3)_2$[Y]-Ac-Tyr-Val-Gly (**9B**):* In a N_2-filled glove box, Ac-Tyr-Val-Gly (**9**) (30 mg, 79 µmol, 1 equiv), [Ir(dFCF$_3$ppy)$_2$(dtbpy)](PF$_6$) (cat. **4**, 12 µmol, 15 mol%) and $NaSO_2CF_3$ (247mg, 1.580 mmol, 20 equiv) were added to a 4 mL vial with a stir bar. To the reaction vial were added 395 µL CH_3CN and 395 µL 10% AcOH in H_2O. The vial was sealed and placed in the MSD photoreactor[1] and irradiated with the following settings: fan rate 4700 rpm; blueLED 100% (13.2 W); stir rate 1000 rpm. The reaction was irradiated for 16 h. The resultant mixture was taken from the MSD reactor and diluted with 2.8 mL of 1:1 H_2O:CH_3CN mixture (total volume 3.2 mL). A portion of the reaction mixture (2.4 mL, 75% of the content) was then purified using a Gilson HPLC (Waters SunFire Prep C18 19 x 100mm, flow 30 mL/min) with 10-90% CH_3CN/H_2O (0.05% TFA) affording 13.7 mg (50.6%) of **9A** as a white solid and 2.9 mg (9.3%) of **9B** as a white solid after overnight lyophilization (yield was adjusted based on the percentage of purified crude).

*CF_3[Y]-Ac-Tyr-Val-Gly (***9A***)* ^{1}H-NMR (500 MHz, methanol-*d*$_4$) δ 7.39 (d, *J* = 2.2 Hz, 1H), 7.28 (dd, *J* = 2.2, 8.4 Hz, 1H), 6.86 (d, *J* = 8.4 Hz, 1H), 4.70 – 4.62 (m, 1H), 4.27 (dd, *J* = 5.5, 7.7 Hz, 1H), 4.05 – 3.80 (m, 2H), 3.08 (dd, *J* = 5.5, 14.0 Hz, 1H), 2.84 (dd, *J* = 9.1, 14.0 Hz, 1H), 2.2 (m, 1H), 2.09 (s, 3H), 0.98 (d, *J* = 6.8, 12.9 Hz, 6H). ^{13}C-NMR (125 MHz, methanol-*d*$_4$) δ 172.29, 172.11, 171.84, 171.21, 154.46, 133.74, 127.53, 127.13, 124.05 (q, J = 270Hz), 116.41, 116.18, 58.48, 54.72, 40.35, 36.34, 30.78, 20.95, 18.27, 17.13.^{19}F-NMR (471 MHz, methanol-*d*$_4$) δ -63.54. HRMS (ESI) [M+H]+ calculated for $C_{19}H_{25}F_3N_3O_6^+$ 448.1617; found 448.1690.

*$(CF_3)_2$[Y]-Ac-Tyr-Val-Gly (***9B***)* ^{1}H-NMR (500 MHz, methanol-*d*$_4$) δ 7.70 (s, 2H), 4.70 (dd, *J* = 5.1, 9.5 Hz, 1H), 4.29 – 4.23 (m, 1H), 4.05 – 3.78 (m, 2H), 3.17 (dd, *J* = 5.1, 14.1 Hz, 1H), 2.90 (dd, *J* = 9.5, 14.1 Hz, 1H), 2.2 (m, 1H), 2.11 (s, 3H), 0.99 (dd, *J* = 6.8, 12.1 Hz, 6H). ^{13}C-NMR (125 MHz, methanol-*d*$_4$) δ 172.31, 171.80, 171.72, 171.17, 151.93, 131.12 (q, *J* = 5.0 Hz), 129.36, 123.45 (q, *J* = 270 Hz), 120.36, 58.53 54.29 , 40.28, 36.16, 30.77, 20.86, 18.27, 17.05. ^{19}F-NMR (471 MHz, methanol-*d*$_4$) δ -63.00. HRMS (ESI) $[M+H]^+$ calculated for $C_{20}H_{24}F_6N_3O_6^+$ 516.1491; found 516.1568.

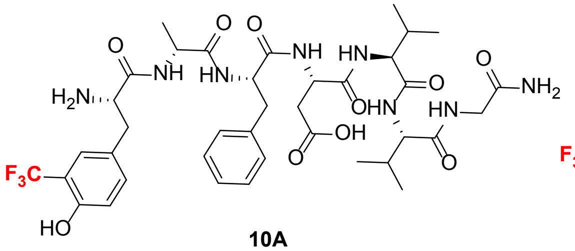

10A

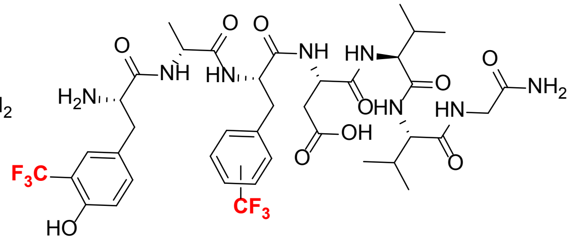

ortho-, meta-, para-S11, S-12, S-13

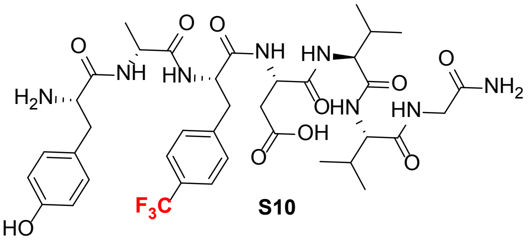

S10

*CF_3[Y]-Deltorphin I (***10A**), *CF_3[F]-Deltorphin I* (**S10**) and **(***$(CF_3)_2$[Y,F]-Deltorphin I (***S11 -13**): In a N_2-filled glove box, deltorphin I (**10**) (70 mg, 91 μmol, 1 equiv), [Ir(dFCF$_3$ppy)$_2$(dtbpy)](PF$_6$) (**4**, 14 μmol, 15 mol%) and $NaSO_2CF_3$ (285 mg, 1800 μmol, 20 equiv) were added to a 4 mL vial with a stir bar. To the reaction vial were added 1520 μL CH_3CN and 1520 μL aqueous 10% AcOH. The vial was sealed and placed in the MSD photoreactor[1] and irradiated with the following settings: fan rate 4700 rpm; blue LED 100% (13.2 W); stir rate 1000 rpm. The reaction was irradiated for 16 h. The reaction mixture was diluted with EtOAc (3 mL) and 1 mL of H_2O was added. The solution yields were determined by adding an internal standard α,α,α-trifluorotoluene (Aldrich) in d_6-DMSO. Ethyl acetate was washed with H_2O three times and the combined aqueous layer was lyophilized. The reaction mixture was re-constituted with 2 mL of DMSO. The resulting solution was purified using a Waters Peptide CSH prep column (30 x 150 mm) with a 25 min run time (50 mL/min flow) and a gradient of 77:23 to 62:38 water:acetonitrile with 0.16% TFA. The fractions containing the desired products were combined and lyophilized under reduced pressure to afford 2.6 mg (3.4%) of CF_3-Deltorphin I (1:1.6 = **10A**:**S10**) and 1.9 mg (2.3%) of $(CF_3)_2$-Deltorphin I as white solids (2.5:3:1 = **S11**:**S12**:**S13**). The isolated yields were low due to the attempt to maximize purity of final compounds for characterization. NMR and HRMS of **10A, S10-S13** were obtained and the data are included below.

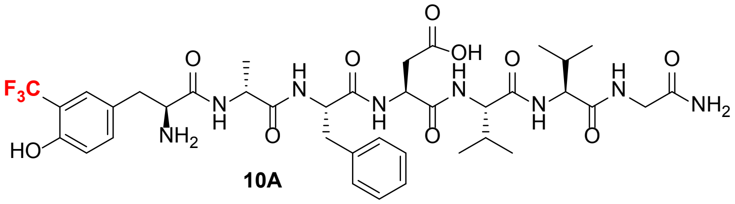

10A

10A: ^{1}H-NMR (500 MHz, methanol-d_4) δ 7.42 (d, J = 8.4 Hz, 1H), 7.38 – 7.17 (m, 6H), 6.96 (d, J = 8.4 Hz, 1H), 4.83 (m, 1H) 4.60 (dd, J = 4.3, 10.7 Hz, 1H), 4.23 – 4.13 (m, 2H), 4.12 – 4.04 (m,

1H), 4.01 (t, *J* = 7.4 Hz, 1H), 3.96 (d, *J* = 16.6Hz, 1H) ,3.72 (d, *J* = 16.6 Hz, 1H), 3.34 (m, 1H), 3.20 – 3.00 (m, 3H), 2.90 – 2.77 (m, 2H), 2.19 (m, 2H), 1.06 – 0.83 (m, 15H). ^{19}F-NMR (471 MHz, methanol-d_4) δ -63.86. ^{13}C-NMR is unavailable due to an insufficient quantity of isolated trifluoromethylated product.

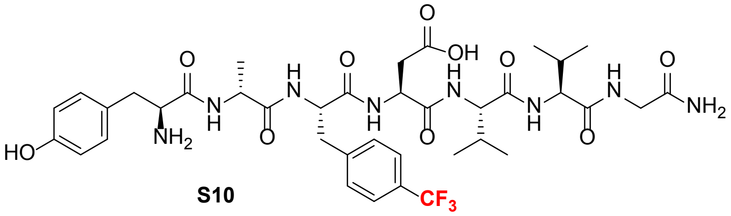

S10

S10: ^{1}H-NMR (600 MHz, methanol-d_4) δ 7.60 (d, *J* = 8.0 Hz, 2H), 7.46 (d, *J* = 8.0 Hz, 2H), 7.12 – 7.07 (m, 2H), 6.81 – 6.75 (m, 2H), 4.83 (m, 1H), 4.67 – 4.63 (m, 1H), 4.18 (d, *J* = 7.7 Hz, 1H), 4.13 (m, 1H), 4.06 (d, *J* = 7.7 Hz, 1H), 3.99 (m, 1H), 3.92 (d, *J* = 16.4Hz), 1H), 3.78 (d, *J* = 16.4Hz, 1H), 3.46 – 3.39 (m, 1H), 3.10 (m, 1H), 3.04 – 2.87 (m, 3H), 2.81 (dd, *J* = 6.4, 16.9 Hz, 1H), 2.26 – 2.11 (m, 2H), 1.05 – 0.91 (m, 15H). ^{19}F-NMR (471 MHz, methanol-d_4) δ -63.97. ^{13}C-NMR is unavailable due to an insufficient quantity of isolated trifluoromethylated product.

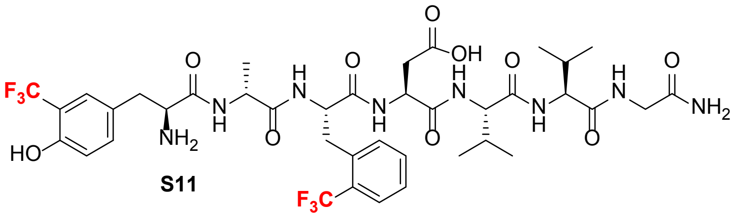

S11

S11: ^{1}H-NMR (500 MHz, methanol-d_4) δ 7.69 (d, *J* = 7.9 Hz, 1H), 7.56 (t, *J* = 7.5 Hz, 1H), 7.46 – 7.41 (m, 3H), 7.33 (dd, *J* = 2.2, 8.5 Hz, 1H), 6.96 (d, *J* = 8.4 Hz, 1H), 4.80 (m, 1H), 4.74 (m, 1H), 4.18 (m, 2H), 4.09 – 3.99 (m, 2H), 3.93, (d, *J* = 16.6Hz, 1H), 3.76 (d, *J* = 16.6 Hz, 1H), 3.65 (d, *J* = 4.2 Hz, 1H), 3.20 – 2.96 (m, 4H), 2.83 (dd, *J* = 6.4, 16.8 Hz, 1H), 2.19 (m, 2H), 1.07 – 0.86 (m, 15H). ^{19}F-NMR (471 MHz, methanol-d_4) δ -60.54, -63.84. ^{13}C-NMR is unavailable due to an insufficient quantity of isolated trifluoromethylated product.

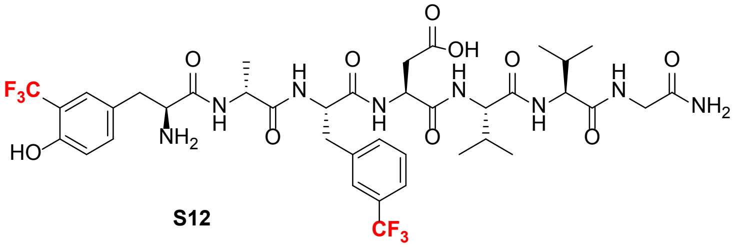

S12

S12: ^{1}H-NMR (500 MHz, methanol-d_4) δ 7.59 (s, 1H), 7.55 – 7.46 (m, 3H), 7.42 (d, *J* = 2.2 Hz, 1H), 7.32 (dd, *J* = 2.2, 8.5 Hz, 1H), 6.96 (d, *J* = 8.4 Hz, 1H), 4.75-4.64 (m, 2H), 4.19 – 4.12 (m, 2H), 4.06 (dd, *J* = 2.2, 7.7 Hz, 1H), 4.01 (t, *J* = 7.4 Hz, 1H), 3.96 (d, *J* = 16.6Hz,1H), 3.72 (d, *J* = 16.6 Hz, 1H), 3.43 (dd, *J* = 4.1, 14.4 Hz, 1H), 3.19 – 2.89 (m, 4H), 2.81 (dd, *J* = 6.3, 16.9 Hz, 1H), 2.18 (m, 2H), 1.05 – 0.91 (m, 15H).^{19}F-NMR (471 MHz, methanol-d_4) δ -63.86, -64.04. ^{13}C-NMR is unavailable due to an insufficient quantity of isolated trifluoromethylated product.

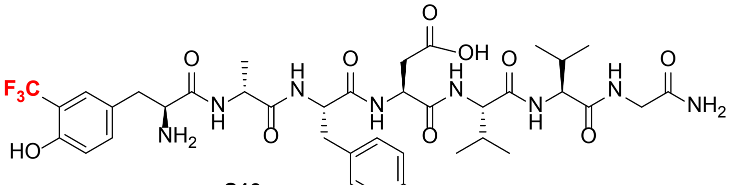

S13

S13: ^{1}H-NMR (500 MHz, methanol-d_4) δ 7.60 (d, *J* = 8.1 Hz, 2H), 7.46 (d, *J* = 8.1 Hz, 2H), 7.41 (s, 1H), 7.31 (d, *J* = 8.2 Hz, 1H), 6.95 (d, *J* = 8.4 Hz, 1H), 4.70 – 4.58 (m, 2H), 4.25 - 4.19 (m, 2H), 4.12 – 3.95 (m, 2H), 3.92 (d, *J* = 16.9 Hz, 1H), 3.76 (d, *J* = 16.9 Hz, 1H), 3.42 (dd, *J* = 14.6, 4.4 Hz, 1H), 3.16 – 2.92 (m, 3H), 2.80 (m, 2H), 2.30 – 2.07 (m, 2H), 1.08 – 0.88 (m, 15H). ^{19}F-NMR (471 MHz, methanol-d_4) δ -63.85, -63.97. ^{13}C-NMR is unavailable due to an insufficient quantity of isolated trifluoromethylated product.

HRMS LC/MS/MS Generated on Thermo Orbitrap System

1. CF_3[Y]-deltorphin I (~20% of **S10**) **10A**

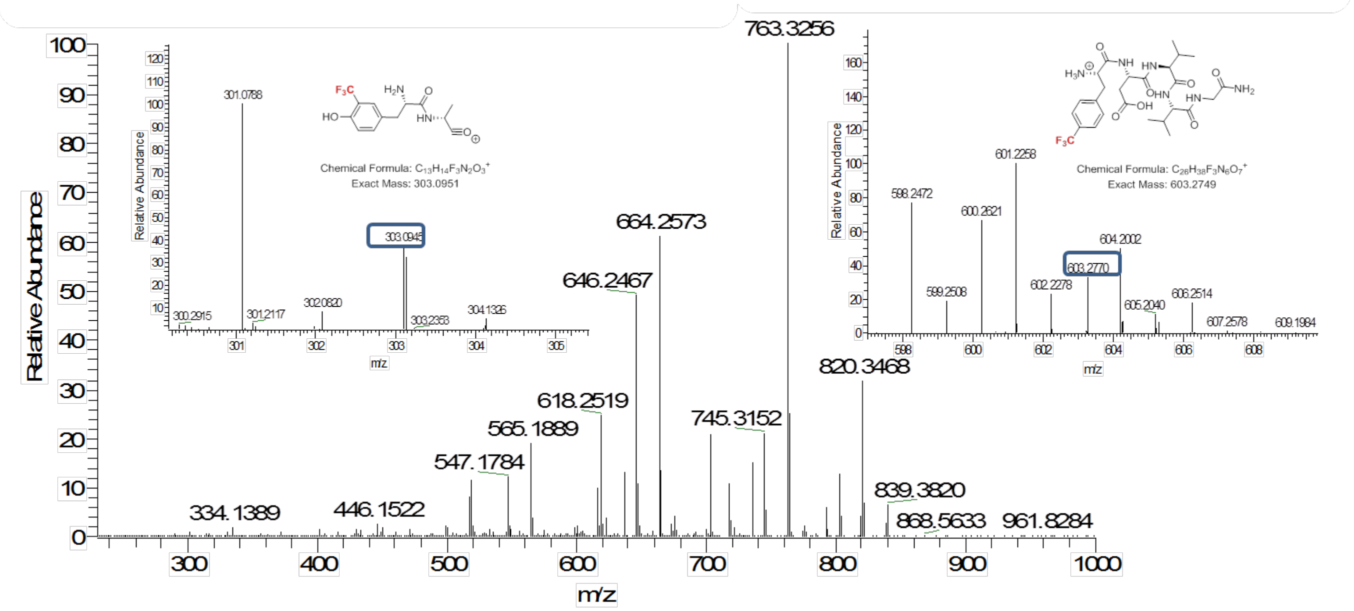

2. *p*-CF_3[F] deltorphin I **S10**

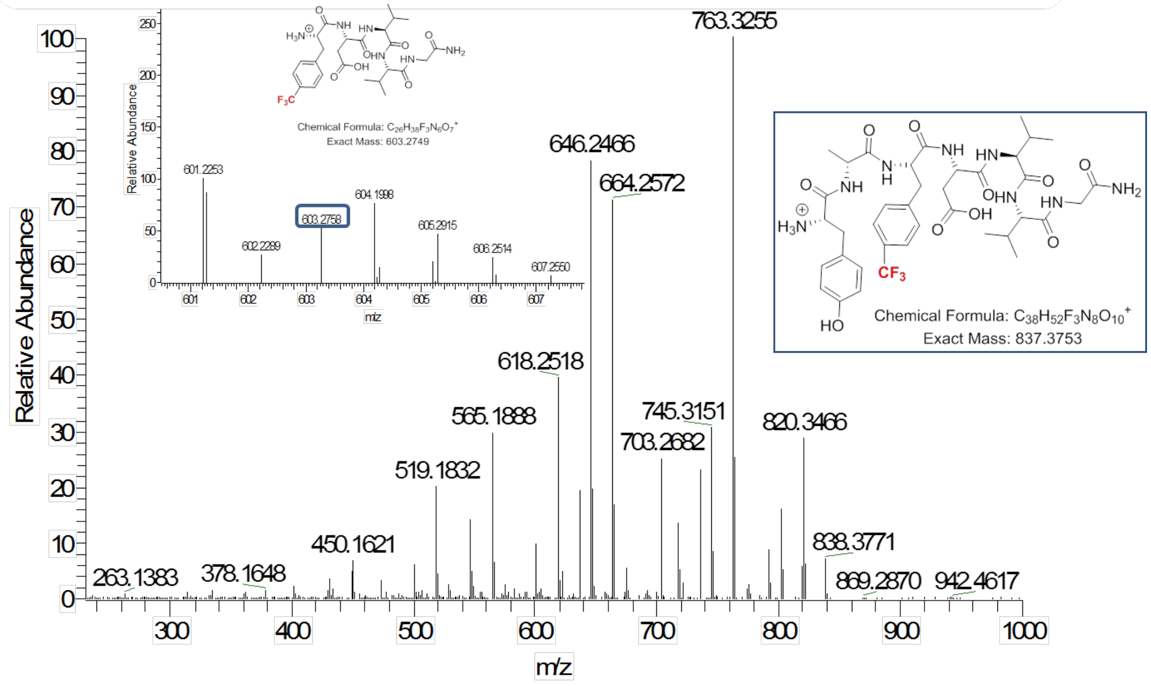

3. *o*-CF_3[Y]-*o*-CF_3[F] deltorphin I **S11**

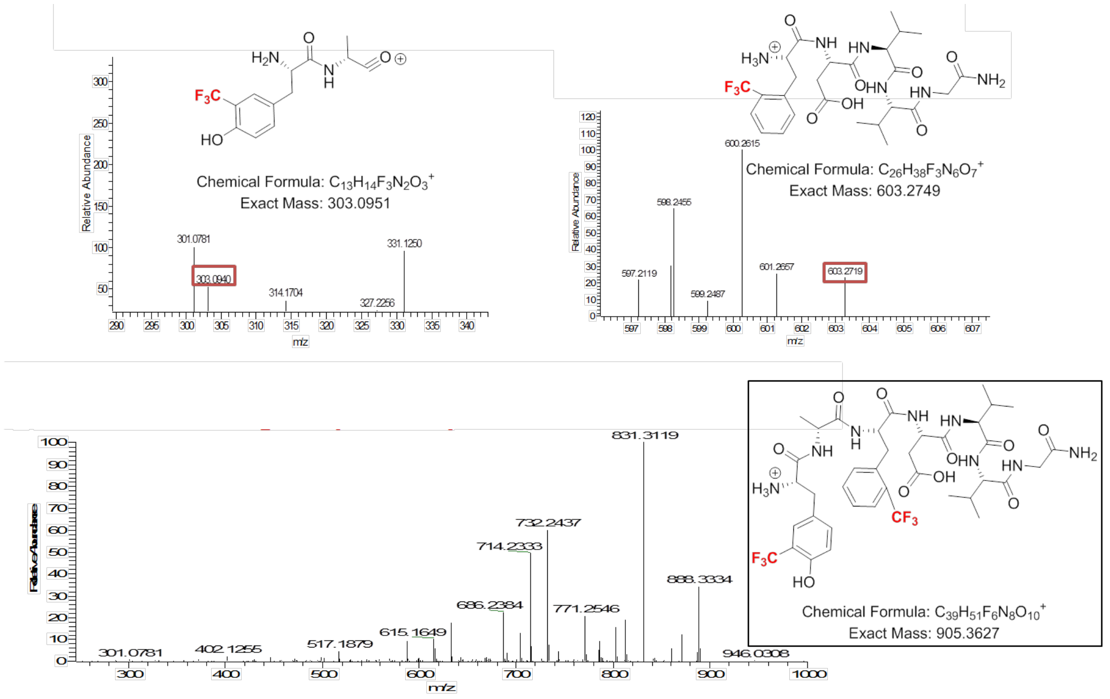

4. *o*-CF_3[Y]-*m*-CF_3[F] deltorphin I **S12**

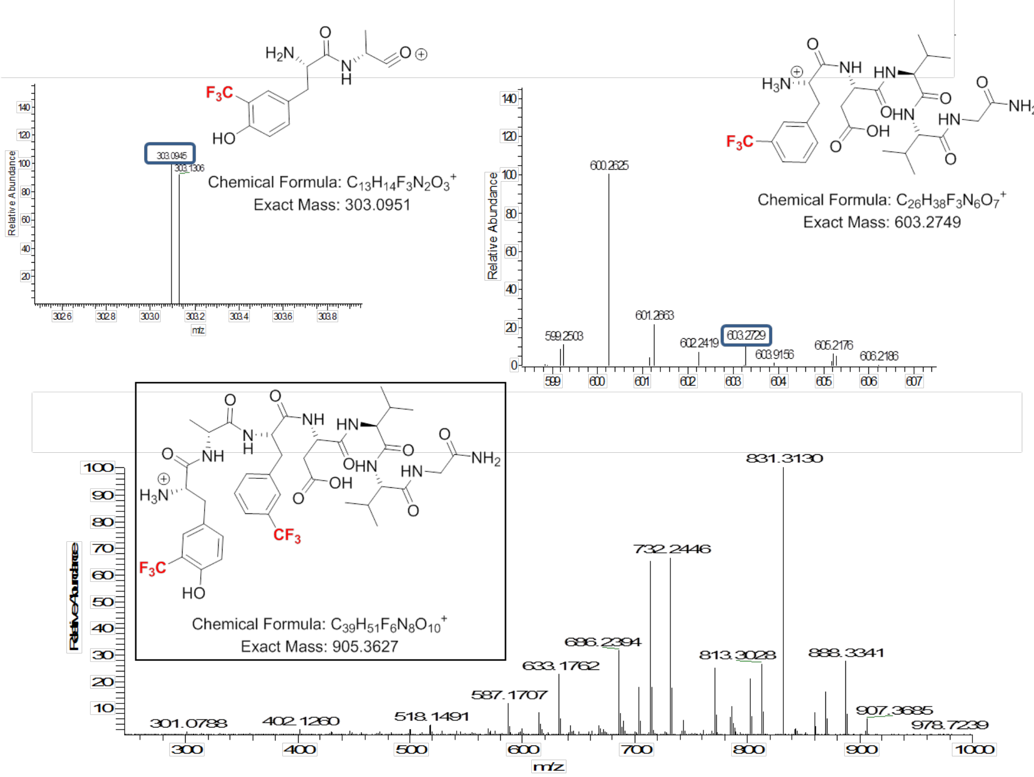

5. *o*- CF_3[Y]-*p*-CF_3[F] deltorphin I **S13**

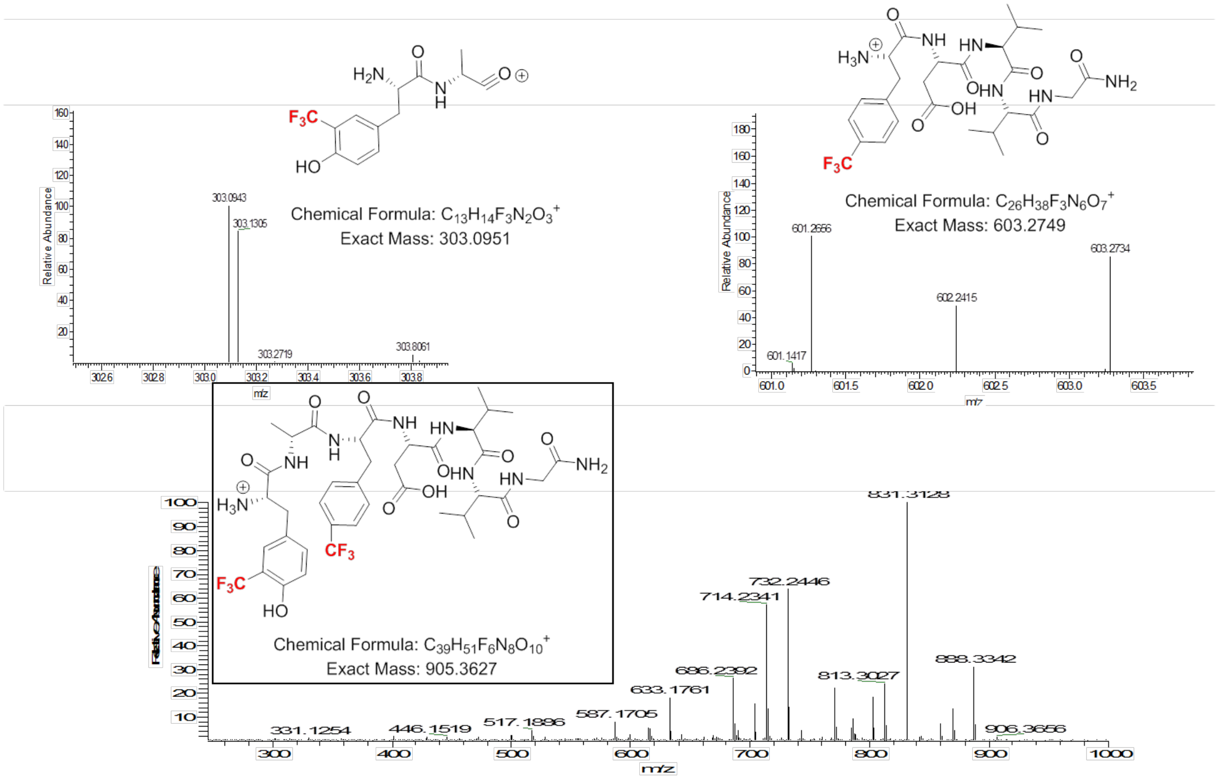

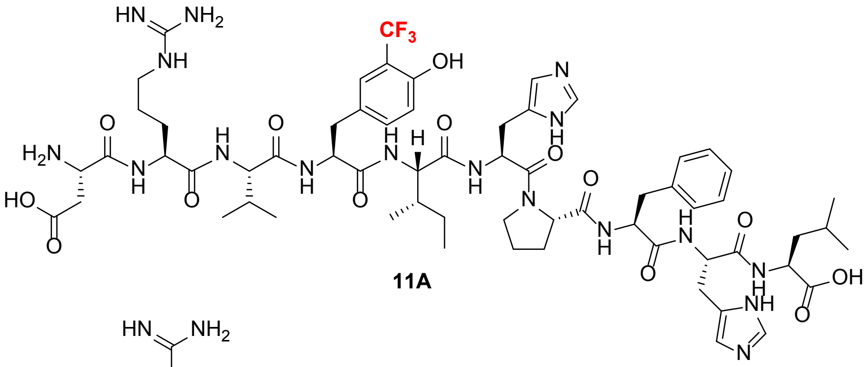

11A

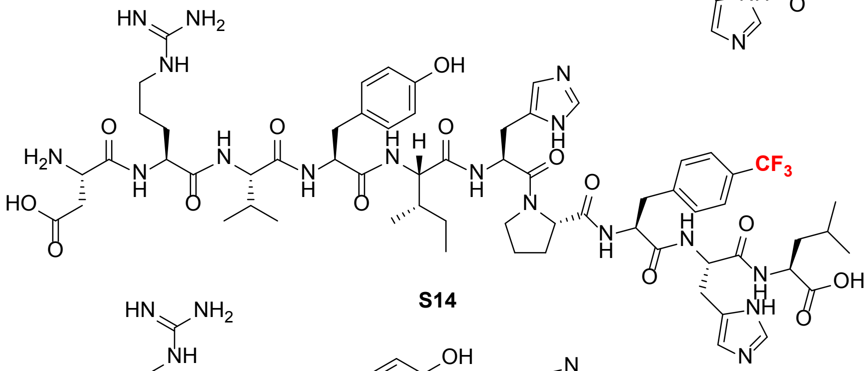

S14

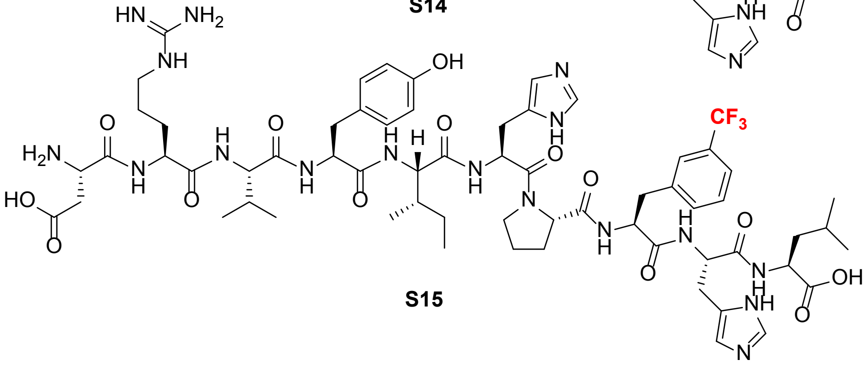

S15

CF_3[Y]-Angiotensin I (**11A**) *and CF_3[F]-Angiotensin I* (**S14 & S15**): To a vial containing angiotensin I (**11**) (100.0 mg, 0.071 mmol) and zinc trifluoromethanesulfinate (70.5 mg, 0.213 mmol, 3 equiv) was added 660 µL of 10% acetic acid in water (final concentration 0.11 M). This mixture was transferred into a N_2-filled glove box. To this solution was added a 70% solution of TBHP in water (49 µL, 0.354 mmol, 5 equiv) in a dropwise manner. The vial was sealed and the mixture was allowed to stand at room temperature for 16 h. The reaction mixture was lyophilized and reconstituted into DMSO. The resulting solution was purified using a Waters Peptide CSH prep column (30 x 150 mm) with a 27 min run time (50 mL/min flow) and a gradient of 78:22 to 67:33 water:acetonitrile with 0.16% TFA. The fractions containing the desired products were combined and lyophilized under reduced pressure to afford 11.0 mg (10.3%) of CF_3[Y]-Angiotensin I (**11A**) and 2.9 mg (2.0%) of CF_3[F]-Angiotensin I (**S14&S15).** The CF_3-Phe modified analogss were an inseparable regioisomeric mixture at the *meta*- and *para*- positions.

CF_3[Y]-Angiotensin I (**11A**): ^{1}H-NMR (500 MHz, methanol-d_4) δ 8.74 (s, 1H), 8.69 (s, 1H), 7.38 (d, *J* = 1.7 Hz 1H), 7.34 (s, 2H), 7.28 (dd, *J* = 8.4, 1.7 Hz, 1H), 7.26 – 7.20 (m, 4H), 7.17 (m,

1H), 6.84 (d, *J* = 8.4 Hz, 1H), 4.91 (t, *J* = 6.4 Hz, 1H), 4.68 – 4.62 (m, 2H), 4.53 (dd *J* = 8.5, 5.9 Hz, 1H), 4.44 – 4.32 (m, 3H), 4.24 (dd, *J* = 4.8, 8.4 Hz, 1H), 4.10 (dd, *J* = 7.4, 2.2 Hz, 2H), 3.77 (dt, *J* = 9.7, 6.8 Hz, 1H), 3.56 (d, *J* = 9.7, 6.8 Hz, 1H), 3.26 (dd, *J* = 15, 6.4 Hz, 1H), 3.22-3.07 (m, 6H), 3.05-2.98 (m, 3H), 2.88 – 2.80 (m, 2H), 2.16 (m, 1H), 2.03-1.88 (m, 3H), 1.88-1.67 (m, 5H), 1.67-1.59 (m, 4H), 1.47 (m, 1H), 1.16 (m, 1H), 0.96 (d, *J* = 6.3 Hz, 3H), 0.93 (d, *J* = 6.1Hz, 3Hz), 0.89 (d, *J* = 6.8 Hz, 3H), 0.85 (m, 9H). ^{13}C-NMR (150 MHz, methanol-d_4) δ 176.41, 174.90, 173.59, 173.49, 173.44, 173.41, 174.39, 173.32, 173.12, 171.69, 169.82, 158.77, 156.00, 138.47, 135.52, 135.12, 135.05, 130.77, 130.56, 130.46 (2C), 129.65 (2C), 128.96, 128.67 (q, *J* = 4.6Hz), 128.00, 126.53 (q, *J* = 270Hz), 119.36, 118.90, 117.99, 117.76 (q, $^2J_{CF}$ = 30Hz), 62.01, 60.71, 59.49, 56.71, 55.97, 54.81, 53.63, 52.62, 51.71, 51.19, 48.60, 42.03, 41.51, 38.08, 37.93, 37.85, 36.55, 32.20, 30.83, 30.07, 27.56, 28.60, 26.29, 26.15, 26.14, 26.12, 23.60, 21.92, 19.88, 19.07, 16.01, 11.40. ^{19}F-NMR (471 MHz, Methanol-d_4) δ -63.36

CF_3[F]-Angiotensin I (**S14 & S15**): **Isomer 1** ^{1}H-NMR (600 MHz, methanol-d_4) δ 8.74 (s, 1H), 8.69 (s, 1H), 7.54 (d, *J* = 7.9 Hz, 2H), 7.45 (d *J* = 7.9 Hz, 2H), 7.36 (s, 1H), 7.33, (s, 1H), 7.03 (d, *J* = 8.4 Hz, 2H), 6.65 (d, *J* = 8.4 Hz, 2H), 4.89 (m, 1H), 4.65 (t, *J* = 5.8Hz. 1H), 4.63 – 4.50 (m, 2H), 4.37 (m, 2H), 4.32 (m, 1H), 4.25 (dd, *J* = 7.6, 5.0 Hz, 1H), 4.08 (m, 2H), 3.77 (m, 1H), 3.57 m, 1H), 3.28-3.21 (m, 2H), 3.27-3.04 (m, 6H), 2.97 (dd, 2H) 2.79 (m, 2H), 2.12 (m, 1H), 1.97 (m, 1H), 1.92 (m, 2H), 1.83-1.54 (m, 9H), 1.40 (m, 1H), 1.11 (m, 1H), 0.96 (d, *J* = 6.3 Hz, 3H), 0.92 (d, *J* = 6.2 Hz, 3H), 0.89 (d, *J* = 6.7 Hz, 3H), 0.87 – 0.82 (m, 9H). **Isomer 2** ^{1}H-NMR (600 MHz, methanol-d_4) δ 8.74 (s, 1H), 8.70 (s, 1H), 7.55 (s, 1H), 7.52 (d, *J* = 7.8 Hz 1H), 7.50 (d, *J* = 7.8 Hz, 1H), 7.45 (t, *J* = 7.8 Hz, 1H), 7.36 (s, 1H), 7.33 (s, 1H), 7.03 (d, *J* = 8.4 Hz, 2H), 6.65 (d, *J* = 8.4 Hz, 2H), 4.89 (m, 1H), 4.65 (t, *J* = 5.8Hz. 1H), 4.63 – 4.50 (m, 2H), 4.37 (m, 2H) 4.32 (m, 1H), 4.25 (dd, *J* = 7.6, 5.0 Hz, 1H), 4.08 (m, 2H), 3.77 (m, 1H), 3.57 m, 1H), 3.28-3.21 (m, 2H), 3.27-3.04 (m, 6H), 2.97 (dd, 2H) 2.79 (m, 2H), 2.12 (m, 1H), 1.97 (m, 1H), 1.92 (m, 2H), 1.83-1.54 (m, 9H), 1.40 (m, 1H), 1.11 (m, 1H), 0.96 (d, *J* = 6.3 Hz, 3H), 0.92 (d, *J* = 6.2 Hz, 3H), 0.89 (d, *J* = 6.7Hz, 3H), 0.87 – 0.82 (m, 9H). ^{13}C-NMR is unavailable due to an insufficient quantity of isolated trifluoromethylated product. ^{19}F-NMR (471 MHz, methanol-d_4) -63.83, -63.84.

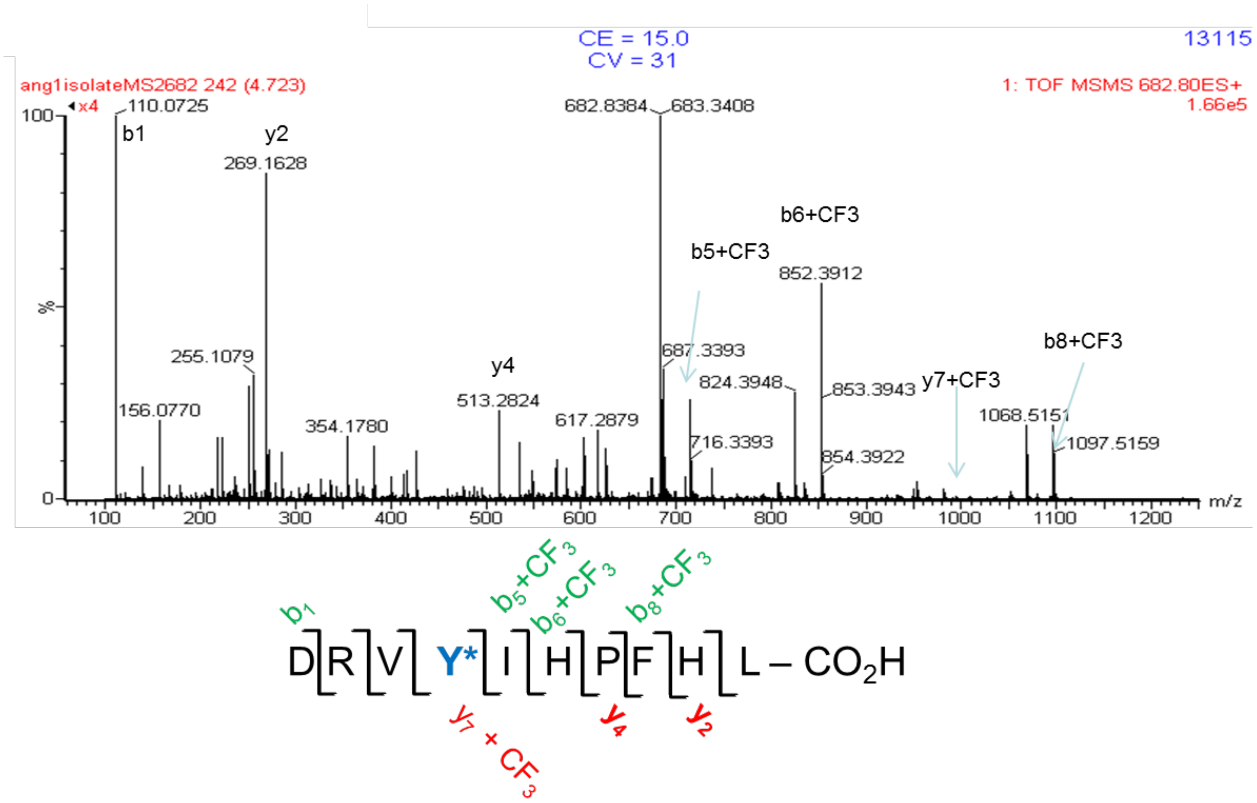

HRMS LC/MS/MS Analysis of Modified CF_3-modified Angiotensin I. Tandem mass spectrum and corresponding sequence maps resulting from the collision-induced dissociation (CID) of singly charged positive ion at m/z = 1364.635. This signal was due to $[M+H]^+$ of angiotensin I modified with CF_3. The *b* ions are shown in green and the *y* ions are shown in red. Fragment masses were consistent with modification of angiotensin I at Tyr4.

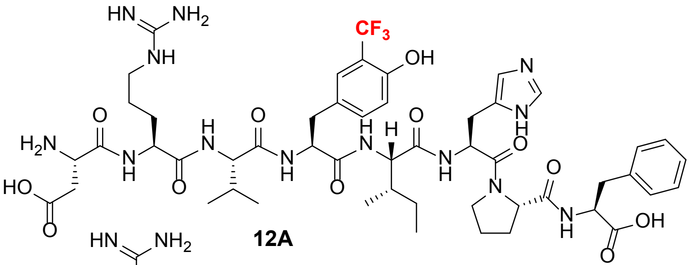

12A

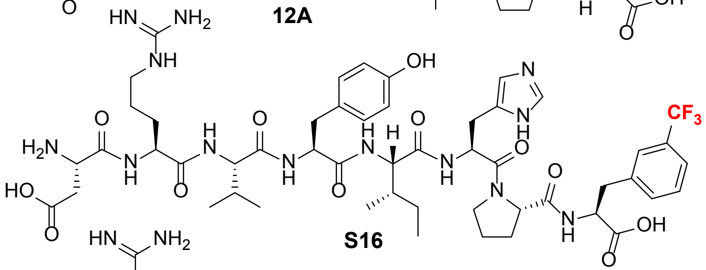

S16

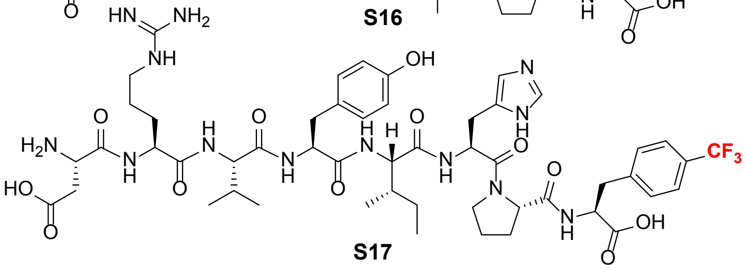

S17

CF_3[Y]-Angiotensin II (**12A**) *and CF_3[F]-Angiotensin II* (**S16 & S17**): To a vial containing angiotensin II·TFA (100.0 mg, 0.071 mmol) and zinc trifluoromethanesulfinate (95 mg, 0.287 mmol, 3 equiv) was added 1550 µL of 10% acetic acid in water (final concentration 0.046 M). This mixture was transferred into a N_2-filled glove box. To this solution was added a 70% solution of TBHP in water (49 µL, 0.354 mmol, 5 equiv) in a dropwise manner. The vial was sealed and the mixture was allowed to stand at room temperature for 16 h. The reaction mixture was lyophilized and reconstituted into DMSO. The resulting solution was purified using a Waters Peptide CSH prep column (30 x 150 mm) with a 27 min run time (50 mL/min flow) and a gradient of 81:19 to 69:31 water:acetonitrile with 0.16% TFA. The fractions containing the desired products were combined and lyophilized under reduced pressure to afford 9.0 mg (8.1%) CF_3[Y]-Angiotensin II (**12A**) and 2.0 mg (2.0%) of CF_3[F]-Angiotensin II (**S16 & 17**).

CF_3[Y]-Angiotensin II (**12A**): ^{1}H-NMR (600 MHz, methanol-d_4) δ 8.69 (s, 1H), 7.38 (d, J = 1.7 Hz, 1H), 7.34 (s, 1H), 7.30-7.22 (m, 5H), 7.18 (t, J = 7.2 Hz, 1H), 6.85 (d, J = 8.3 Hz, 1H), 4.89 (t, J = 6.1 Hz, 1H), 4.66 (t, J = 5.1 Hz, 1H), 4.64 (t, J = 5.1 Hz, 1H), 4.47 (m, 1H), 4.39 (dd, J = 7.7, 5.4 Hz, 1H), 4.25 (d, J =8.1, 4.7 Hz, 1H), 4.12 (m, 2H), 3.78 (m, 1H), 3.50 (m, 1H), 3.25-3.15 (m, 4H), 3.14-3.03 (m, 3H), 2.99 (dd, J = 17, 4.7 Hz, 1H), 2.9-2.78 (m, 2H), 2.24 (m, 1H), 1.90-2.04 (m, 4H), 1.86-1.68 (m, 3H), 1.62 (m, 2H), 1.49 (m, 1H), 1.13 (m, 1H), 0.89 (d, J = 6.7 Hz, 3H), 0.87 – 0.84 (m, 9H). ^{13}C-NMR (150 MHz, methanol-d_4) δ 174.81, 174.52, 173.54, 173.47, 173.39, 173.30, 173.28, 170.36, 169.81, 158.79, 155.99, 138.40, 135.50, 135.20, 130.63 (2C), 130.06, 129.58 (2C), 128.92, 128.67 (q, J = 4.7 Hz), 127.95, 125.6 (q, J = 271Hz), 119.54, 118.00, 117.79 (q, J = 29.4 Hz), 61.66, 60.61, 59.40, 55.93, 55.67, 54.80, 51.67, 51.22,

48.87, 42.04, 38.33, 38.01, 37.92, 36.56, 32.26, 30.90, 30.11, 27.62, 26.28, 26.14, 26.07, 19.87, 19.06, 15.97, 11.40. ^{19}F-NMR (471 MHz, methanol-d_4) δ -63.96.

CF_3[F]-Angiotensin II (containing ~15% of CF_3[Y]-Angiotensin II) (**S16 & S17**): **Isomer 1** ^{1}H-NMR (600 MHz, methanol-d_4) δ 8.73 (s, 1H), 7.56 (d, *J* = 8.2 Hz, 2H), 7.49 (d, *J* = 8.2 Hz, 2H), 7.34 (s, 1H), 7.03 (d, *J* = 8.3 Hz, 2H), 6.65 (d, *J* = 8.3 Hz, 2H), 4.88 (m, 1H) 4.71 (m, 1H), 4.61 (t, *J* = 6.9 Hz, 1H), 4.46 (m, 1H), 4.40 (dd, *J* = 7.8, 5.6 Hz, 1H), 4.25 (dd, *J* = 8.1, 4.7 Hz, 1H), 4.15 (d, *J* = 7.5 Hz, 1H), 4.10 (dd, *J* = 8.2, 3.2 Hz, 1H), 3.81 (m, 1H), 3.51 (m, 1H), 3.26 (m, 1H), 3.22 - 3.09 (m, 5H), 2.83 (m, 2H), 2.25 (m, 2H), 1.97 (m ,1H), 2.04 -1.92 (m, 4H), 1.82 -1.67 (m, 3H), 1.62 (m, 2H), 1.48 (m, 1H), 1.13 (m, 1H), 0.9 (d, *J* = 6.62 Hz, 3H) 0.87 (m, 9H). **Isomer 2** ^{1}H-NMR (600 MHz, methanol-d_4) δ 8.73 (s, 1H), 7.59 (s, 1H), 7.58 (d, *J* = 7.8 Hz, 1H), 7.5 (d, *J* = 7.8 Hz, 1H), 7.45 (t, *J* = 7.8 Hz, 1H), 7.35 (s, 1H), 7.03 (d, *J* = 8.3 Hz, 2H), 6.65 (d, *J* = 8.3 Hz, 2H), 4.88 (m, 1H) 4.71 (m, 1H), 4.61 (t, *J* = 6.9 Hz, 1H), 4.46 (m, 1H), 4.40 (dd, *J* = 7.8, 5.6 Hz, 1H), 4.25 (dd, *J* = 8.1, 4.7 Hz, 1H), 4.15 (d, *J* = 7.5 Hz, 1H), 4.10 (dd, *J* = 8.2, 3.2 Hz, 1H), 3.81 (m, 1H), 3.51 (m, 1H), 3.26 (m, 1H), 3.22 - 3.09 (m, 5H), 2.83 (m, 2H), 2.25 (m, 2H), 1.97 (m ,1H), 2.04 -1.92 (m, 4H), 1.82 -1.67 (m, 3H), 1.62 (m, 2H), 1.48 (m, 1H), 1.13 (m, 1H), 0.9 (d, *J* = 6.6 Hz, 3H) 0.87 (m, 9H). ^{13}C-NMR is unavailable due to an insufficient quantity of isolated trifluoromethylated product. ^{19}F-NMR (471 MHz, methanol-d_4) δ -63.88.

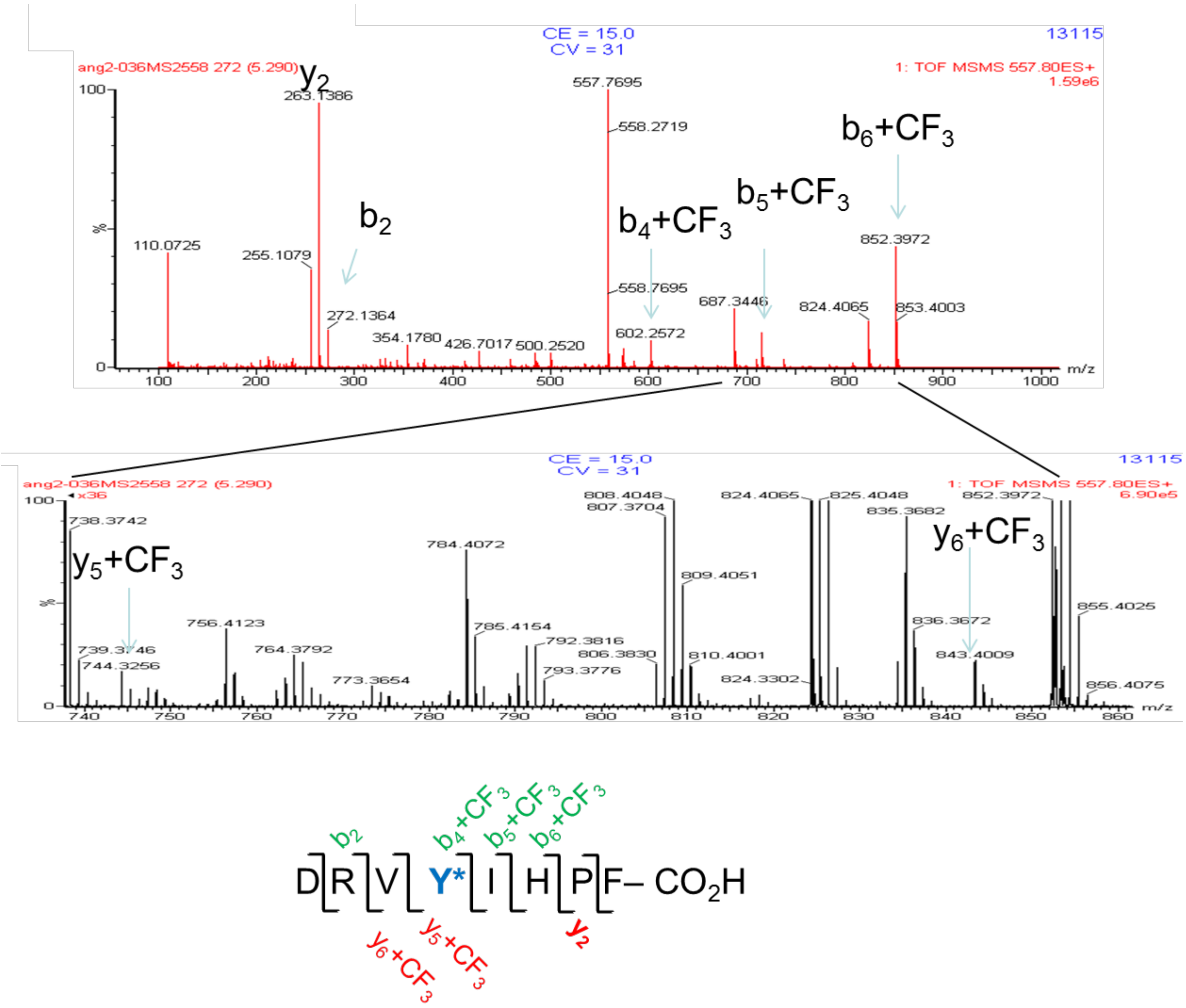

HRMS LC/MS/MS Analysis of Modified CF_3-modified Angiotensin II. Tandem mass spectrum (Waters G2 Q-TOF) and corresponding sequence maps resulting from collision-induced dissociation (CID) of singly charged positive ion at m/z = 1114.5293. This signal was due to the $[M+H]^+$ of angiotensin II modified with CF_3. The *b* ions are shown in green and the *y* ions are shown in red. Fragment masses were consistent with modification of angiotensin II at Tyr4.

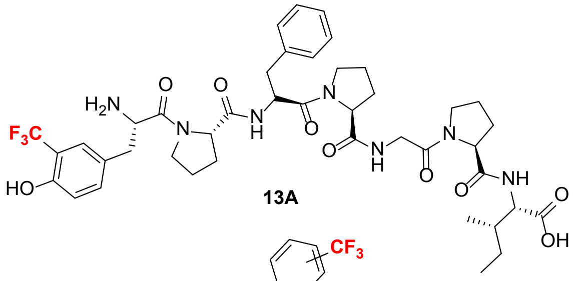

13A

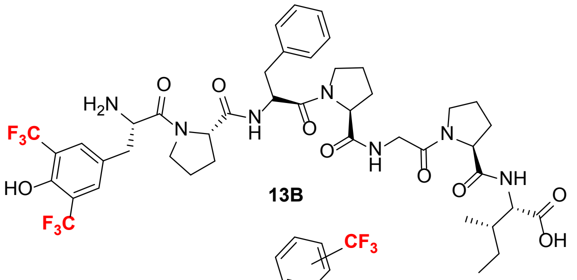

13B

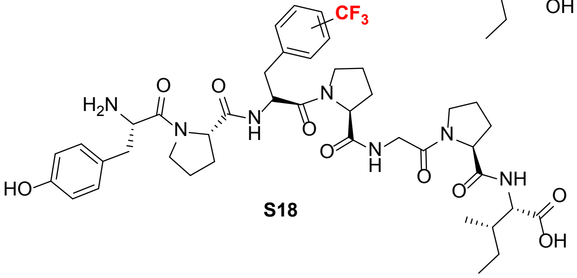

S18

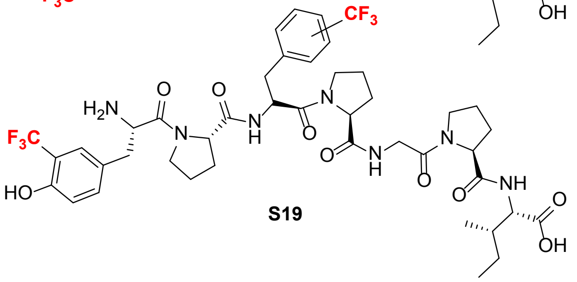

S19

CF_3[Y]-β-casomorphin (**13A**), *CF_3[F]-β-casomorphin* (**S18**), *$(CF_3)_2$-β-casomorphin* (**13B** and **S19**): In a N_2-filled glove box, *β-casomorphin* (50 mg, 63 μmol, 1 equiv), [Ir(dFCF$_3$ppy)$_2$(dtbpy)](PF$_6$) (cat. **4**, 9.5 μmol, 15 mol%) and $NaSO_2CF_3$ (198 mg, 1270 μmol, 20 equiv) were added to a 4 mL vial with a stir bar. To the reaction were added 1060μL CH_3CN and 1060 μL aqueous 10% AcOH. The vial was sealed and placed in the MSD photoreactor[1] and irradiated with the following settings: fan rate 4700 rpm; blue LED 100% (13.2 W); stir rate 1000 rpm. The reaction was irradiated for 16 h. The reaction mixture was diluted with EtOAc (3 mL) and 1 mL of H_2O was added. The ethyl acetate layer was washed with H_2O three times and the combined aqueous layers were lyophilised. The reaction mixture was re-constituted with 2 mL of DMSO. The resulting solution was purified using a Waters Peptide CSH prep column (30 x 150 mm) with a 25 min run time (50 mL/min flow) and a gradient of 76:24 to 62:38 water:acetonitrile with 0.16% TFA. The fractions containing desired products were combined and lyophilized under reduced pressure to afford 9.4 mg (17%) of mono CF_3-*β*-casomorphin (1:1 = CF_3[F] **S18**:*o*-CF_3[Y] **13A**) and 6.1 mg (10%) of bis $(CF_3)_2$-*β*-casomorphin (1.6:3.1 = *o,o*-CF_3[Y] **13B**:*o*-CF_3[Y]-*p*-CF_3[F] **S19**) as a mixture of tyrosine and phenylalanine modified products. The isolated yields were low due to the goal of maximizing the purity of the desired products for structural characterization. Due to the presence of proline rotamers in these structures, it was difficult to interpret each NMR for the connectivity of the trifluoromethyl groups. HRMS LC/MS/MS analyses are reported herein to confirm the structure of the final products.

HRMS LC/MS/MS **Analysis (**Thermo Orbitrap MS System**)**:

1. CF_3[Y]-*β*-casomorphin **(13A)**

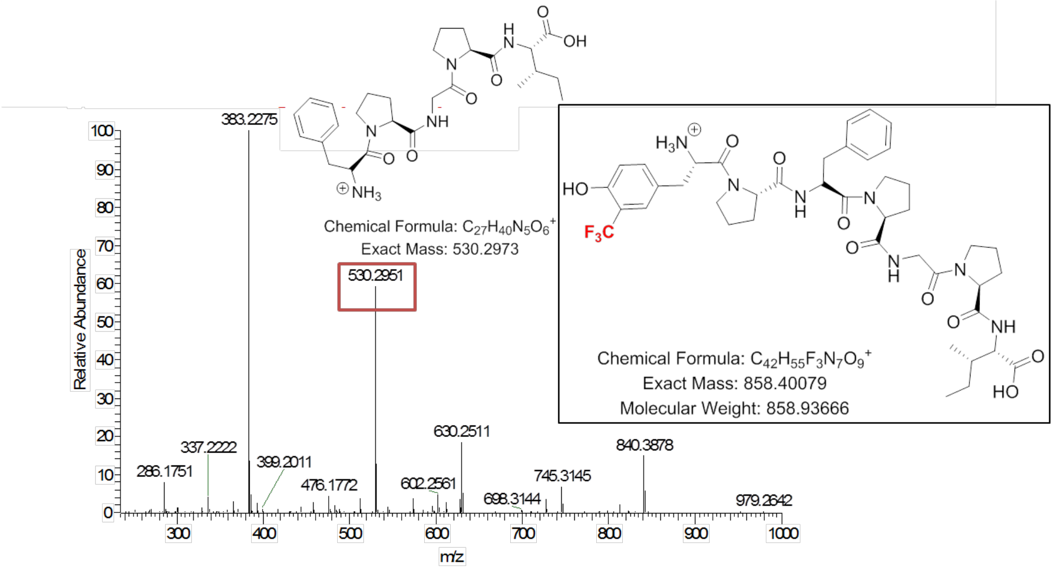

2. CF_3[F]-*β*-casomorphin (**S18**)

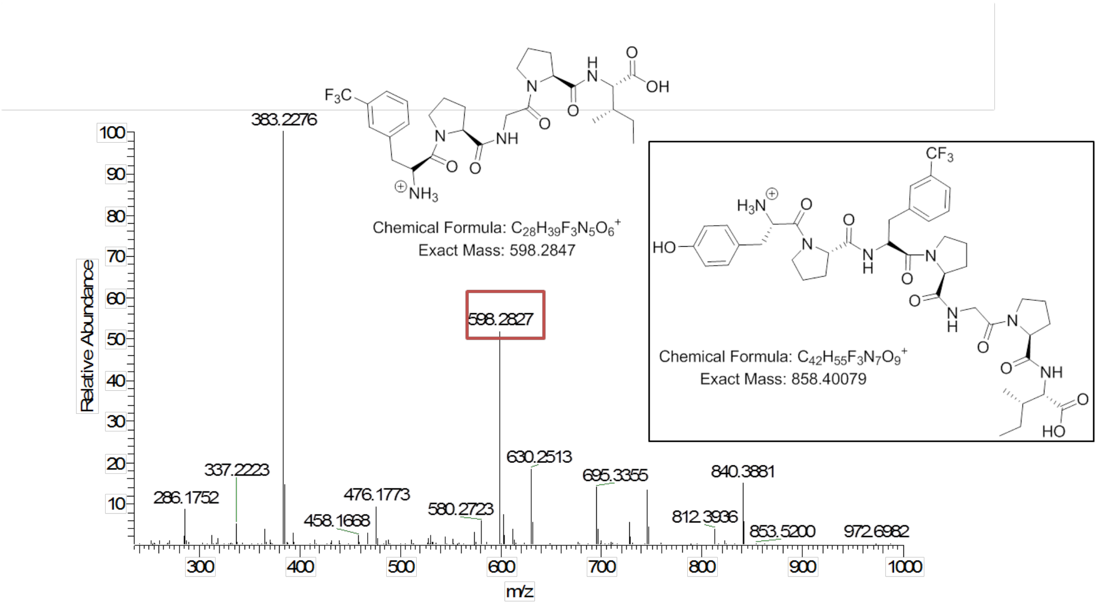

3. *o,o*-CF_3[Y]-*β*-casomorphin (**13B**)

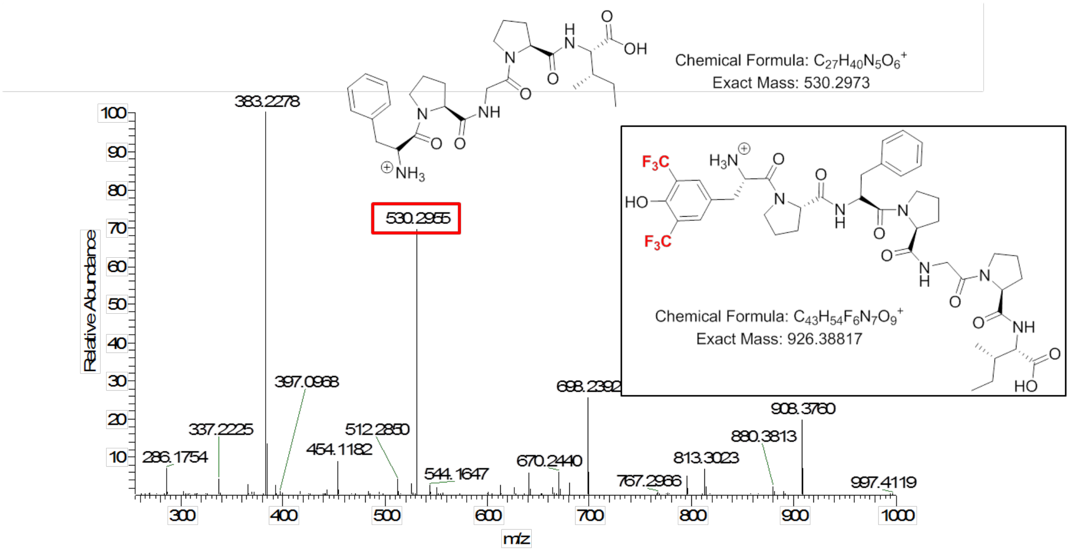

4. *o*-CF_3[Y]-CF_3[F]-*β*-casomorphin (**S19**)

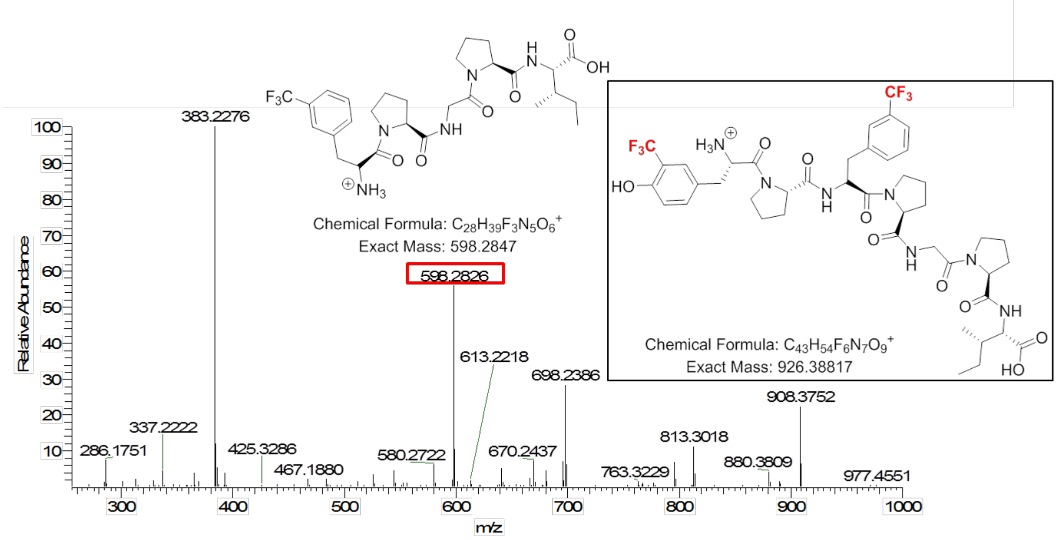

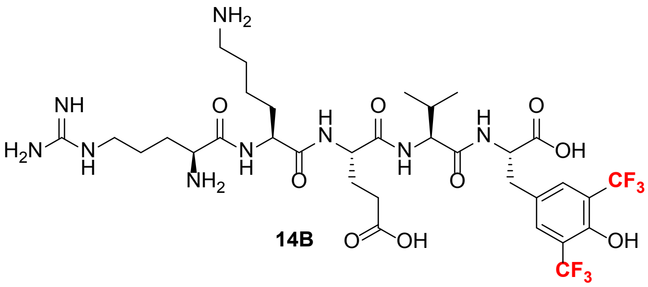

14B

(CF_3)$_2$[Y]-Splenopentin (***14B***): To a 1 mL vial containing splenopentin (**14**) (20.0 mg, 0.029 mmol) and zinc trifluoromethanesulfinate (28.7 mg, 0.086 mmol, 3 equiv) was added 250 µL of 10% acetic acid in water (final concentration 0.116 M). This mixture was transferred into a N_2-filled glove box. To this solution was added a 70% solution of TBHP in water (14 µL, 0.144 mmol, 5 equiv) in a dropwise manner. The vial was sealed and the mixture was allowed to stand at room temperature for 16 h. The reaction mixture was lyophilized and reconstituted into DMSO. The resulting solution was purified using a Waters Peptide CSH prep column (30 x 150mm) with a 25 min run time (50 mL/min flow) and a gradient of 84:16 to 79:21 water:acetonitrile with 0.16% TFA. The fractions with the desired products were combined and lyophilized under reduced pressure to afford 4.0 mg (15%) of bis-trifluoromethylated product **14B.**

14B ^{1}H-NMR (500 MHz, methanol-*d*$_4$) δ 7.66 (s, 2H), 4.62 (dd, *J* = 8.3, 5.9 Hz, 1H), 4.43 (dd, *J* = 9.3, 5.0 Hz, 1H), 4.35 (dd, *J* = 8.3, 5.9 Hz, 1H), 4.15 (t, *J* = 7.1 Hz, 1H), 3.99 (t, *J* = 6.1 Hz, 1H), 3.21 (m, 3H), 3.05 (dd, *J* = 14.1, 8.2 Hz, 1H), 2.93 (t, *J* = 7.4 Hz, 2H), 2.40 (m, 2H), 2.10 (m, 1H), 2.01 (m, 1H), 1.97 – 1.80 (m, 4H), 1.77-1.62 (m, 5H), 1.50 (b, 2H), 0.88 (d, *J* = 6.9 Hz, 3H), 0.87 (d, *J* = 6.9 Hz, 3H). ^{13}C-NMR is unavailable due to an insufficient amount of isolated product. ^{19}F-NMR (471 MHz, methanol-*d*$_4$) δ -63.03. HRMS (ESI) $[M+H]^+$ calculated for $C_{33}H_{50}F_6N_9O_9^+$ 830.3636, found 830.3644.

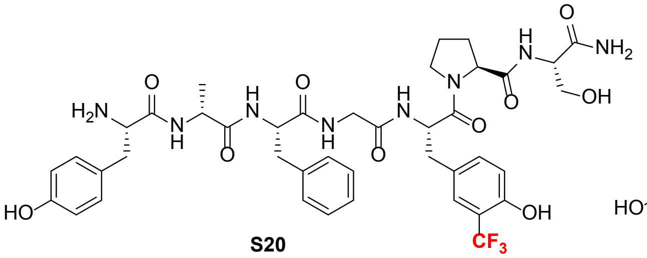

S20

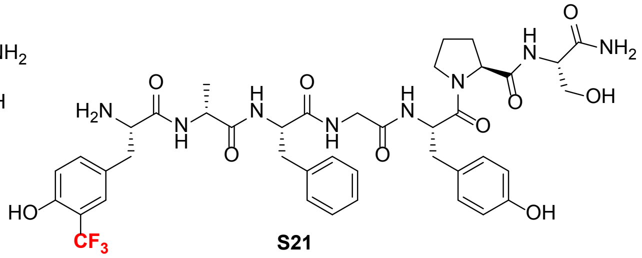

S21

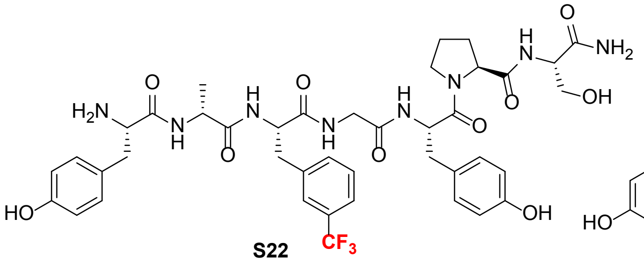

S22

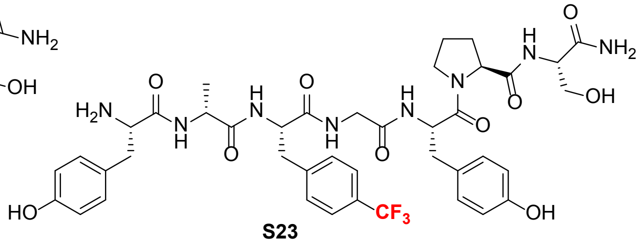

S23

Mixture of CF_3[Y5]-dermorphin, CF_3[Y1]- dermorphin and CF_3[F3]-dermorphin (**S20-23**): To a vial containing dermorphin (**15**) (20.0 mg, 0.025 mmol) and zinc trifluoromethansulfinate (24.7 mg, 0.075 mmol, 3 equiv) was added 250 µL of 10% acetic acid in water (final concentration 0.10 M). This mixture was transferred into a N_2-filled glove box. To this solution was added a 70% solution of TBHP in water (14 µL, 0.144 mmol, 5 equiv) in a dropwise manner. The vial was sealed and the mixture was allowed to stand at room temperature for 16 h. The reaction mixture was lyophilized and reconstituted into DMSO. The resulting solution was purified using a Waters Peptide CSH prep column (30 x 150 mm) with a 28 min run time (50 mL/min flow) and a gradient of 76:24 to 65:35 water:acetonitrile with 0.16% TFA. The combined fractions were lyophilized under reduced pressure to afford 1.5 mg (5.0%) of mono-trifluoromethylated product as a mixture of regioisomers (**S20-23**). NMR spectroscopic analysis of the purified material indicated that it contains mostly CF_3[Y5]- dermophin (**S20**) along with minor amounts of a mixture of CF_3[Y1]-Dermorphin (**S21**) along with CF_3[F3]-Dermorphin isomers **S22-S23**.

Mixture of CF_3[F3]-dermorphin and CF_3[Y]-dermorphin (**S20-S23**): ^{1}H-NMR (500 MHz, methanol-d_4) δ 7.41 (s, 1H), 7.36 (d, J = 10.9 Hz, 1H), 7.34 – 7.29 (m, 2H), 7.29 – 7.17 (m, 9H), 7.10 (d, J = 8.4 Hz, 1H), 7.05 (dd, J = 8.2, 4.5 Hz, 4H), 6.92 (dd, J = 8.2, 4.5 Hz, 1H), 6.87 (d, J = 8.5 Hz, 1H), 6.78 – 6.69 (m, 4H), 4.84 – 4.73 (m, 1H), 4.66 (t, J = 8.1 Hz, 1H), 4.60 (dd, J = 10.9, 4.3 Hz, 1H), 4.57 – 4.50 (m, 1H), 4.46 (dt, J = 8.7, 4.4 Hz, 1H), 4.38 (t, J = 4.8 Hz, 1H), 4.26 (t, J = 5.3 Hz, 1H), 4.20 – 4.10 (m, 1H), 4.00 (q, J = 7.4 Hz, 2H), 3.95 – 3.68 (m, 6H), 3.65 (d, J = 5.3 Hz, 1H), 3.63 – 3.53 (m, 2H), 3.51 – 3.45 (m, 2H), 3.36 – 3.31 (m, 2H), 3.30 (dq, J = 3.6, 1.8 Hz, 17H), 3.24 (m, 1H), 3.03 (m, 4H), 2.84 (m, 3H). ^{13}C-NMR is unavailable due to an insufficient amount of isolated trifluoromethylated product. ^{19}F-NMR (471 MHz, methanol-d_4) δ -63.46 [Y5], -63.66 [Y1], -63.82 [F3], -63.84 [F3] (reference to TFA at -76.90).

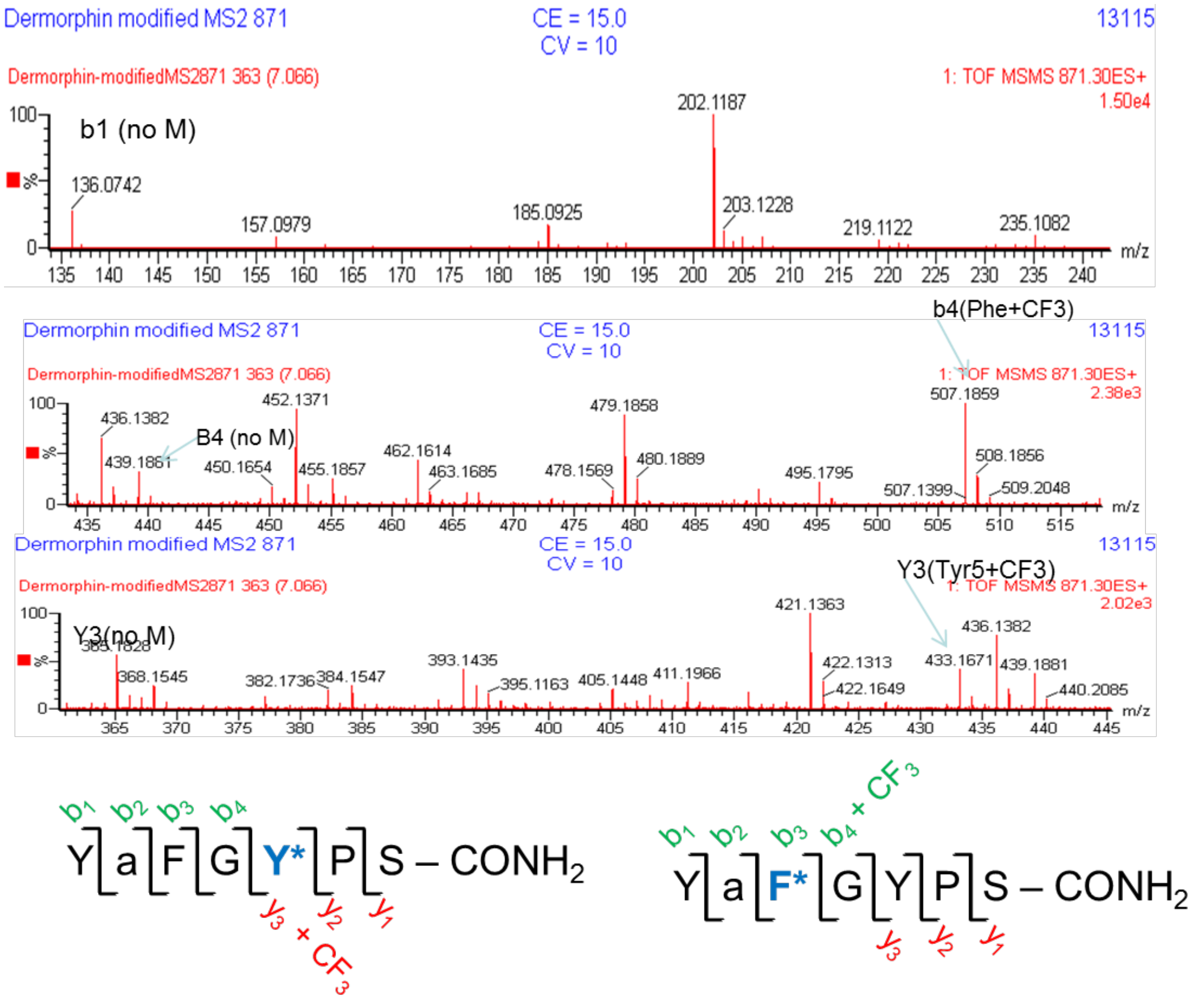

HRMS LC/MS/MS Analysis of CF_3-modified Dermorphin. Tandem mass spectrum (Waters G2 Q-TOF system) and corresponding sequence maps resulting from the collision-induced dissociation (CID) of singly charged positive ion at m/z = 871.3594. This signal was due to the $[M+H]^+$ of dermorphin modified with CF_3. The *b* ions are shown in green and the *y* ions are shown in red. Fragment masses were consistent with modification at both Tyr5 and Phe3 of dermorphin.

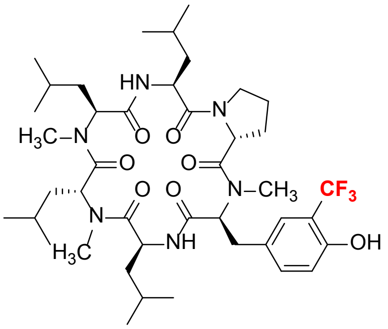

16A

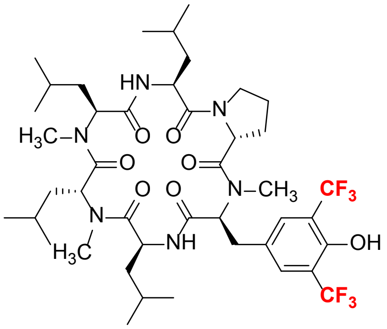

16B

CF_3[Y]-cyclic peptides **16A** and **16B**. In a N_2-filled glove box, cyclic peptide **16** (60 mg, 78 µmol, 1 equiv), [Ir(dFCF$_3$ppy)$_2$(dtbpy)](PF$_6$) (cat**. 4,** 12 µmol, 15 mol%) and $NaSO_2CF_3$ (244 mg, 1560 µmol, 20 equiv) were added to a 4 mL vial with a stir bar. To this reaction were added 2000 µL of ethyl acetate and 2000 µL of aqueous 10% AcOH (0.02 M). The vial was sealed and placed in the MSD photoreactor[1] and irradiated with the following settings: fan rate 4700 rpm; blueLED 100% (13.2W); stir rate 1000 rpm. The reaction was irradiated for 16 h. The reaction mixture was lyophilized and reconstituted into DMSO and filtered prior to purification. The resulting solution was purified using a SunFire Prep C18 OBD column (30 x 150 mm) with a 20 min run time (30 mL/min flow) and a gradient of 10-100% acetonitrile/water with 0.05% TFA. The fractions containing desired products were combined and lyophilized under reduced pressure to afford 3.6 mg (2.8%) of mono CF_3[Y]-cyclic peptide **16A** with ~50% purity and 6.4 mg (7.8%) of $(CF_3)_2$-cyclic peptide **16B** with ~90% purity.

16A ^{1}H-NMR (500 MHz, methanol-d_4) δ 7.98 (d, J = 9.5 Hz, 1H), 7.82 (d, J = 9.5 Hz, 1H), 7.32 (d, J = 2.2 Hz, 1H), 7.27 (d, J = 8.2 Hz, 1H), 7.00 (d, J = 8.9 Hz, 1H), 6.86 (d, J = 8.4 Hz, 1H), 5.61 (t, J = 12.5 Hz, 1H), 5.28 (m, 1H), 5.22 – 5.05 (m, 2H), 4.90 (m, 1H) 4.69 (m, 1H), 3.91 – 3.61 (m, 2H), 3.4 (m, 1H), 3.23 (s, 3H), 3.15 (s, 3H), 3.03 (s, 3H), 2.95 – 2.84 (m, 1H), 2.36 – 1.87 (m, 7H), 1.79 – 1.41 (m, 10H), 1.20 – 0.81 (m, 24H). ^{19}F-NMR (471 MHz, methanol-d_4) δ -63.60. ^{13}C-NMR is unavailable due to an insufficient quantity of isolated trifluoromethylated product. HRMS (ESI) [M+H]$^+$ calculated for $C_{42}H_{66}F_3N_6O_7^+$ 823.4940; found 823.4929.

16B ^{1}H-NMR (500 MHz, methanol-d_4) δ 7.86 (d, J = 9.5 Hz, 1H), 7.64 (s, 2H), 6.97 (d, J = 8.9 Hz, 1H), 5.67 (dd, J = 4.6, 11.9 Hz, 1H), 5.27 (dd, J = 4.0, 11.8 Hz, 1H), 5.20 – 5.00 (m, 2H), 4.89 (m, 1H), 4.59 (t, J = 7.1 Hz, 1H), 3.81 – 3.58 (m, 2H), 3.52 (dd, J = 4.7, 15.3 Hz, 1H), 3.22 (s, 3H), 3.14 (s, 3H), 3.06 – 2.94 (m, 4H), 2.1 (M, 1H), 1.97 – 1.83 (m, 6H), 1.77 – 1.38 (m, 10H), 1.11 – 0.80 (m, 24H). ^{13}C-NMR (125 MHz, methanol-d_4) δ 174.80, 174.01, 172.85, 170.21, 170.20, 168.98, 151.80, 130.56 (d, J = 5.3 Hz), 129.83, 123.40 (q, J = 271Hz), 120.63 (q, J = 30.2 Hz), 56.80, 55.96, 55.04, 52.55, 48.66, 47.81, 47.18, 41.80, 41.36, 37.12, 36.07, 31.61, 30.54, 30.29, 30.10, 27.69, 25.12, 24.98, 24.82, 24.69, 24.56, 22.49, 22.16, 21.98 21.75,

21.52, 21.29, 20.71, 19.94. ^{19}F-NMR (471 MHz, methanol-d_4) δ -62.99. HRMS (ESI) $[M+H]^+$ calculated for $C_{43}H_{65}F_6N_6O_7^+$ 891.4813; found 891.4869.

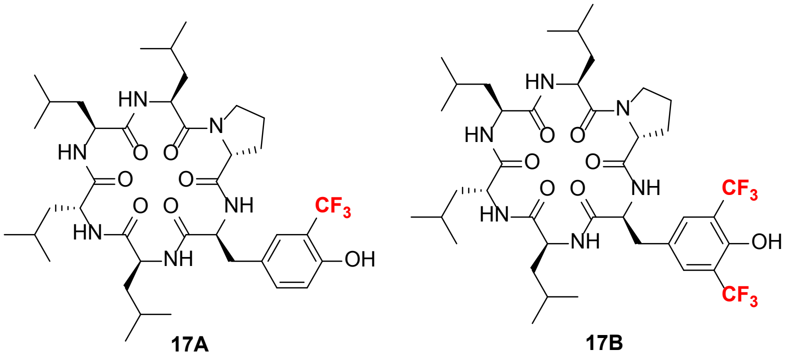

17A **17B**

*CF_3[Y]-cyclic peptides **17A** and **17B** .* In a N_2-filled glove box, cyclic peptide **17** (25 mg, 33 μmol, 1 equiv), [Ir(dFCF$_3$ppy)$_2$(dtbpy)](PF$_6$) (cat. **4**, 4.9 μmol, 15 mol%) and $NaSO_2CF_3$ (102 mg, 651 μmol, 20 equiv) were added to a 4 mL vial with a stir bar. To this reaction were added 543 μL of EtOAc and 543 μL of aqueous 10% AcOH. The vial was sealed and placed in the MSD photoreactor[1] and irradiated with the following settings: fan rate 4700 rpm; blueLED 100% (13.2 W); stir rate 1000 rpm. The reaction was irradiated for 16 h. The reaction mixture was lyophilized and reconstituted into DMSO. The resulting solution was purified using a SunFire Prep C18 OBD column (30 x 150 mm) with a 20 min run time (30 mL/min flow) and a gradient of 10-100% acetonitrile/water with 0.05% TFA. The fractions containing desired products were combined and lyophilized under reduced pressure to afford 6.5 mg (10%) of mono CF_3[Y]-cyclic peptide **17A** in ~90% purity and 13 mg (19%) of $(CF_3)_2$-cyclic peptide **17B** in ~90% purity.

17A ^{1}H-NMR (500 MHz, methanol-d_4) δ 7.39 (d, *J* = 2.2 Hz, 1H), 7.30 (dd, *J* = 2.3, 8.4 Hz, 1H), 6.89 (d, *J* = 8.4 Hz, 1H), , 4.92 (t, *J* = 6.8 Hz, 1H), 4.57 (dd, *J* = 5.5, 8.6 Hz, 1H), 4.45 – 4.33 (m, 2H), 4.24 (m, 2H), 3.81 – 3.58 (m, 2H), 3.36 (m, 1H), 3.01 – 2.80 (m, 1H), 2.10 – 2.00 (m, 3H), 1.76 – 1.55 m, 13H), 1.08 – 0.84 (m, 24H). ^{13}C-NMR data is unavailable due to low isolated sample quantity of **17A.** ^{19}F-NMR (471 MHz, methanol-d_4) δ -63.64. HRMS (ESI) $[M+H]^+$ mono-CF_3 calculated for $C_{39}H_{60}F_3N_6O_7^+$, 781.4470; found 781.4474.

17B ^{1}H-NMR (500 MHz, methanol-d_4) δ 7.60 (s, 2H), 4.96 – 4.90 (m, 1H), 4.58 (dd, *J* = 6.5, 8.5 Hz, 1H), 4.43 (dd, *J* = 3.7, 10.9 Hz, 1H), 4.40 – 4.33 (m, 1H), 4.25 (dd, *J* = 3.7, 10.9 Hz, 1H), 4.19 (dd, *J* = 6.5, 9.8 Hz, 1H), 3.76 (dd, *J* = 6.5, 9.8 Hz, 1H), 3.69 – 3.60 (m, 1H), 3.39 (m, 1H), 3.00 – 2.90 (m, 1H), 2.10 – 2.00 (m, 2H), 1.97 – 1.88 (m, 1H), 1.66 – 1.52 (m, 13H), 1.03 – 0.86 (m, 24H). ^{13}C-NMR (125 MHz, methanol-d_4) δ 173.64, 173.00, 172.46, 172.1, 171.38, 170.55, 130.83, 120.76, 79.47 – 75.22 (m), 60.71, 54.91, 52.53, 52.10, 51.38, 41.51, 40.36, 39.91, 39.28, 34.57, 28.41, 24.75, 24.71, 24.57, 24.50, 24.40, 22.26, 21.88, 21.50, 21.22, 21.12, 19.88. ^{19}F-NMR (471 MHz, methanol-d_4) δ -63.11. HRMS (ESI) $[M+H]^+$ calculated for $C_{40}H_{59}F_6N_6O_7^+$, 849.4344; found 849.4296.

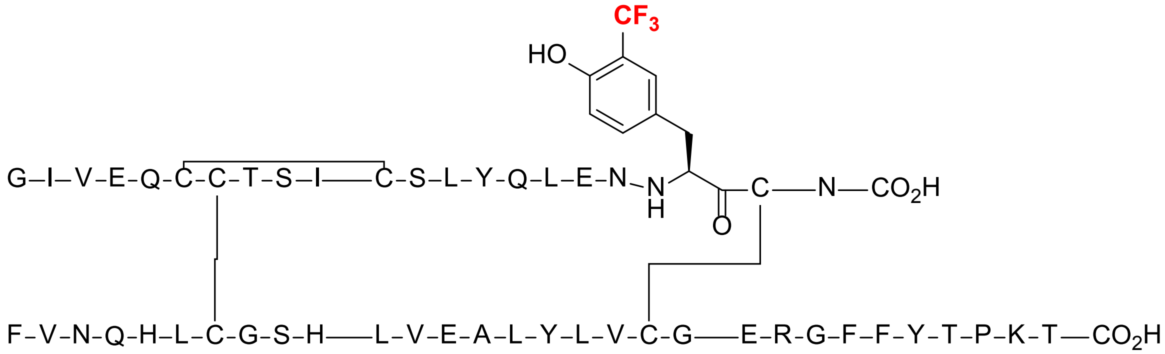

CF_3[A19]-Insulin **18**. To a 40 mL vial charged with a Teflon-coated stir bar and parent insulin (304 mg, 0.052 mmol) was added DMSO (3 mL), H_2O (0.9 mL) and AcOH (0.9 mL) and the solution was stirred gently at room temperature. Sodium trifluoromethanesulfinate (217 mg, 1.39 mmol, 27 equiv) in DMSO (final concentration 0.7 M) was added and the mixture was cooled to 0 °C under N_2. To this solution was added a 70% solution of aq. TBHP (360 µL, 2.63 mmol, 50 equiv) in a dropwise manner and stirred at 30 °C for 16 h under N_2. The crude reaction mixture was filtered through a 0.45 µm PVDF membrane. The product was diluted with 1:10 H_2O:DMF and the resulting solution was purified by a Waters mass spec-directed purification system using a Waters CSH Peptide C18 column (30 x 250 mm, 5 micron packing, 130 Å pore size) with a 30 min run time (50 mL/min flow) and a gradient of 10-45% acetonitrile in water with 0.1% TFA over 25 minutes. The fractions containing the desired products were combined and lyophilized under reduced pressure to afford 35.4 mg (11.5 %) of A19-modified CF_3[Y] insulin **18** and 6.3 mg of a mixture of CF_3 modified insulins as white solid. A procedure for HRMS/MS of these products were provided in section I of this supporting information.

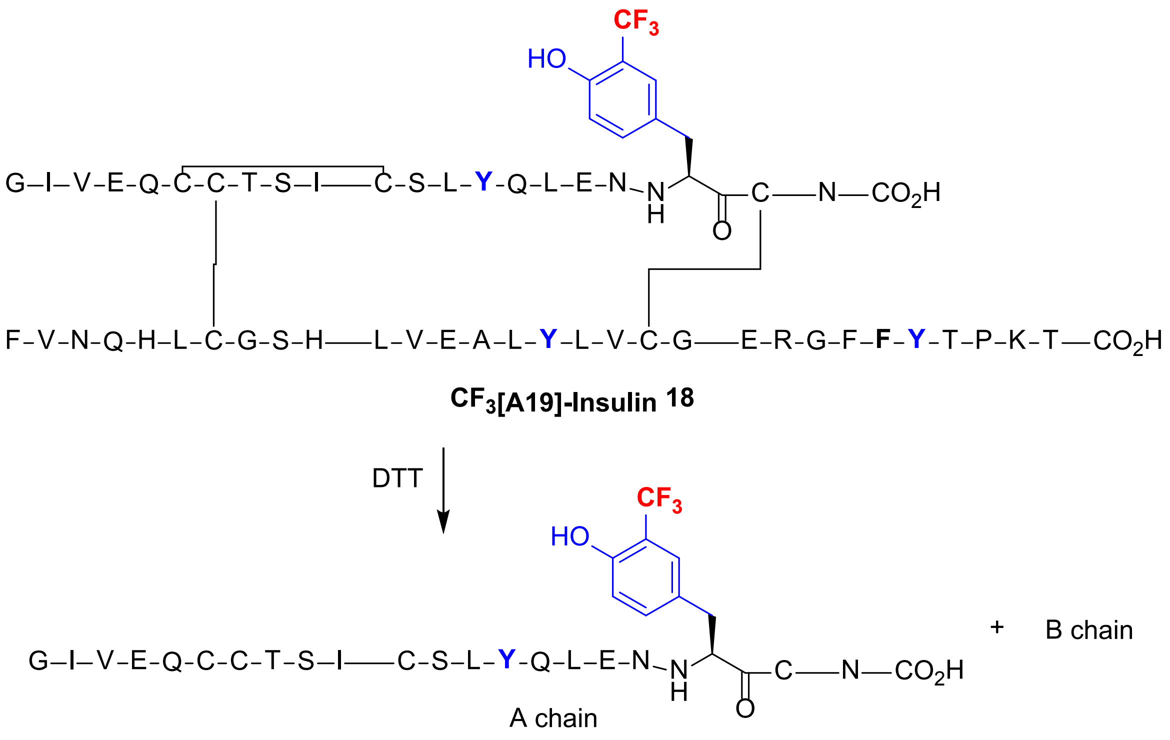

HRMS LC/MS/MS analysis of A19-modified CF_3[Y] insulin major using Bruker FT-ICR:

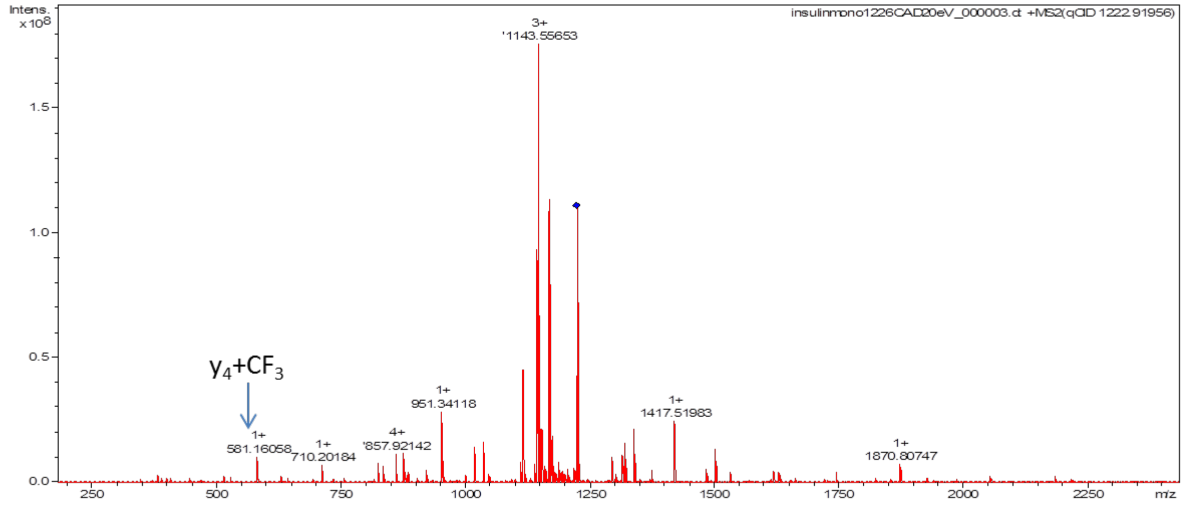

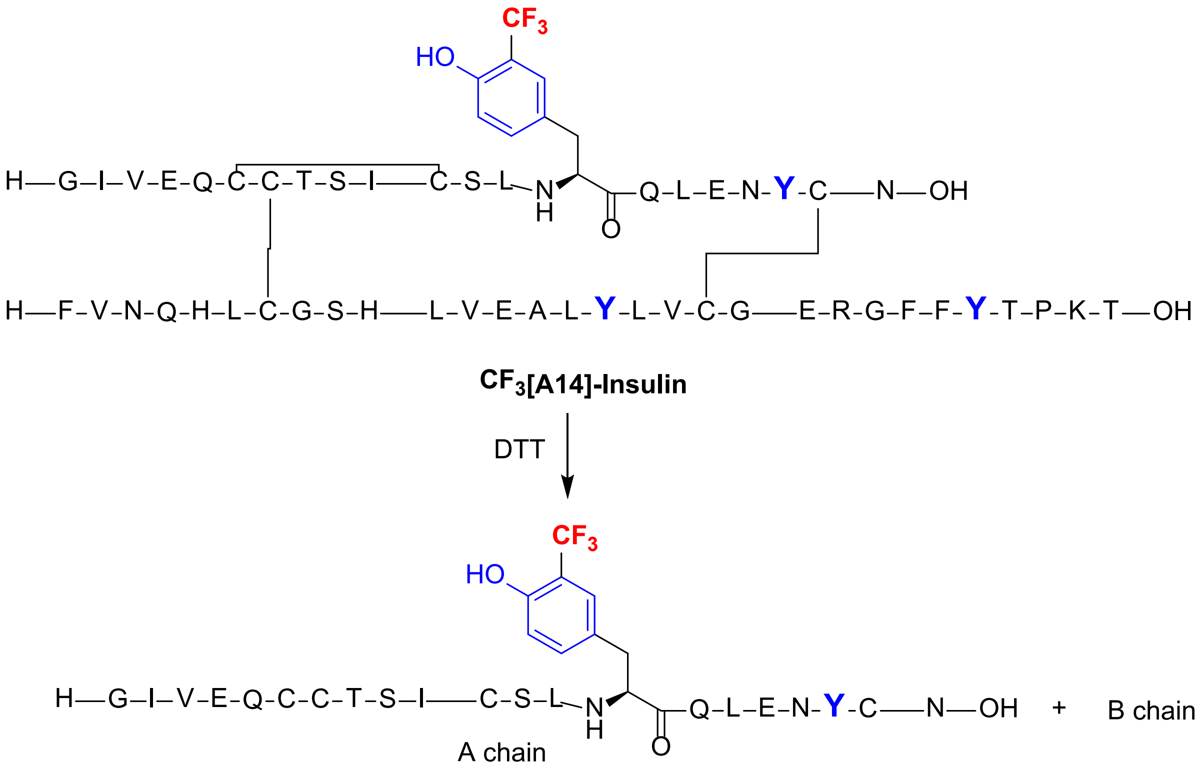

HRMS LC/MS/MS analysis of A14-modified CF_3[Y] insulin (minor) using Bruker FT-ICR:

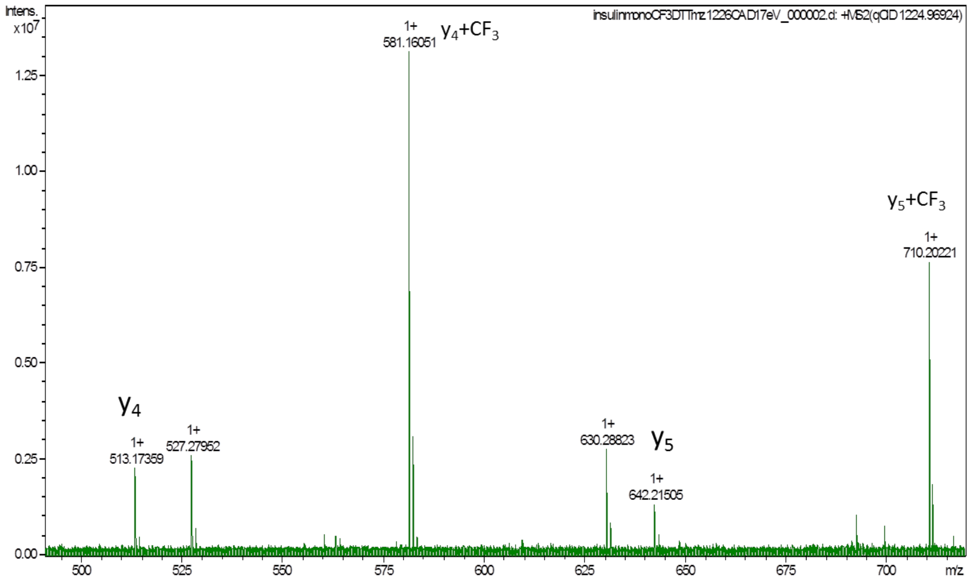

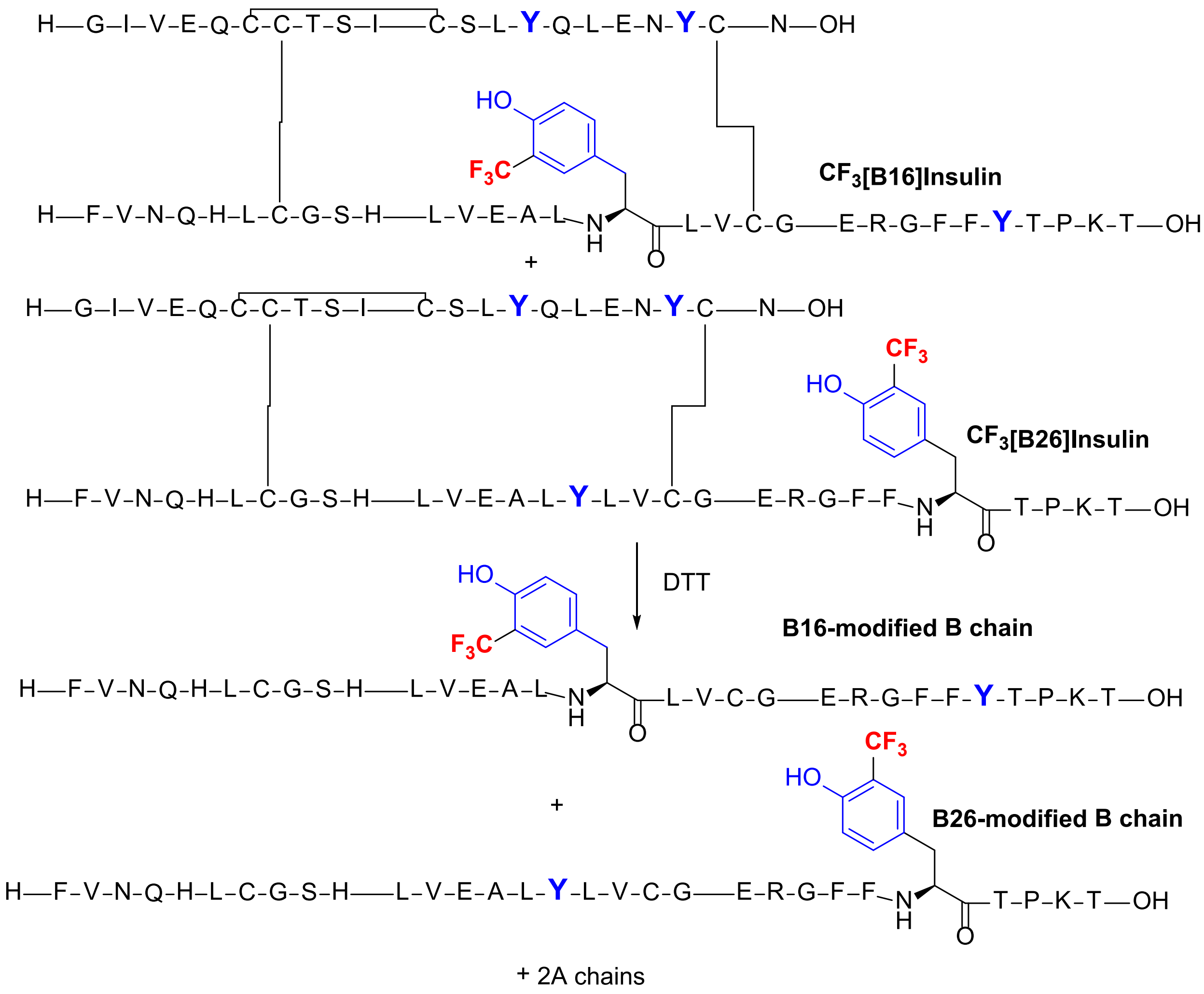

HRMS LC/MS/MS analysis of a mixture of B16 and B26-modified CF_3[Y] insulin:

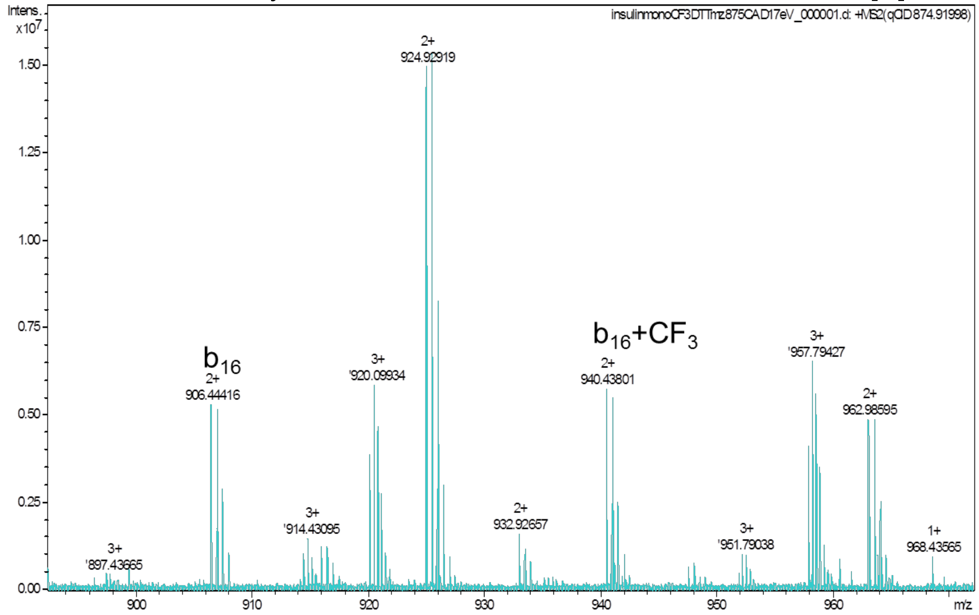

c. Synthesis of azido-containing cyclic peptides

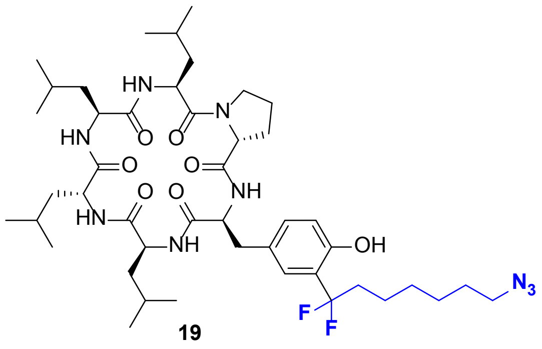

19

Compound 19: To a 4 mL vial charged with a Teflon-coated stir bar, compound **17** (81 mg, 0.113 mmol) and sodium difluoroheptylazidosulfinate (89 mg, 0.339 mmol, 3 equiv) were added. The vial was placed under N_2 and a solution of acetic acid (32 µL), water (291 µL) and DMSO (808 µL) was added. To this solution was added a 70% solution of aq. TBHP (88 µL, 0.536 mmol, 5 equiv) in a dropwise manner and the reaction was stirred at 45 ^{o}C for 24 h. The reaction mixture was lyophilized and reconstituted into 1 mL of 1:1 DMSO:MeOH. The resulting solution was purified by preparative HPLC using a Waters SunFire C18 preparative column (19 x 100mm) with a 20 min run time (30 mL/min flow) and a gradient of 50-100% acetonitrile in water with 0.16% TFA. The fractions containing the desired product were combined and lyophilized under reduced pressure to afford 7.5 mg (4.4%) of **19** as a white solid (92% purity) isolated as the TFA salt.

^{1}H-NMR (600 MHz, acetonitrile-d_3) δ 7.90 – 7.71 (b, 1H), 7.37 (b, 1H), 7.30 (d, 2.0 Hz, 1H), 7.18 (dd, *J* = 8.1, 2.1 Hz, 1H), 7.01 (b, 1H), 6.89 (d, 8.3 Hz, 1H), 4.78 (d, *J* = 7.1 Hz, 1H), 4.39 (b, 4H), 4.11 (b, 1H), 3.69 (b, 1H), 3.58 (b, 1H), 3.33 -3.24 (m, 2H), 3.23 – 3.19 (m, 1H), 3.01 – 2.86 (m, 1H), 2.34 (b, 2H), 2.3-1.98 (m, 2H), 1.87 (b, 1H), 1.75– 1.47 (m, 14H), 1.35 (d, *J* = 6.3, 5.6 Hz, 6H), 1.40-1.30 (b, 6H), 1.0-0.84 (m, 24H); ^{13}C-NMR (150 MHz, acetonitrile-d_3) δ 173.35, 171.40, 172.14, 170.72, 160.95, 152.94, 137.16, 131.60, 128.54, 127.14, 60.44, 60.00, 55.48, 54.35, 53.1, 51.51, 50.81, 48.91, 48.06, 47.52, 41.03, 39.90, 38.01, 36.25, 28.39, 28.34, 28.29, 26.22, 26.09, 24.75, 24.68, 24.46, 24.39, 24.36, 23.90, 22.43, 22.26, 21.90, 21.54, 21.40, 21.01, 20.38, 13.52. ^{19}F-NMR (471 MHz, acetonitrile-d_3) δ -93.97 (d, *J* = 79.3 Hz). HRMS (ESI) ([M+H]+) calculated for $C_{45}H_{72}F_2N_9O_7^+$ 888.5517, found 888.5533.

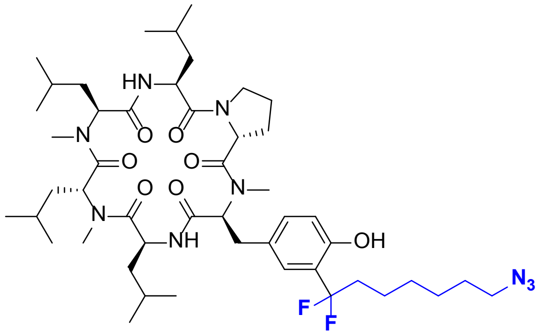

Compound 20. To a 4 mL vial charged with a Teflon-coated stir bar, compound **16** (81 mg, 0.107 mmol), $ZnCl_2$ (21.9 mg, 0.161 mmol, 1.5 equiv) and sodium difluoroheptylazidosulfinate (85 mg, 0.321 mmol, 3 equiv) were added. The vial was placed under N_2 and a solution of acetic acid (100 µL) and water (900 µL) was added. To this solution was added a 70% solution of aq. TBHP (88 µL, 0.536 mmol, 5 equiv) in a dropwise manner and stirred at 45 ^{o}C for 24 h. The reaction mixture was lyophilized and reconstituted into 1 mL of 1:1 DMSO:MeOH. The resulting solution was purified by preparative HPLC using a Waters SunFire C18 preparative column (19 x 100mm) with a 20 min run time (30 mL/min flow) and a gradient of 50%-100% acetonitrile in water with 0.16% TFA. The fractions containing the desired product were combined and lyophilized under reduced pressure to afford 7.5 mg (6.2%) of **20** as white solid (96% purity) as the TFA salt.

^{1}H-NMR (600 MHz, methylene chloride-d_2) δ 7.49 (d, J = 9.6 Hz, 1H), 7.13 (s, 1H), 7.12 (d, J = 8.6 Hz), 6.80 (d, J = 8.4 Hz, 2H), 5.62 (dd, J = 12.0, 4.6 Hz, 1H), 5.19 (dd, J = 9.1, 3.0 Hz, 1H), 5.04 (dt, J = 9.3, 7.2 Hz, 1H), 4.96 (dd, J = 9.7, 5.7 Hz, 1H), 4.84 (q, J = 7.6 Hz, 1H), 4.39 (t, J = 6.8 Hz, 1H), 3.76 (q, J = 8.6, 7.9 Hz, 1H), 3.56 (q, J = 9.6, 7.6 Hz, 1H), 3.51 (dd, J = 15.4, 4.6 Hz, 1H), 3.25 (t, J = 7.0 Hz, 2H), 3.17 (s, 3H), 3.07 (s, 3H), 2.94 (s, 3H), 2.80 (dd, J = 15.4, 12.0 Hz, 1H), 2.24 (tt, J = 17.3, 7.8 Hz, 2H), 2.14 – 2.06 (m, 1H), 1.97-1.77 (m, 9H) 1.68 – 1.47 (m, 8H), 1.46 – 1.40 (m, 2H), 1.39 – 1.33 (m, 4H), 1.02 (d, J = 6.6 Hz, 3H), 0.99 – 0.92 (m, 18H), 0.85 (d, J = 6.6 Hz, 3H). ^{13}C-NMR data is unavailable due to the low isolated quantity of **20.** ^{19}F-NMR (471 MHz, acetonitrile-d_3) δ -93.79 (d, J = 70.7 Hz). HRMS (ESI) $[M+H]^+$ calculated for $C_{48}H_{78}F_2N_9O_7^+$ 930.5987, found 930.5995.

XI. Insulin Assays

Insulin Receptor Binding Assays were run in a scintillation proximity assay (SPA) 384-well format using cell membranes prepared from CHO cells overexpressing human insulin receptor(B) grown in F12 media containing 10% FBS and antibiotics (G418, Penicillin/Streptavidin). Cell membranes were prepared in 50 mM Tris buffer, pH 7.8, containing 5 mM $MgCl_2$. The assay buffer contained 50 mM Tris buffer, pH 7.5, 150 mM NaCl, 1 mM $CaCl_2$, 5 mM $MgCl_2$, 0.1% BSA and protease inhibitors (Complete-Mini-Roche). Cell membranes were added to WGA PVT PEI SPA beads (5 mg/mL final concentration) followed by addition of test molecules at appropriate concentrations. After 5-15 min incubation at room temperature, [^{125}I]-insulin was added at 0.015 nM final concentration for a final total volume of 50 μL. The mixture was incubated with shaking at room temperature for 1 to 12 hours followed by scintillation counting to determine [^{125}I]-insulin binding to IR and the titration effects of test molecules on this interaction.

Insulin Receptor Phosphorylation Assays were performed as follows: The insulin receptor phosphorylation assays were performed using the commercially available Meso Scale Discovery (MSD) pIR assay (Meso Scale Discovery, 9238 Gaithers Road, Gaitherburg, MD). CHO cells stably expressing human insulin receptor(B) were grown in F12 cell media containing 10% FBS and antibiotics (G418, Penicillin/Streptavidin) for at least 8 hours and then serum starved by switching to F12 media containing 0.5% BSA (insulin-free) in place of FBS for overnight growth. Cells were harvested and frozen in aliquots for use in the MSD pIR assay. Briefly, the frozen cells were plated in 96-well (40,000 cells/well) clear tissue culture plates and allowed to recover. Test molecules at the appropriate concentrations were added and the cells were incubated for 8 min at 37 °C. The media was aspirated and chilled, and MSD cell lysis buffer was added as per MSD kit instructions. The cells were lysed on ice for 40 min and the lysate then mixed for 10 minutes at room temperature. The lysate was transferred to the MSD kit pIR detection plates. The remainder of the assay was carried out following the MSD kit recommended protocol.

XII. References

(1) Le, M. K. Wismer, Z- C. Shi, R. Zhang, D. V. Conway, G. Li, P. Vachal, I.W. Davies, and D. W. C. MacMillan *ACS Cent. Sci.* **2017** *3* (6), 647.

(2) Brueckl, Y. J. T.; Baxter, R. D.; Fujiwara, Y.; Seiple, I. B.; Su, S.; Blackmond, D. G.; Baran, P. S. *Proc. Nat. Acad. Sci,* 2011, **108**, 14411.

(3) (a) Prasad, D.; König, B. *Chem. Commun.* **2014**, *50*, 6688. (b) DiRocco, D. A.; Rovis, T. *J. Am. Chem. Soc.* **2012**, *134*, 8094. (c) Cai, S.; Zhao, X.; Wang, X.; Liu, Q.; Li, Z.; Wang, D. Z. *Angew. Chem. Int. Ed.* **2012**, *51,* 8050.

XIII. Spectroscopic Data for Characterized Compounds

^{1}H NMR (600 MHz, Methanol-d_4)

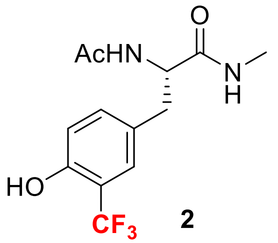

2

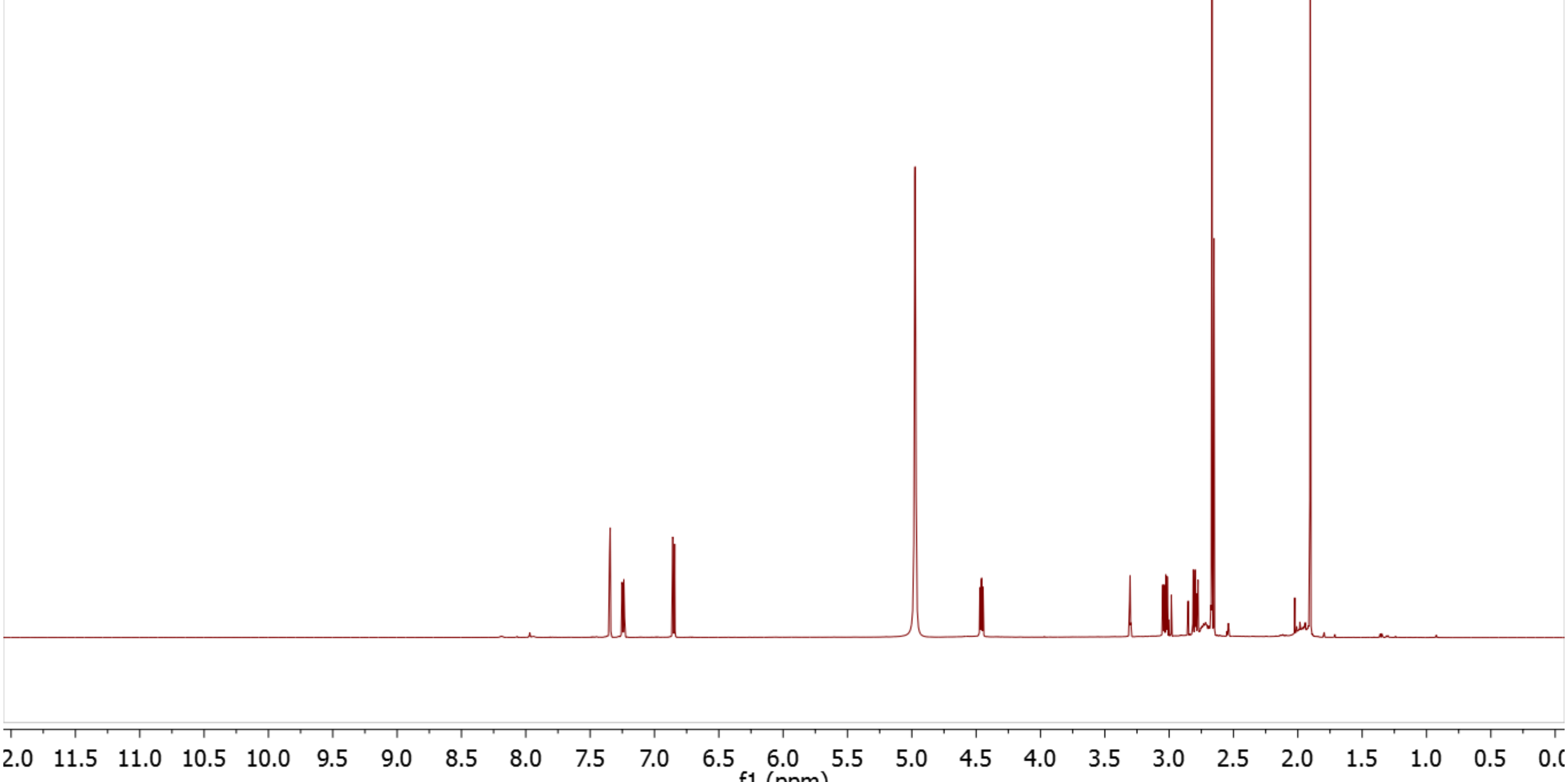

^{13}C NMR (150 MHz, Methanol-d_4)

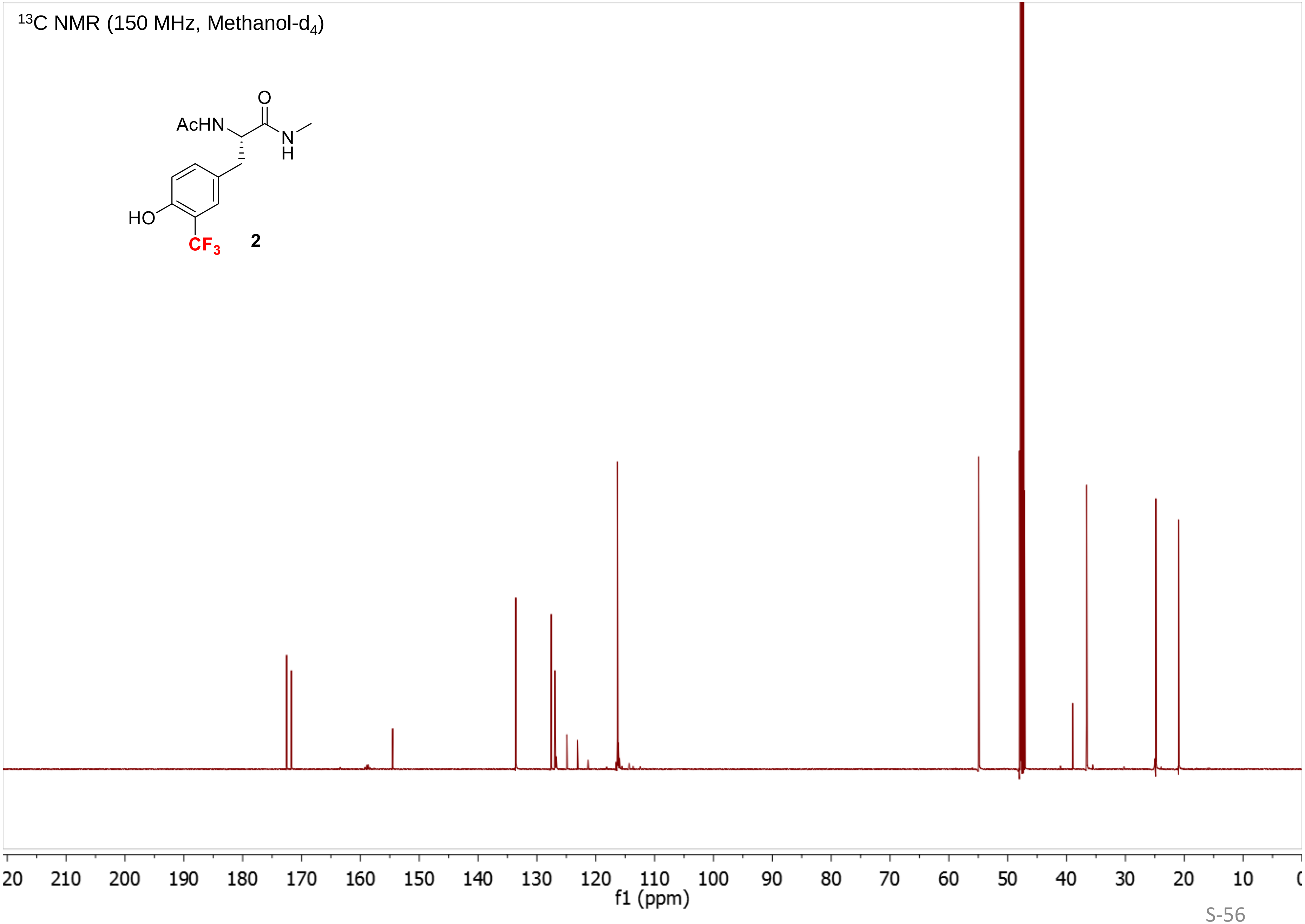

^{19}F NMR (564 MHz, Methanol-d_4)

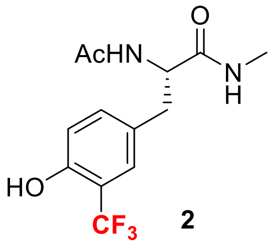

2

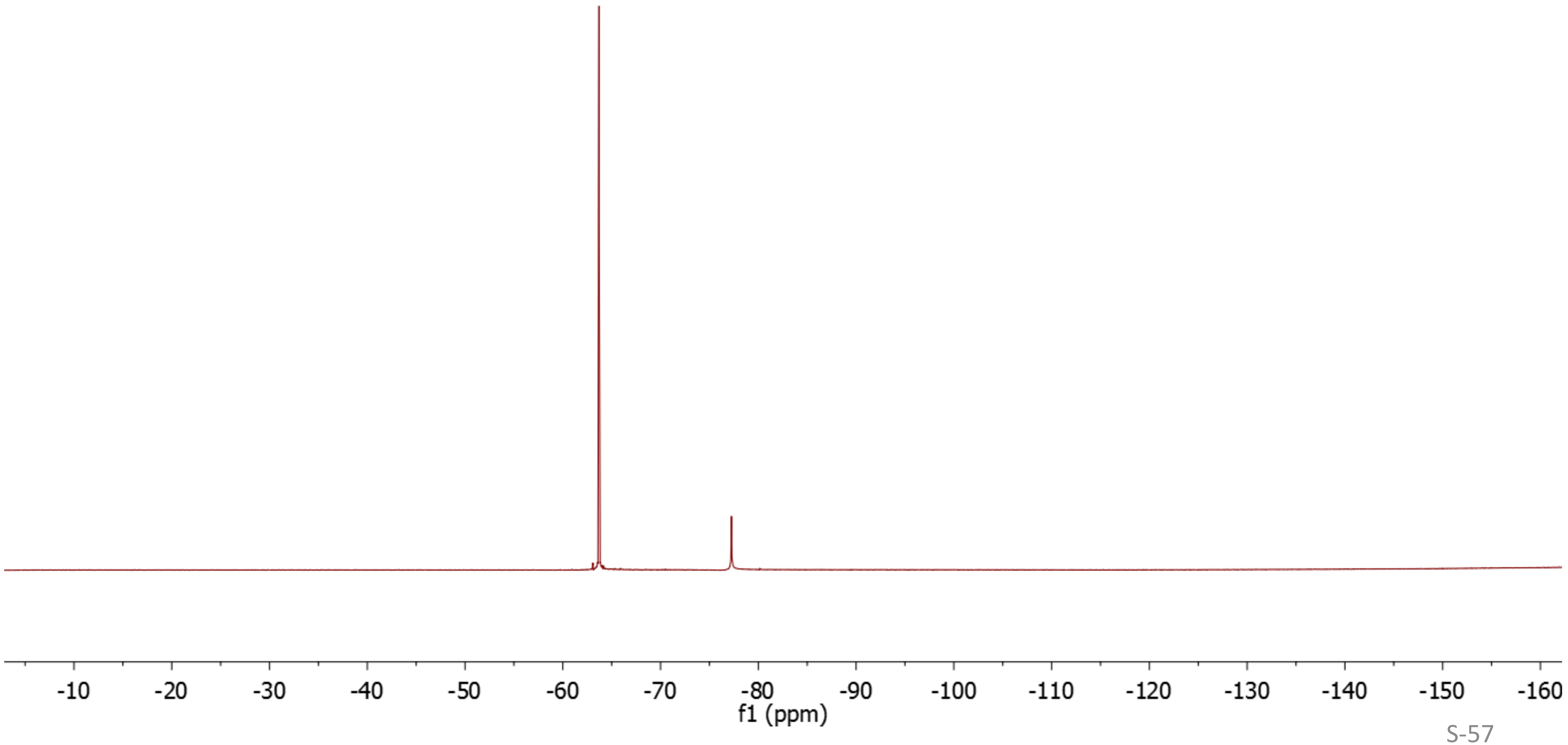

^{1}H NMR (600 MHz, Methanol-d_4)

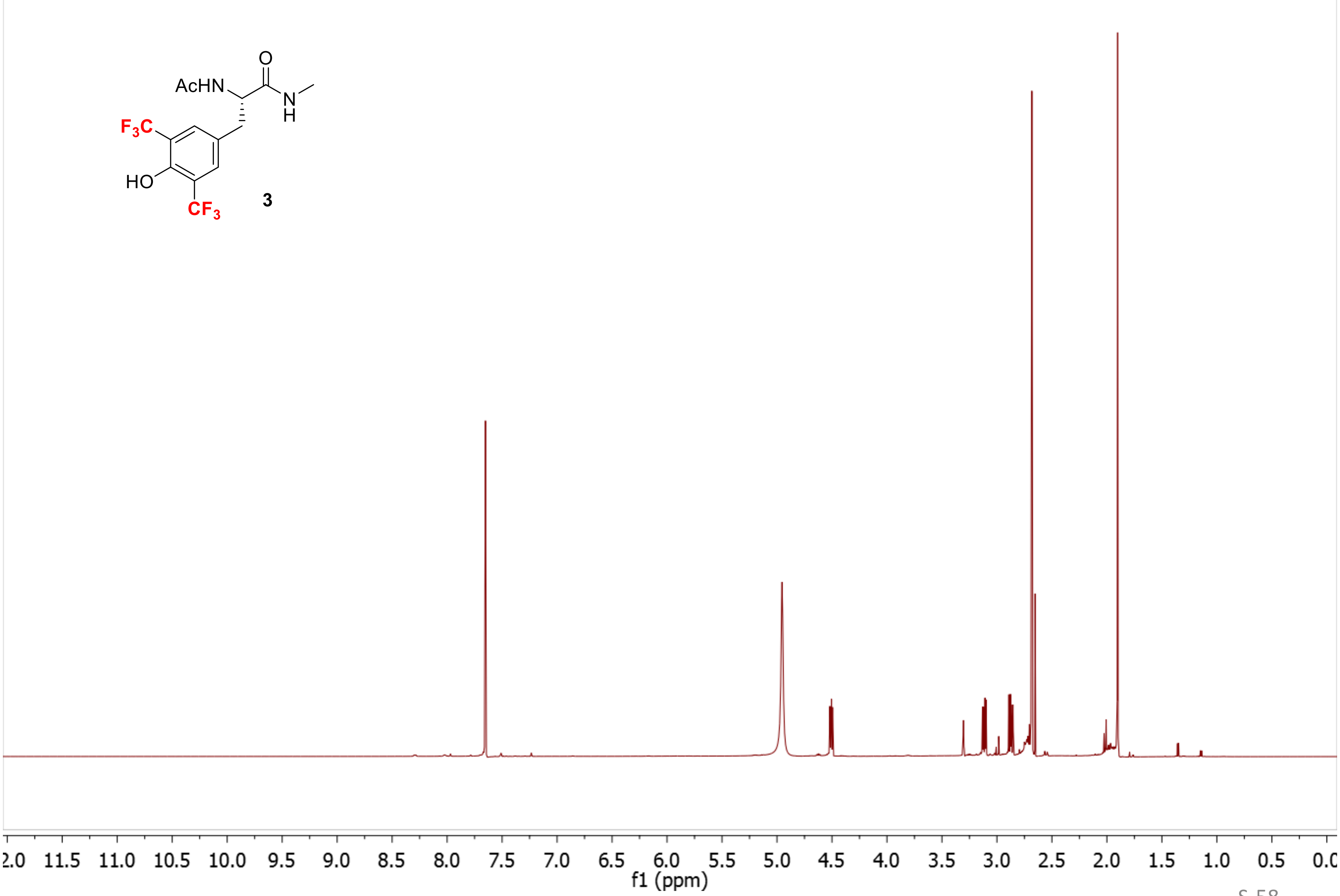

^{13}C NMR (150 MHz, Methanol-d_4)

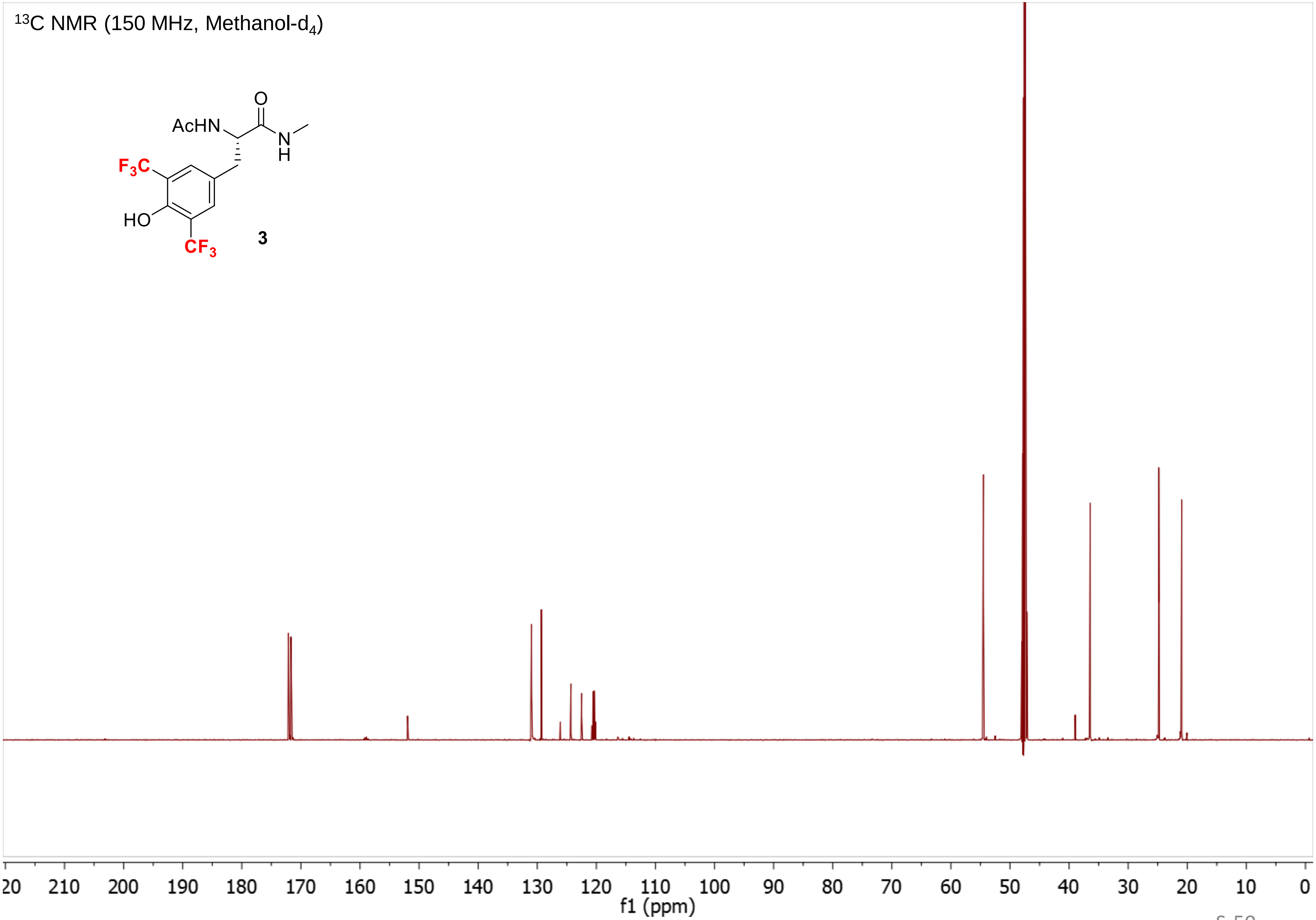

^{19}F NMR (564 MHz, Methanol-d_4)

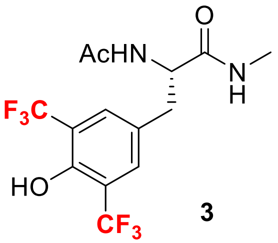

3

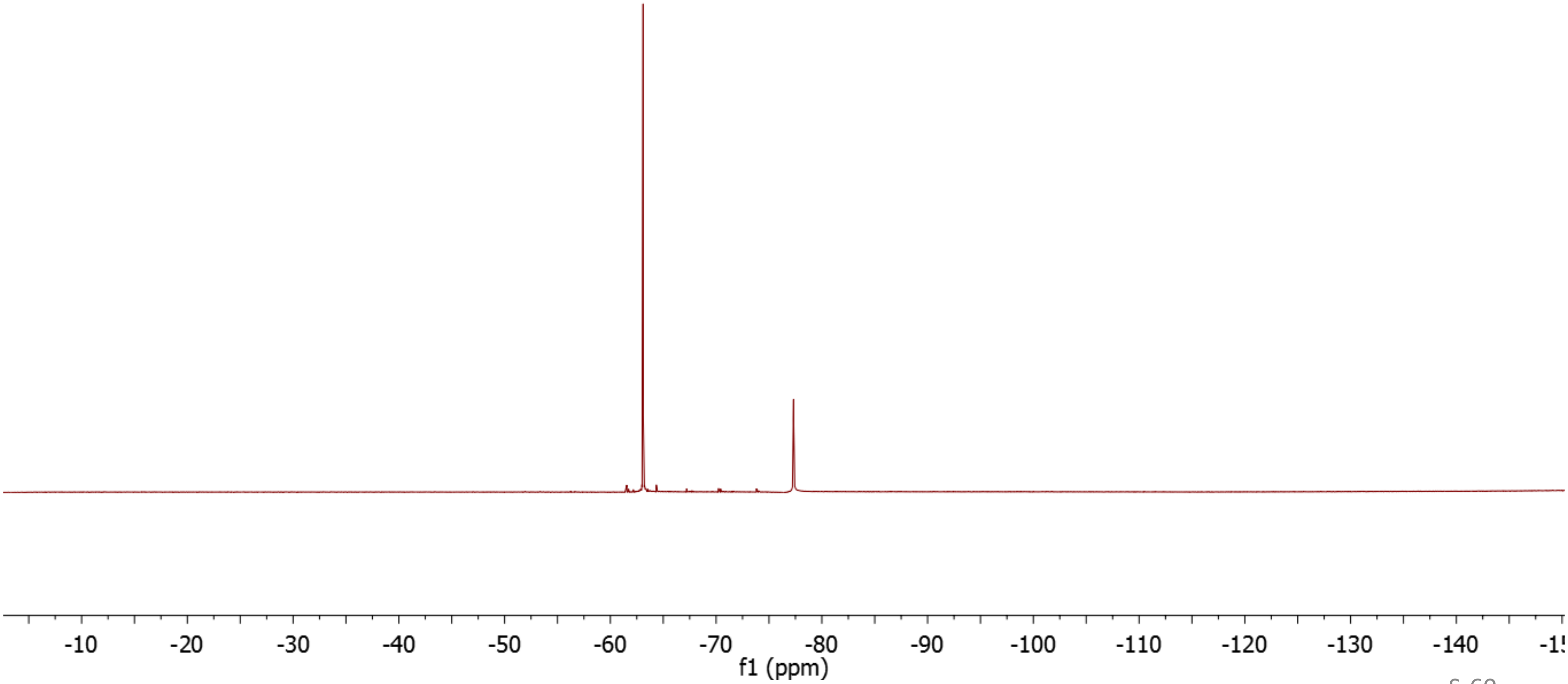

^{1}H NMR (500 MHz, Methanol-d_4)

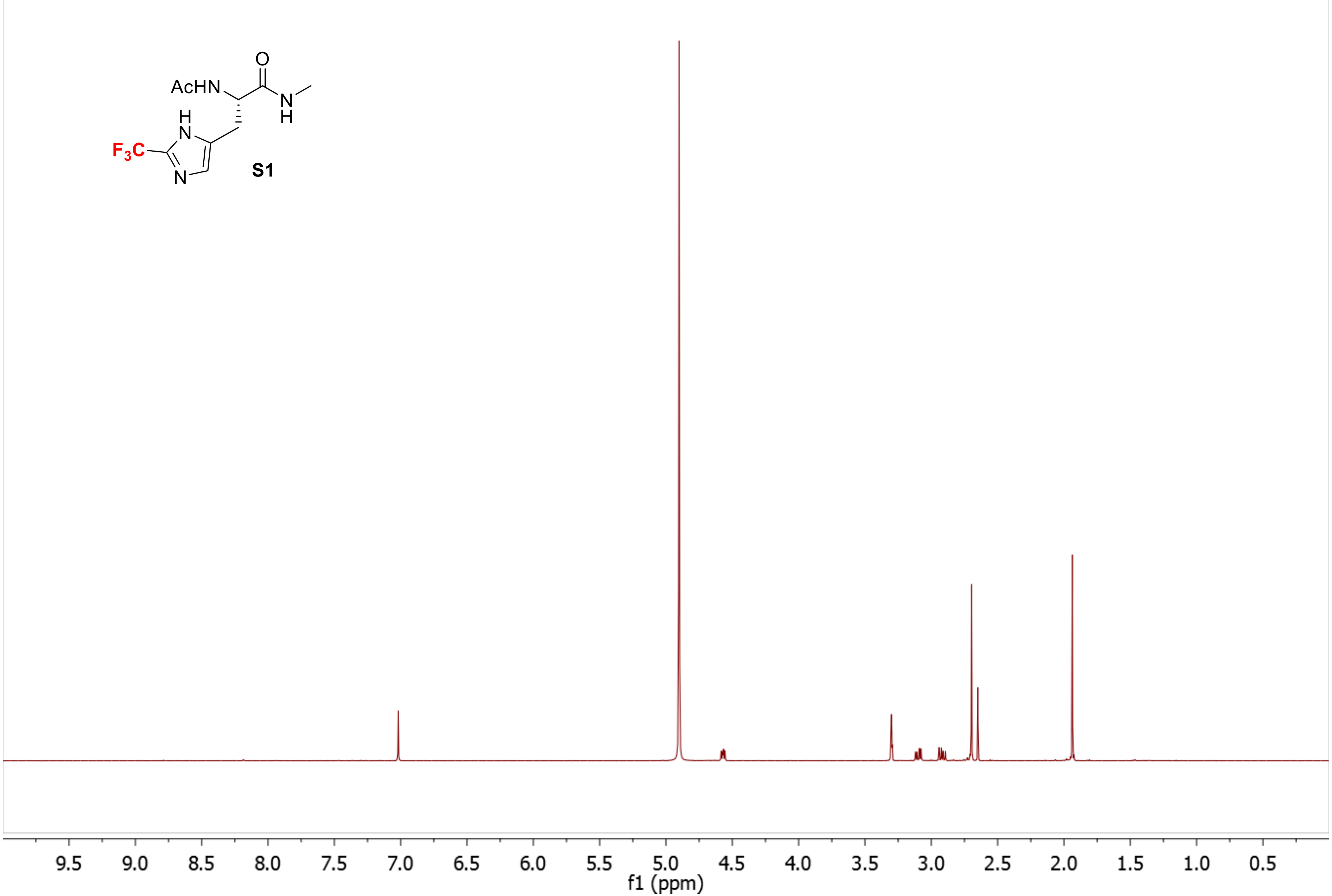

^{13}C NMR (150 MHz, Methanol-d_4)

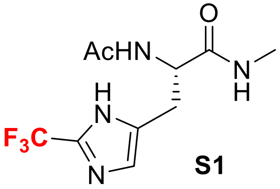

S1

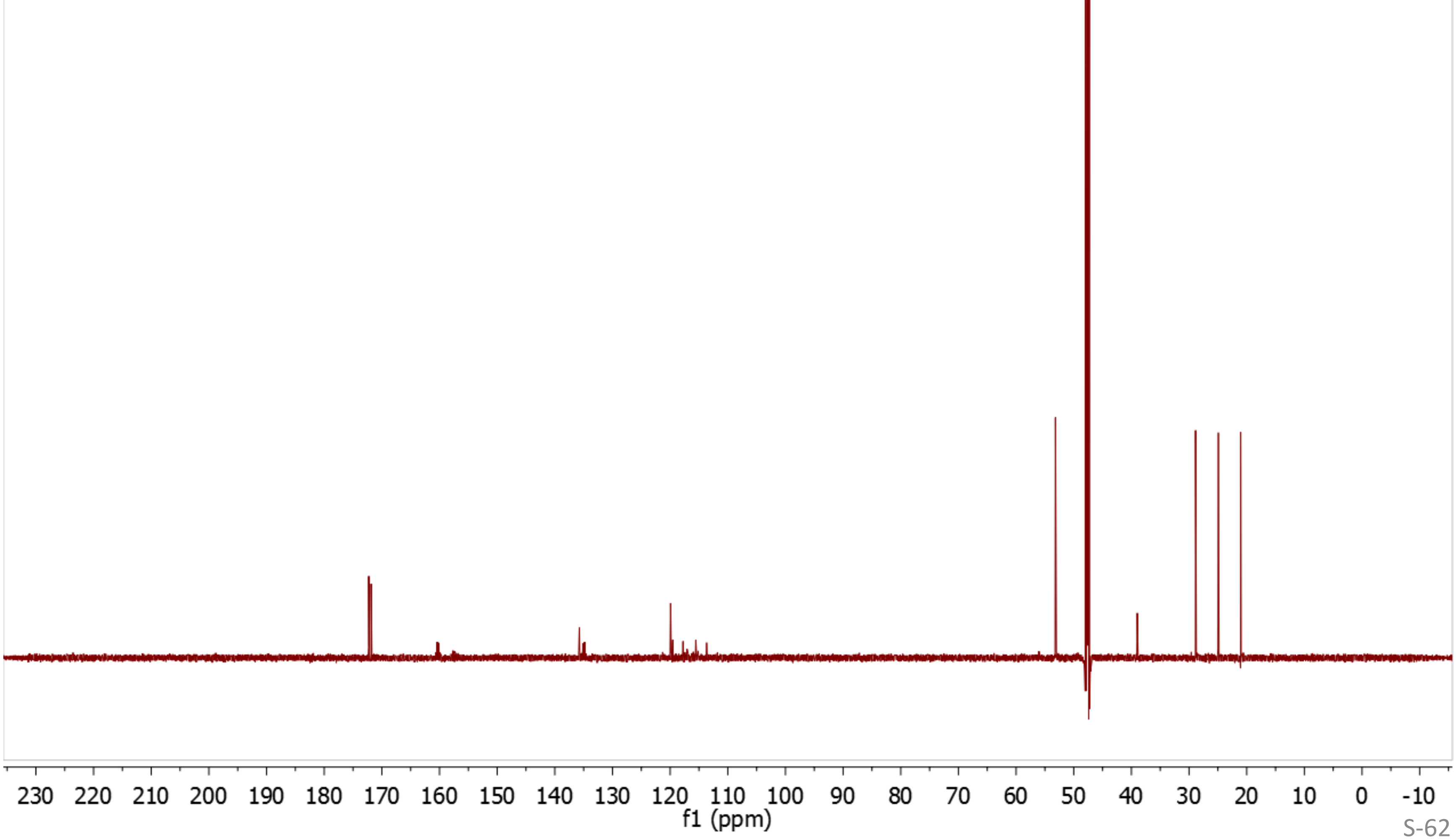

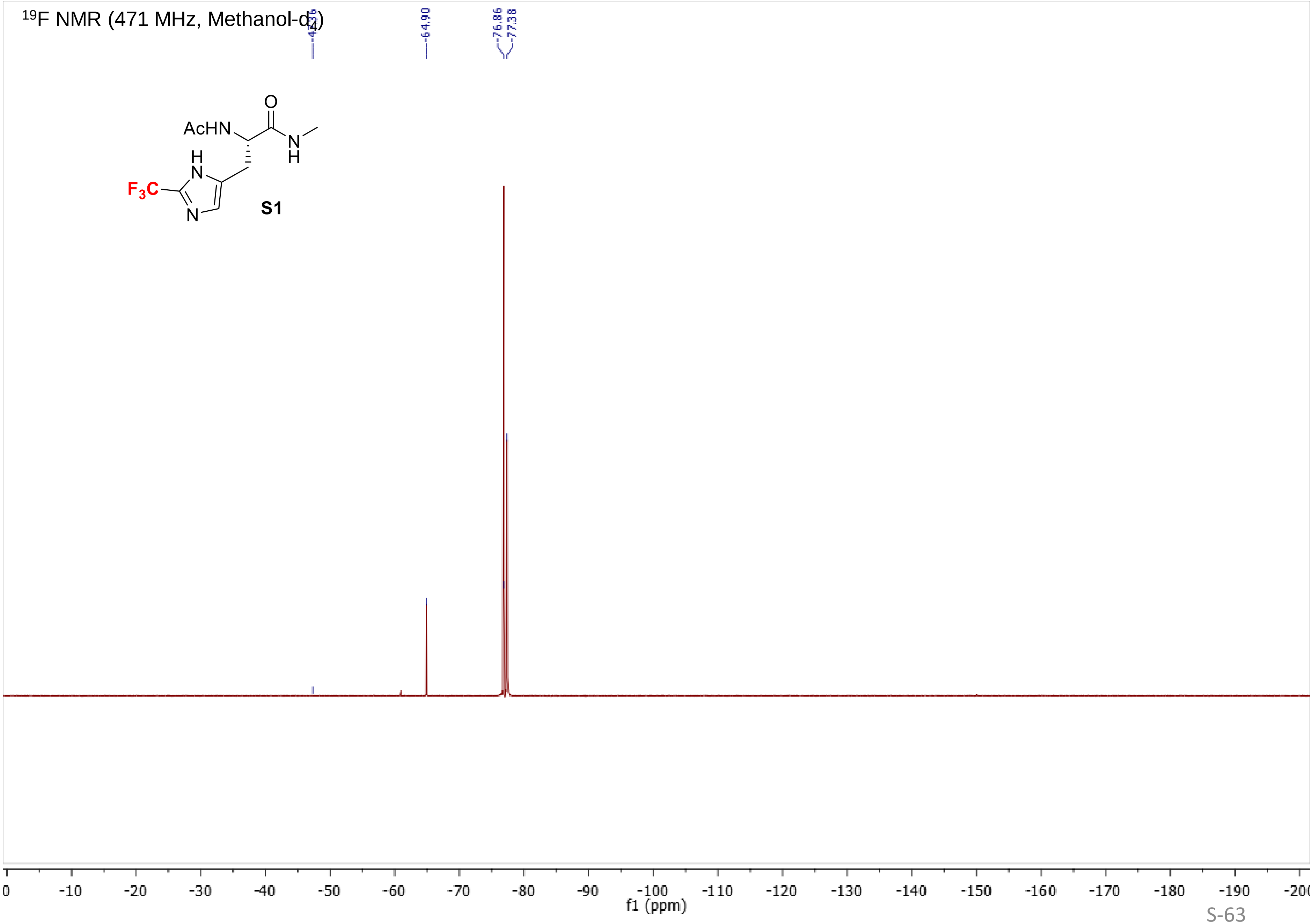
19F NMR (471 MHz, Methanol-d4)
-64.90
-76.86
-77.38
AcHN
O
N
H
H
N
F3C
N
S1
0
-10
-20
-30
-40
-50
-60
-70
-80
-90
-100
-110
-120
-130
-140
-150
-160
-170
-180
-190
f1 (ppm)

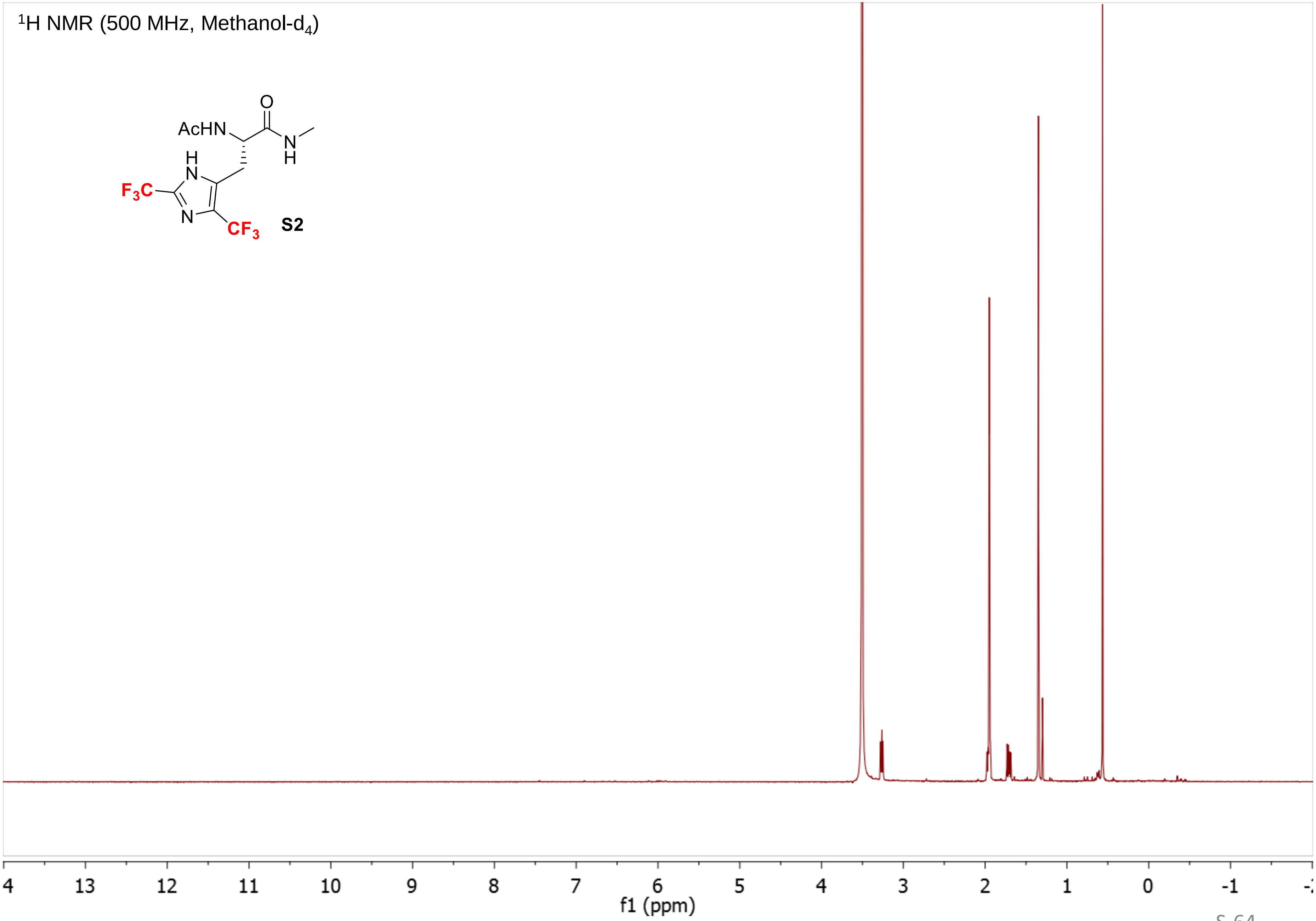

1H NMR (500 MHz, Methanol-d4)
AcHN
O
N
H
H
N
N
F3C
CF3
S2
4
13
12
11
10
9
8
7
6
5
4
3
2
1
0
-1
f1 (ppm)

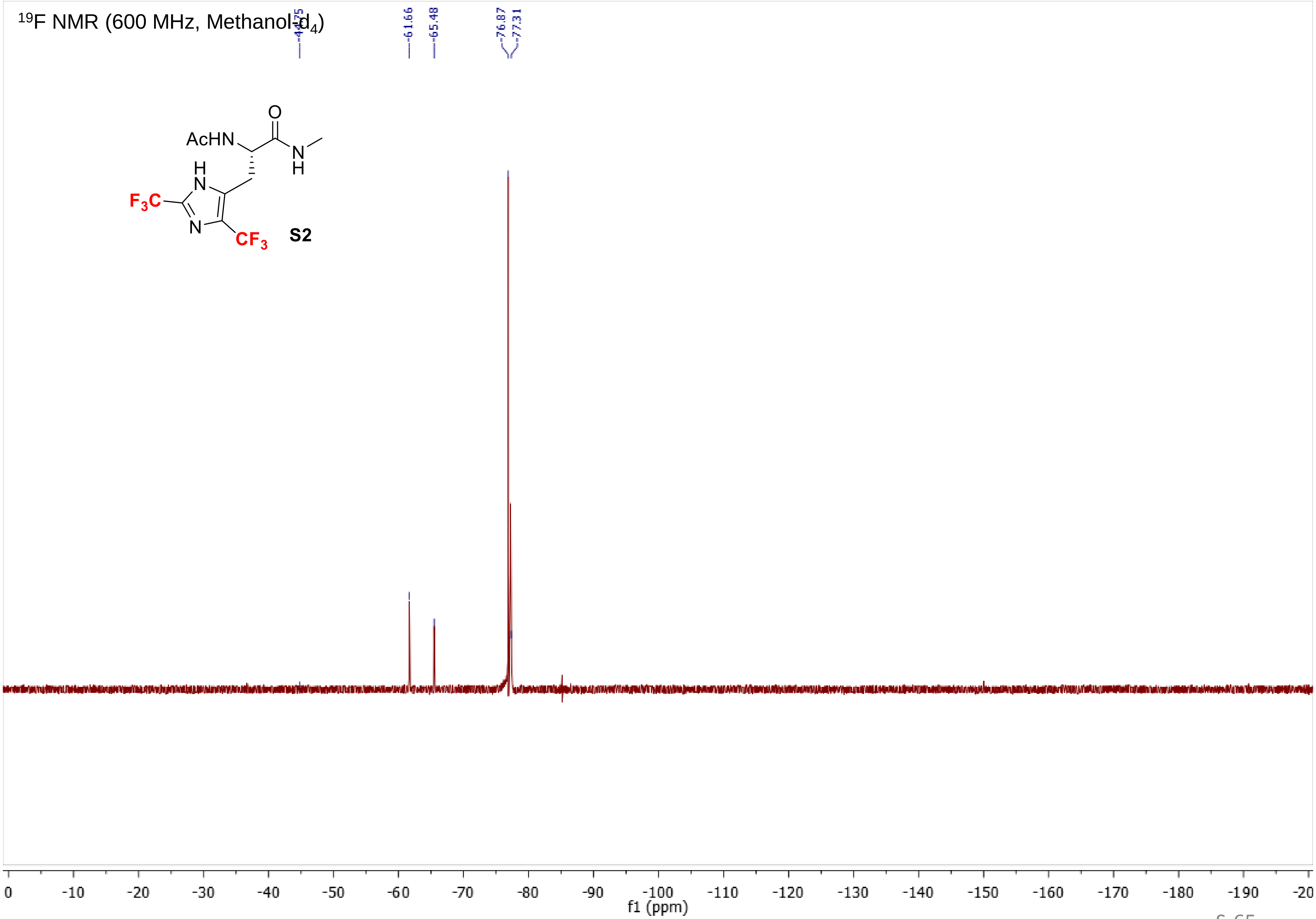

19F NMR (600 MHz, Methanol-d4)
-44.75
-61.66
-65.48
-76.87
-77.31
AcHN
O
N
H
F3C
CF3
S2
0
-10
-20
-30
-40
-50
-60
-70
-80
-90
-100
-110
-120
-130
-140
-150
-160
-170
-180
-190
-20
f1 (ppm)

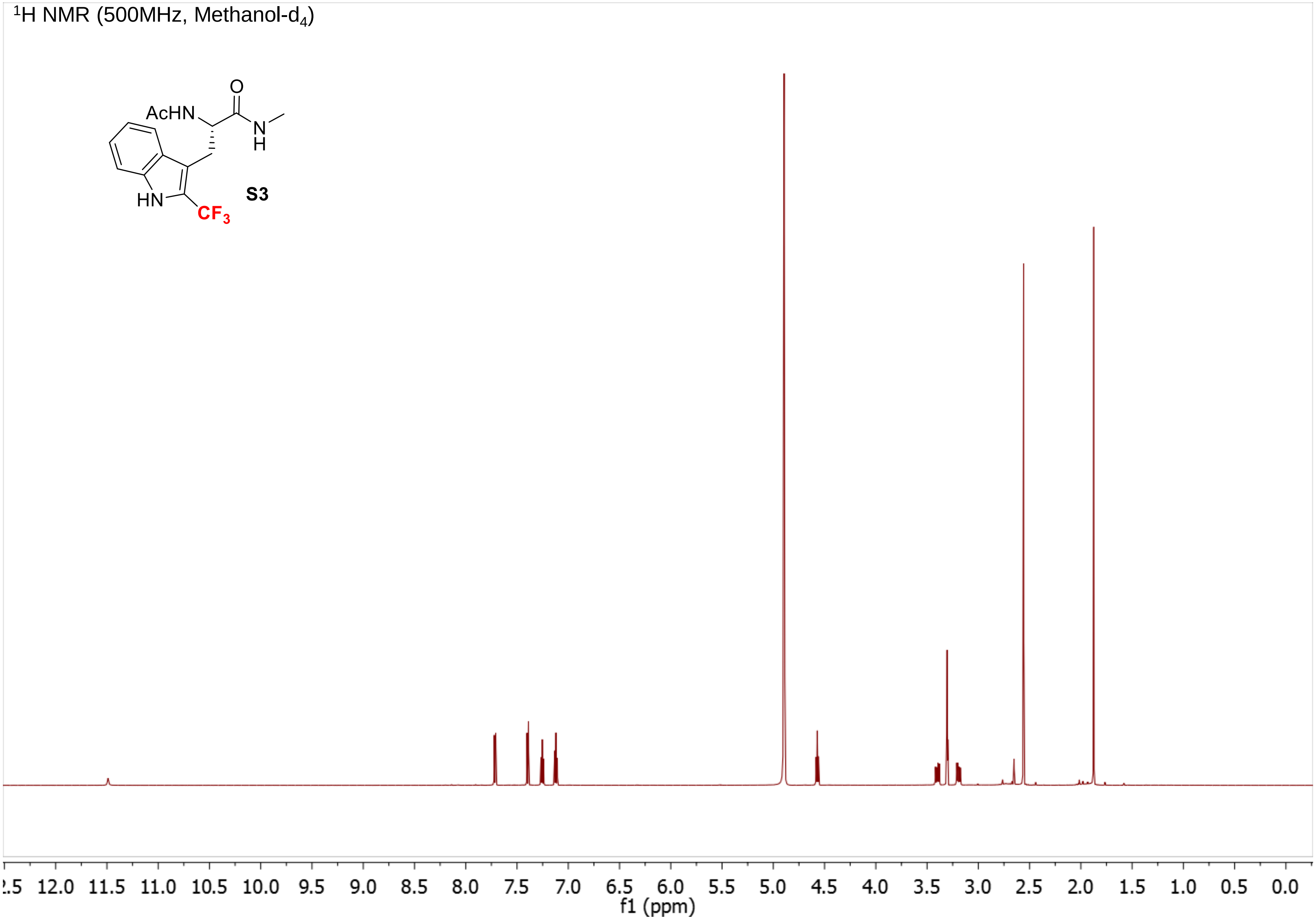

1H NMR (500MHz, Methanol-d4)
AcHN
O
N
H
HN
CF3
S3
.5 12.0 11.5 11.0 10.5 10.0 9.5 9.0 8.5 8.0 7.5 7.0 6.5 6.0 5.5 5.0 4.5 4.0 3.5 3.0 2.5 2.0 1.5 1.0 0.5 0.0
f1 (ppm)

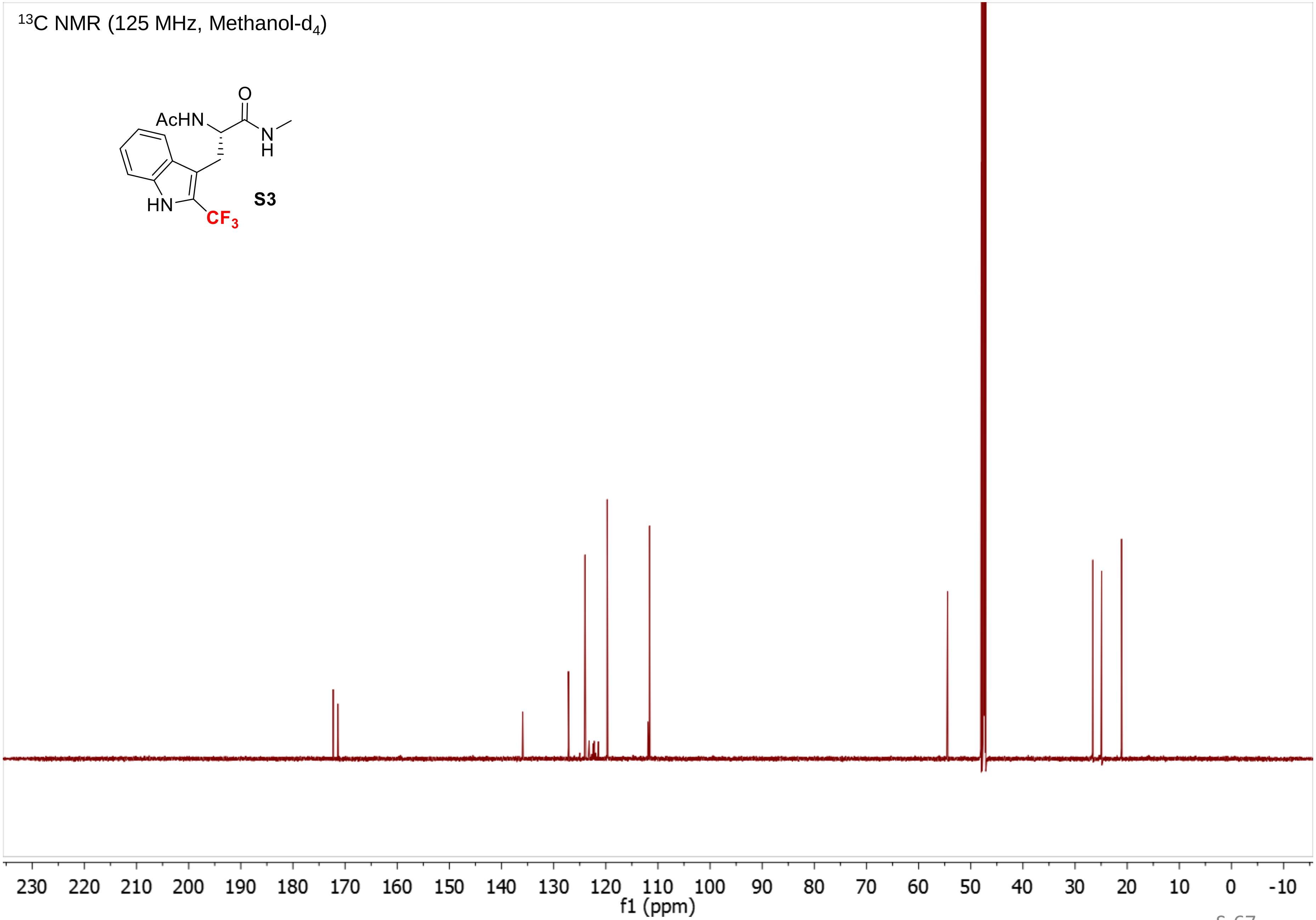

^{13}C NMR (125 MHz, Methanol-d$_4$)
AcHN
O
N
H
HN
CF$_3$
S3
230 220 210 200 190 180 170 160 150 140 130 120 110 100 90 80 70 60 50 40 30 20 10 0 -10
f1 (ppm)

^{19}F NMR (471 MHz, Methanol-d_4)

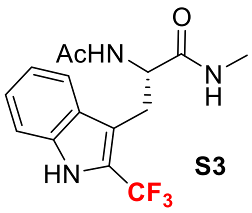

S3

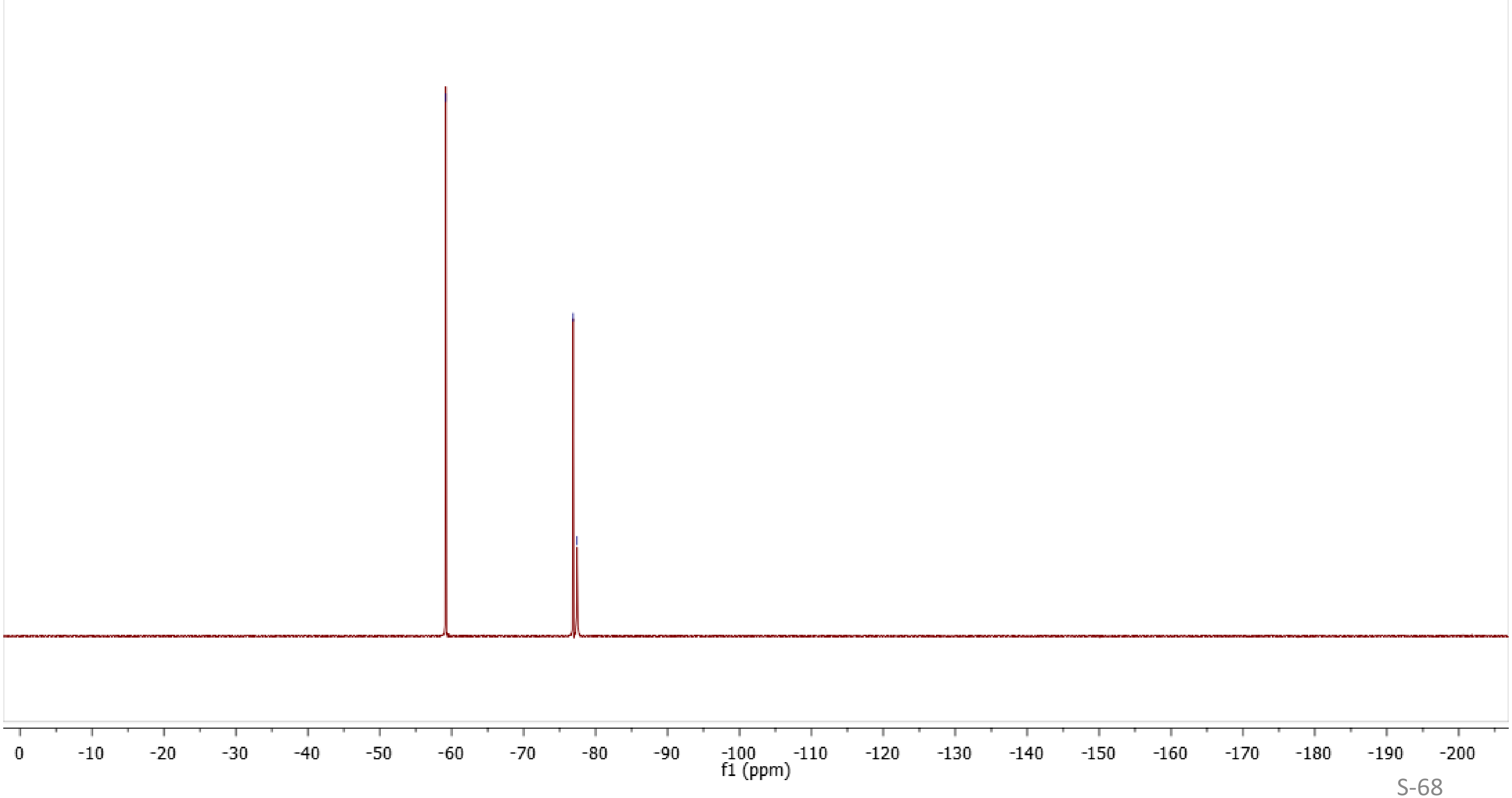

^{1}H NMR (500 MHz, Methanol-d_4)

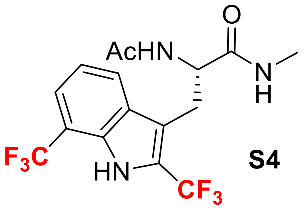

S4

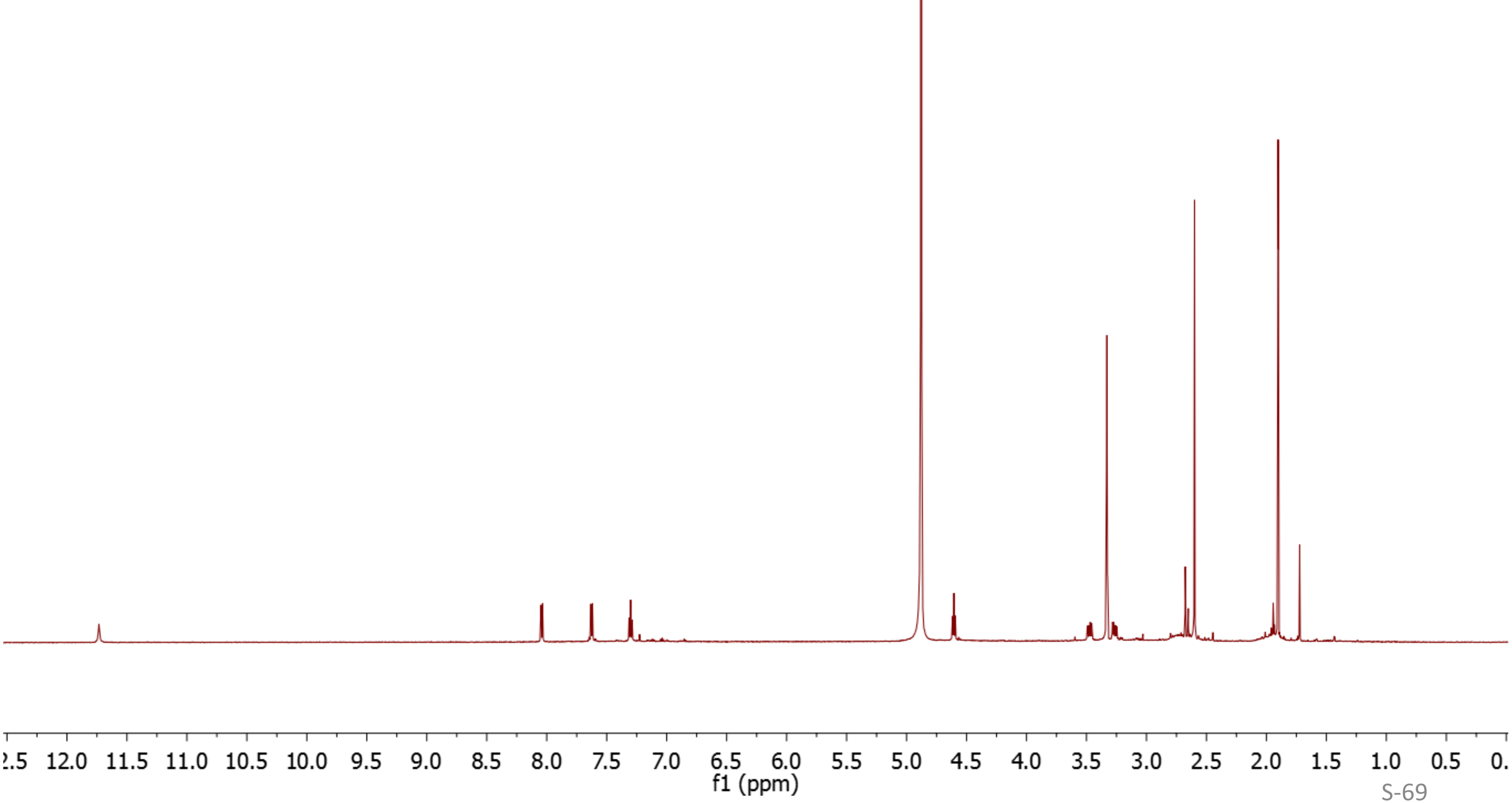

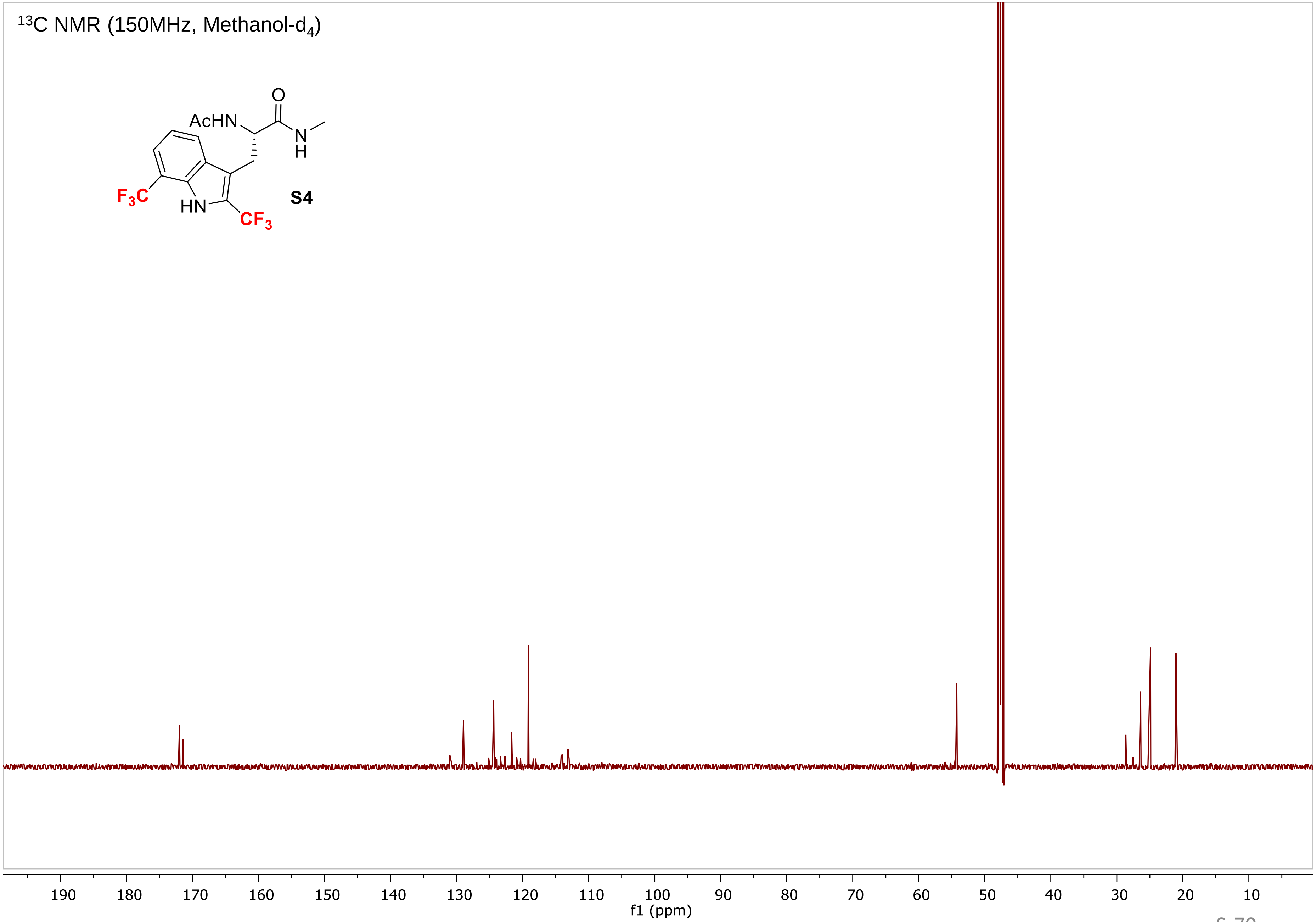
13C NMR (150MHz, Methanol-d4)
AcHN
O
N
H
F3C
HN
CF3
S4
190
180
170
160
150
140
130
120
110
100
90
80
70
60
50
40
30
20
10
f1 (ppm)

^{19}F NMR (470MHz, Methanol-d_4)

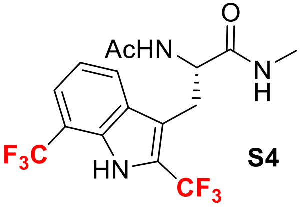

S4

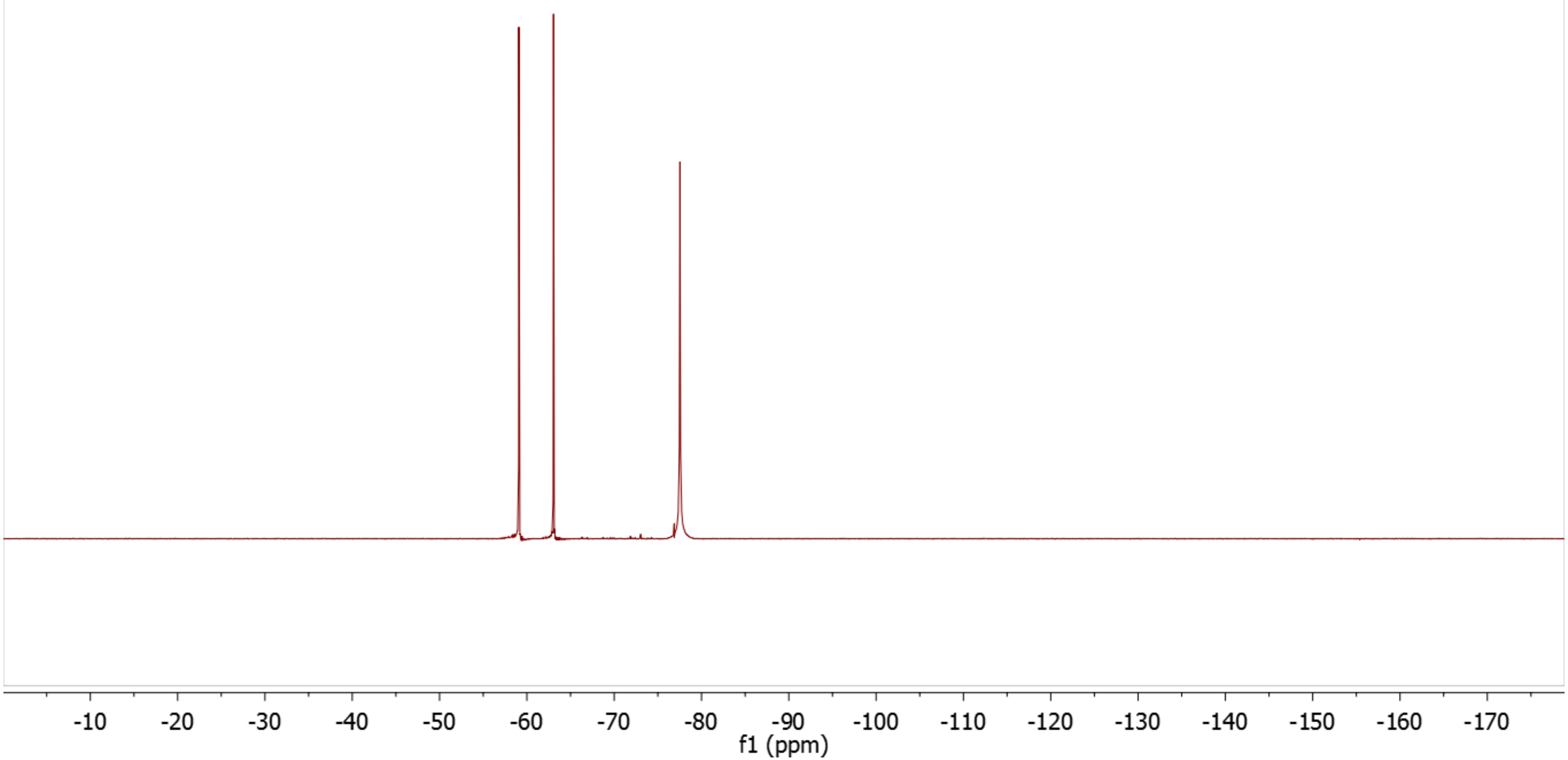

^{1}H NMR (500 MHz, Methanol-d_4)

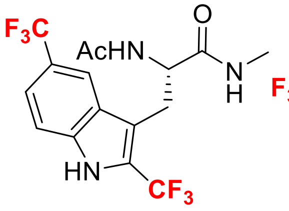

S5

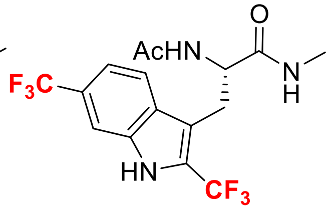

S6

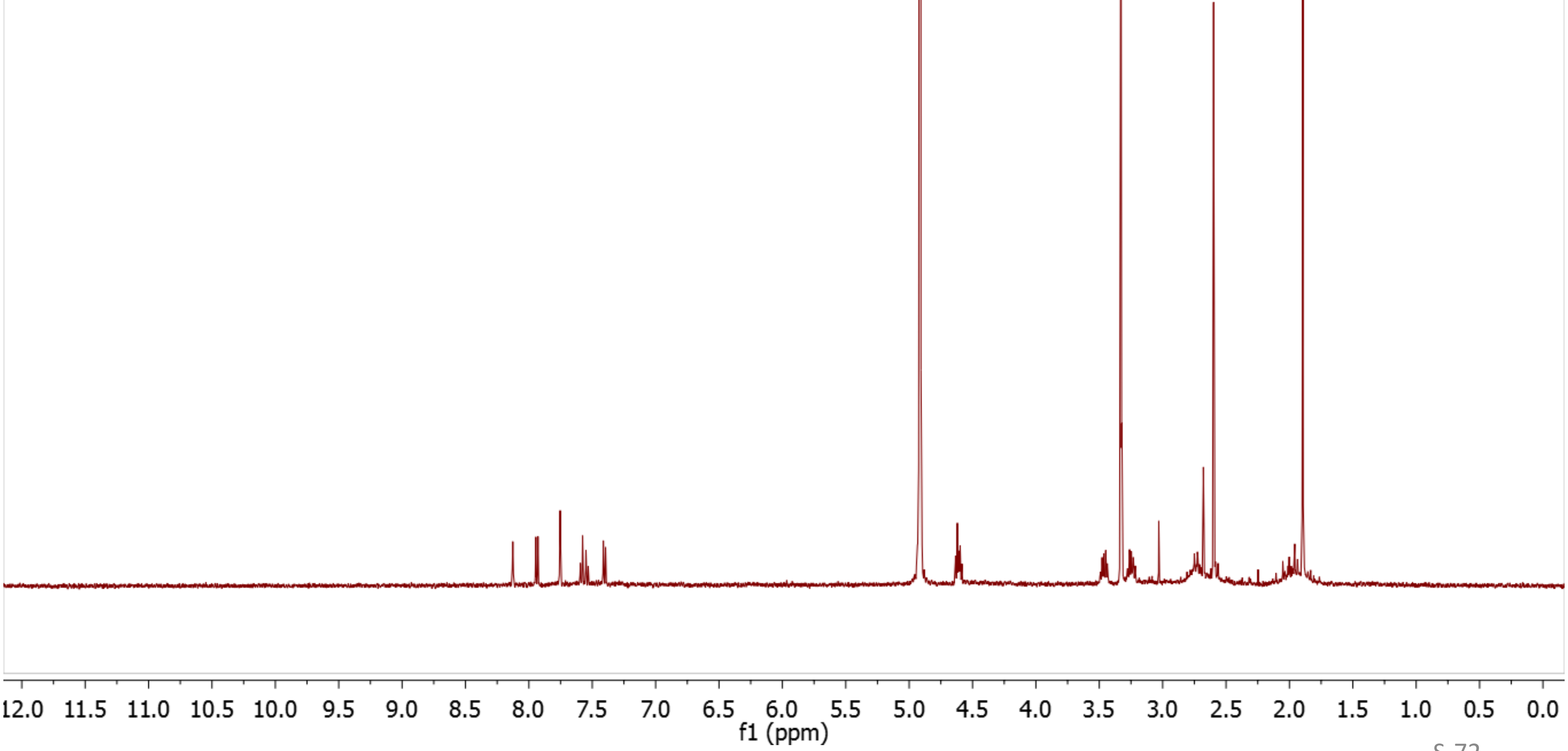

COSY of **S5** and **S6**

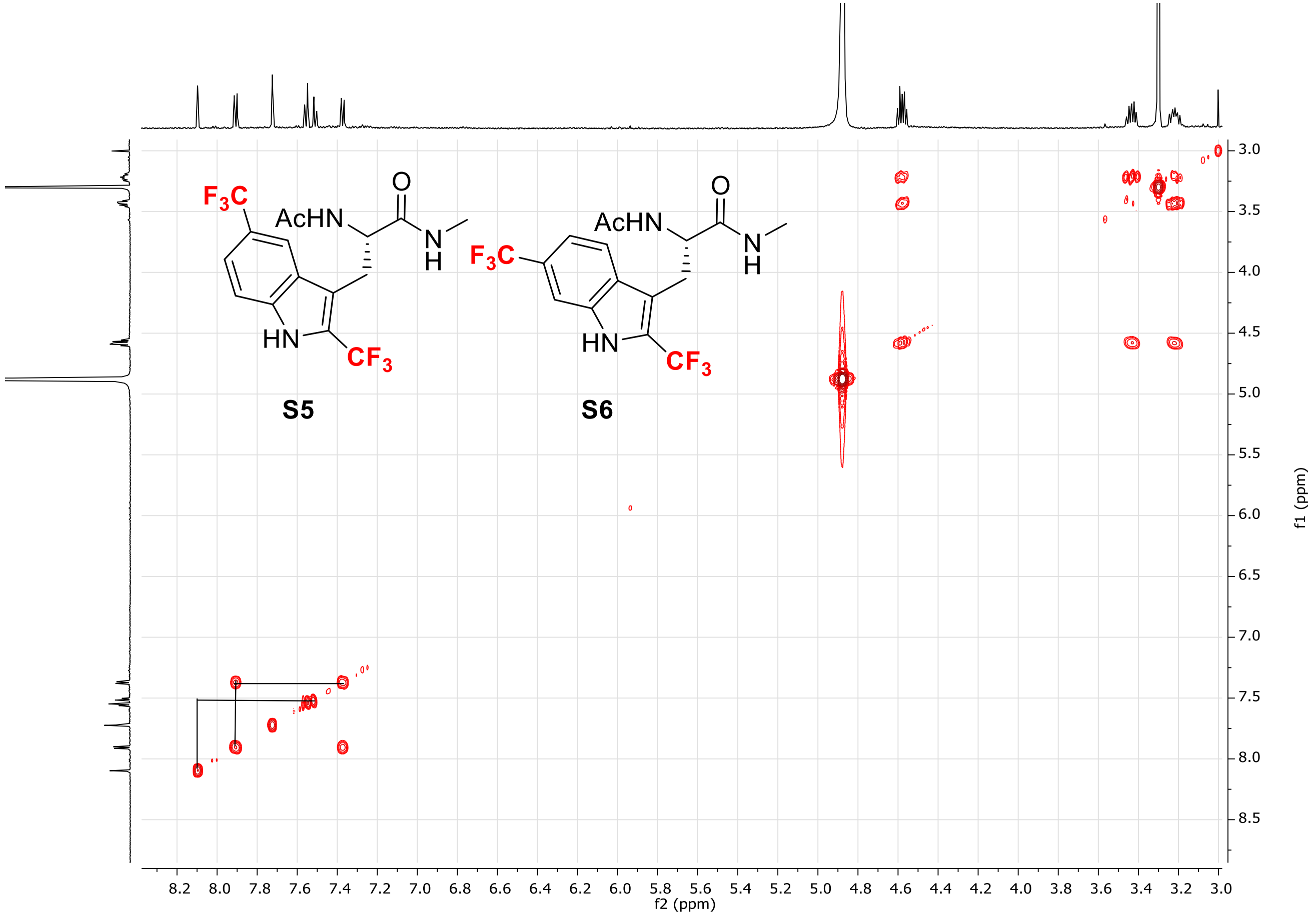

^{19}F NMR (471 MHz, Methanol-d$_4$)

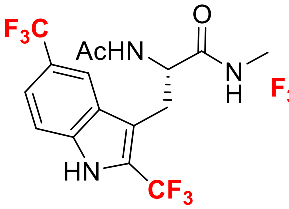

S5

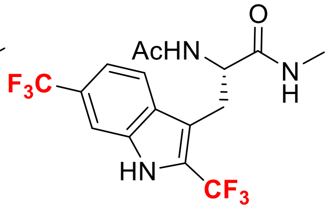

S6

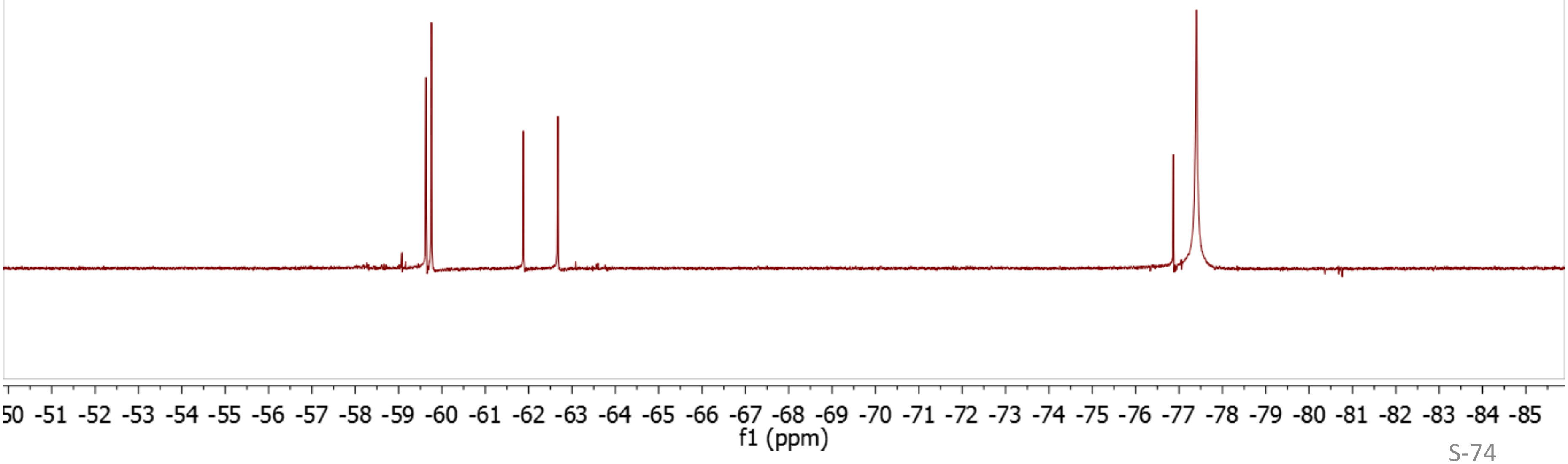

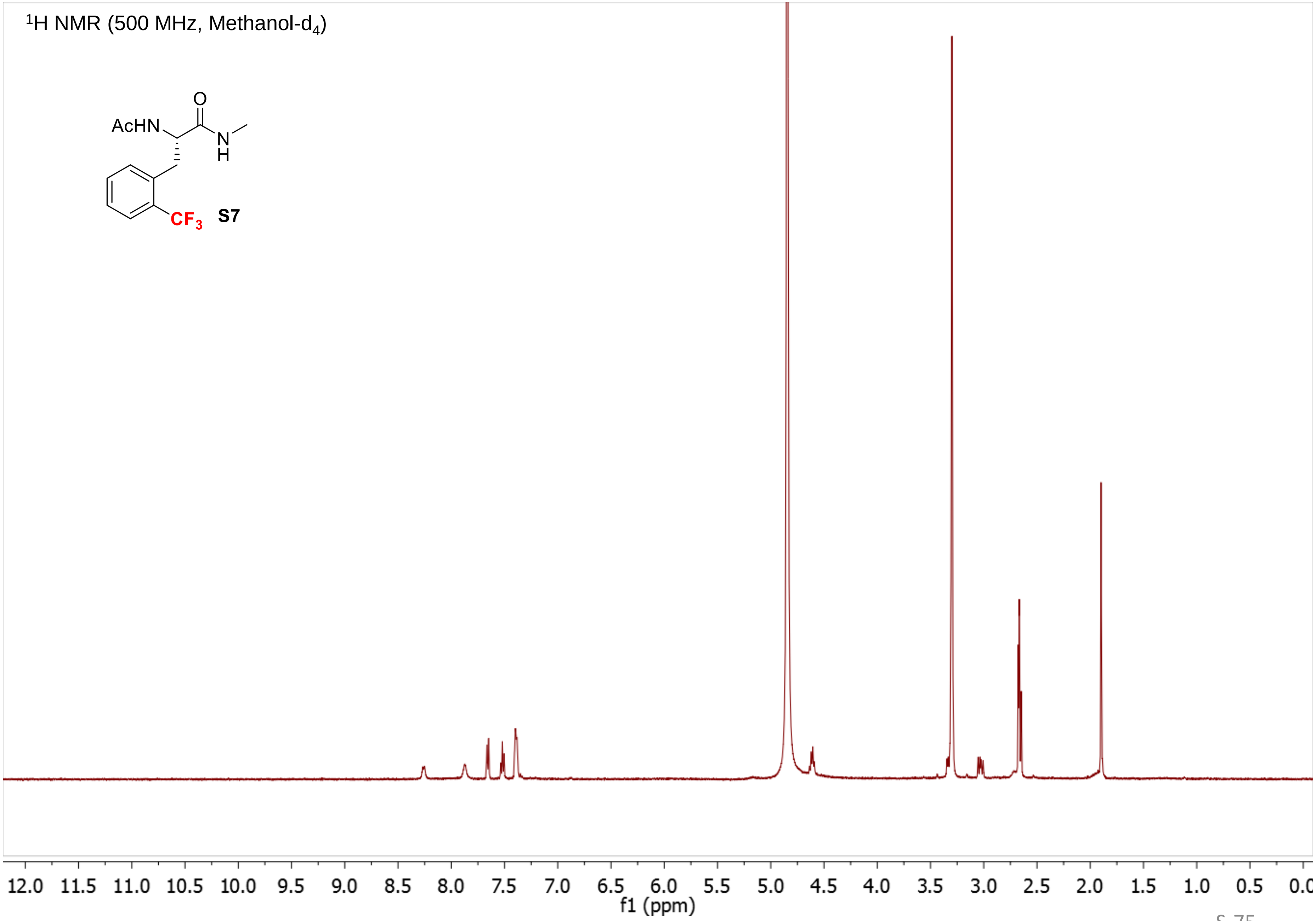

1H NMR (500 MHz, Methanol-d4)
O
AcHN
N
H
CF3
S7
12.0 11.5 11.0 10.5 10.0 9.5 9.0 8.5 8.0 7.5 7.0 6.5 6.0 5.5 5.0 4.5 4.0 3.5 3.0 2.5 2.0 1.5 1.0 0.5
f1 (ppm)

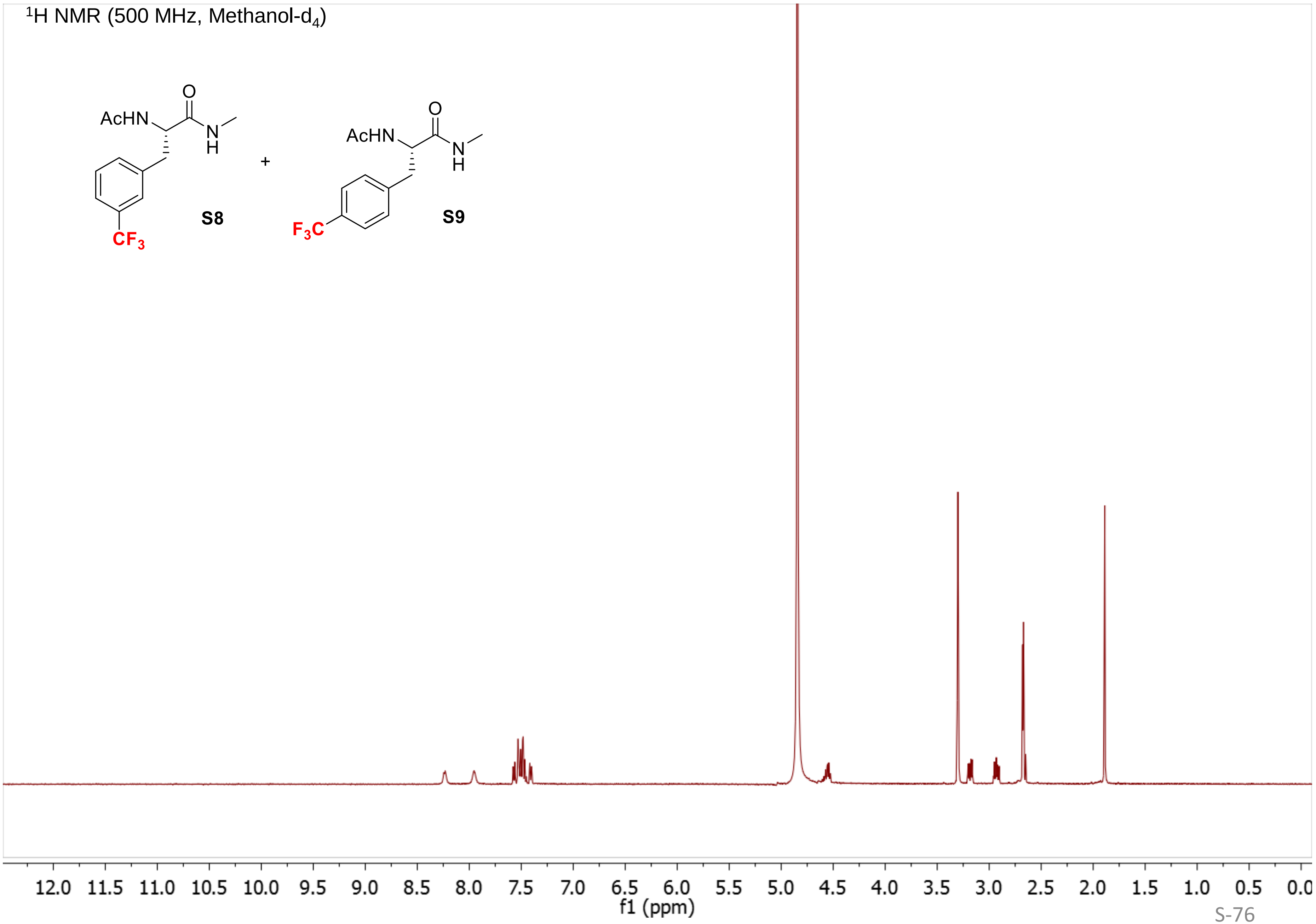
1H NMR (500 MHz, Methanol-d4)
AcHN
O
N
H
CF3
S8
+
AcHN
O
N
H
F3C
S9
12.0 11.5 11.0 10.5 10.0 9.5 9.0 8.5 8.0 7.5 7.0 6.5 6.0 5.5 5.0 4.5 4.0 3.5 3.0 2.5 2.0 1.5 1.0 0.5 0.0
f1 (ppm)

^{19}F NMR (471 MHz, Methanol-d_4)

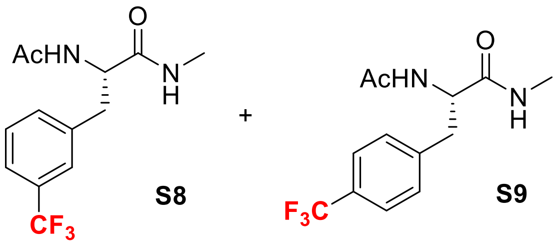

S8

S9

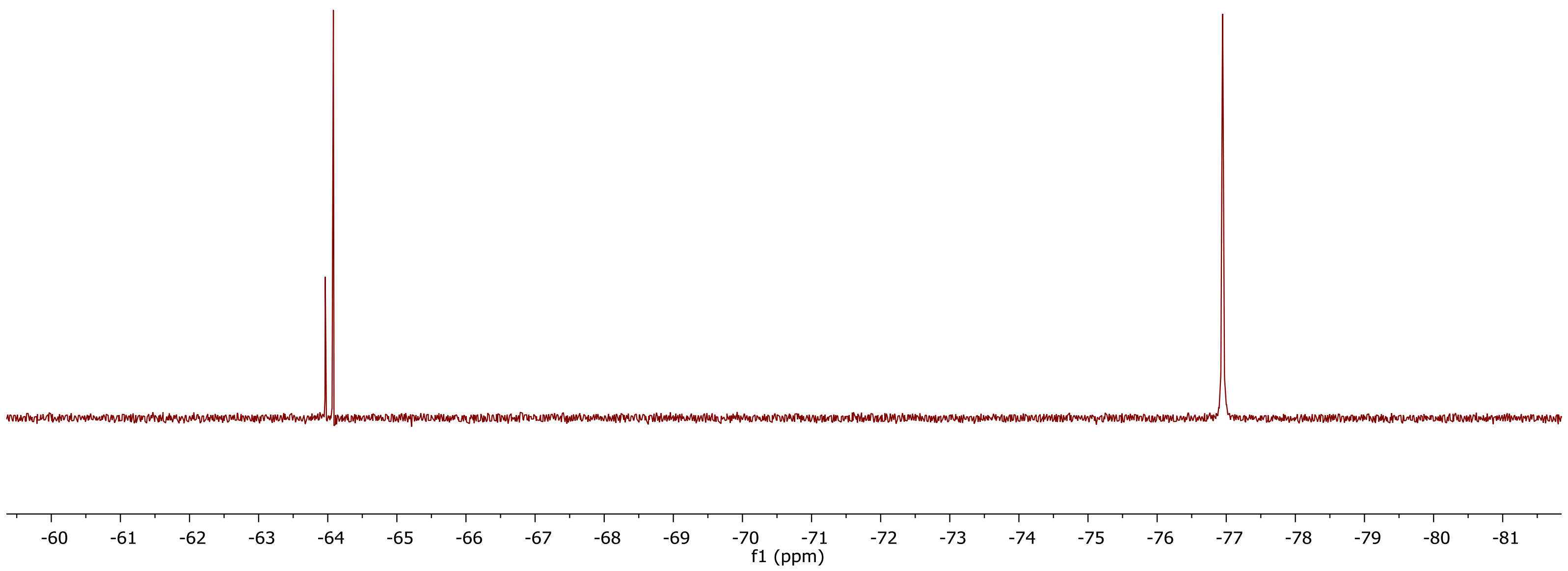

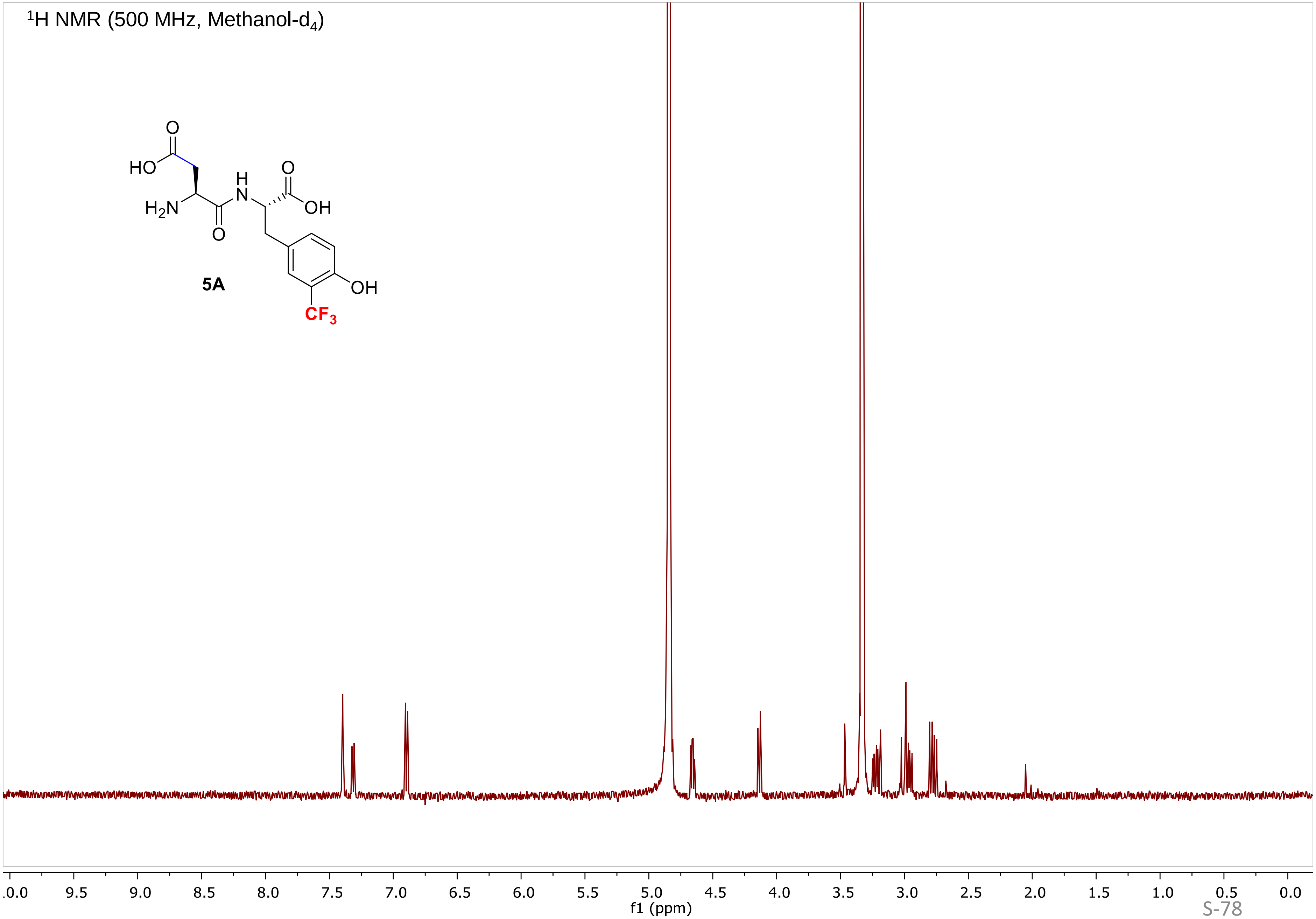

1H NMR (500 MHz, Methanol-d4)
O
HO
H
N
O
H2N
OH
O
5A
OH
CF3
.0.0
9.5
9.0
8.5
8.0
7.5
7.0
6.5
6.0
5.5
5.0
4.5
4.0
3.5
3.0
2.5
2.0
1.5
1.0
0.5
0.0
f1 (ppm)

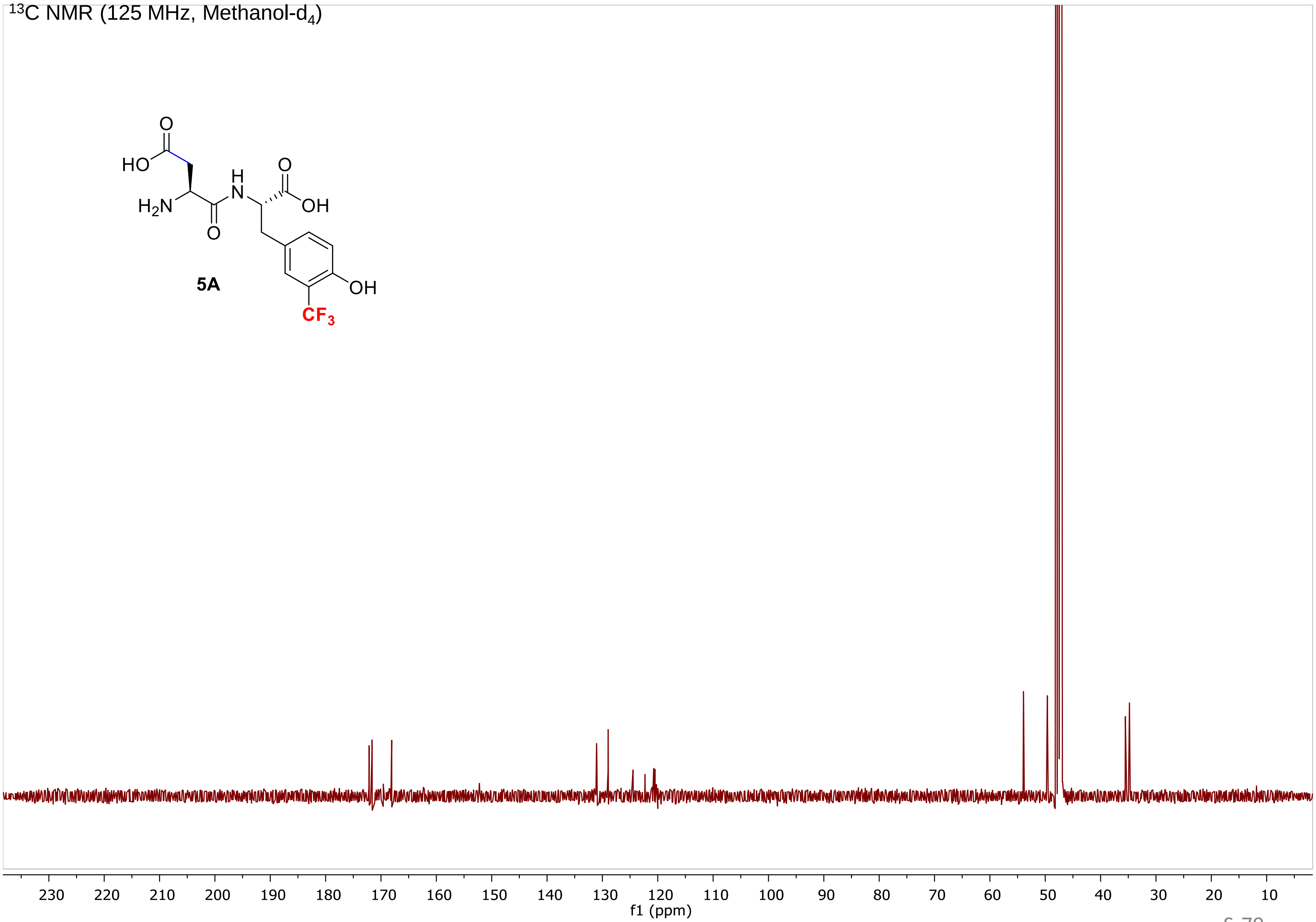
^{13}C NMR (125 MHz, Methanol-d$_4$)
HO
O
H$_2$N
H
N
O
OH
O
OH
CF$_3$
5A
230
220
210
200
190
180
170
160
150
140
130
120
110
100
90
80
70
60
50
40
30
20
10
f1 (ppm)

^{19}F NMR (125 MHz, Methanol-d_4)

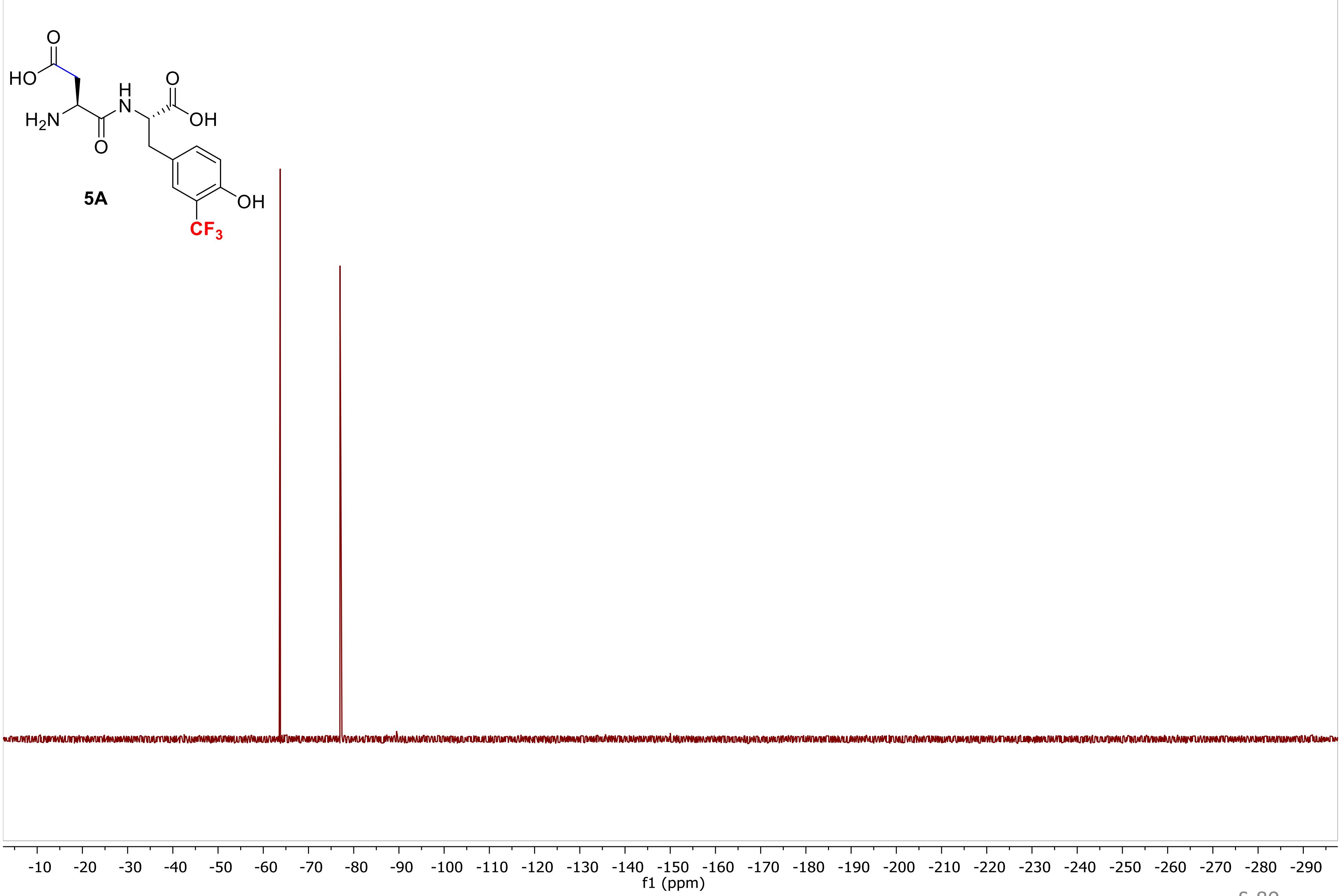

^{1}H NMR (500MHz, Methanol-d_4)

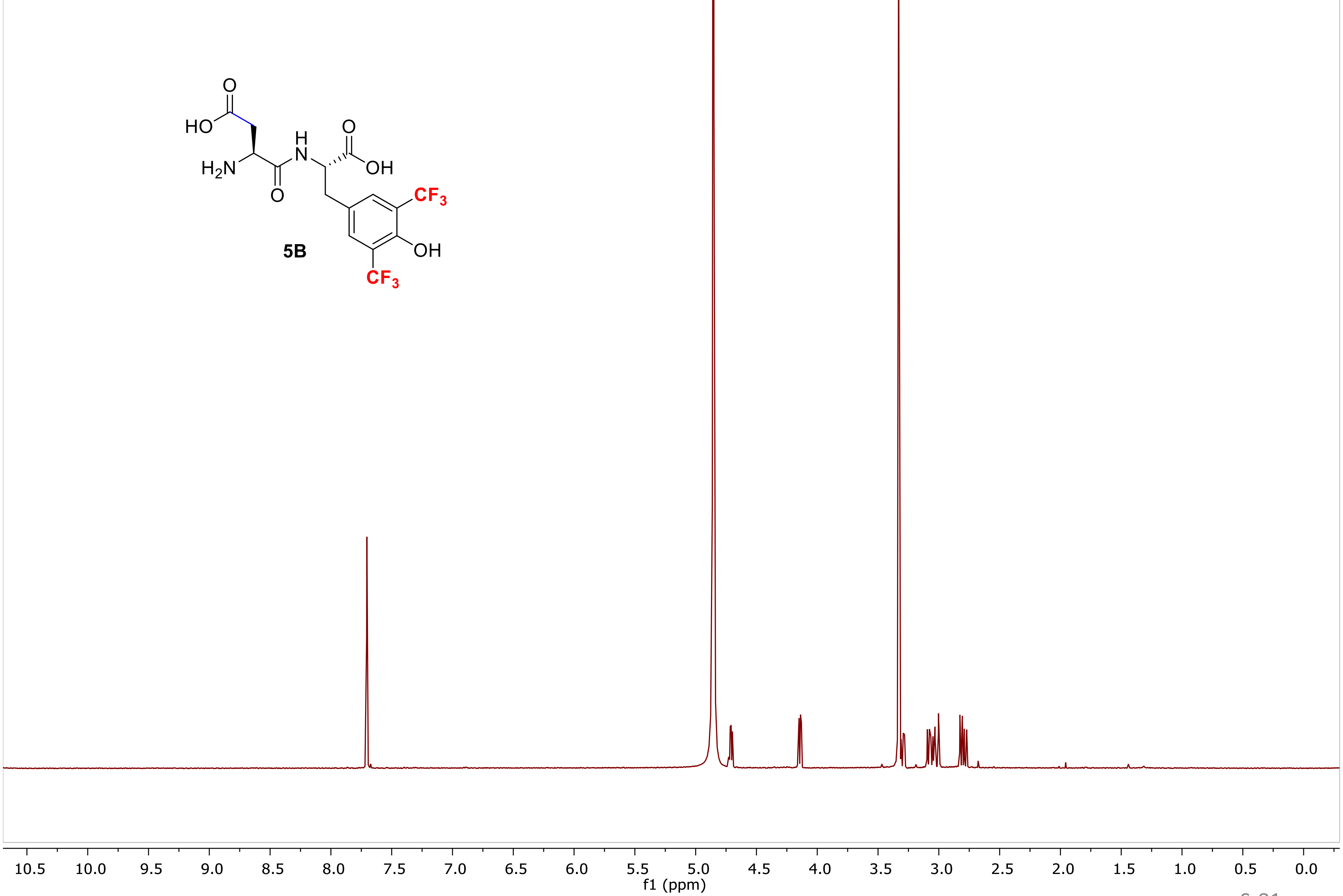

^{19}F NMR (471 MHz, Methanol-d$_4$)

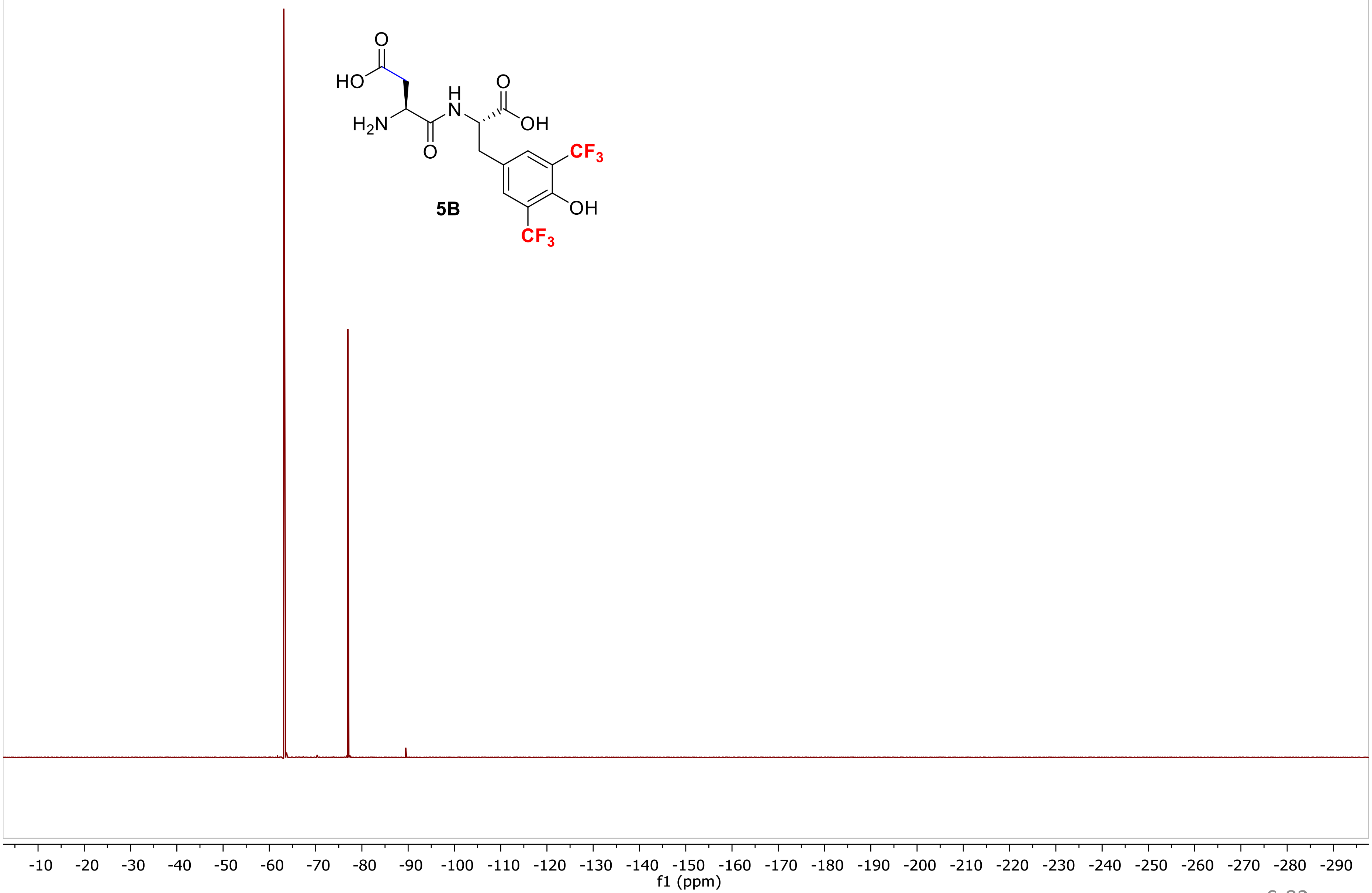

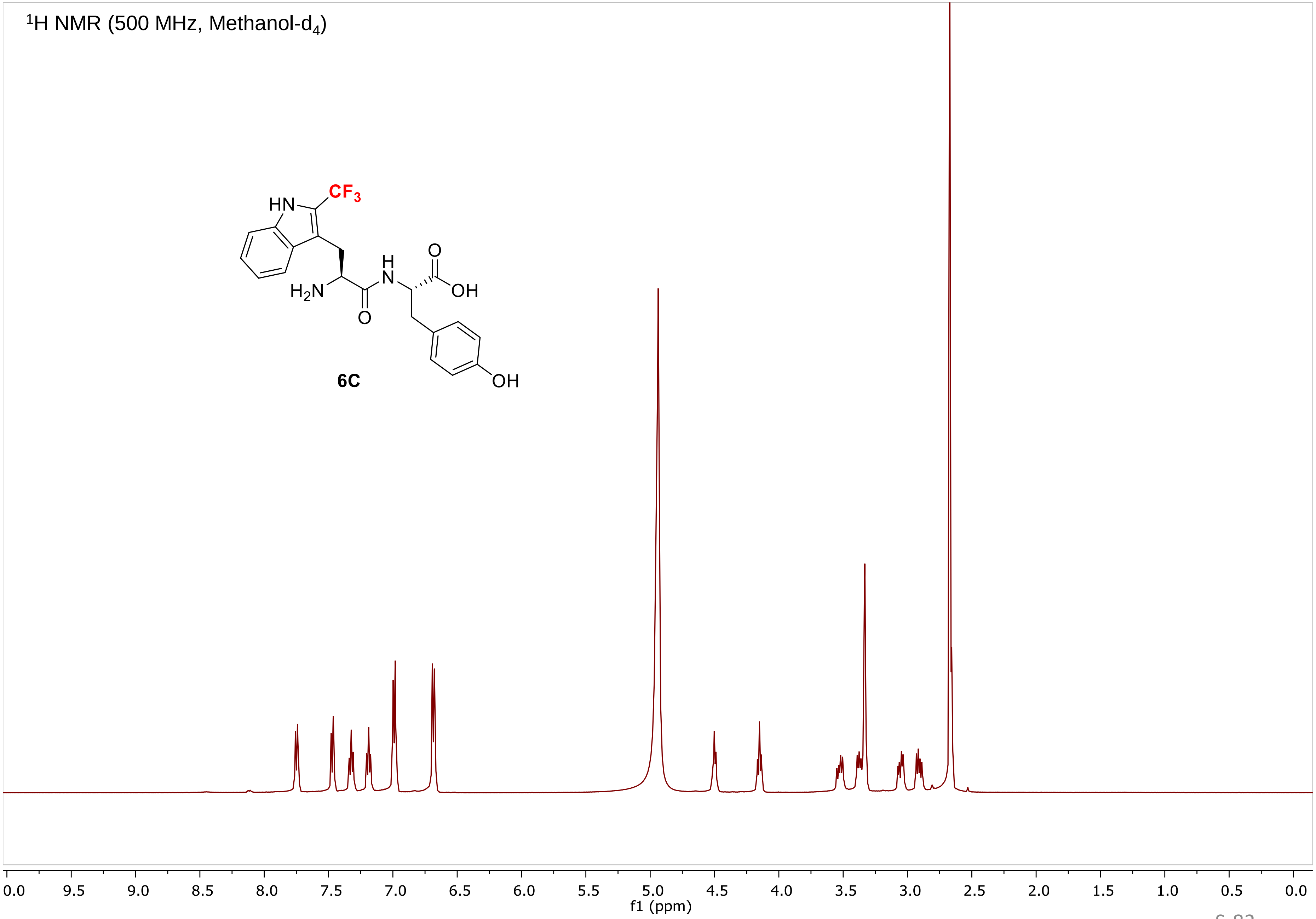

^{1}H NMR (500 MHz, Methanol-d$_4$)
HN
CF$_3$
H$_2$N
H
N
O
OH
O
OH
6C
0.0 9.5 9.0 8.5 8.0 7.5 7.0 6.5 6.0 5.5 5.0 4.5 4.0 3.5 3.0 2.5 2.0 1.5 1.0 0.5 0.0
f1 (ppm)

^{13}C NMR (125 MHz, Methanol-d_4)

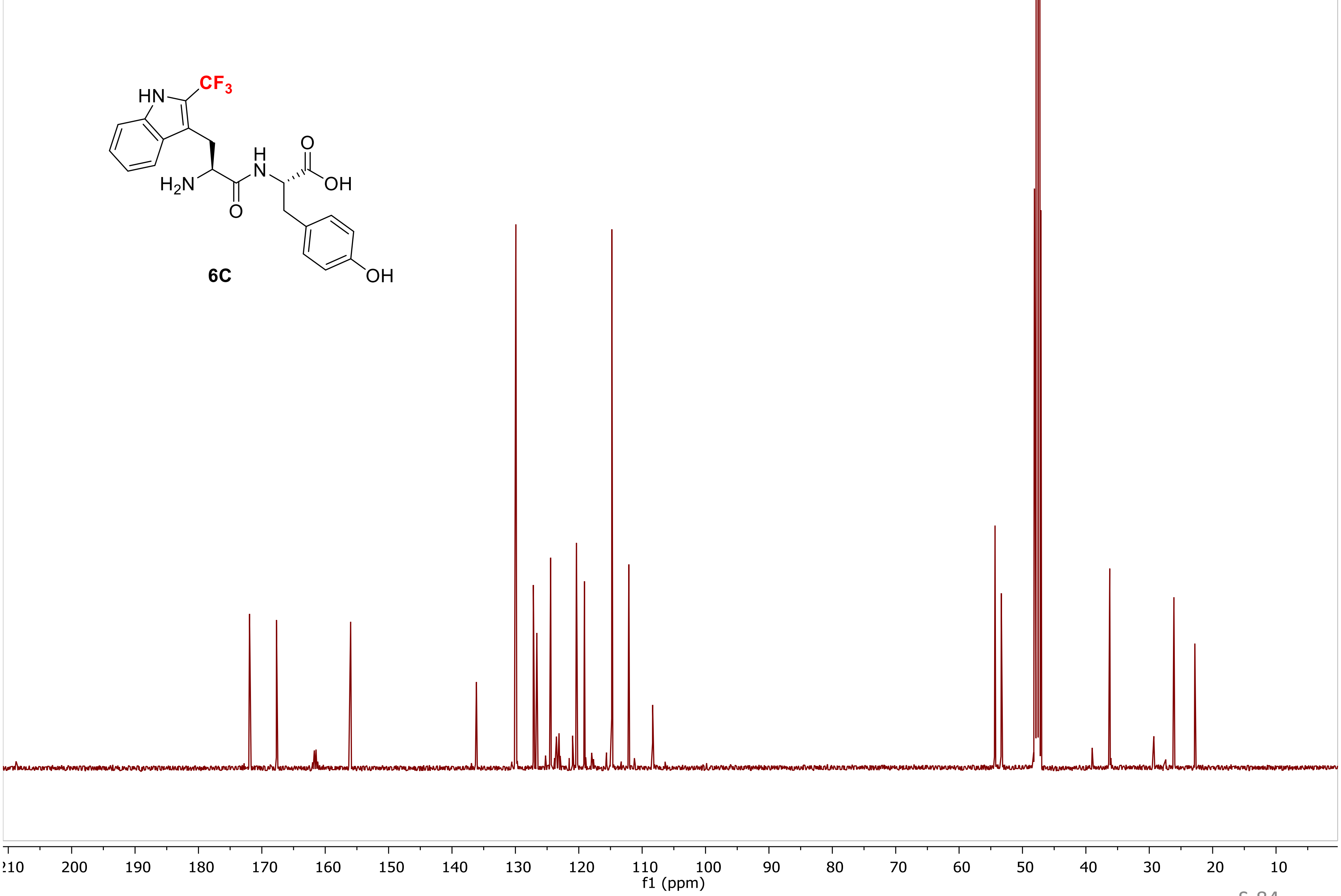

^{19}F NMR (471 MHz, Methanol-d_4)

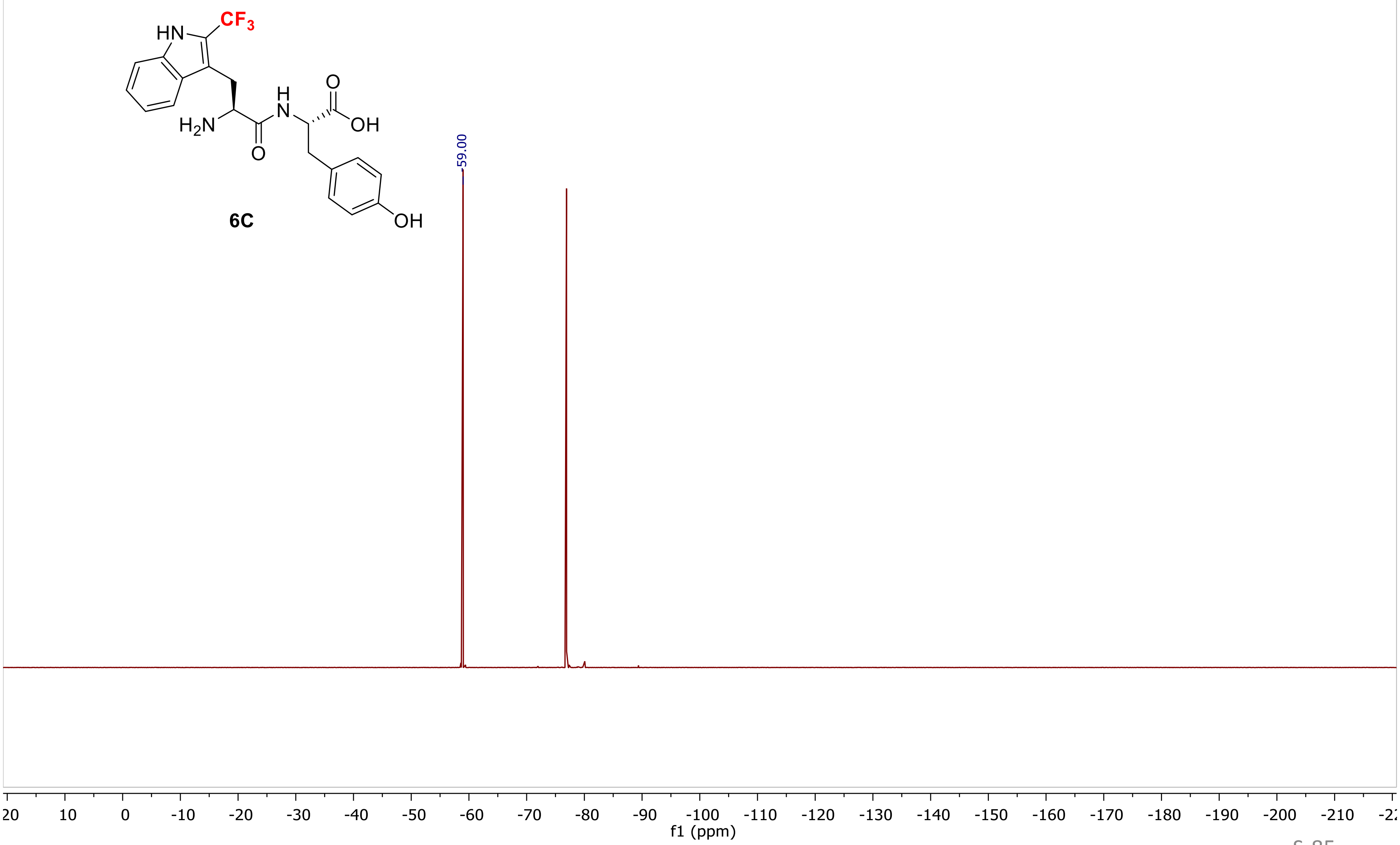

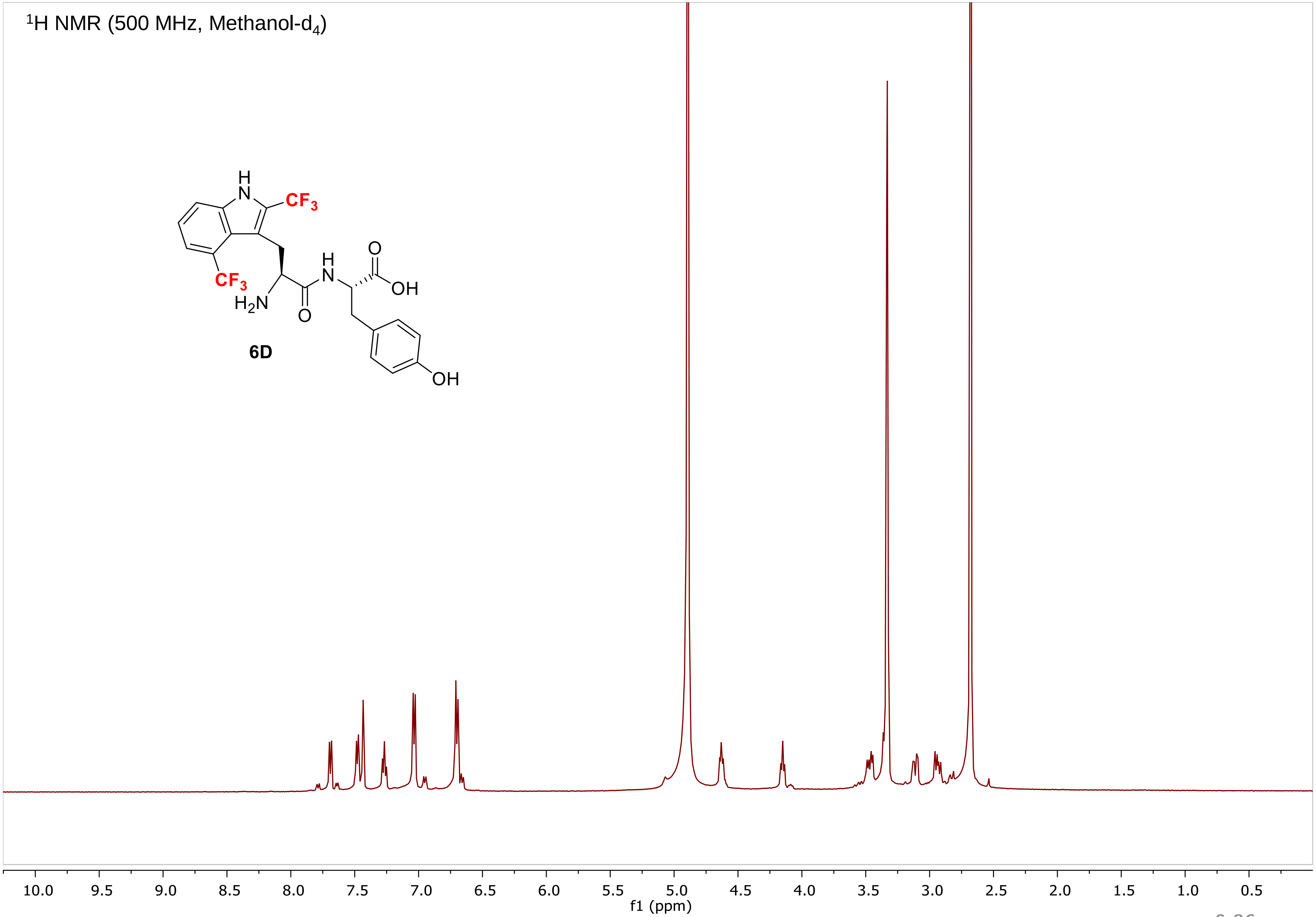
^{1}H NMR (500 MHz, Methanol-d$_4$)
H
N
CF$_3$
CF$_3$
H
N
O
OH
H$_2$N
O
6D
OH
10.0 9.5 9.0 8.5 8.0 7.5 7.0 6.5 6.0 5.5 5.0 4.5 4.0 3.5 3.0 2.5 2.0 1.5 1.0 0.5
f1 (ppm)

^{13}C NMR (125 MHz, Methanol-d_4)

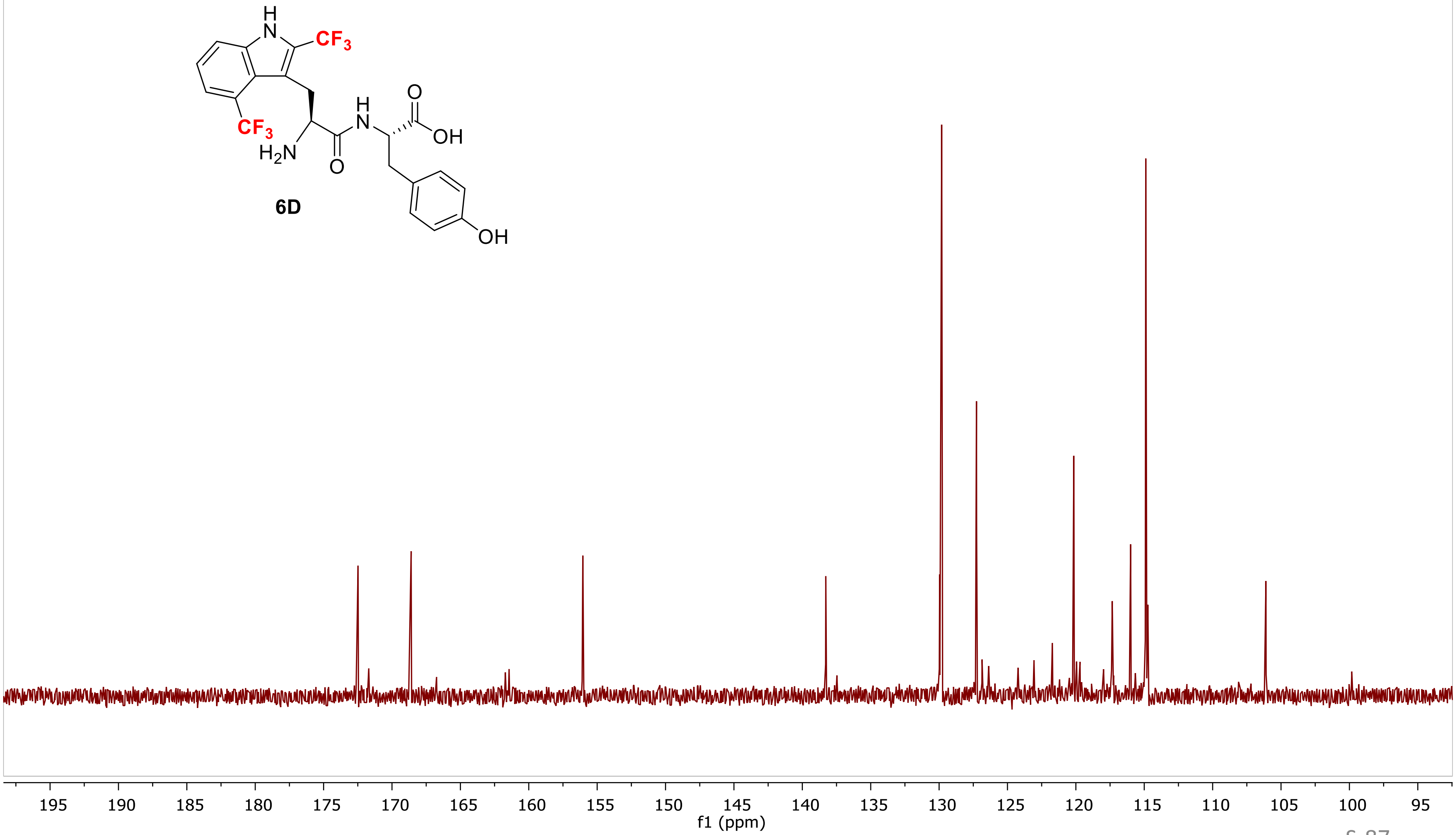

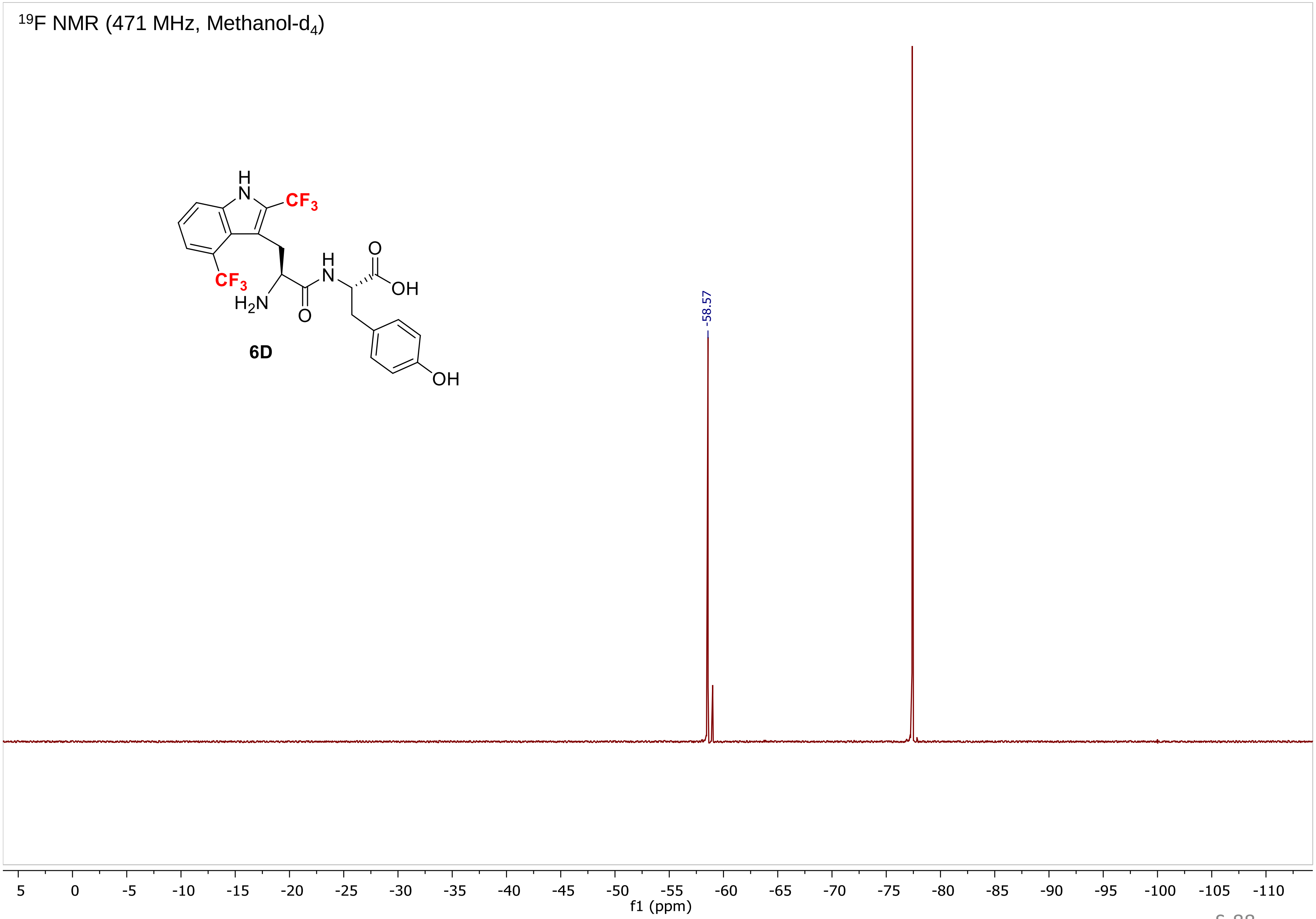

^{19}F NMR (471 MHz, Methanol-d_4)
6D
-58.57
5 0 -5 -10 -15 -20 -25 -30 -35 -40 -45 -50 -55 -60 -65 -70 -75 -80 -85 -90 -95 -100 -105 -110
f1 (ppm)

^{1}H NMR (500 MHz, Methanol-d_4)

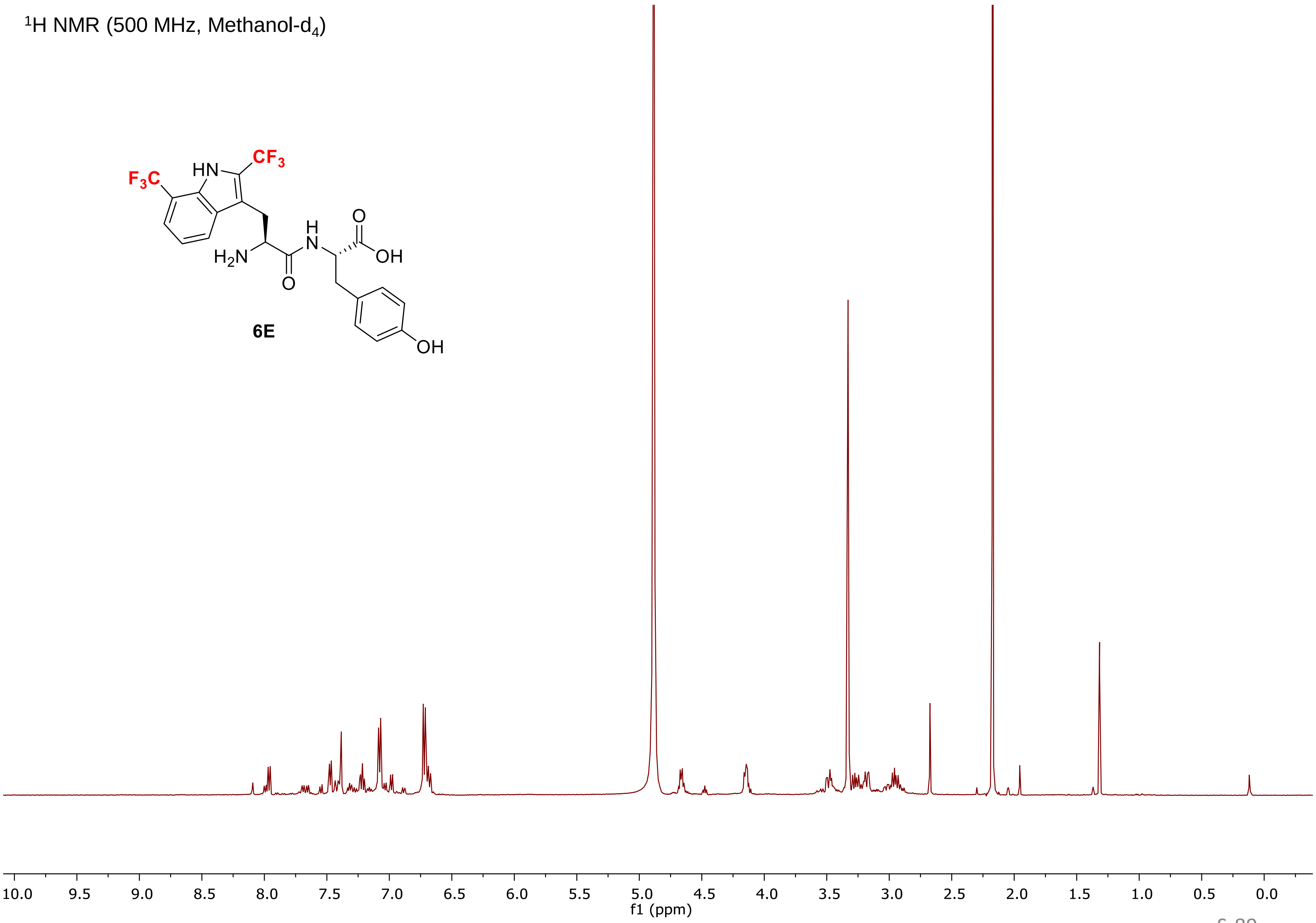

^{19}F NMR (471 MHz, Methanol-d_4)

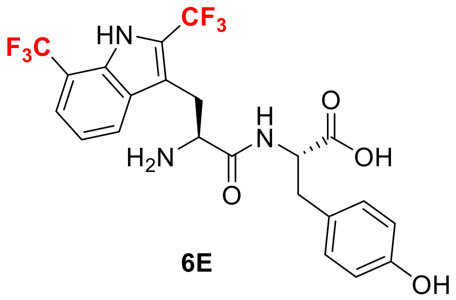

6E

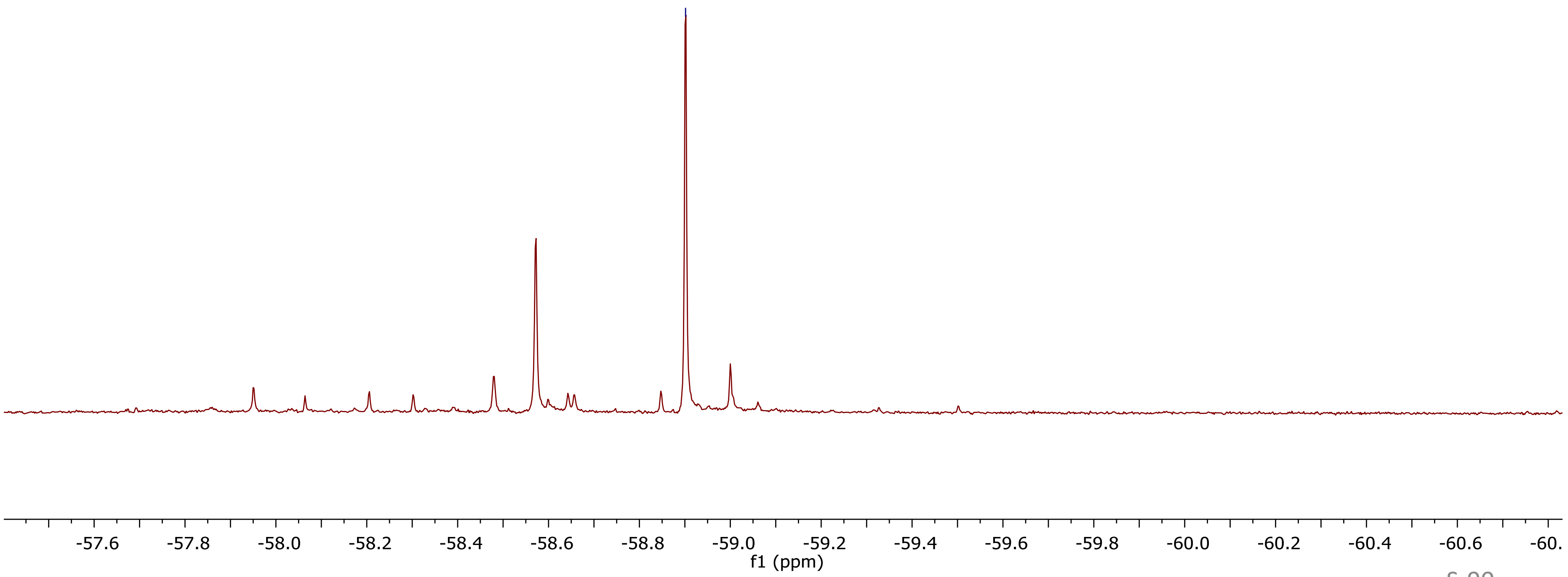

^{1}H NMR (500 MHz, Methanol-d_4)

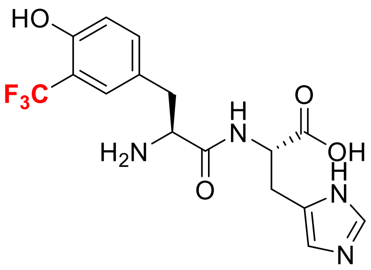

7A

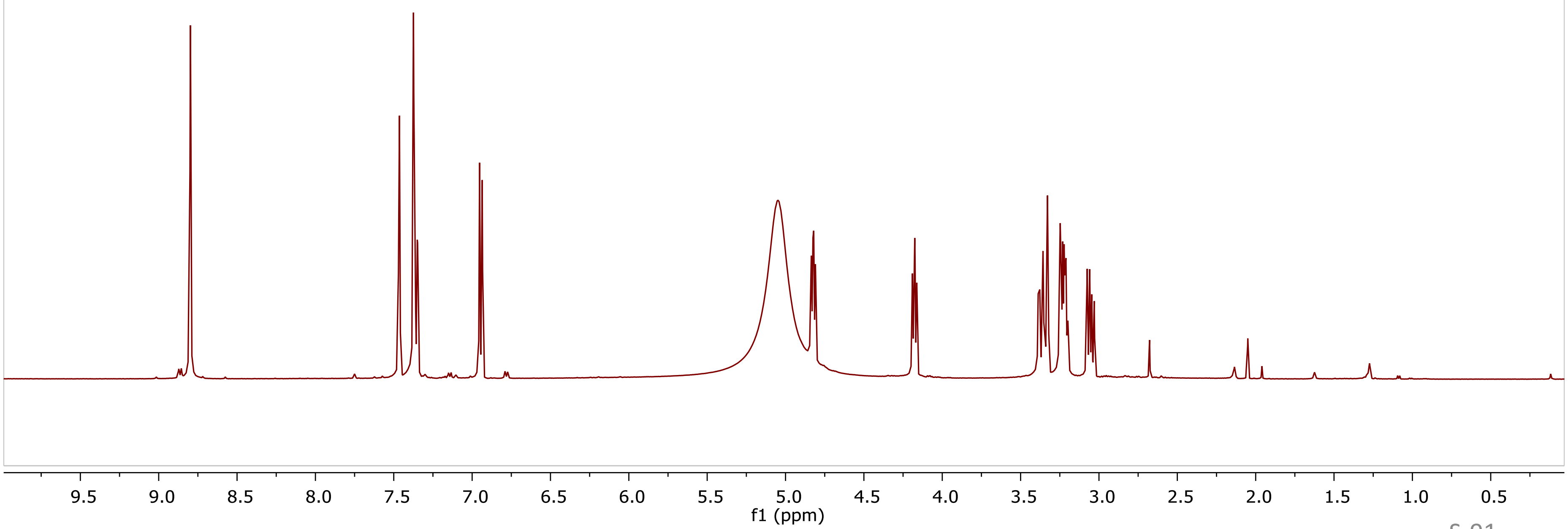

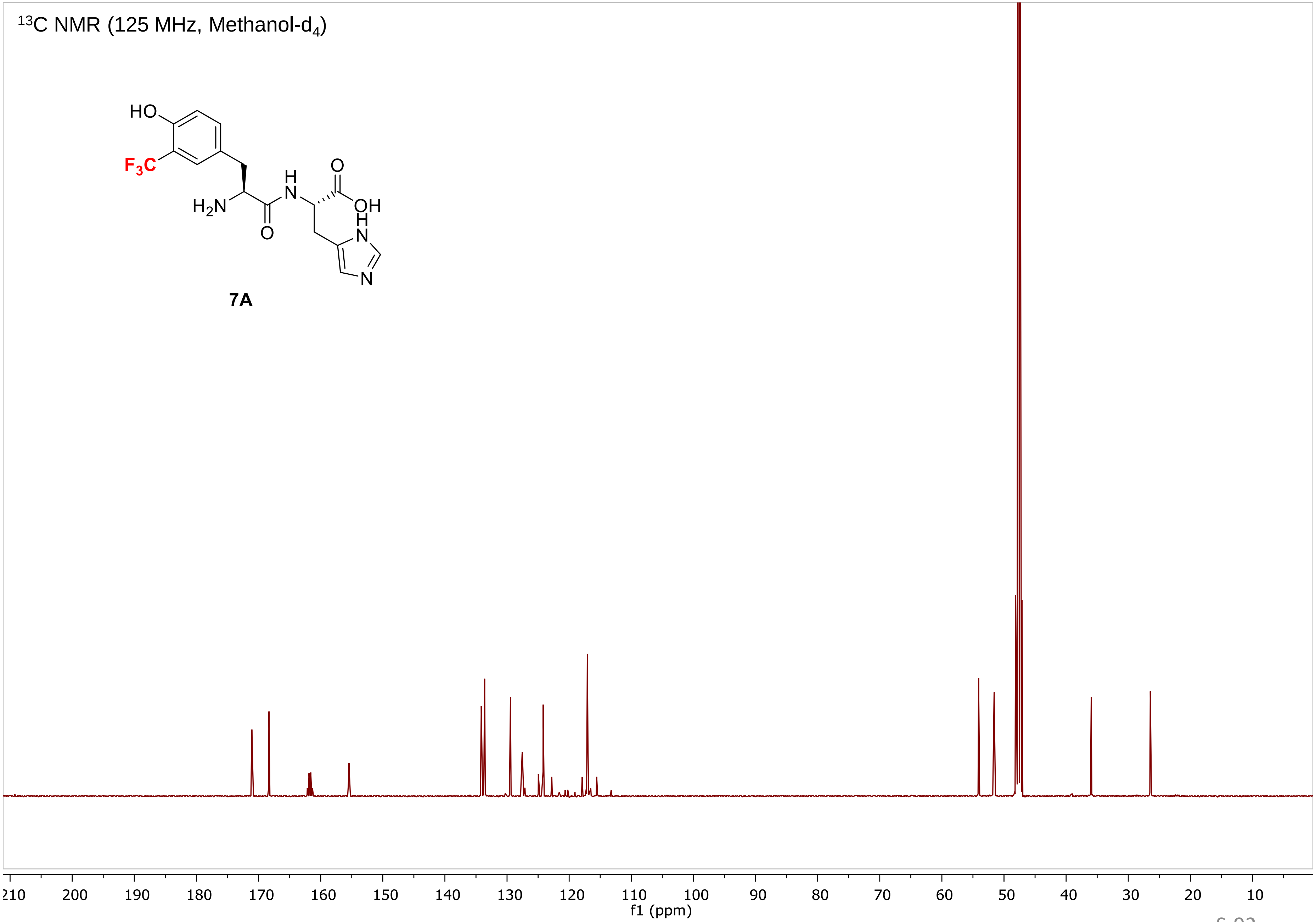

13C NMR (125 MHz, Methanol-d4)
HO
F3C
H2N
O
H
N
OH
H
N
N
7A
210
200
190
180
170
160
150
140
130
120
110
100
90
80
70
60
50
40
30
20
10
f1 (ppm)

^{19}F NMR (471 MHz, Methanol-d_4)

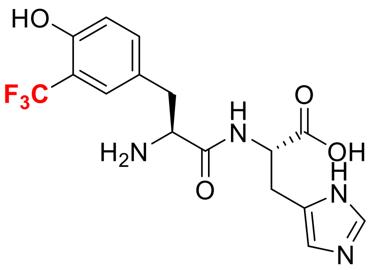

7A

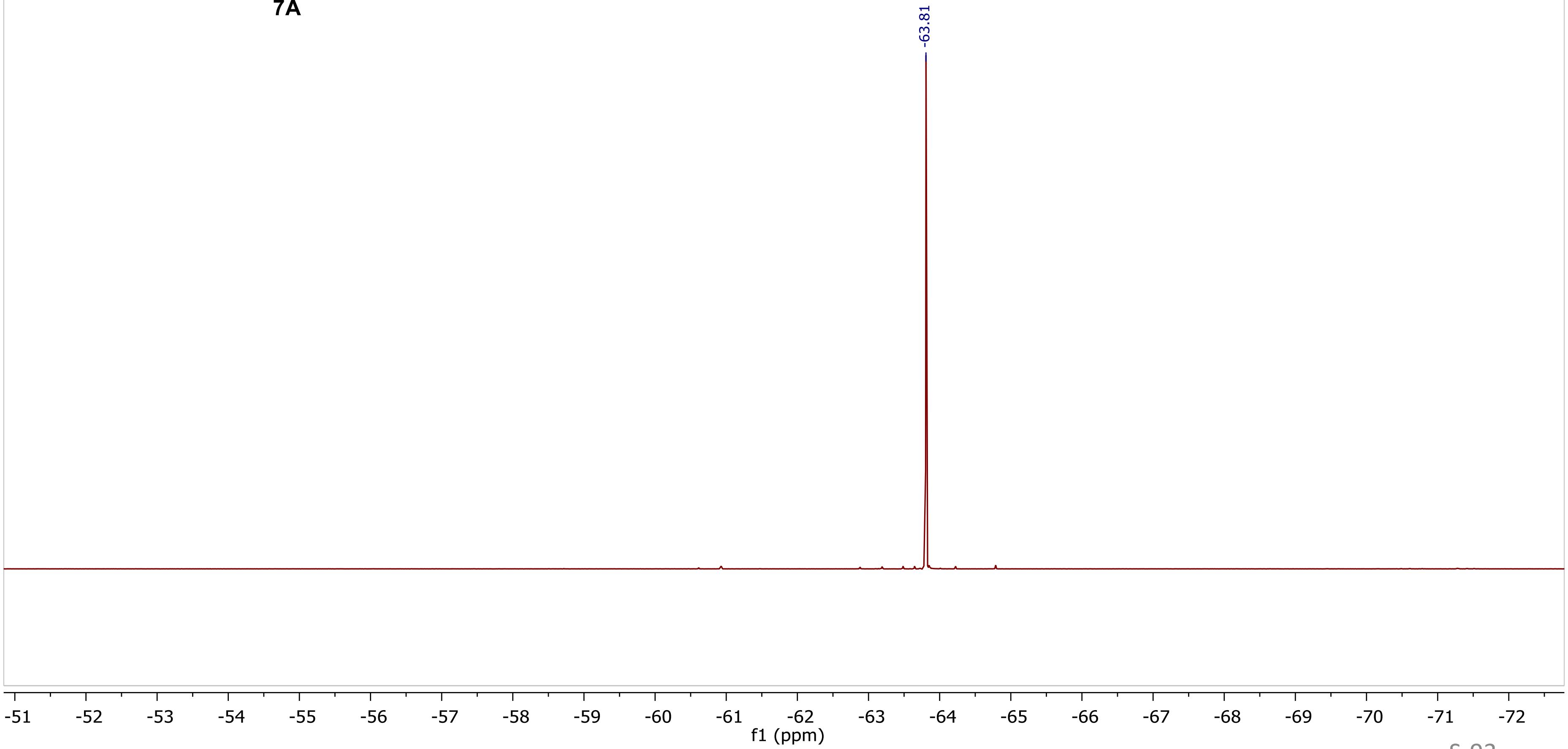

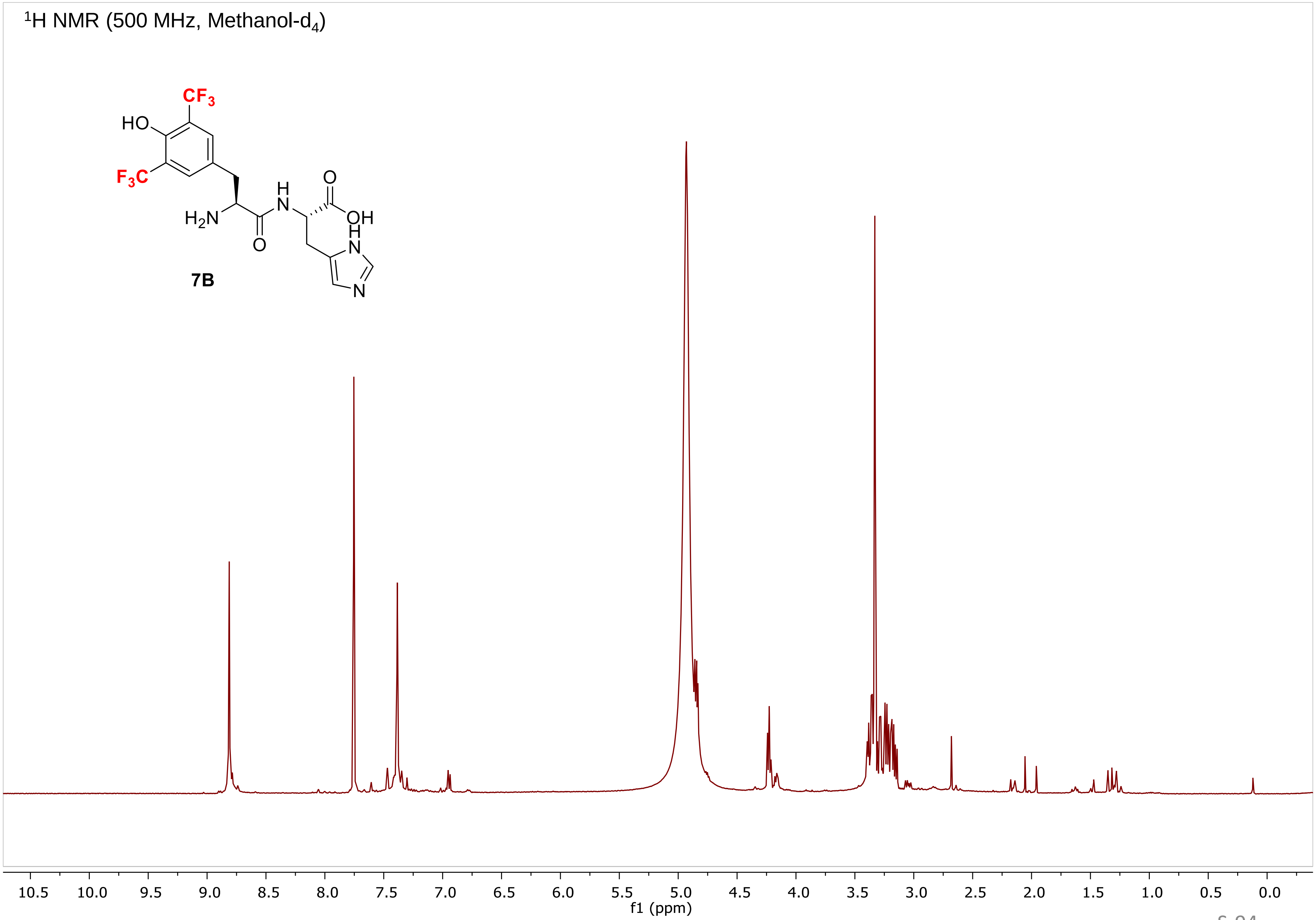
^{1}H NMR (500 MHz, Methanol-d$_4$)
CF$_3$
HO
F$_3$C
H$_2$N
H
N
O
OH
H
N
N
O
7B
10.5 10.0 9.5 9.0 8.5 8.0 7.5 7.0 6.5 6.0 5.5 5.0 4.5 4.0 3.5 3.0 2.5 2.0 1.5 1.0 0.5 0.0
f1 (ppm)

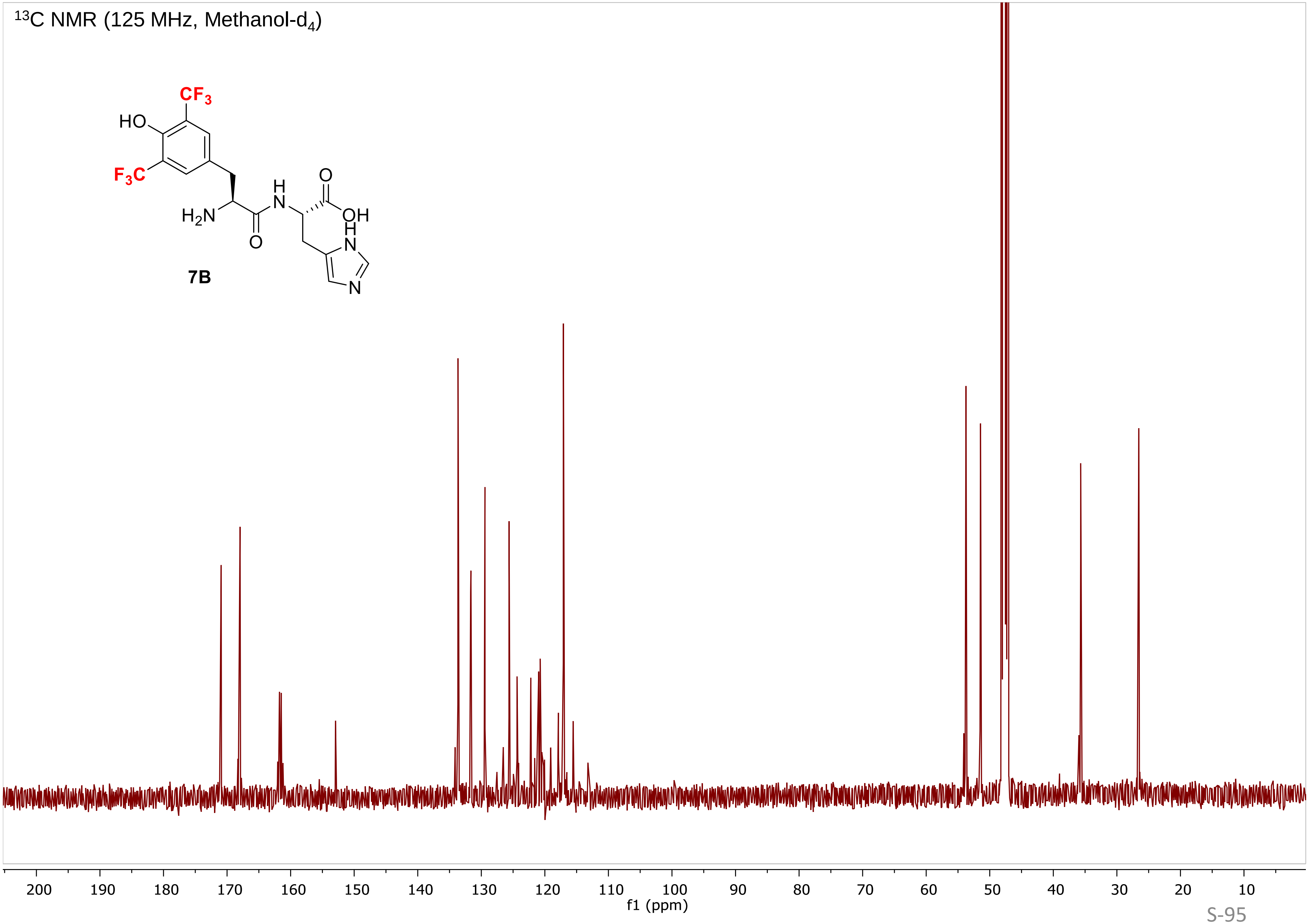

^{13}C NMR (125 MHz, Methanol-d$_4$)
CF$_3$
HO
F$_3$C
H$_2$N
O
H
N
O
OH
H
N
N
7B
200
190
180
170
160
150
140
130
120
110
100
90
80
70
60
50
40
30
20
10
f1 (ppm)

^{19}F NMR (471 MHz, Methanol-d_4)

7B

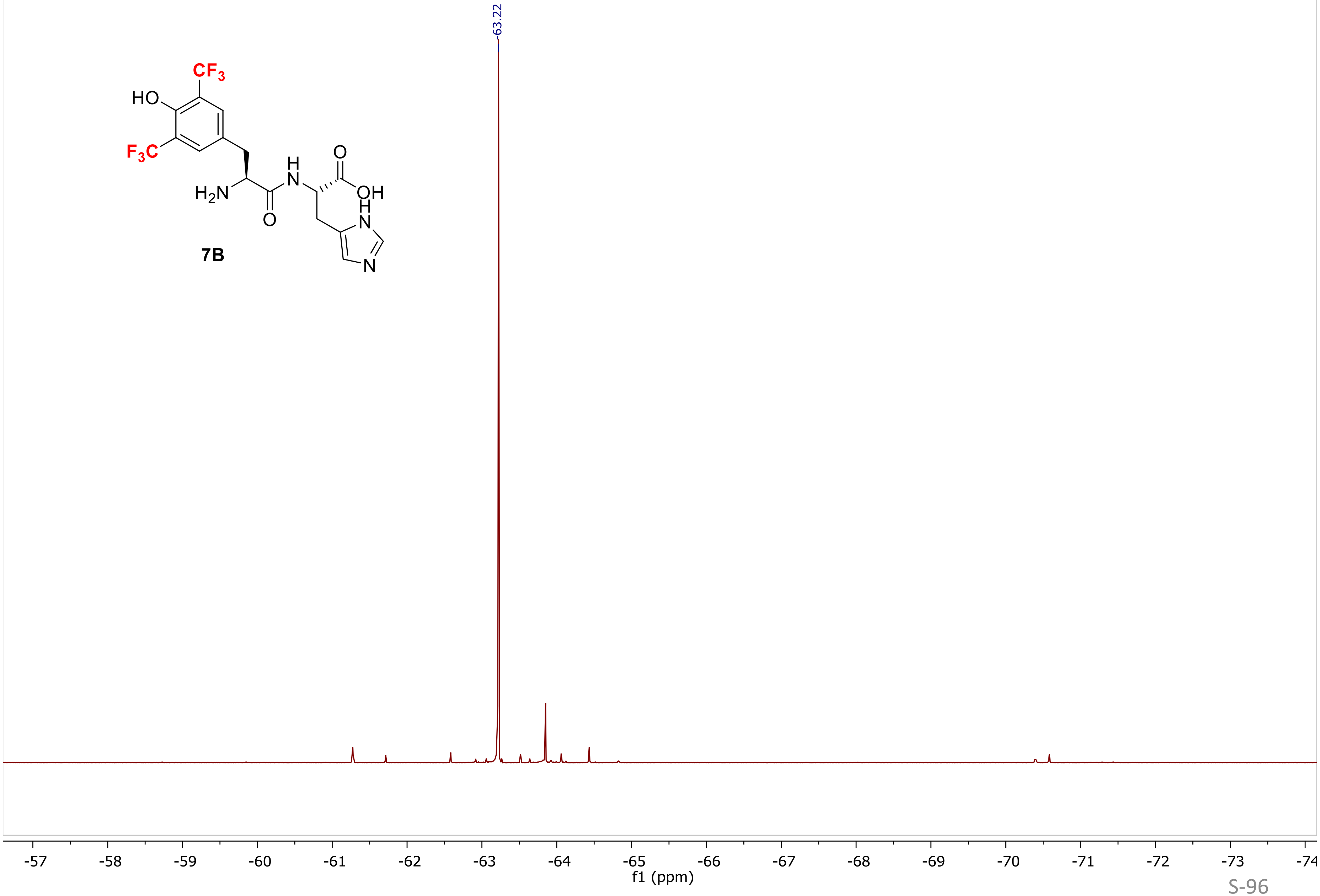

^1H NMR (500 MHz, Methanol-d_4)

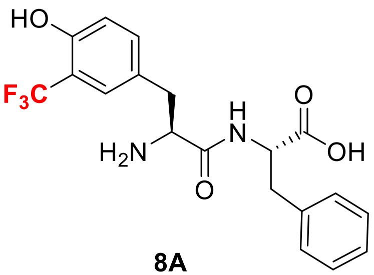

8A

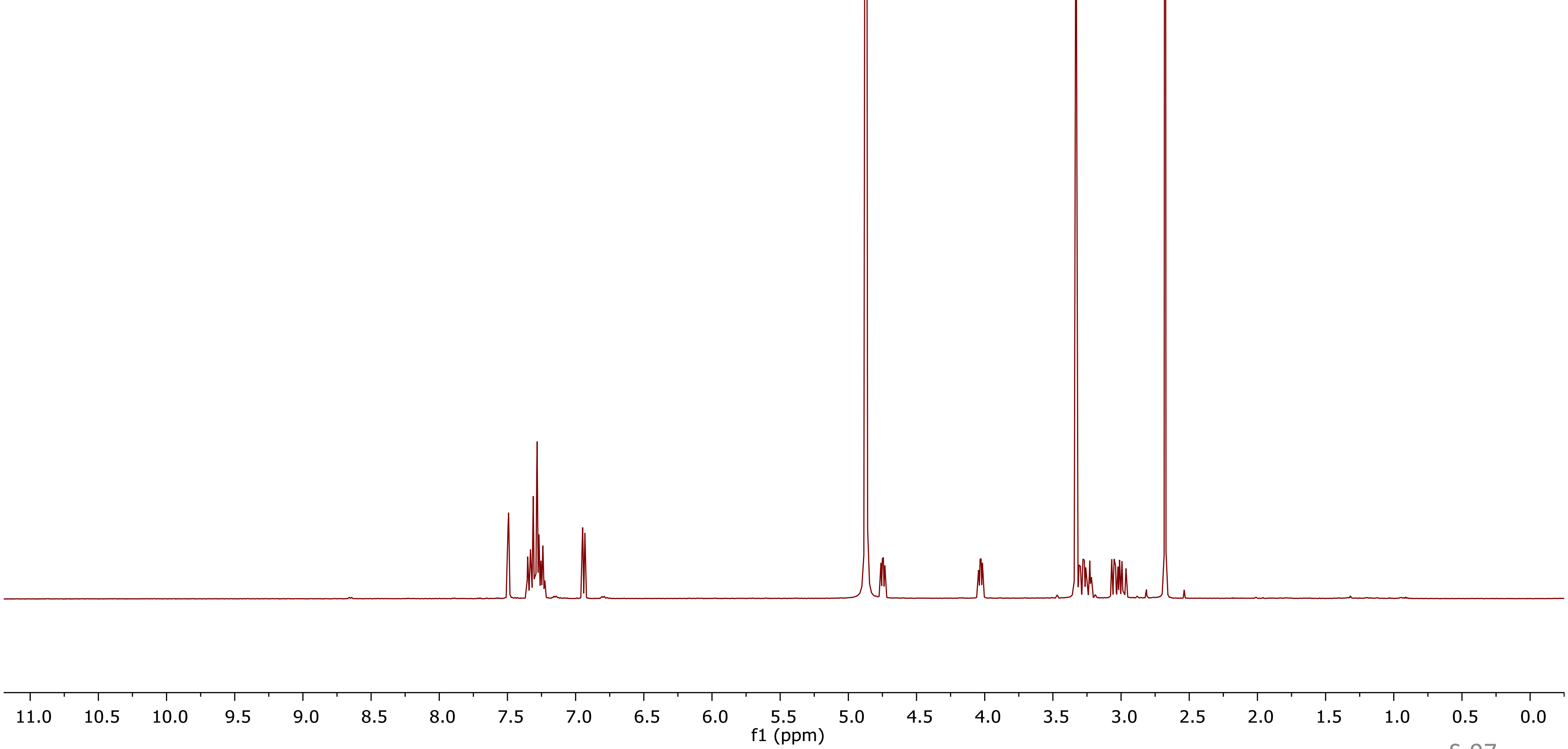

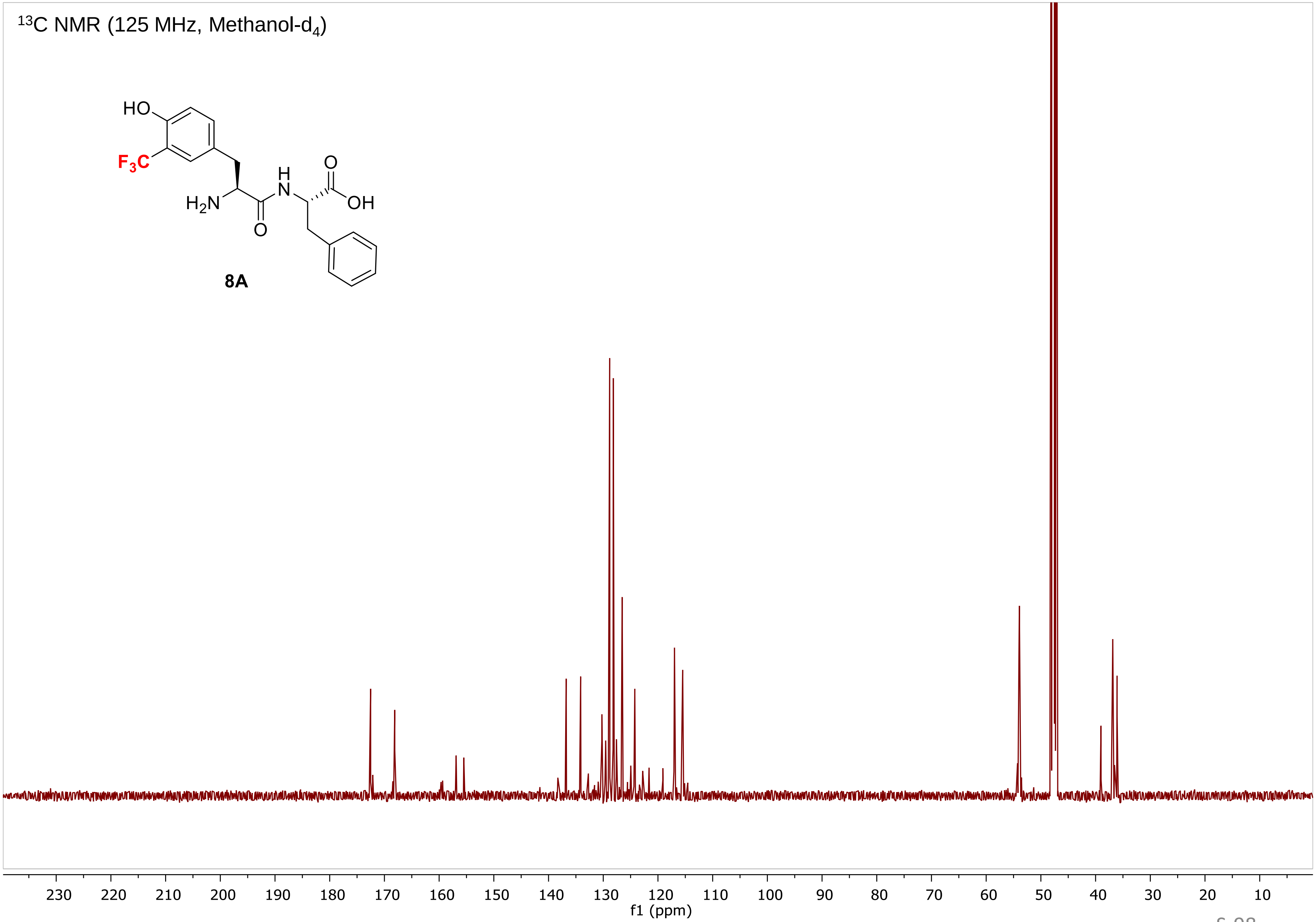

13C NMR (125 MHz, Methanol-d4)
HO
F3C
H2N
H
N
O
OH
O
8A
230
220
210
200
190
180
170
160
150
140
130
120
110
100
90
80
70
60
50
40
30
20
10
f1 (ppm)

^{19}F NMR (471 MHz, Methanol-d_4)

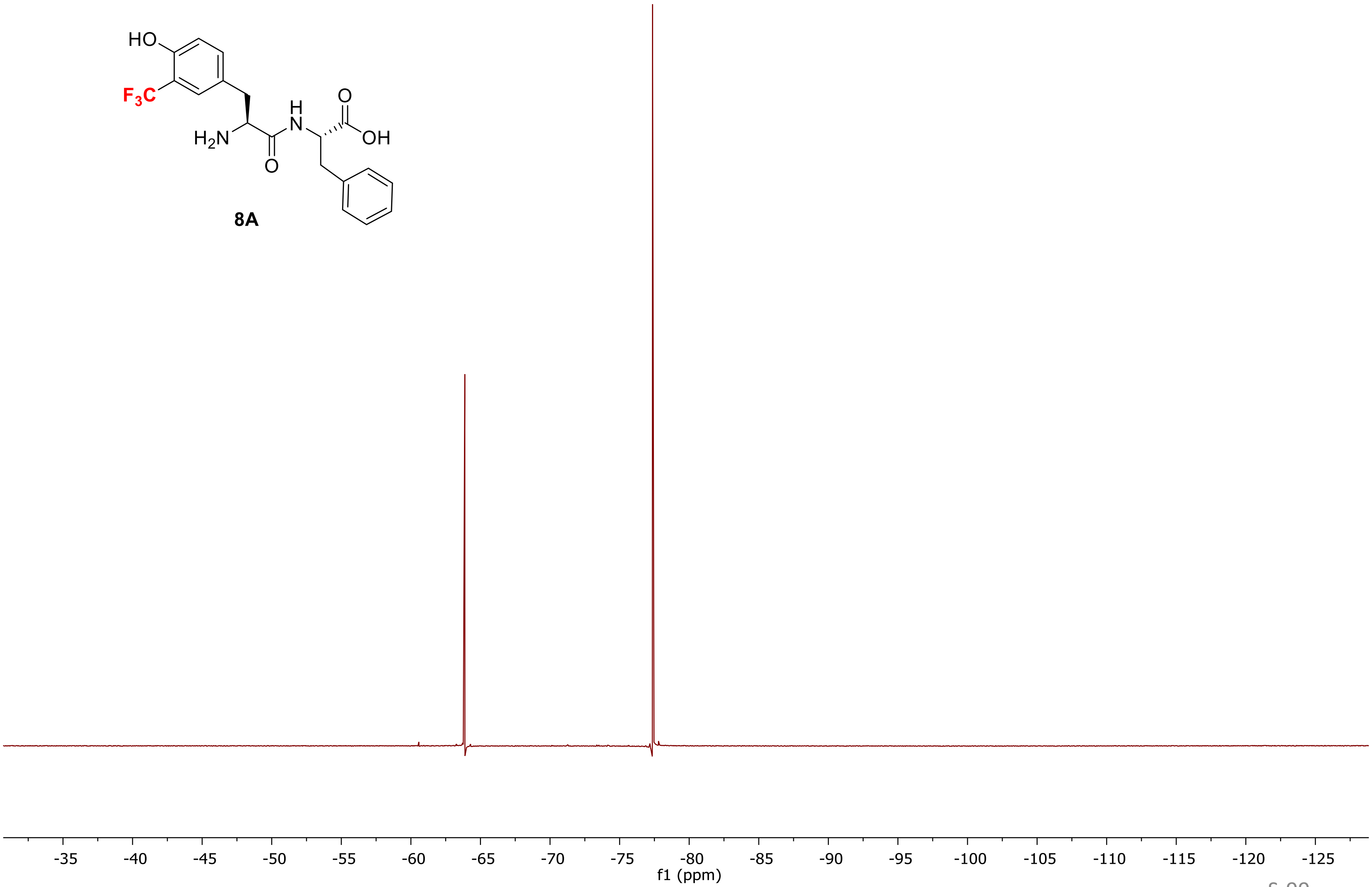

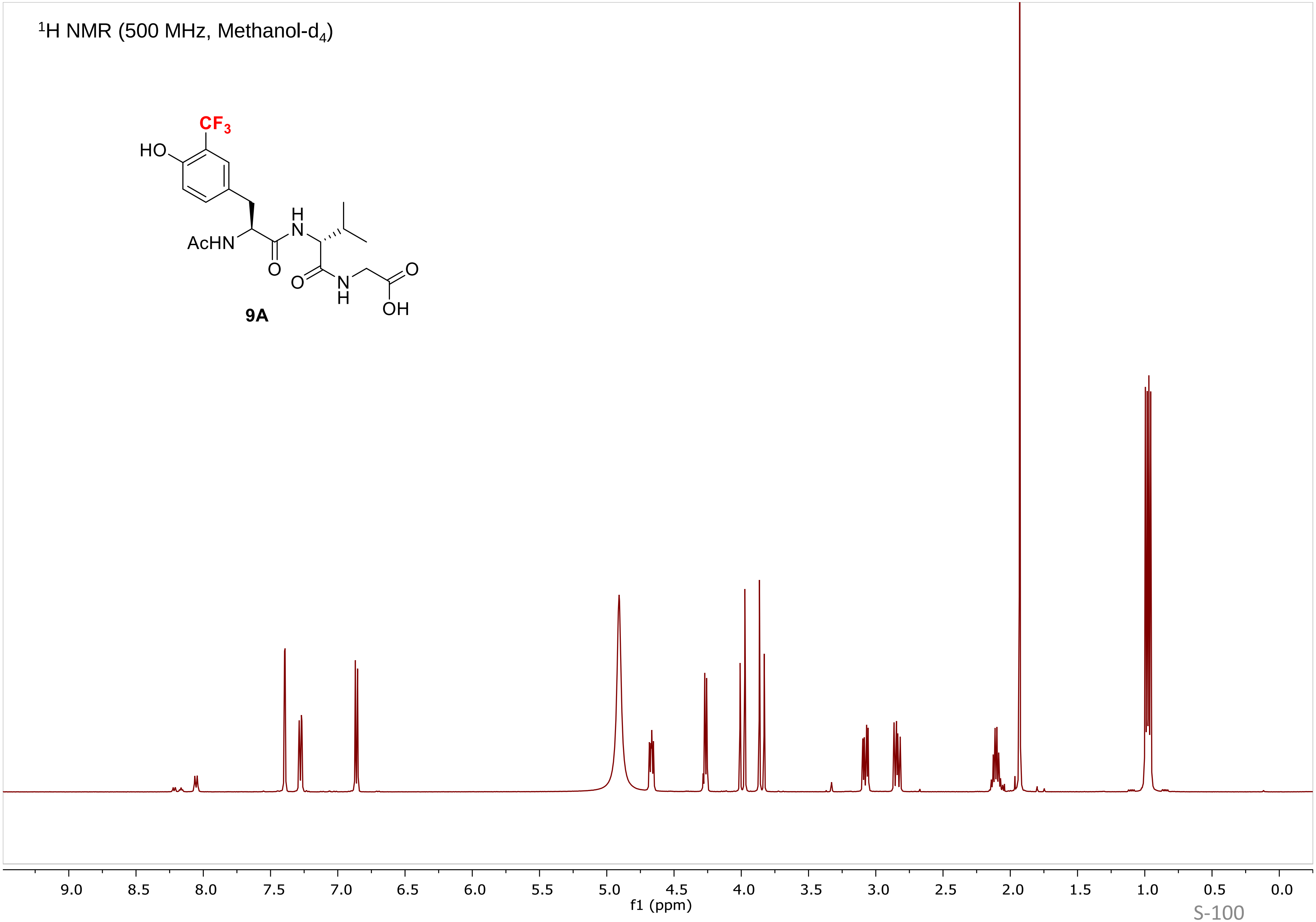

^{1}H NMR (500 MHz, Methanol-d$_4$)
CF3
HO
AcHN
H
N
O
O
N
H
O
OH
9A
9.0
8.5
8.0
7.5
7.0
6.5
6.0
5.5
5.0
4.5
4.0
3.5
3.0
2.5
2.0
1.5
1.0
0.5
0.0
f1 (ppm)

^{13}C NMR (125 MHz, Methanol-d_4)

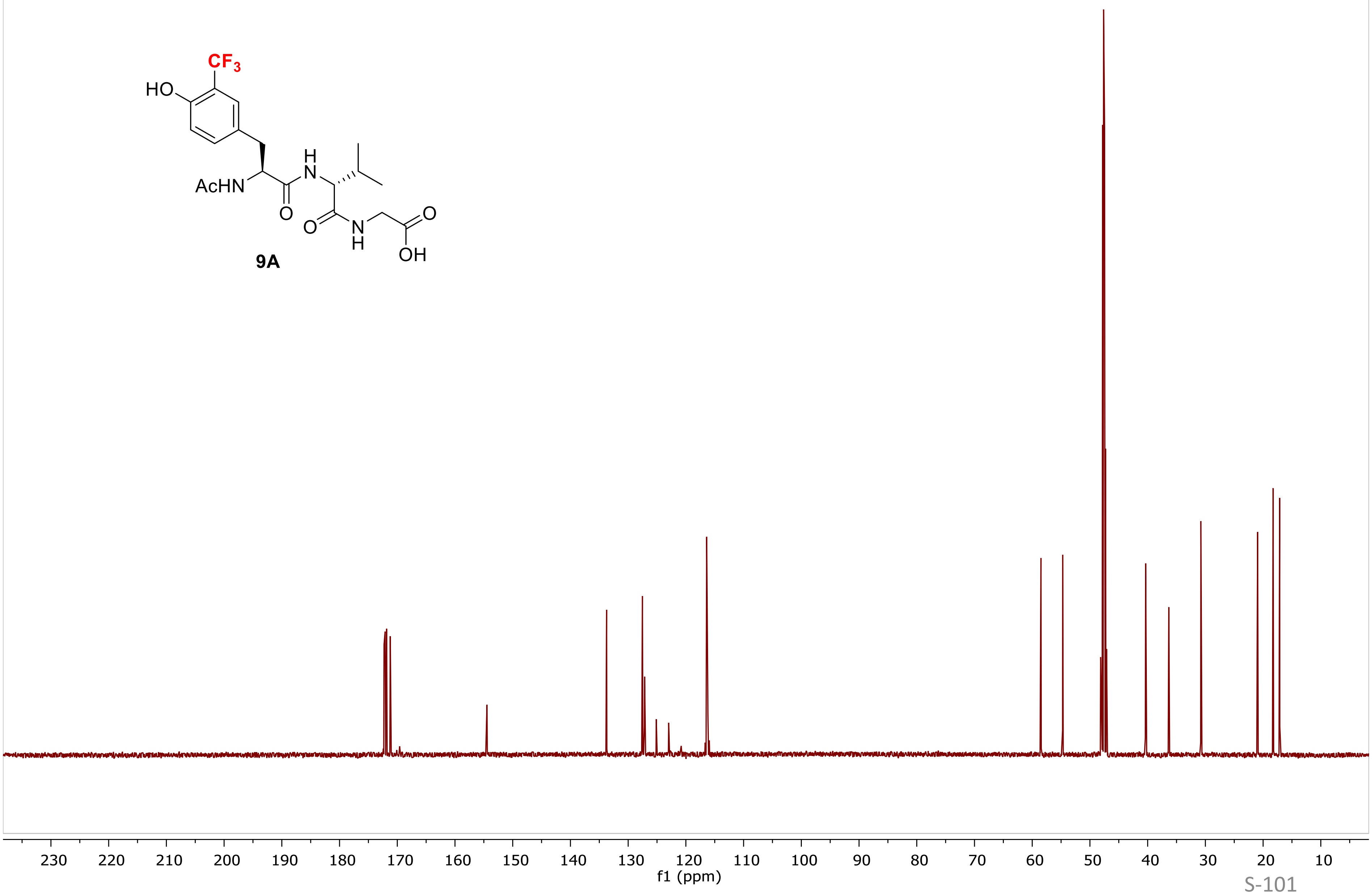

^{19}F NMR (471 MHz, Methanol-d_4)

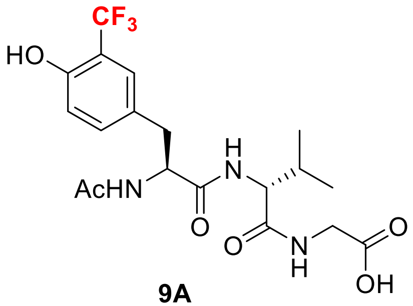

9A

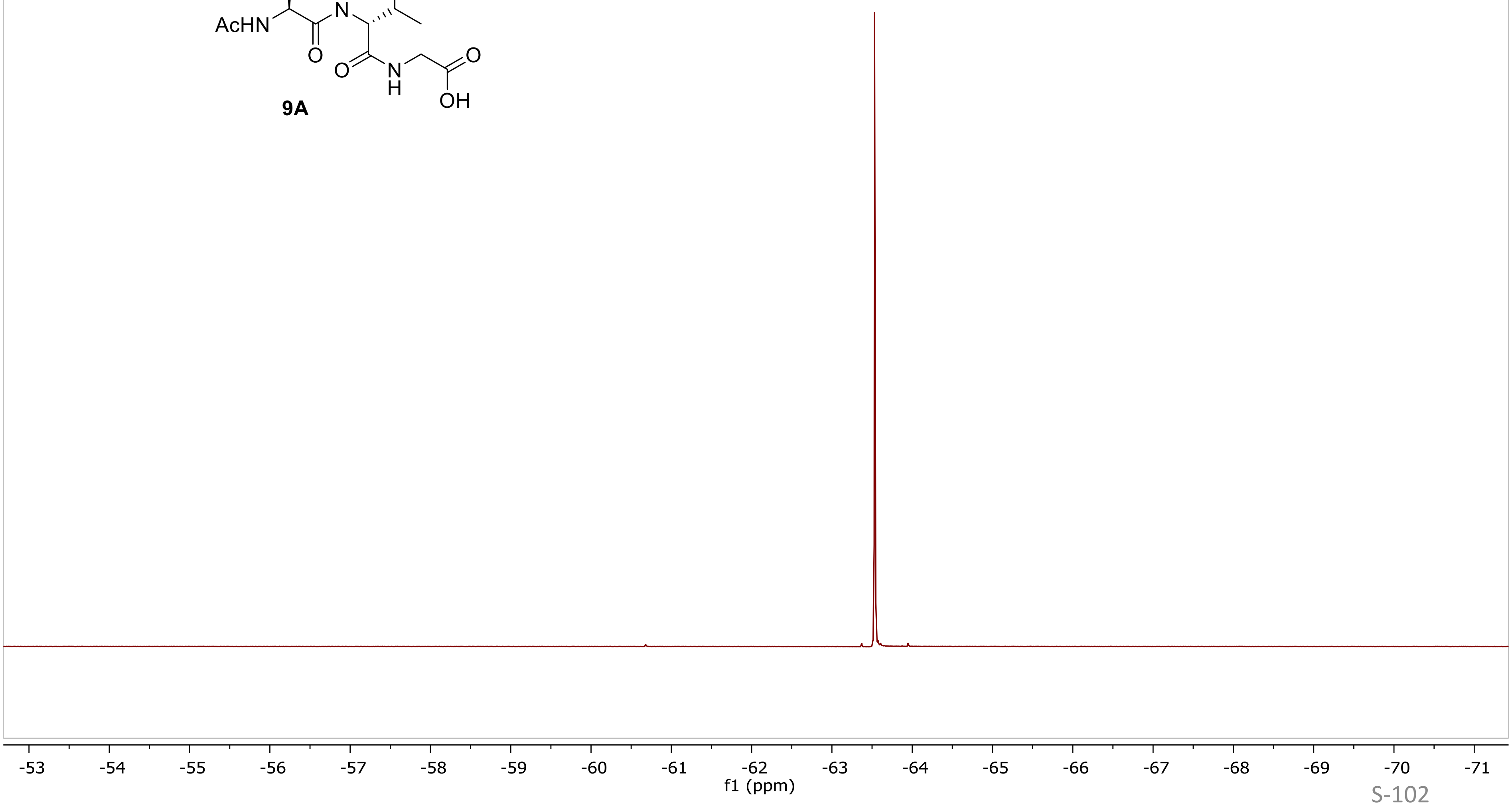

^{1}H NMR (500 MHz, Methanol-d4)

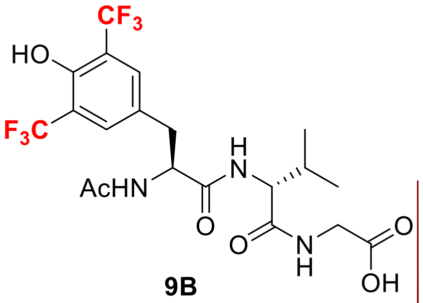

9B

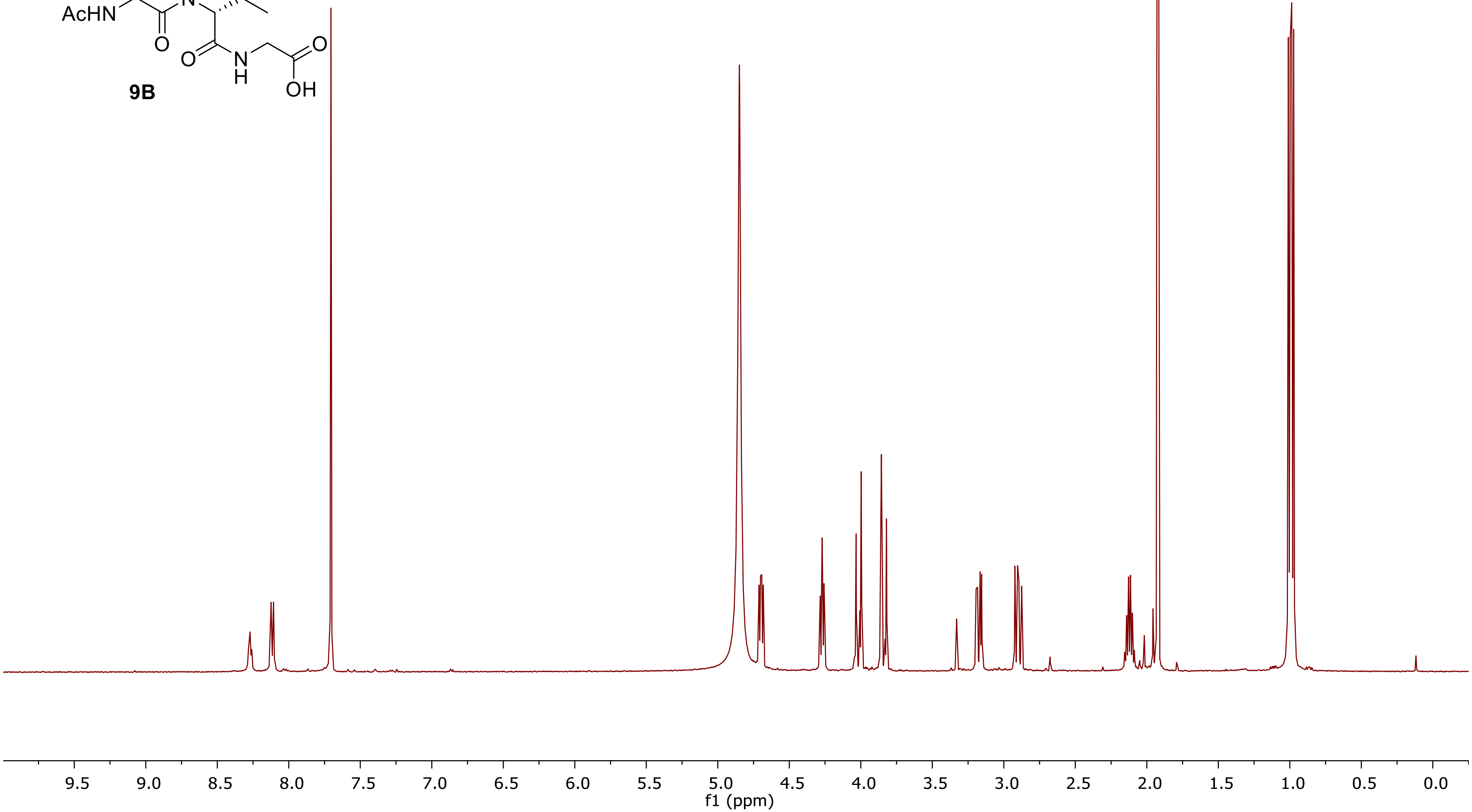

^{13}C NMR (125 MHz, Methanol-d_4)

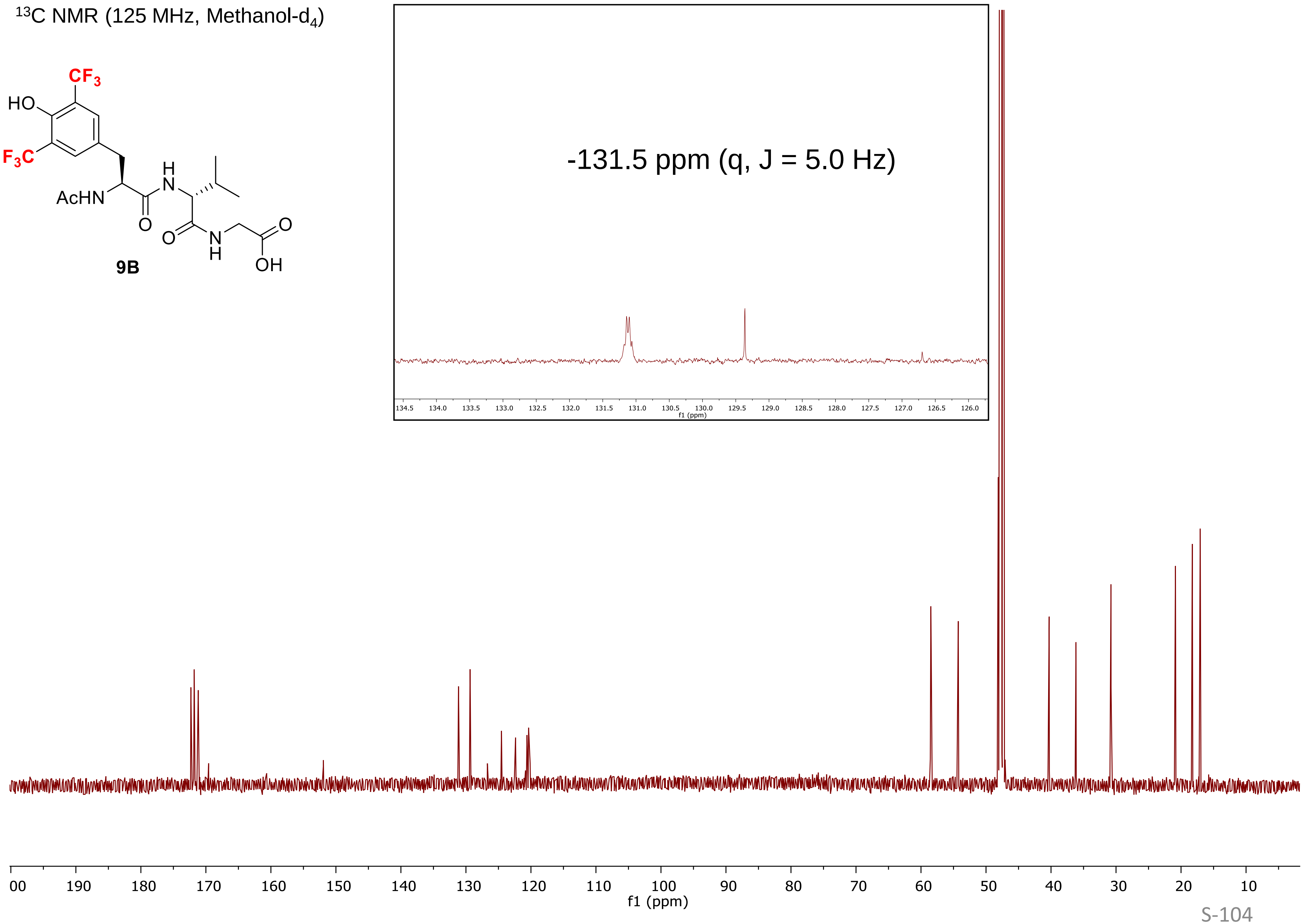

^{19}F NMR (471 MHz, Methanol-d_4)

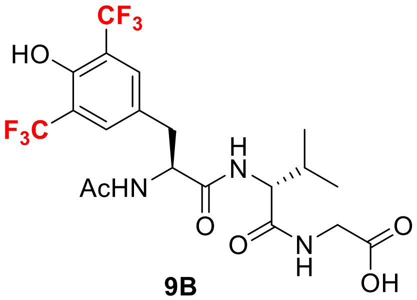

9B

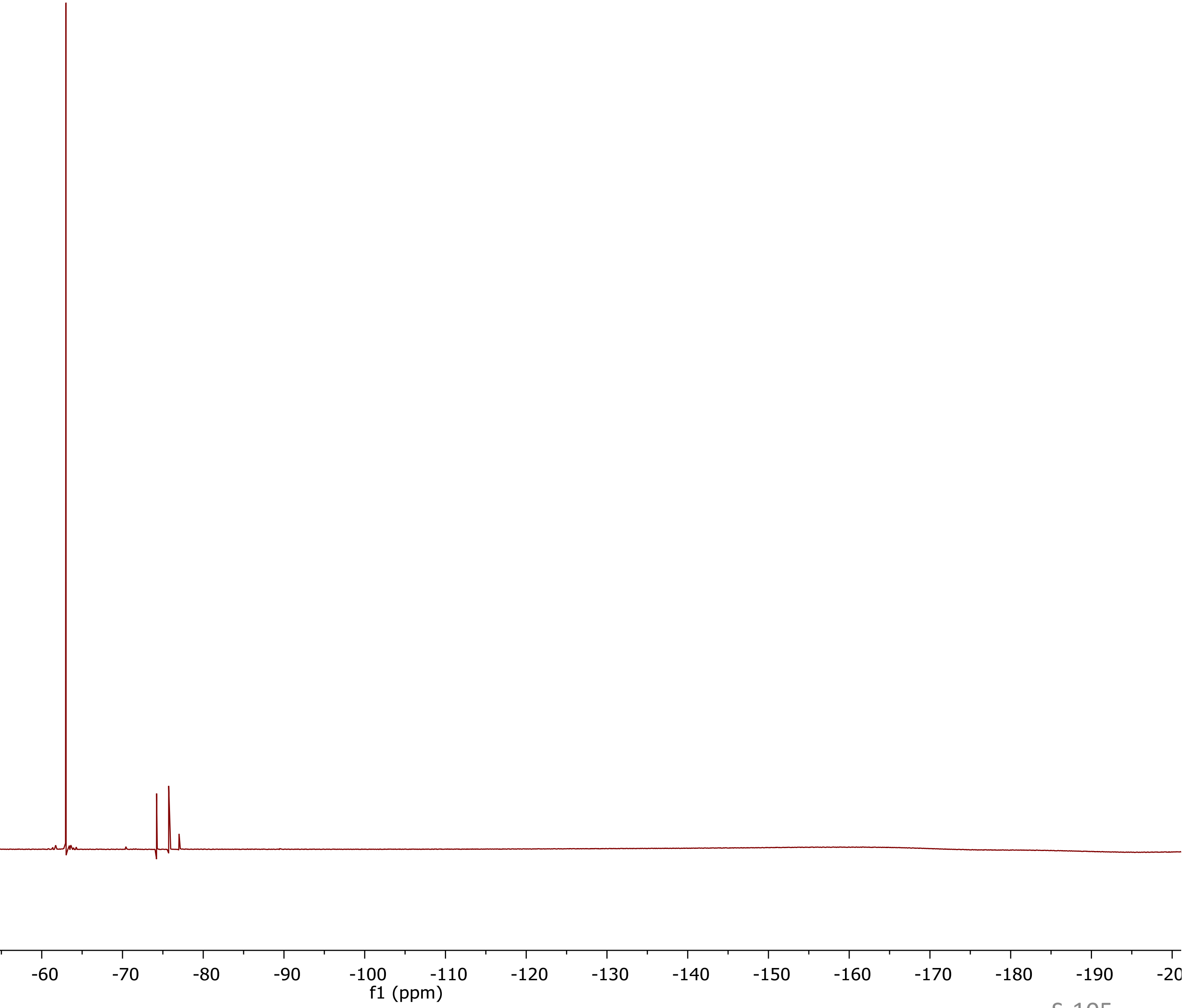

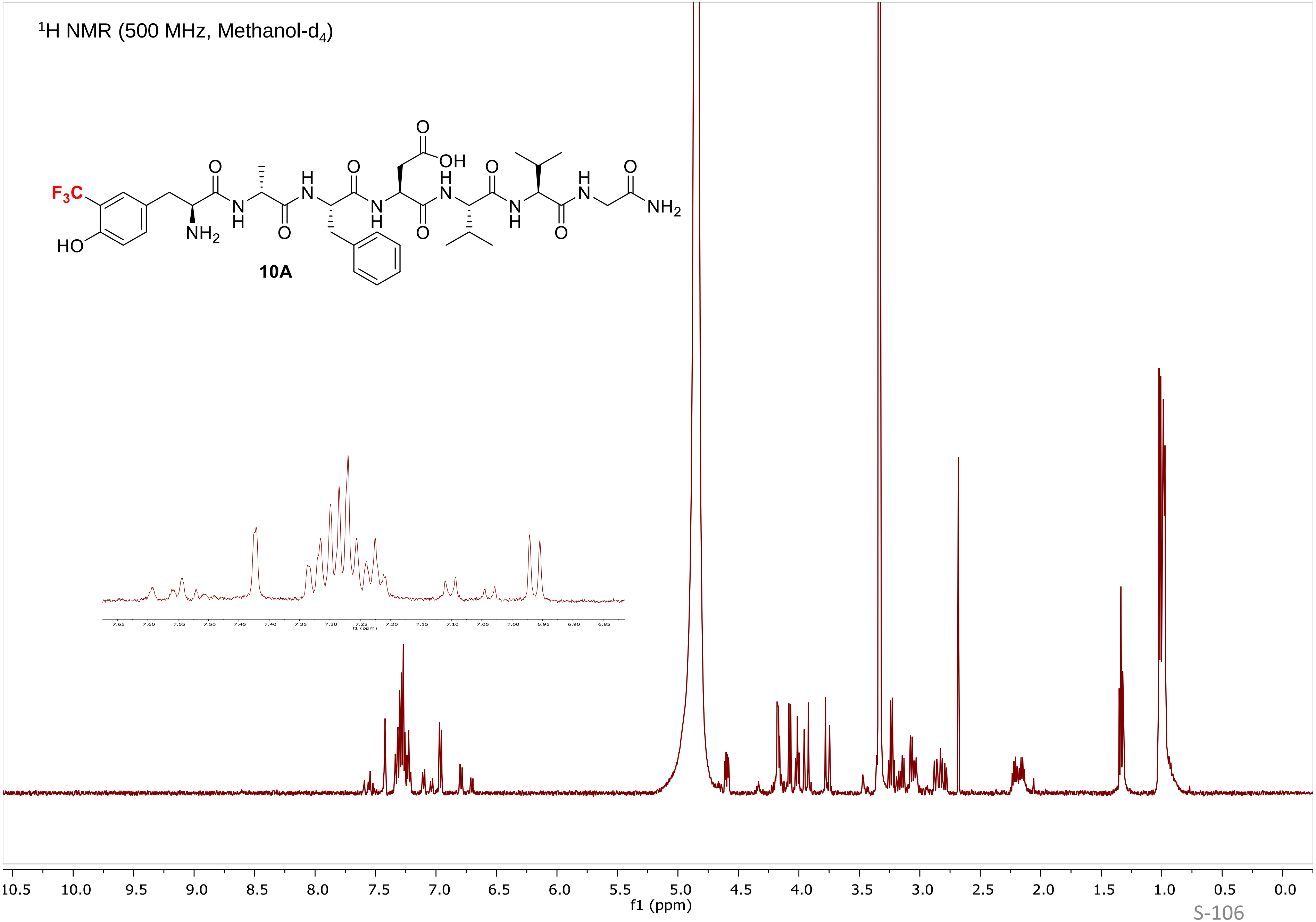

1H NMR (500 MHz, Methanol-d4)
F3C
HO
NH2
10A
OH
NH2
f1 (ppm)
10.5 10.0 9.5 9.0 8.5 8.0 7.5 7.0 6.5 6.0 5.5 5.0 4.5 4.0 3.5 3.0 2.5 2.0 1.5 1.0 0.5 0.0

^{19}F NMR (471 MHz, Methanol-d_4)

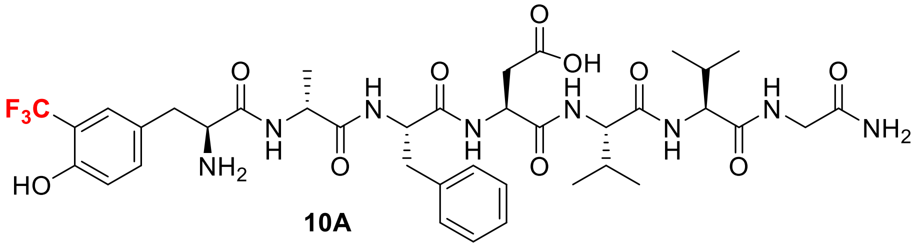

10A

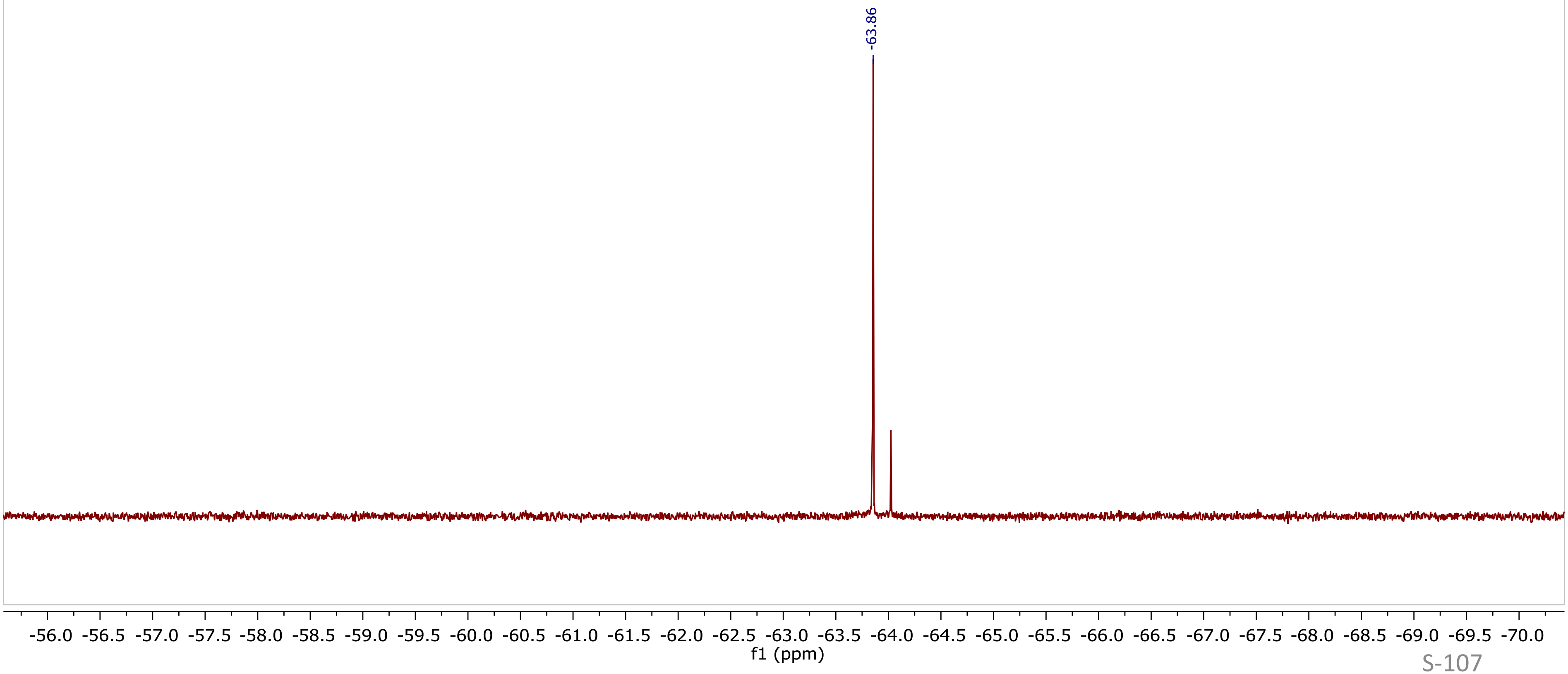

Overlay of ^{1}HNMR of **10A** and compound **10**

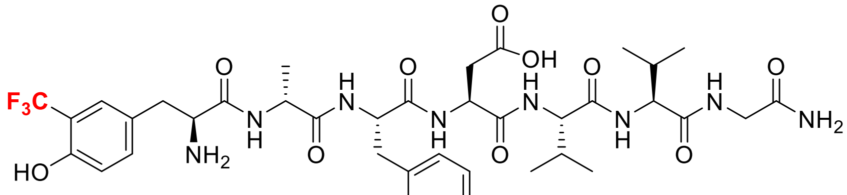

10A

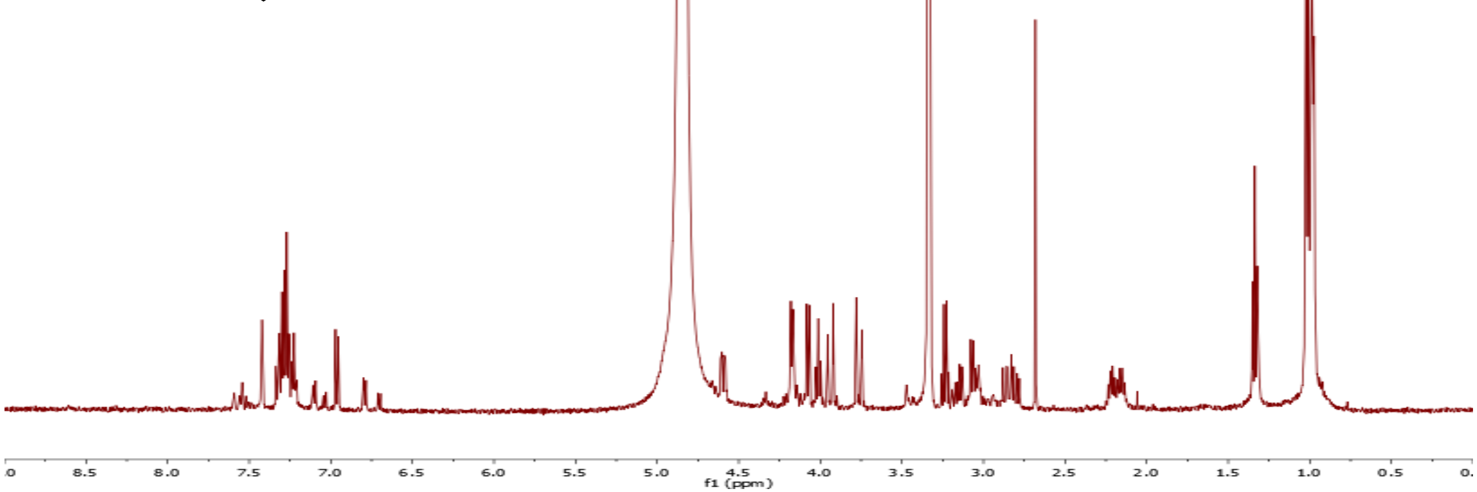

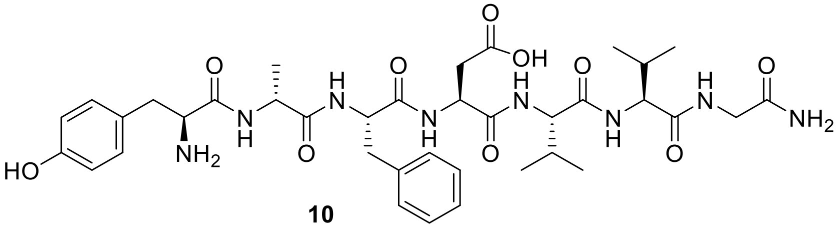

10

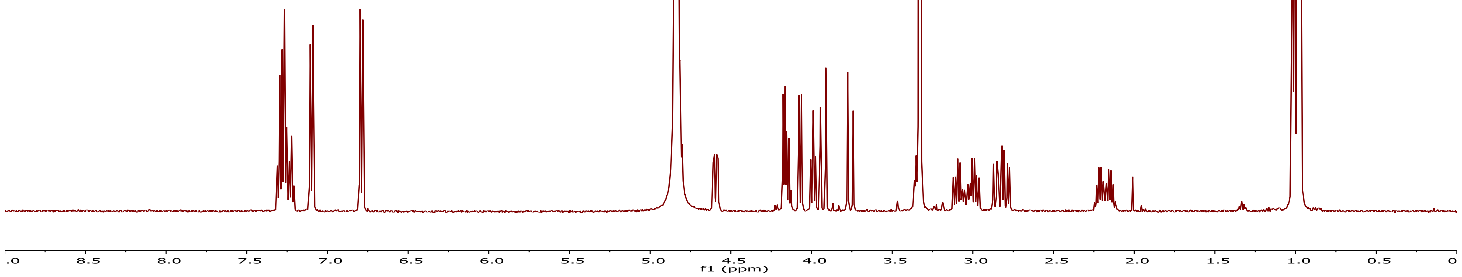

COSY (**10A**)

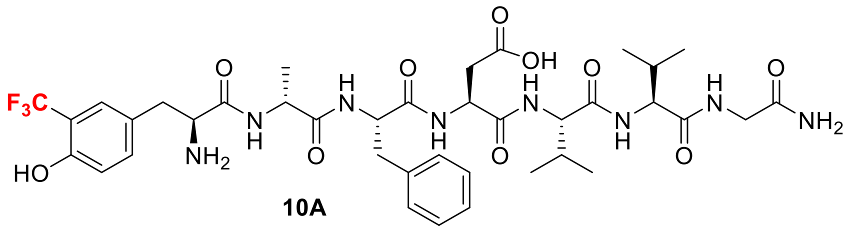

10A

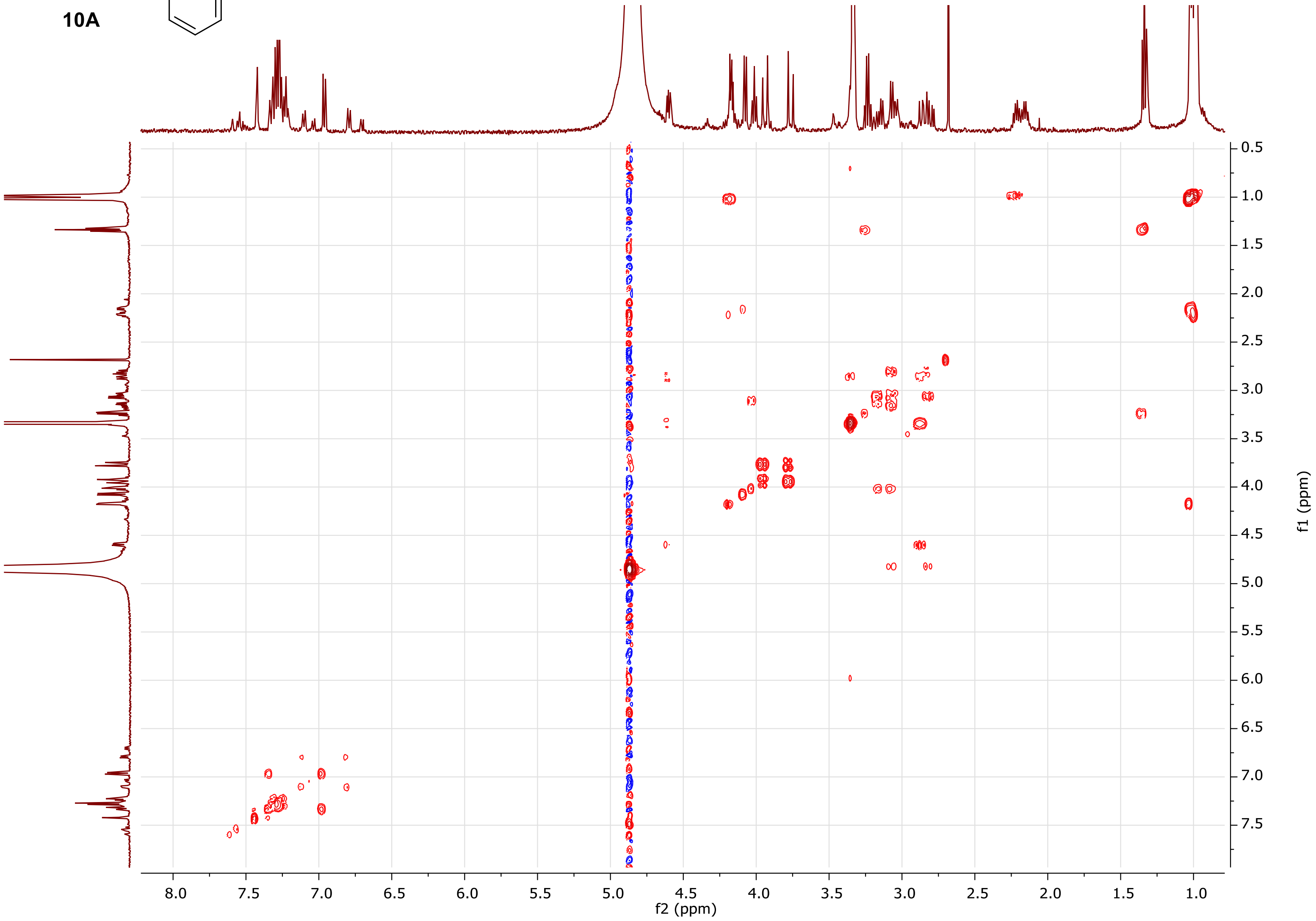

COSY, aromatic (**10A**)

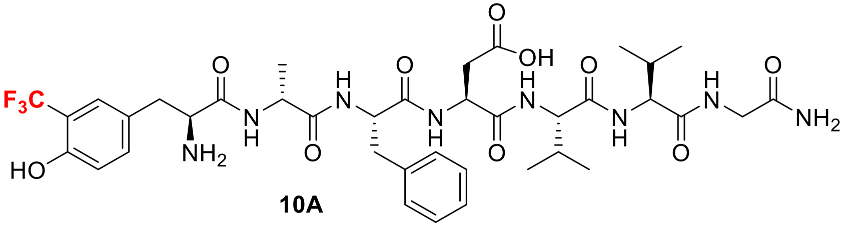

10A

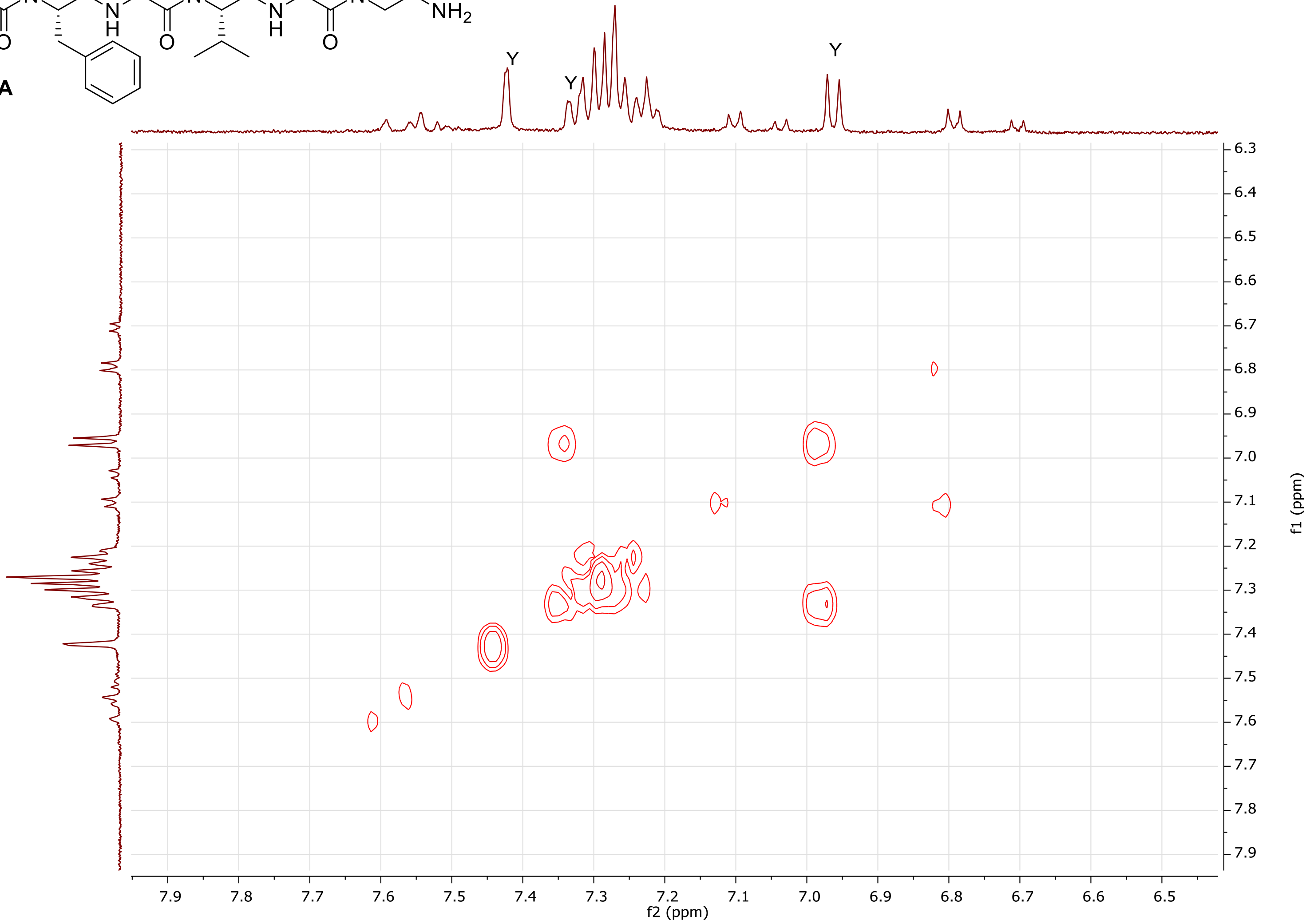

^{1}H NMR (600 MHz, Methanol-d_4)

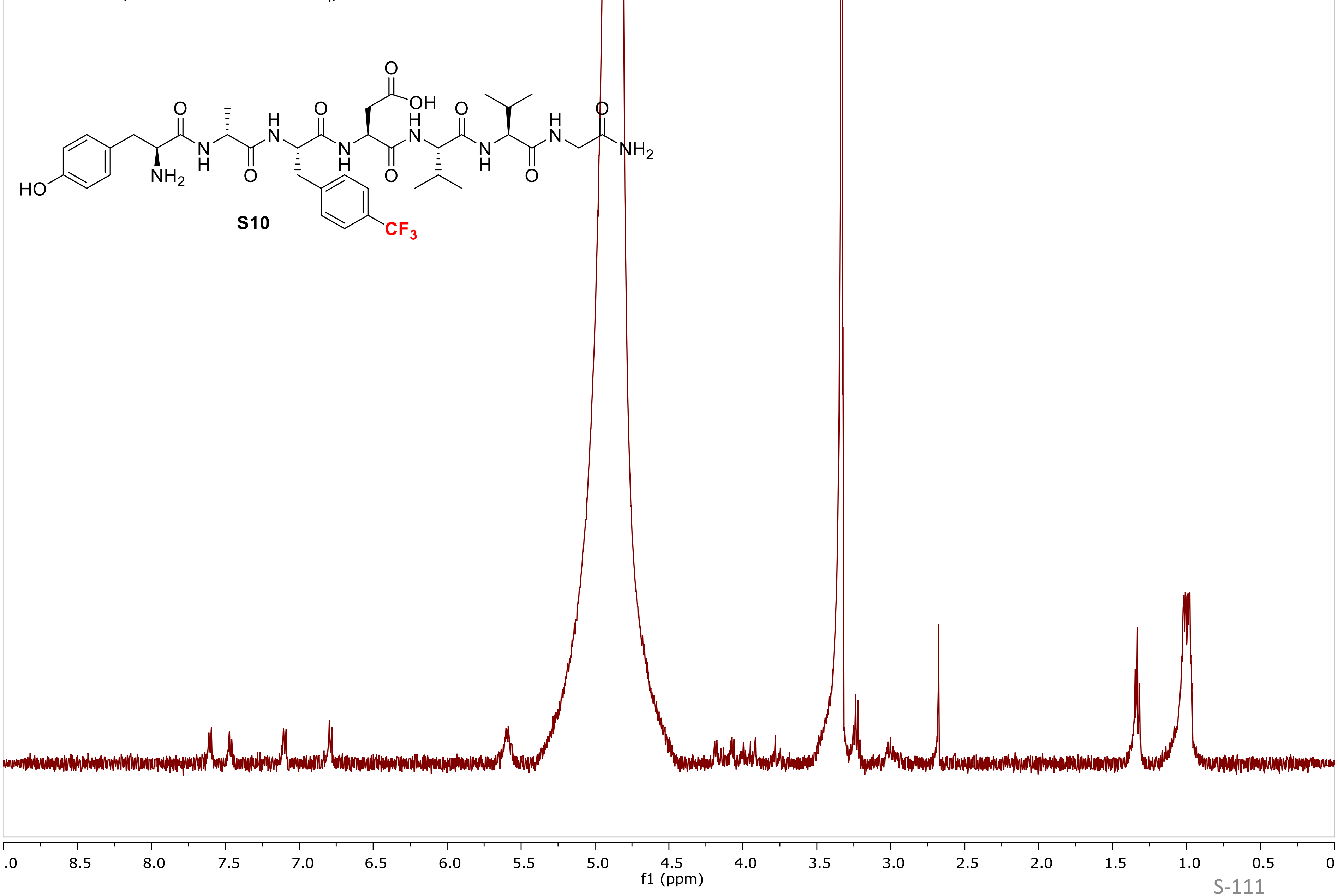

^{19}F NMR (471 MHz, Methanol-d_4)

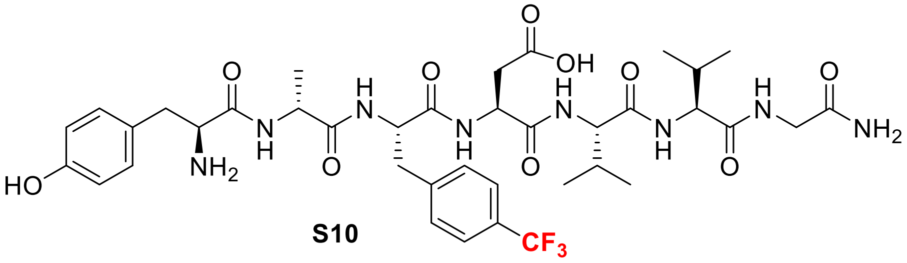

S10

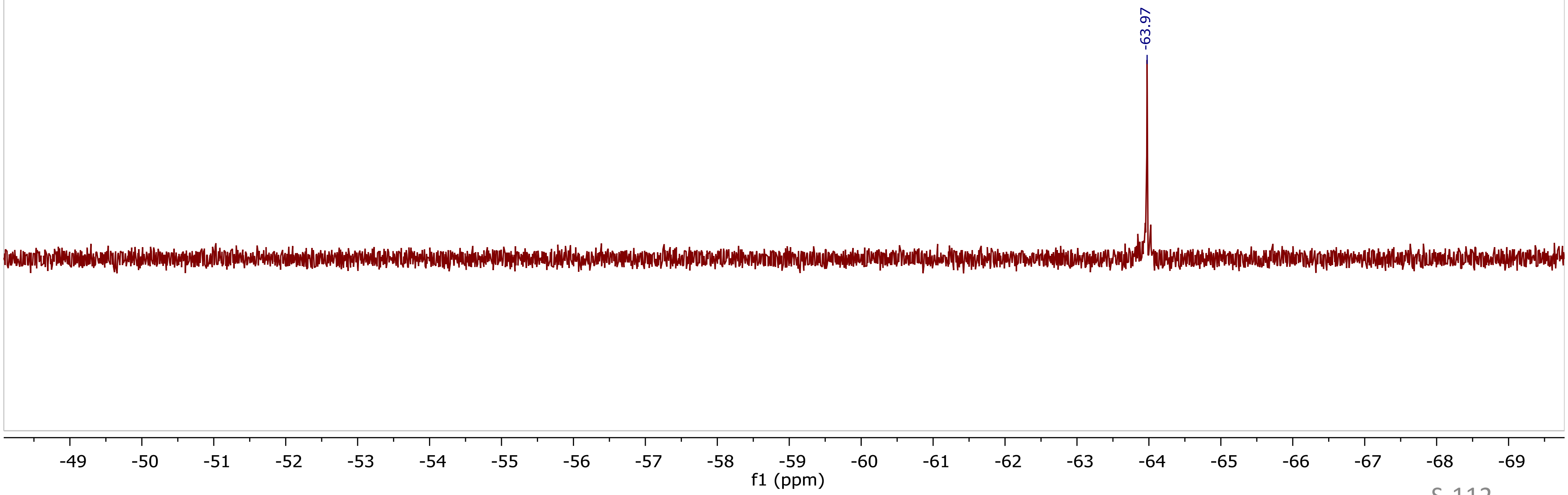

Overlay of ^{1}HNMR of **S10** and compound **10**

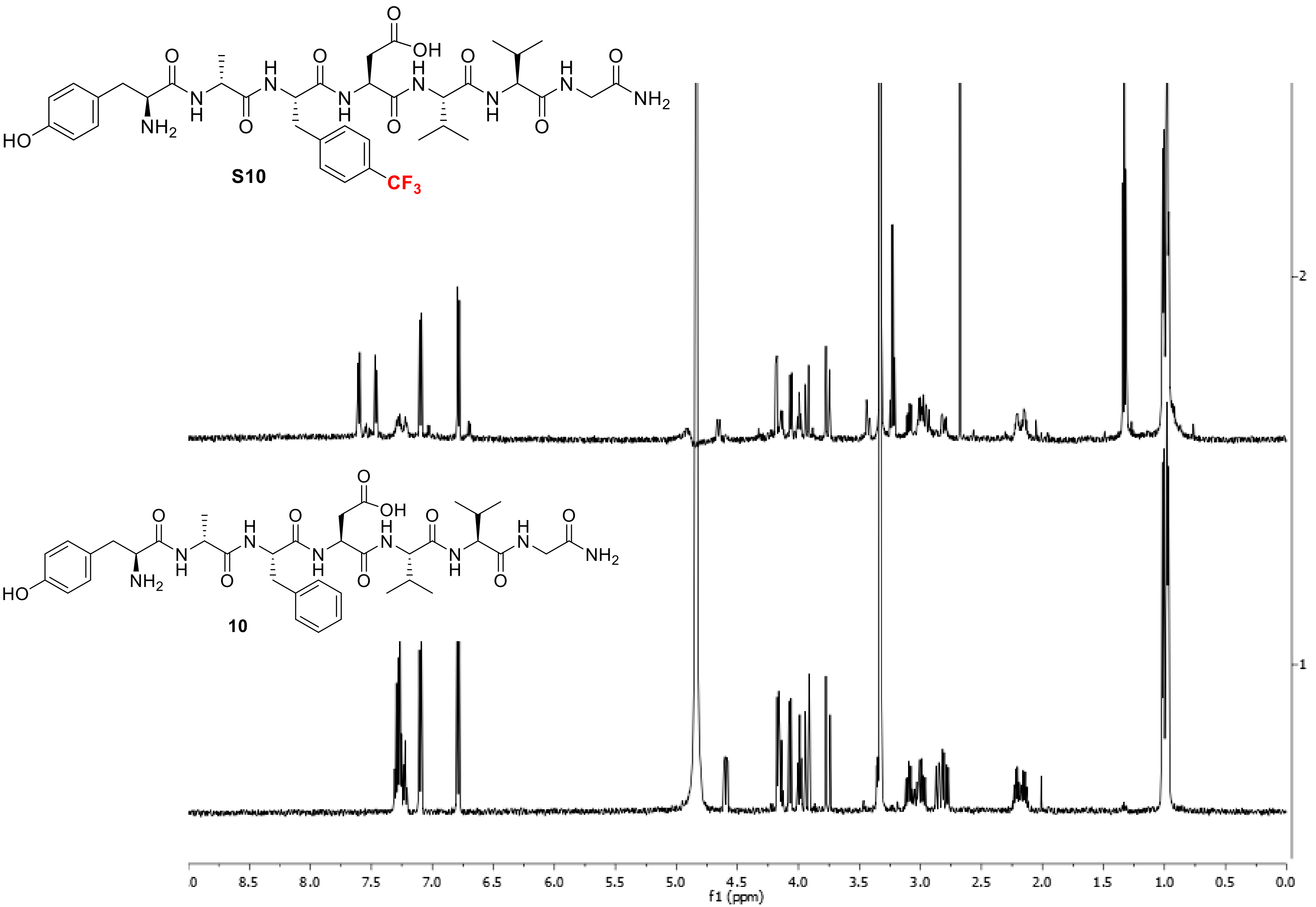

COSY, aromatic (**S10**)

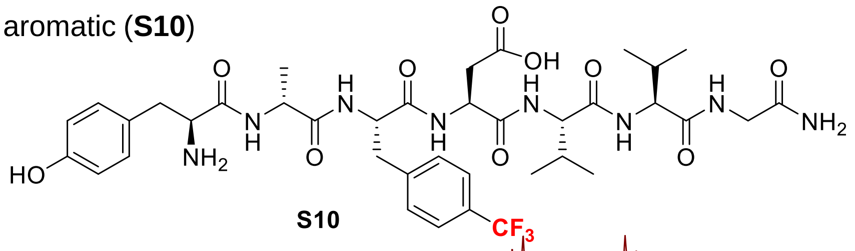

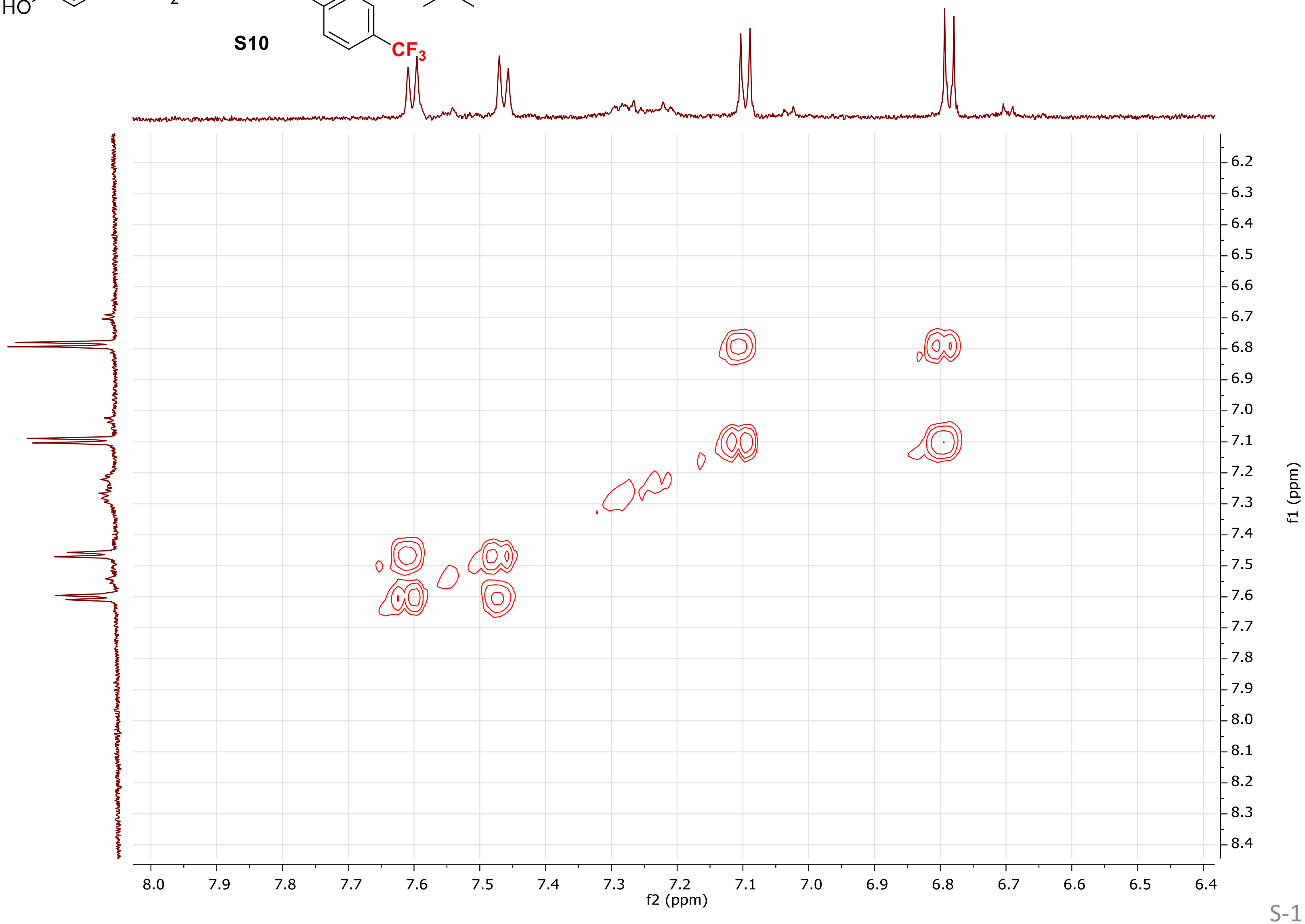

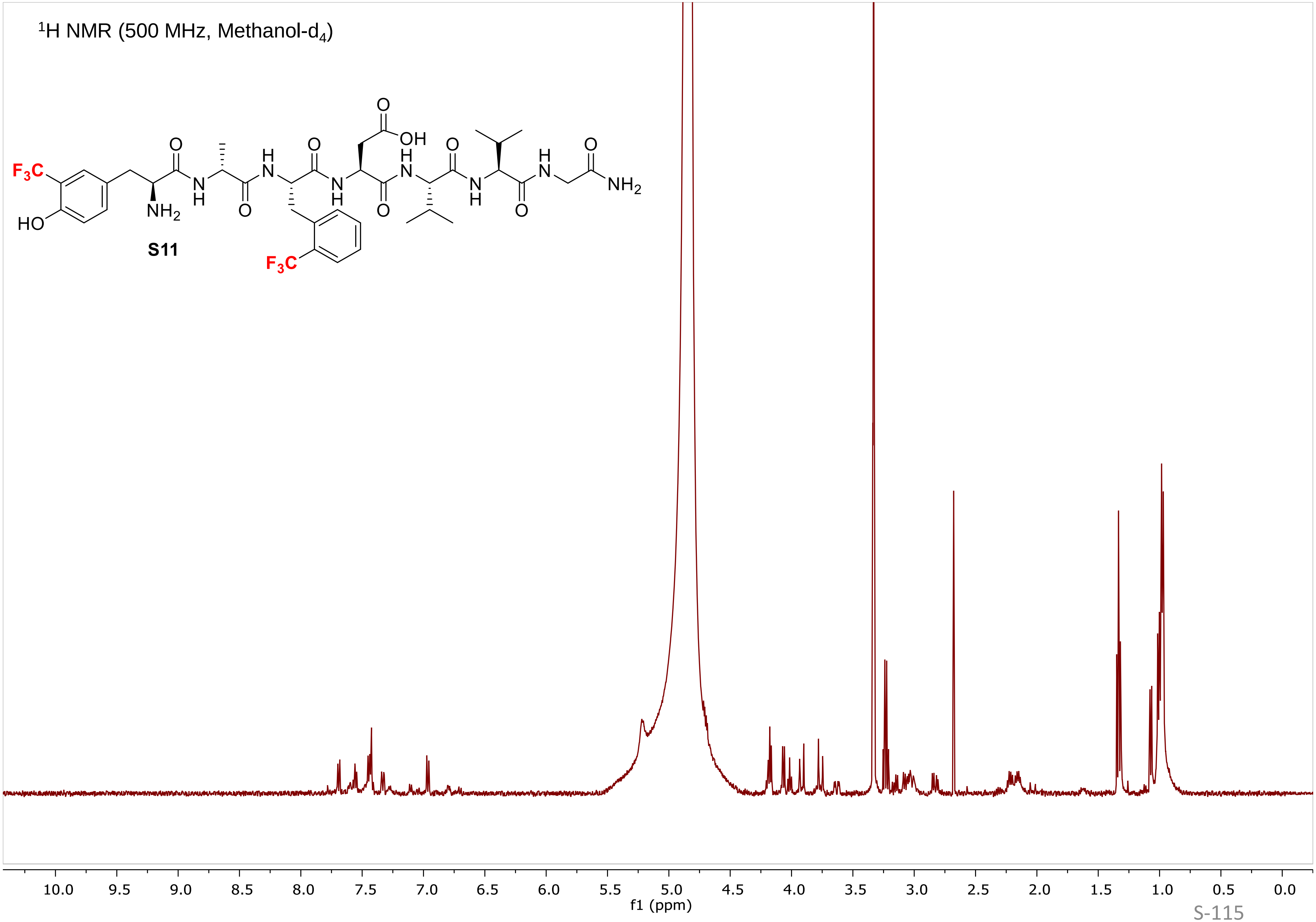
1H NMR (500 MHz, Methanol-d4)
S11
10.0 9.5 9.0 8.5 8.0 7.5 7.0 6.5 6.0 5.5 5.0 4.5 4.0 3.5 3.0 2.5 2.0 1.5 1.0 0.5 0.0
f1 (ppm)

^{19}F NMR (471 MHz, Methanol-d_4)

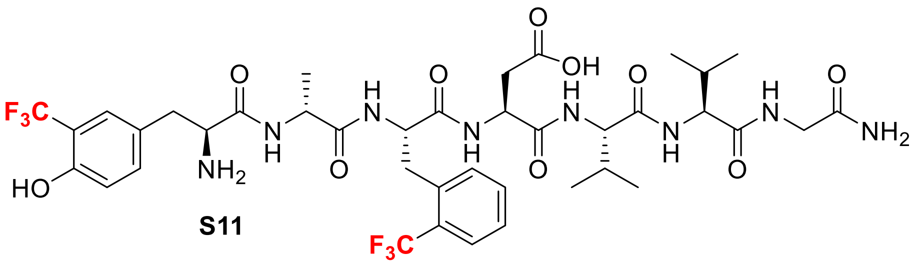

S11

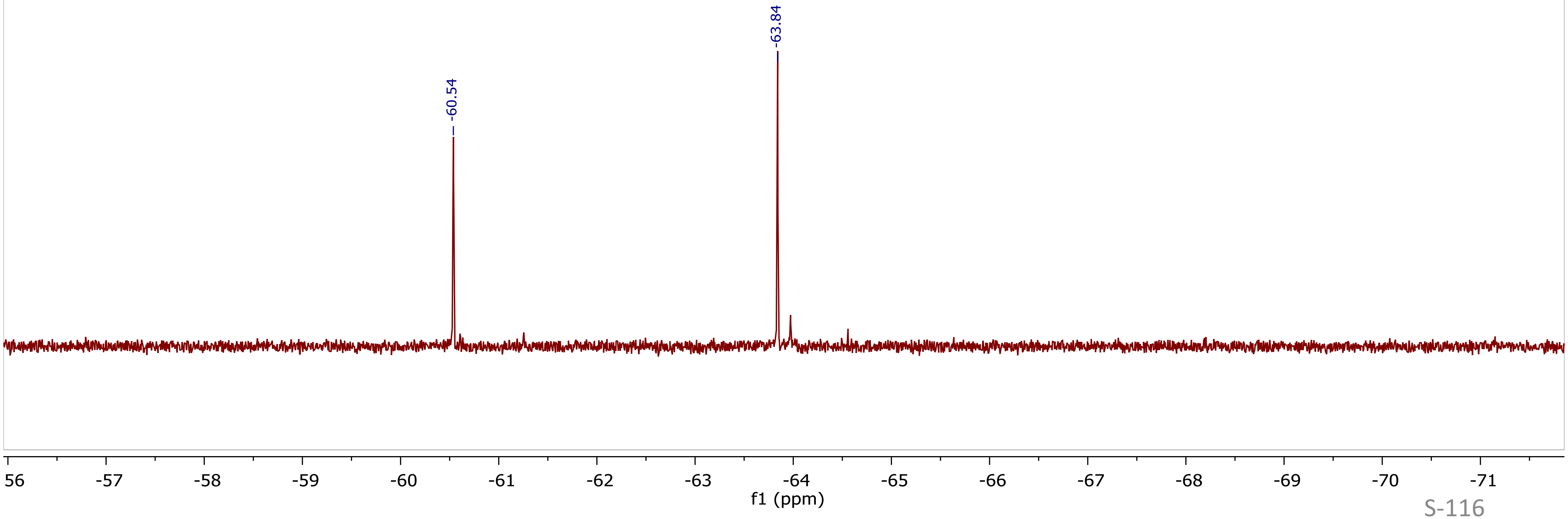

Overlay of ^{1}HNMR of **S11** and compound **10**

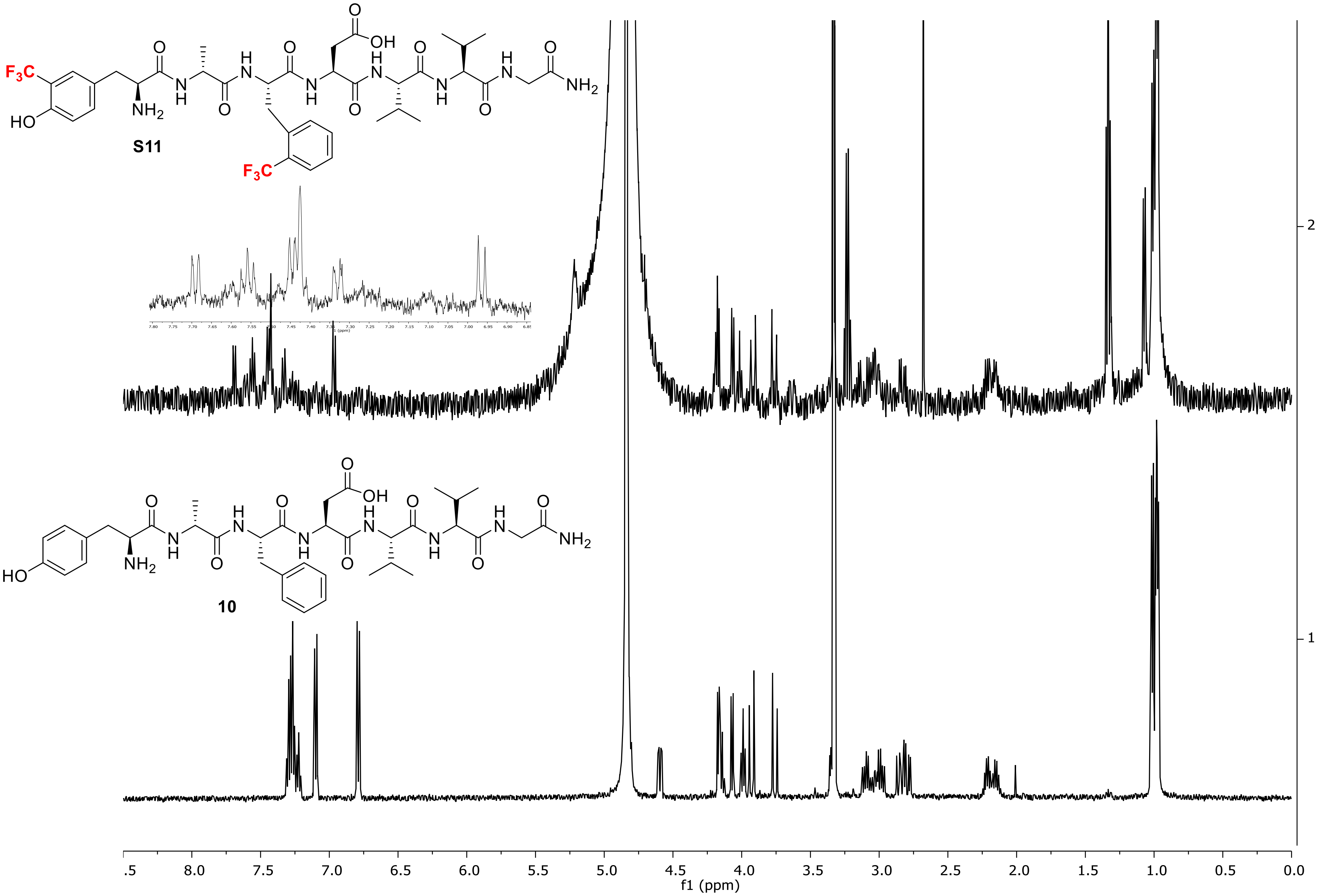

COSY, aromatic (**S11**)

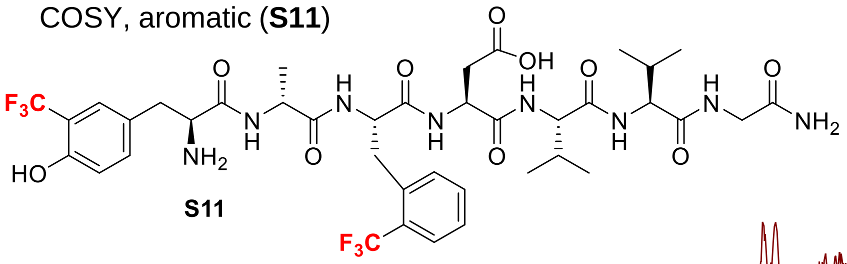

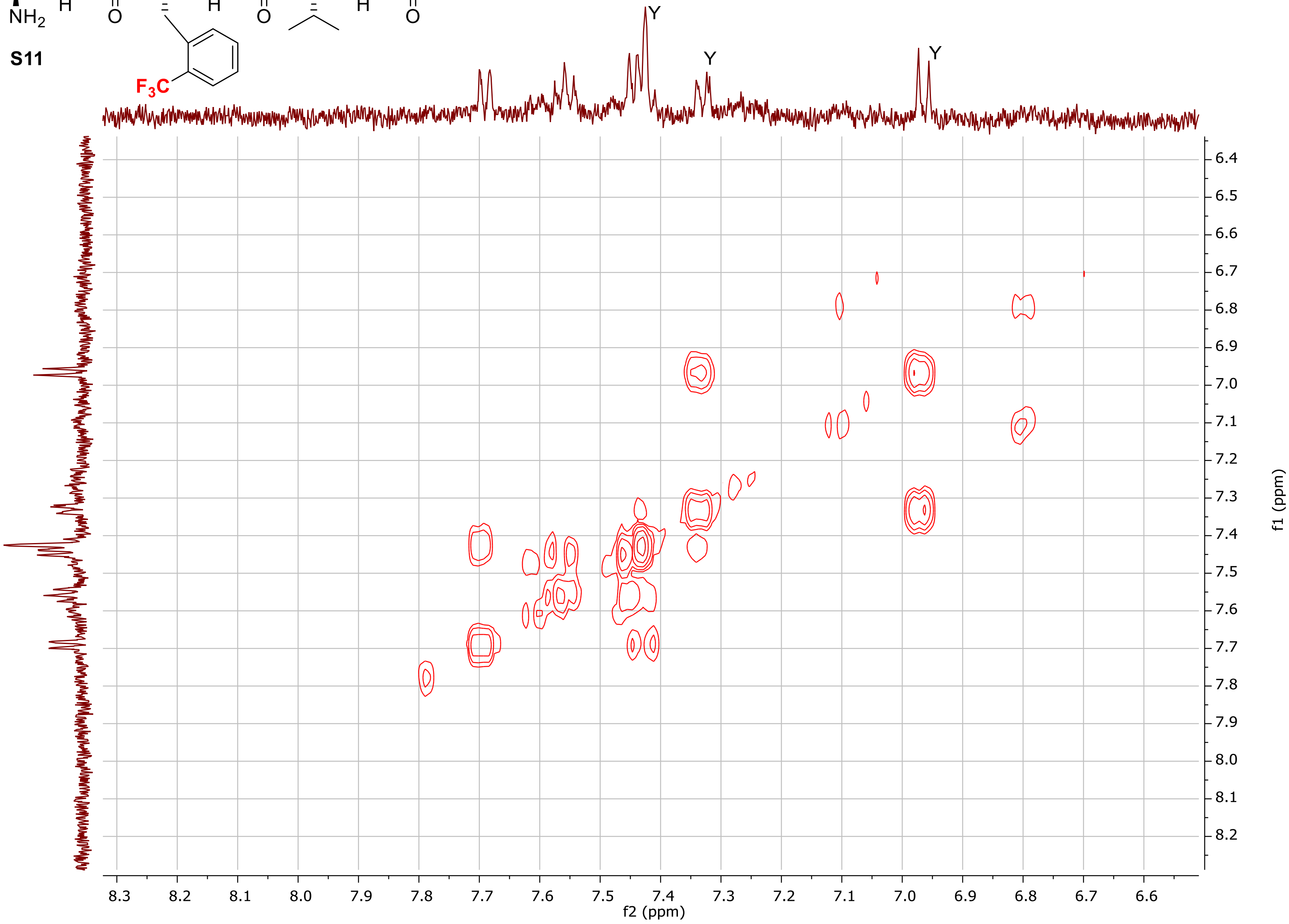

^1H NMR (500 MHz, Methanol-d_4)

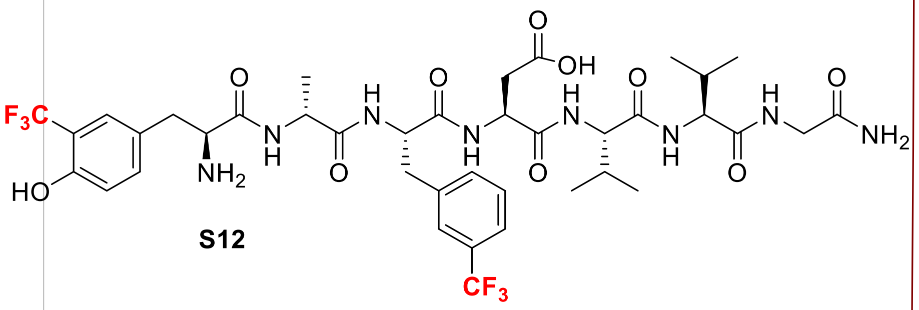

S12

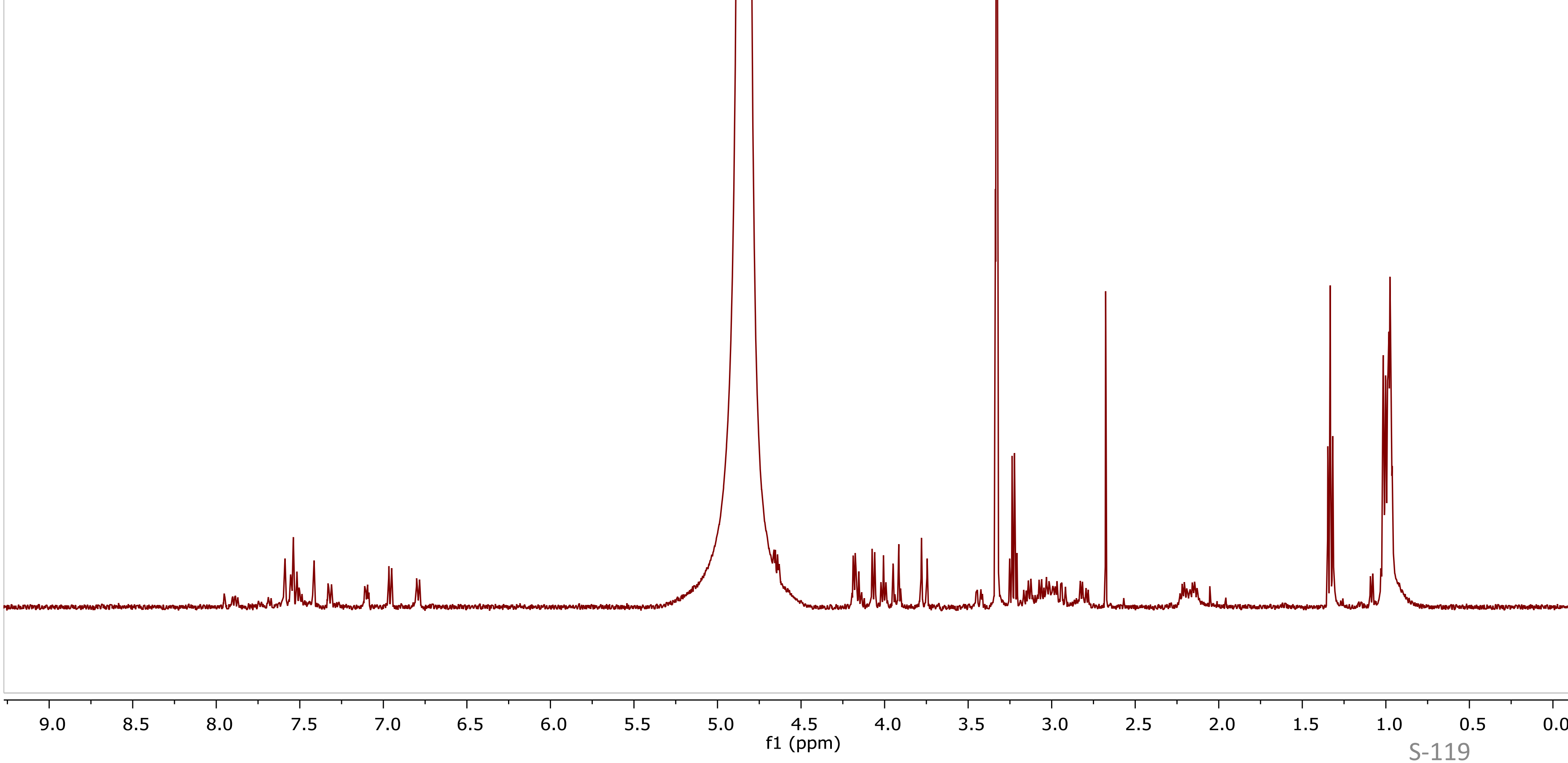

^{19}F NMR (471 MHz, Methanol-d_4)

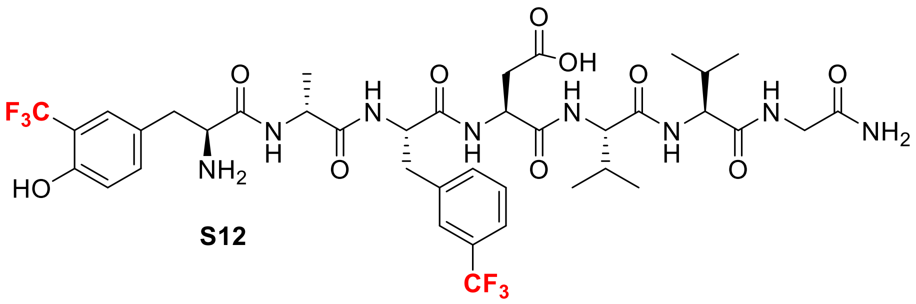

S12

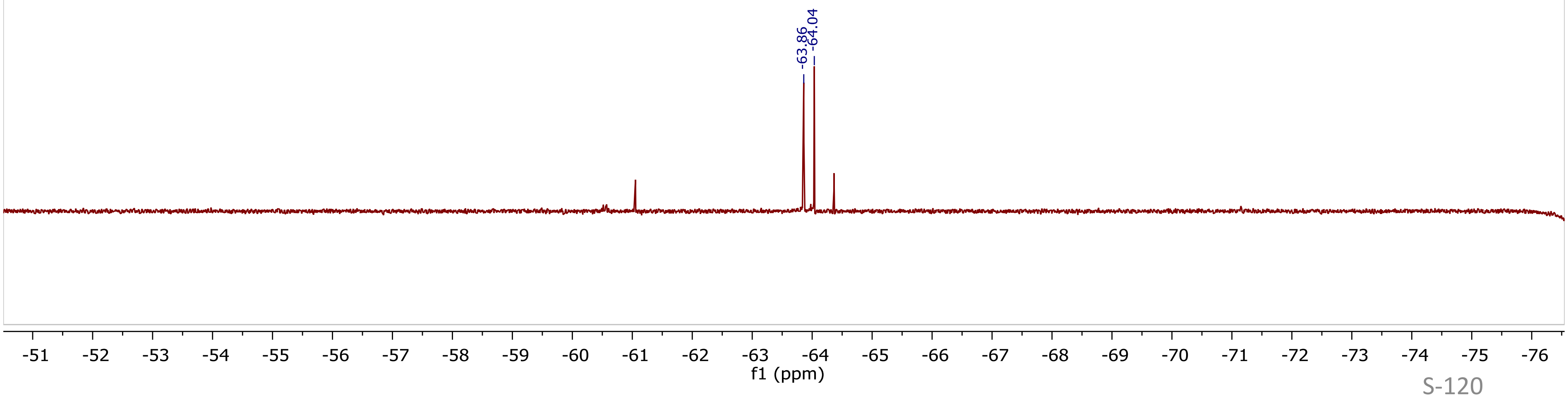

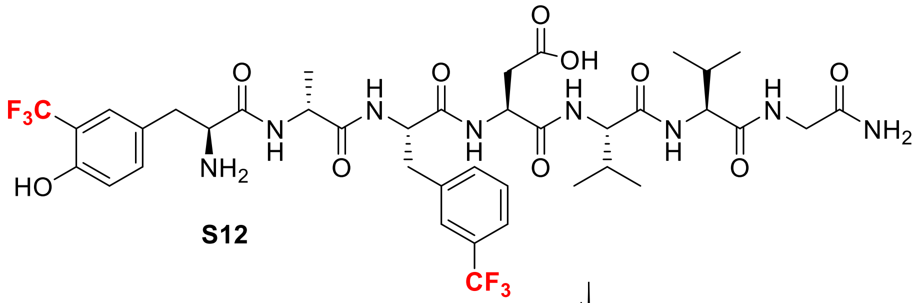

S12

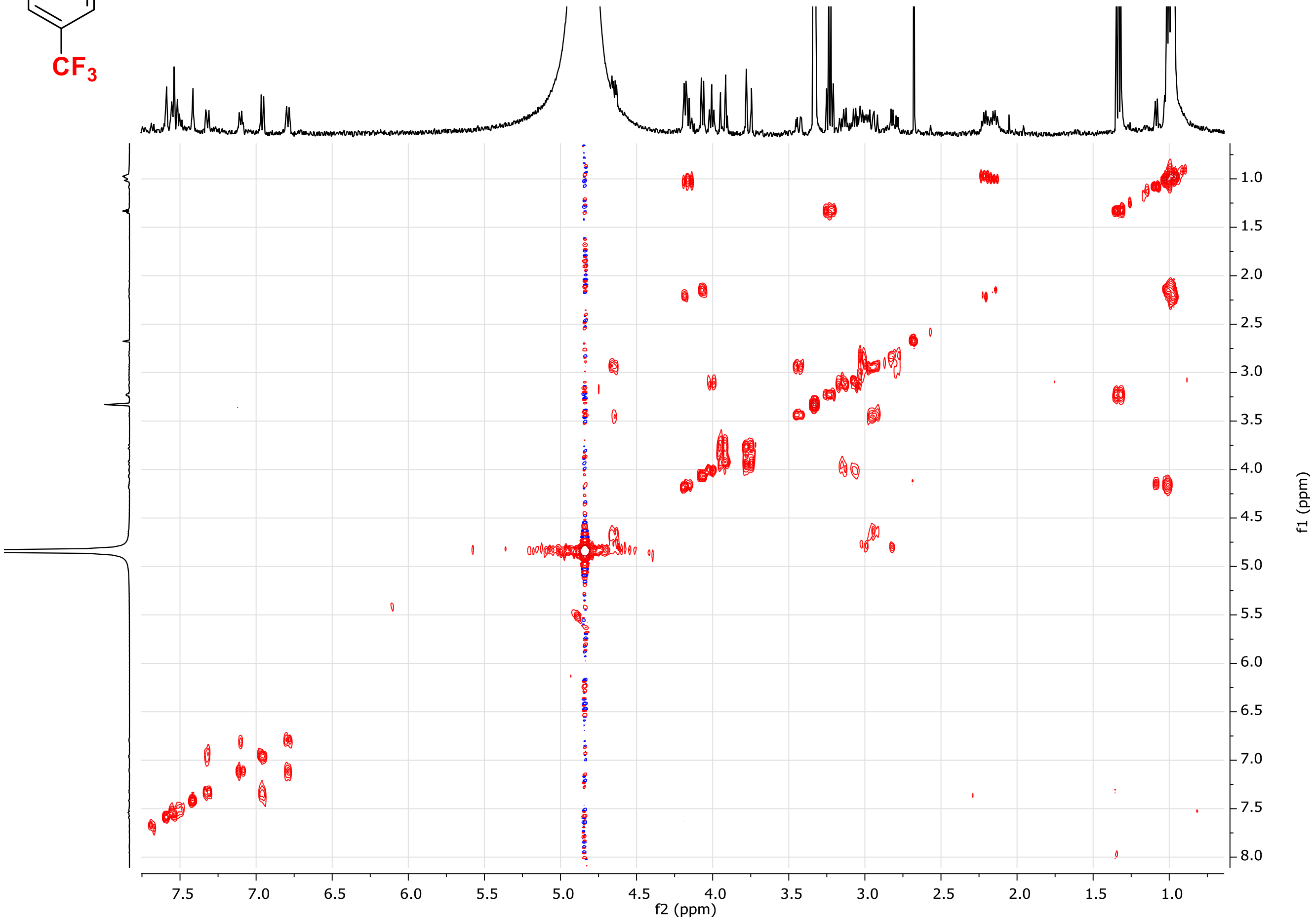

^{1}H NMR (500 MHz, Methanol-d4)

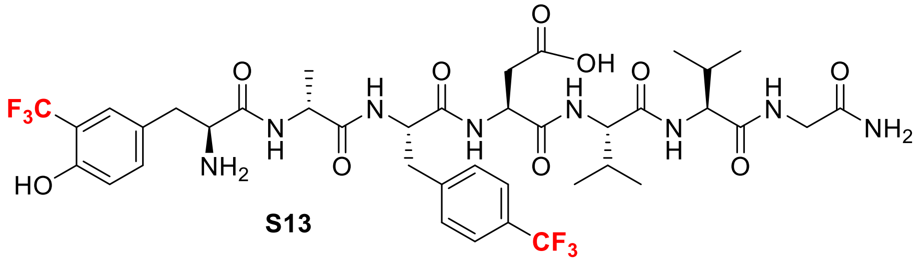
S13

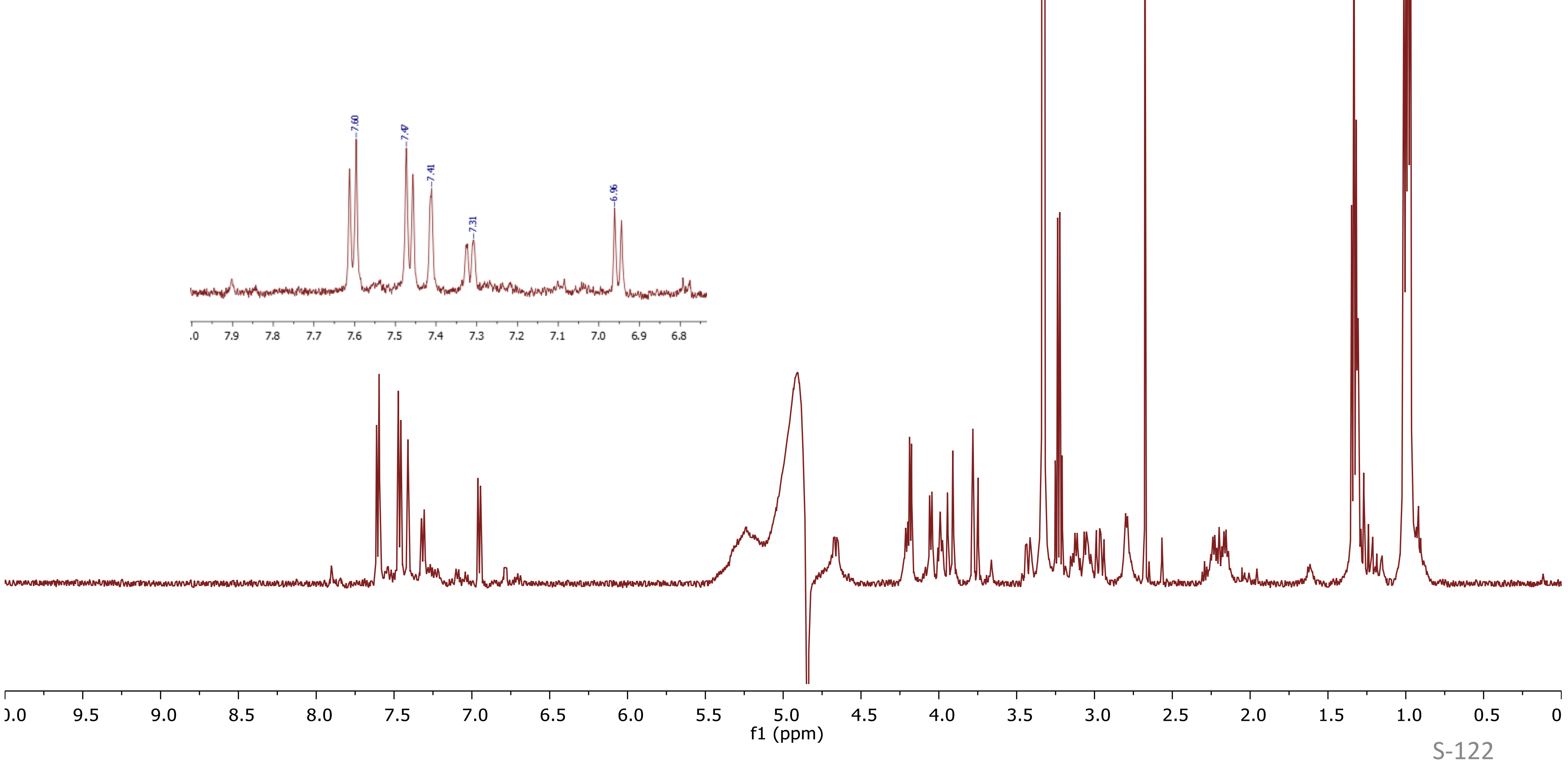

^{19}F NMR (471 MHz, Methanol-d_4)

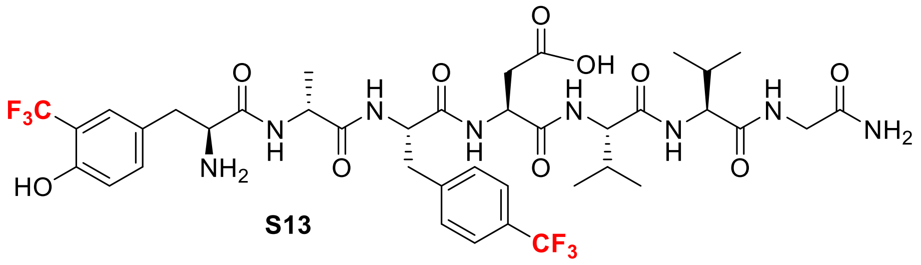

S13

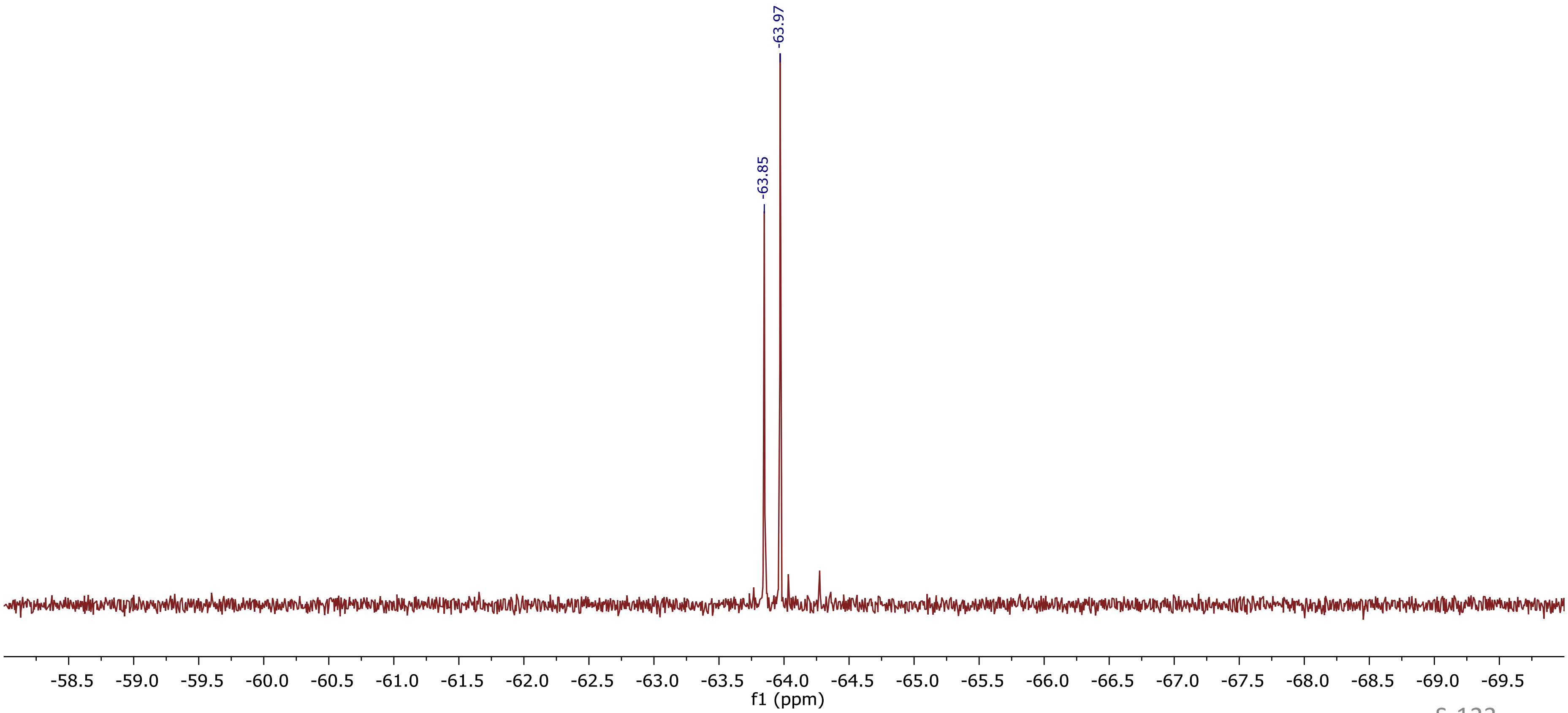

Overlay of ^{1}HNMR of **S13** and compound **10**

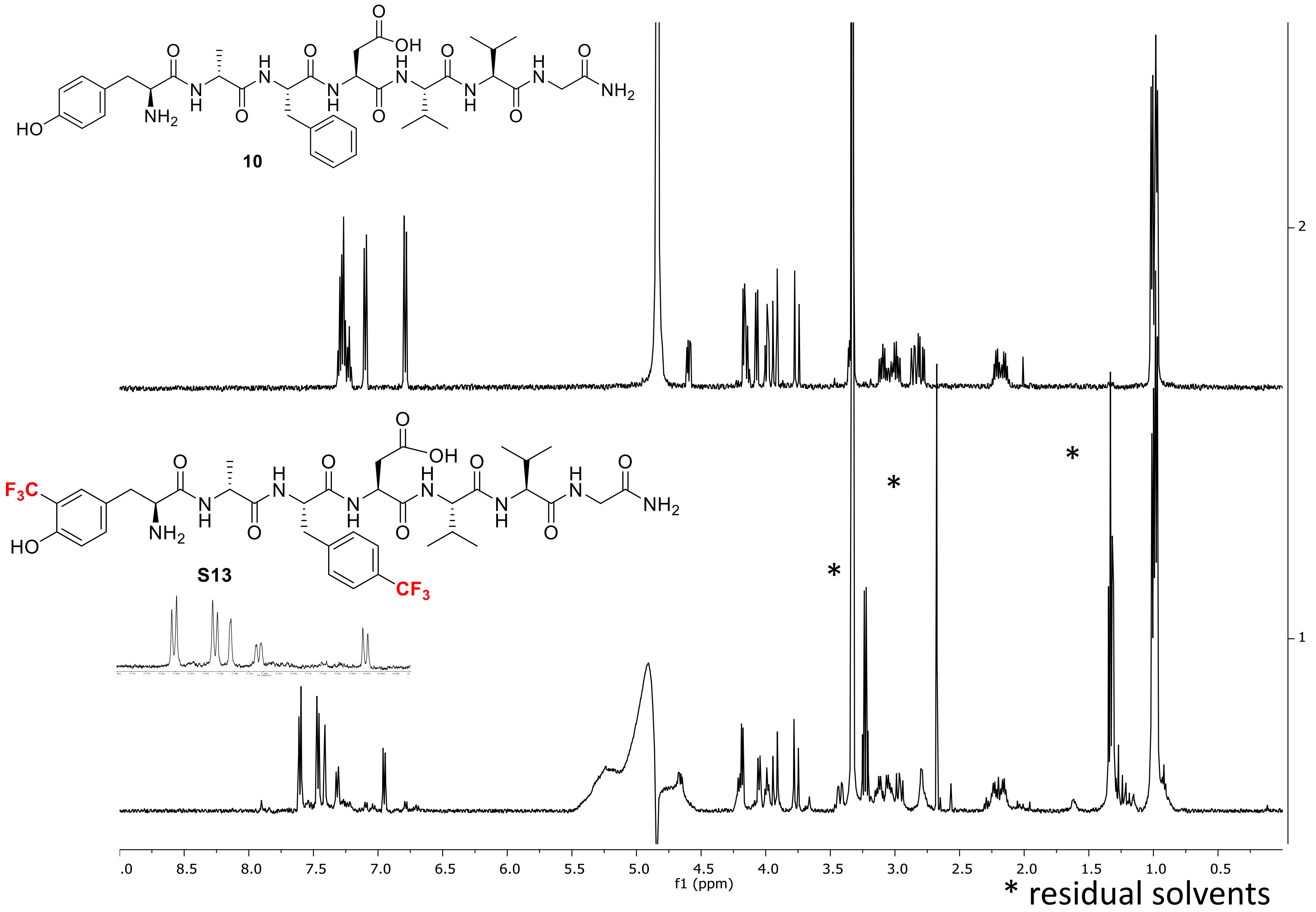

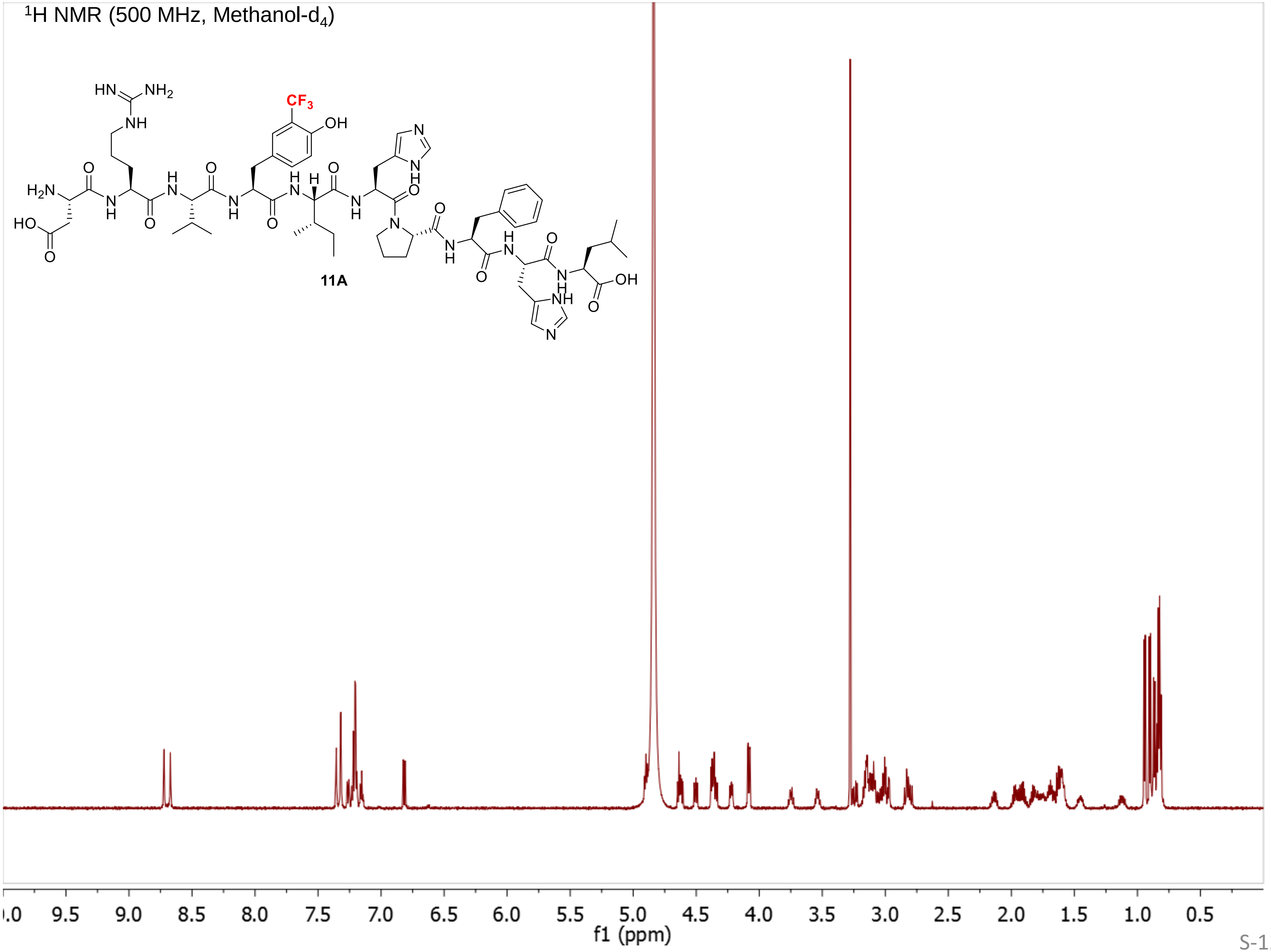
^{1}H NMR (500 MHz, Methanol-d$_4$)
11A
CF$_3$
f1 (ppm)
9.5 9.0 8.5 8.0 7.5 7.0 6.5 6.0 5.5 5.0 4.5 4.0 3.5 3.0 2.5 2.0 1.5 1.0 0.5

^{13}C NMR (150 MHz, Methanol-d_4)

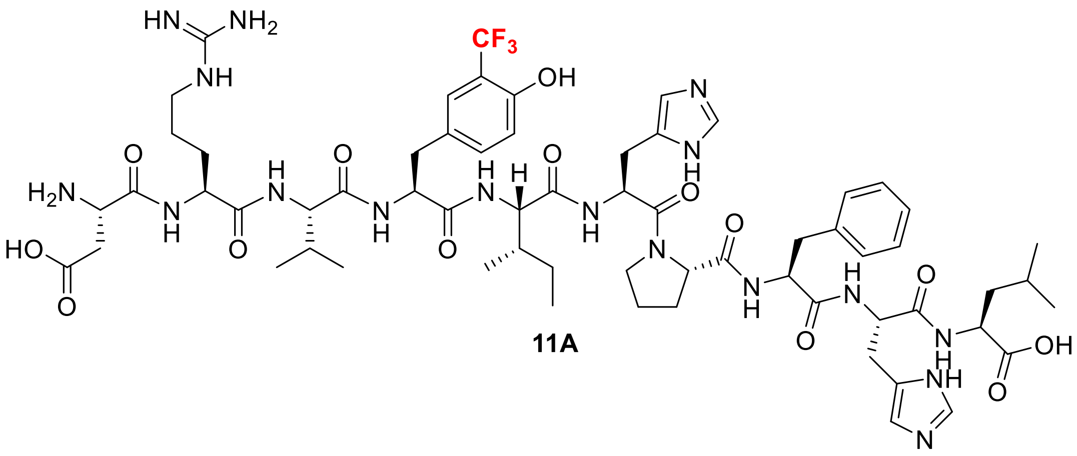

11A

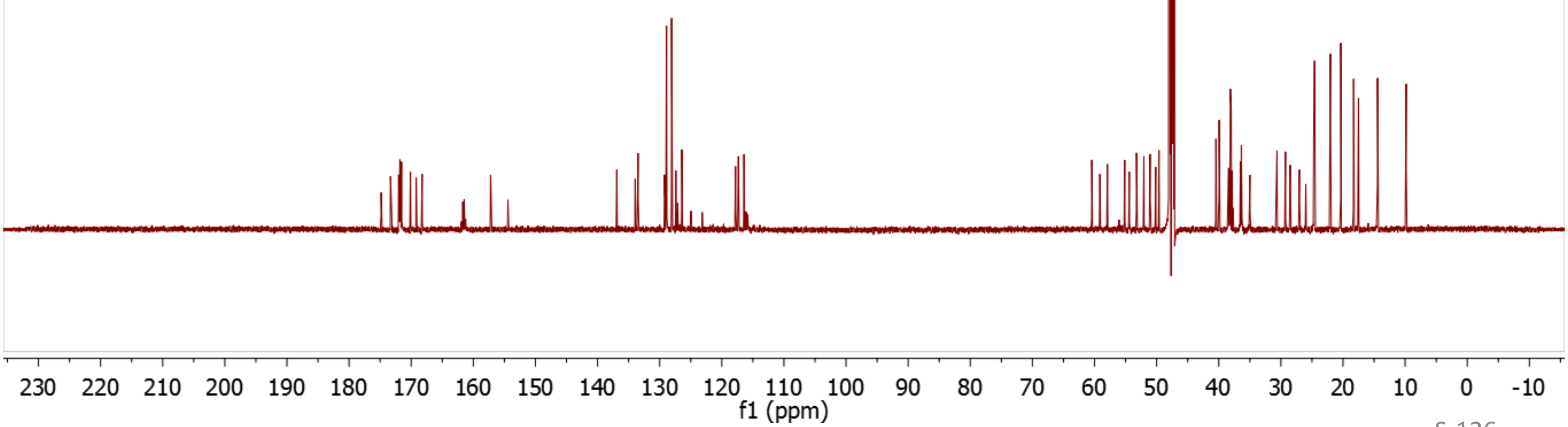

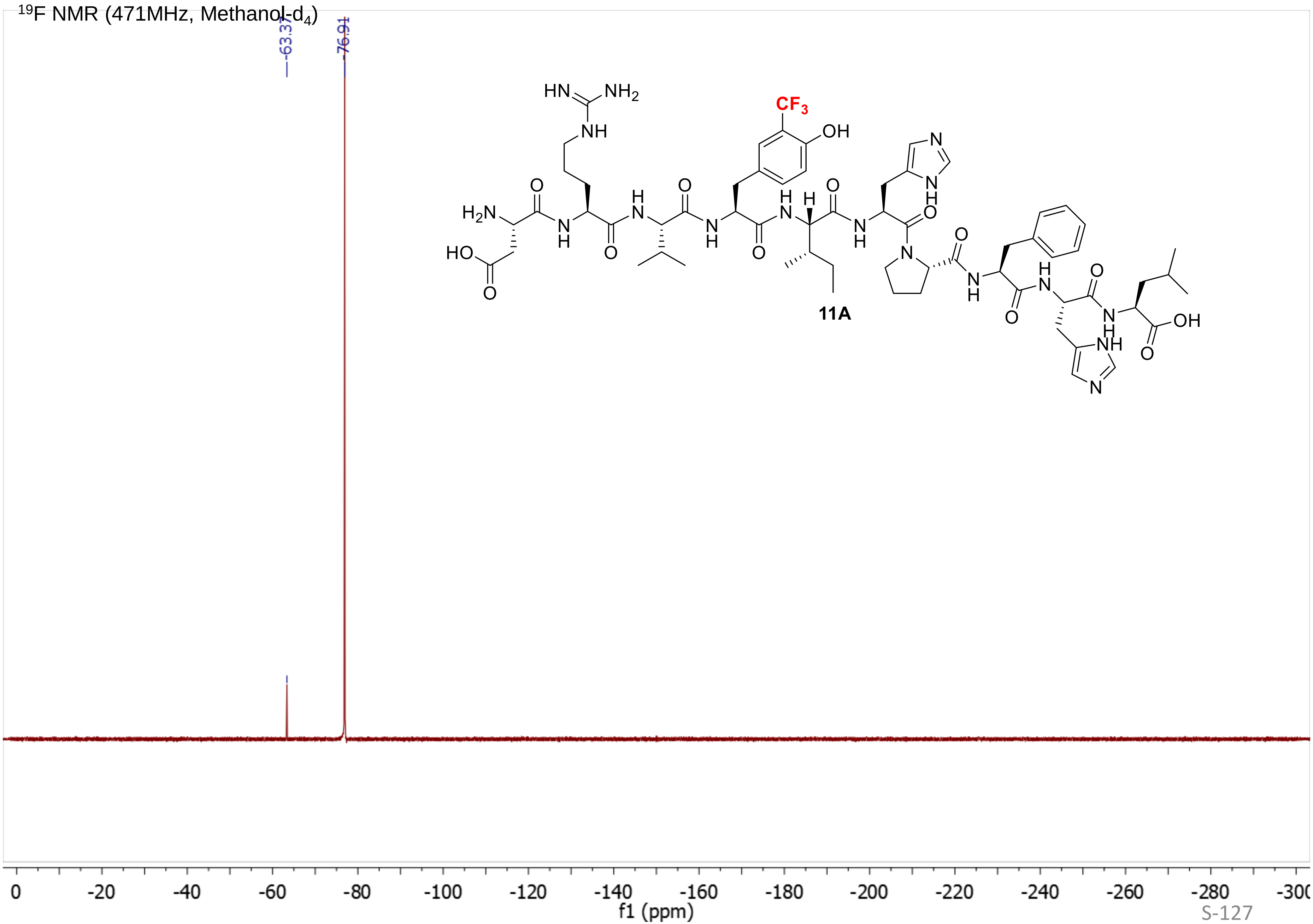

19F NMR (471MHz, Methanol-d4)
-63.37
-76.91
11A
CF3
0 -20 -40 -60 -80 -100 -120 -140 -160 -180 -200 -220 -240 -260 -280 -300
f1 (ppm)

HSQC (**11A**)

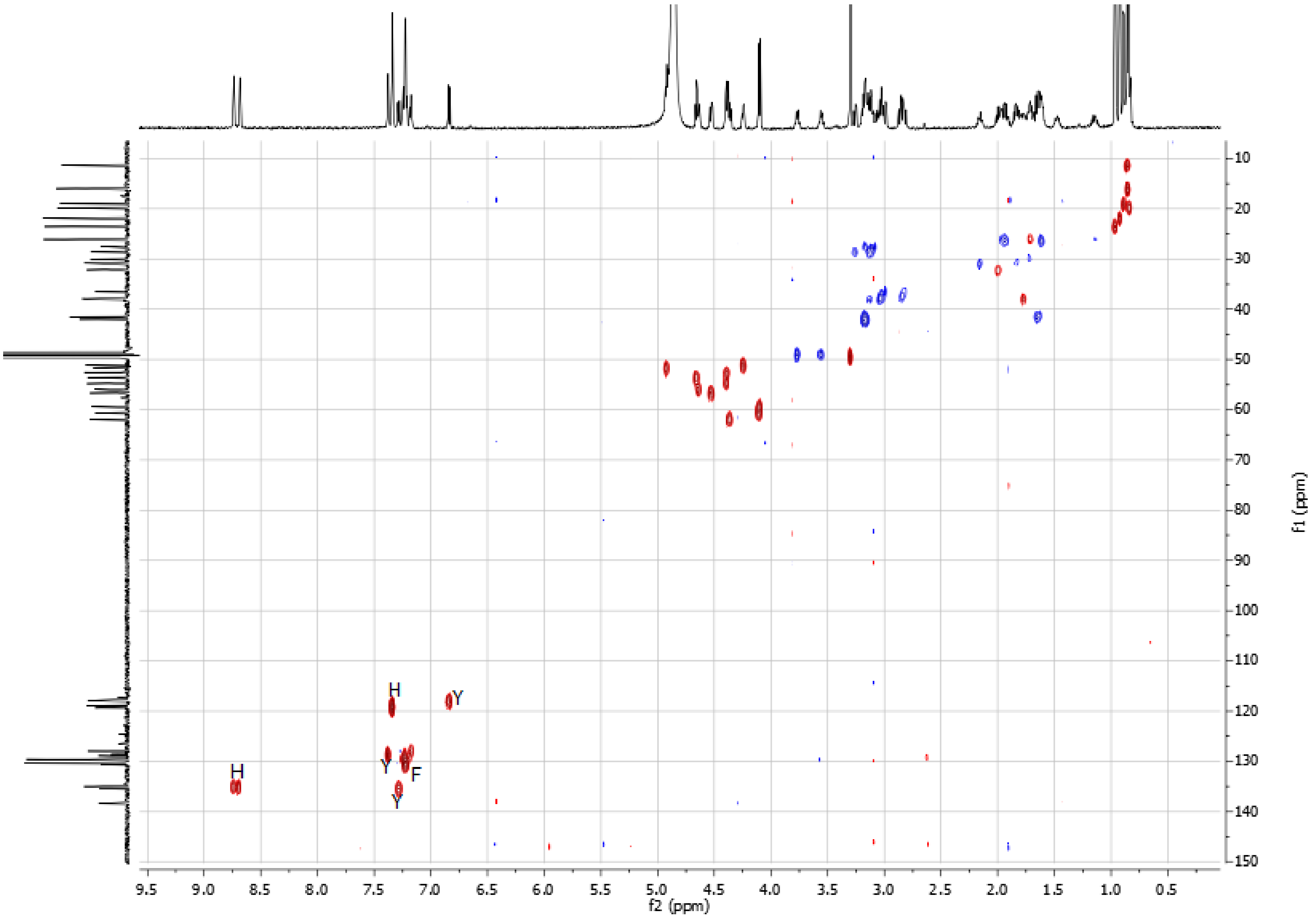

HSQC aromatic region (**11A**)

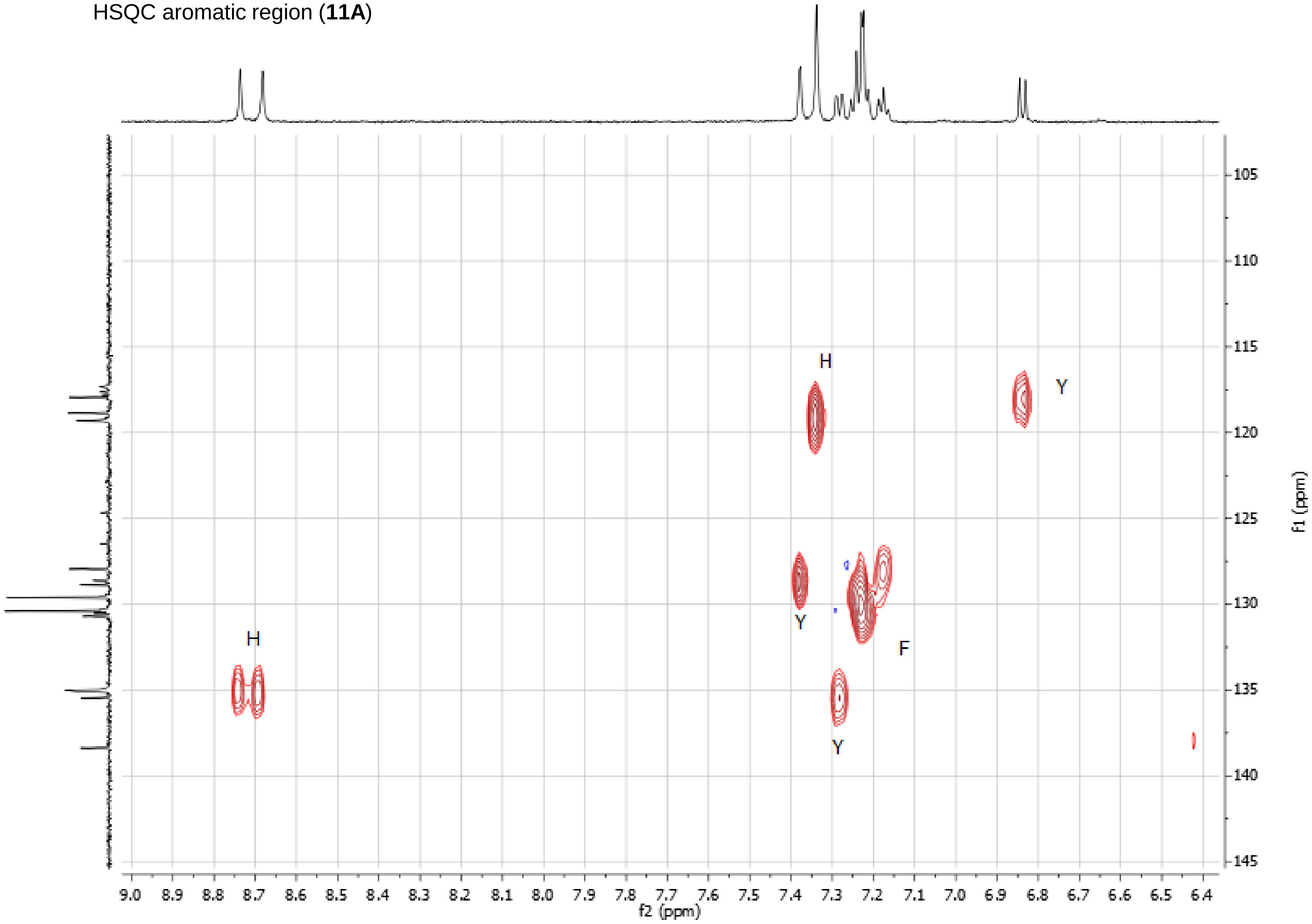

COSY (compound **11A**)

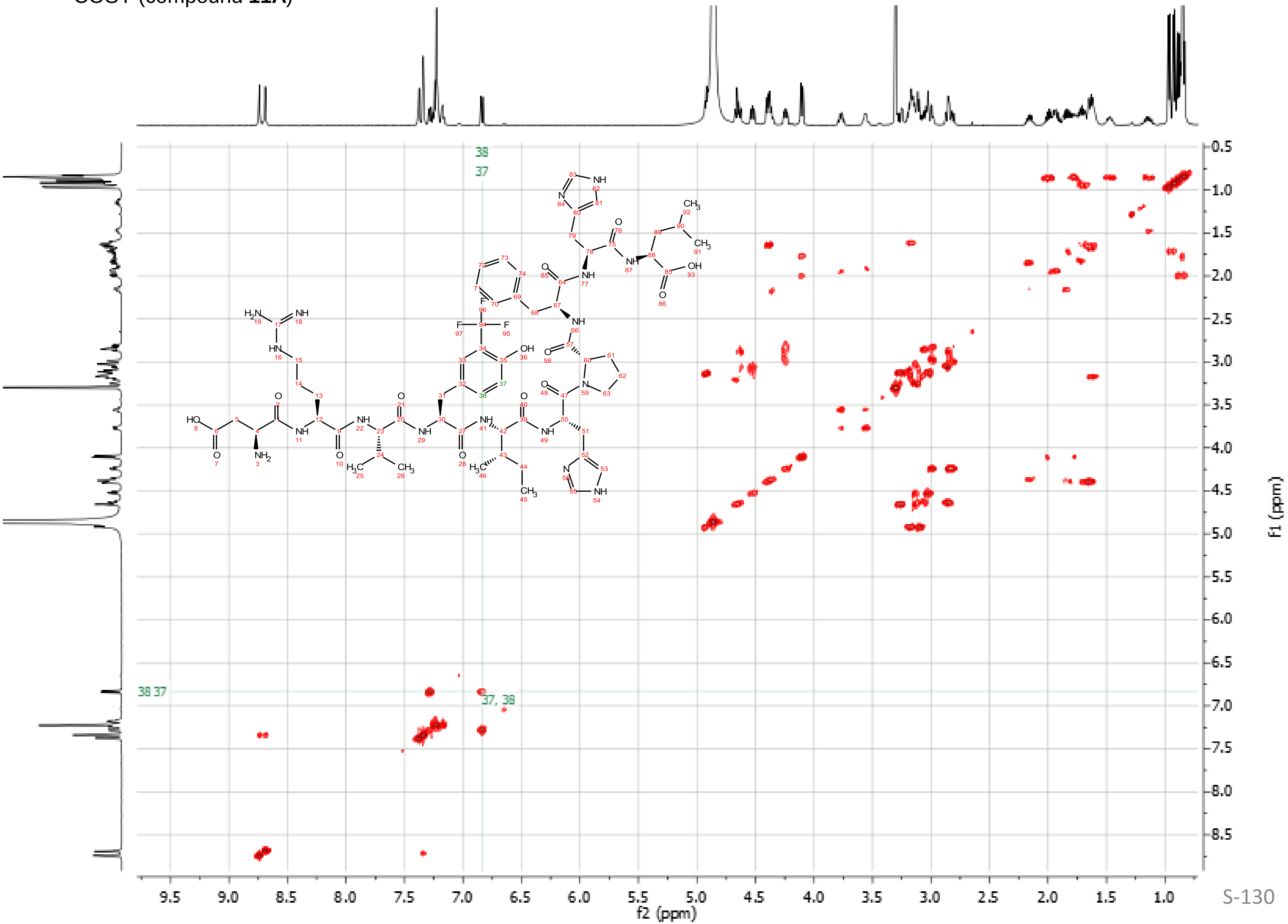

COSY, aromatic region (compound **11A**)

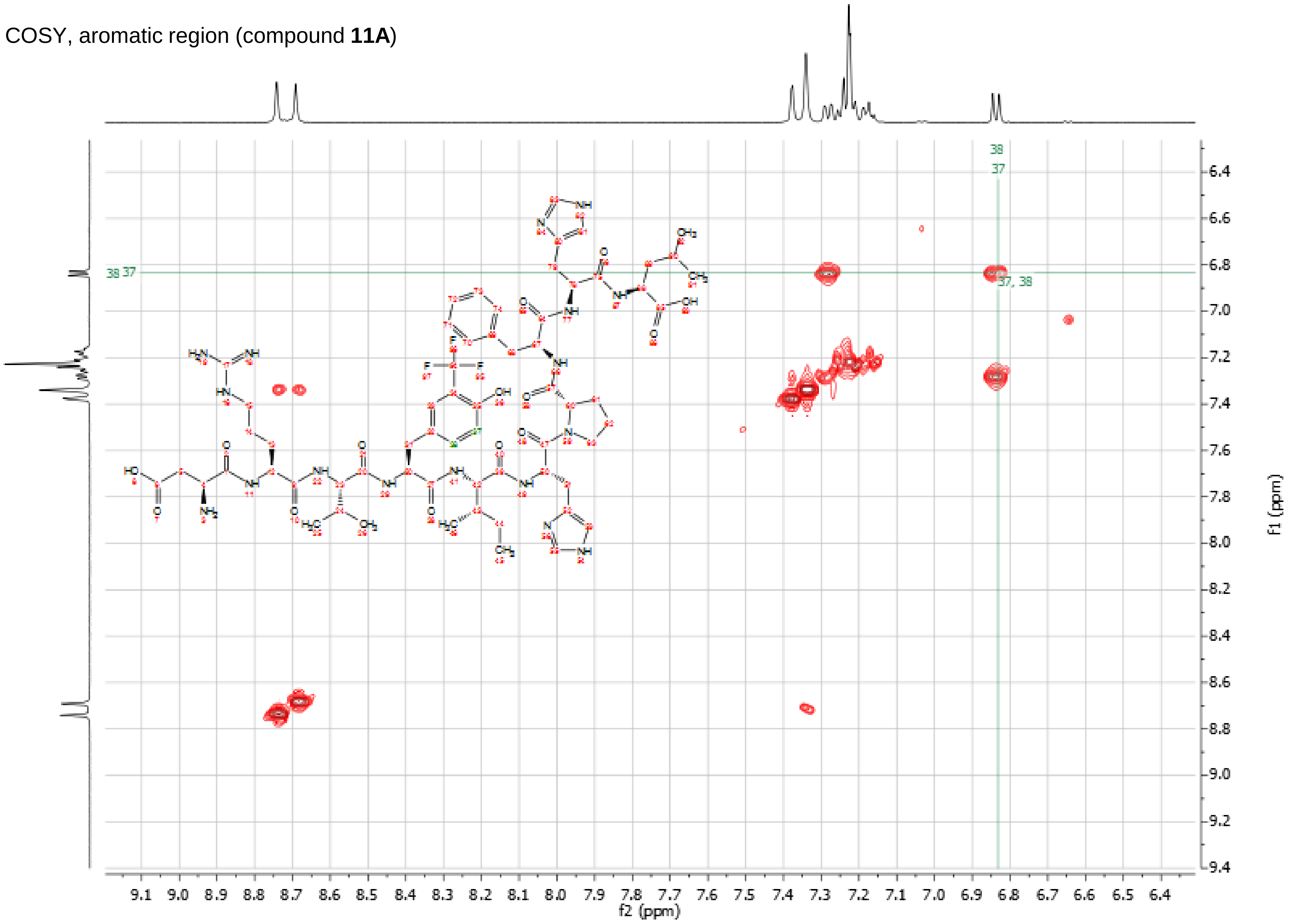

HMBC (**11A**)

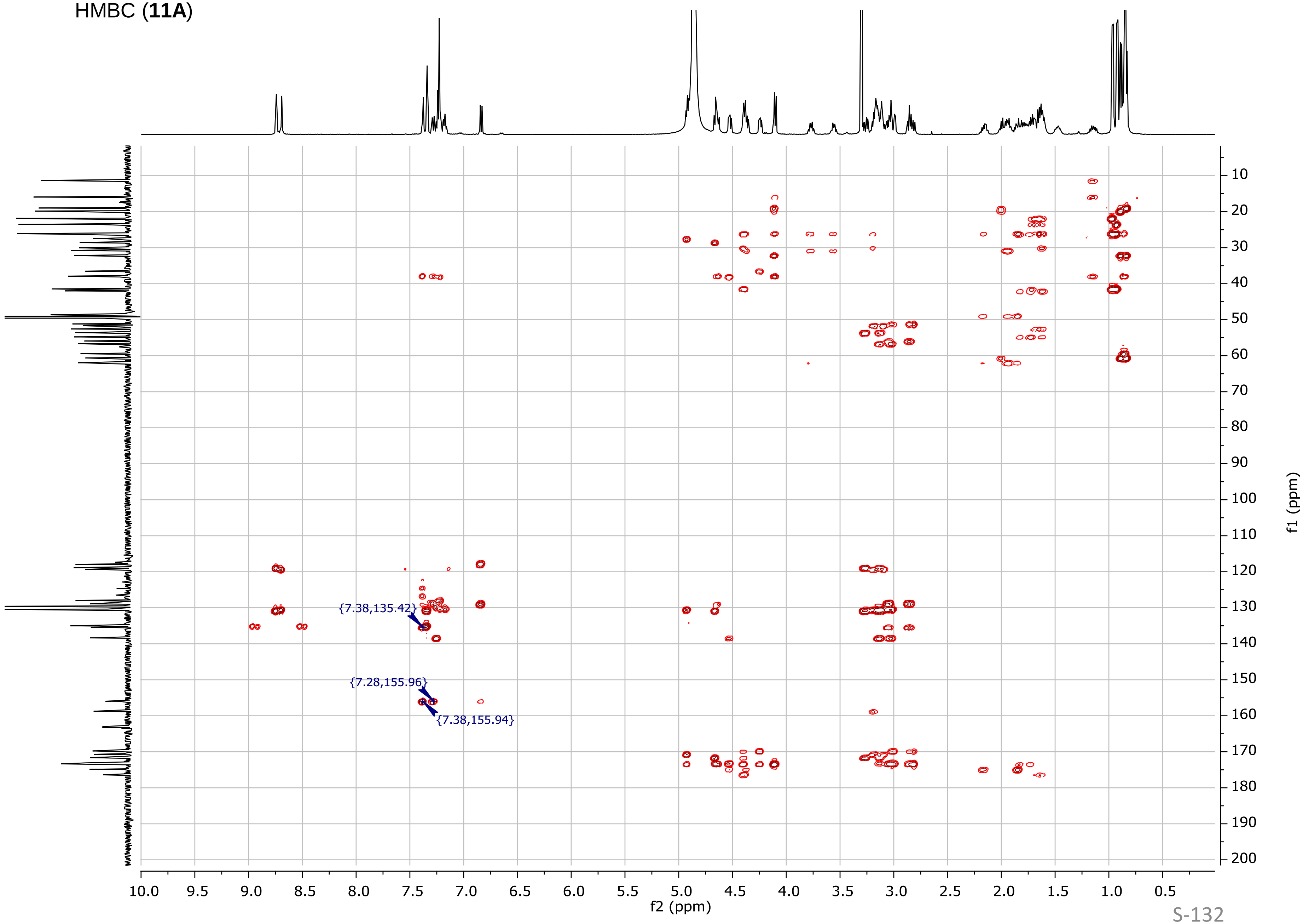

HMBC expansion (**11A**)

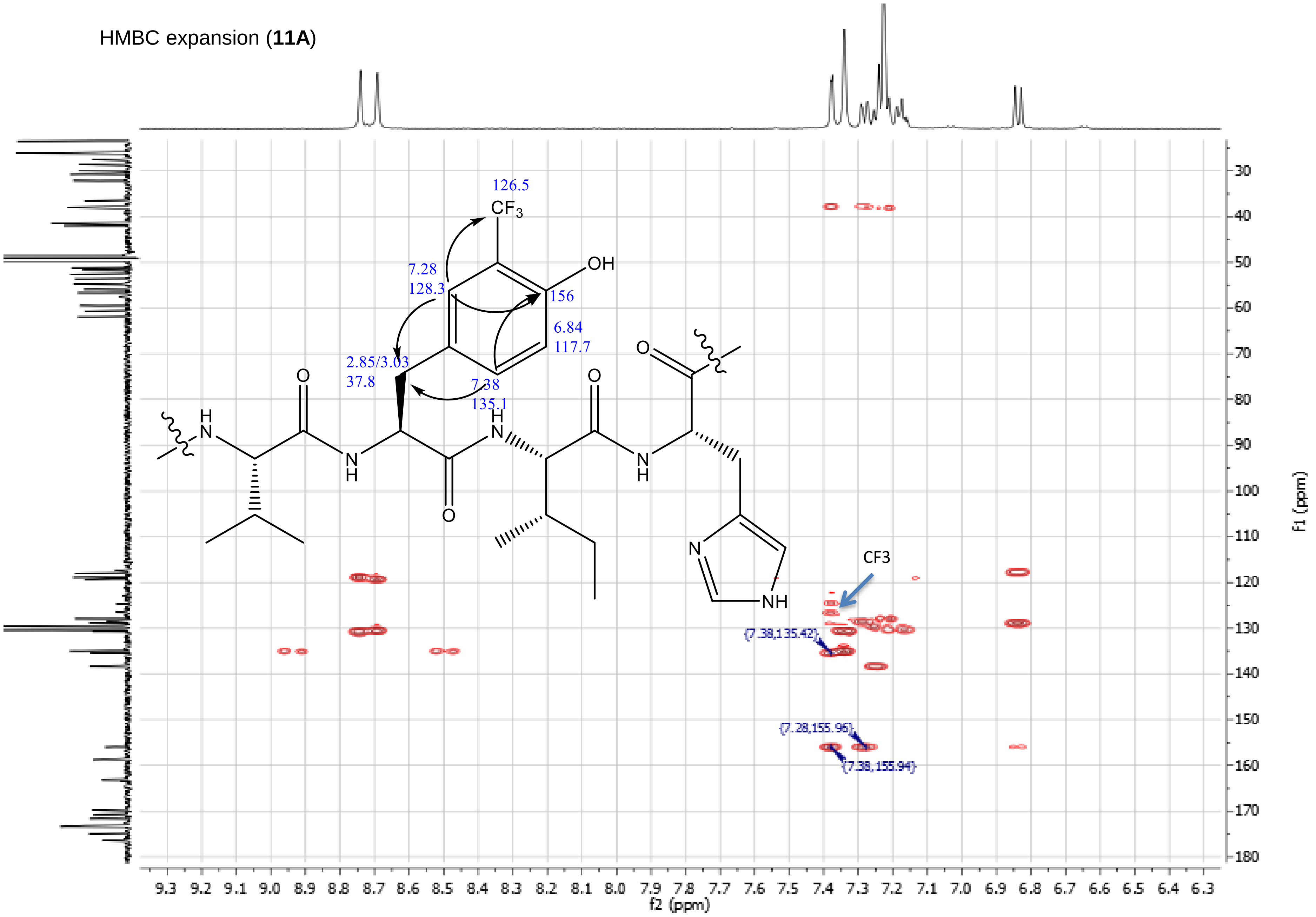

gHMBC aromatic (**11A**)

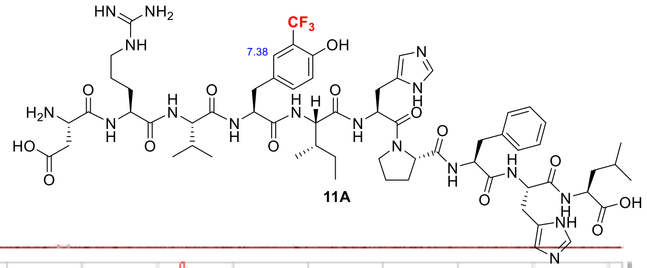

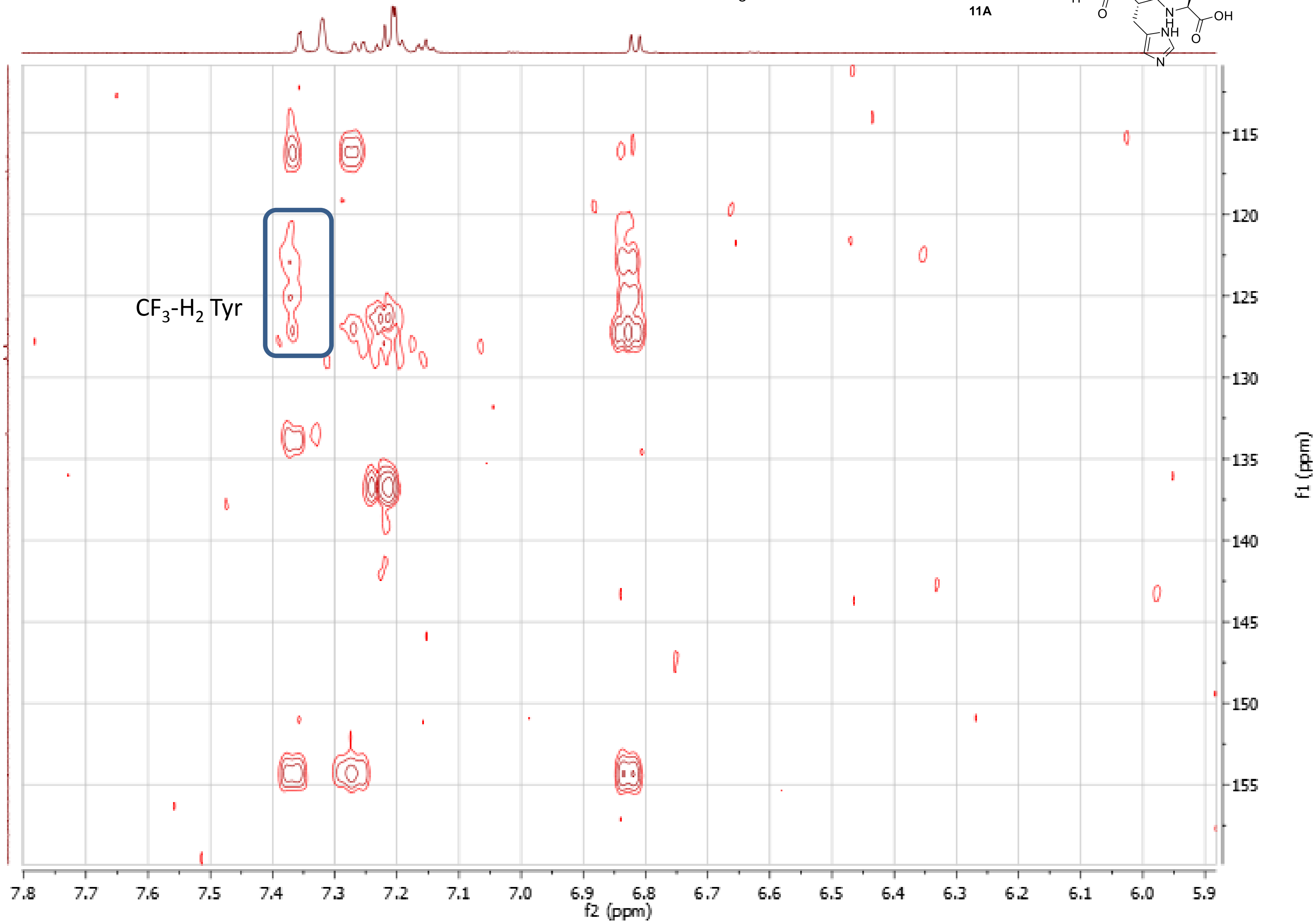

^1H NMR (600 MHz, Methanol-d_4)

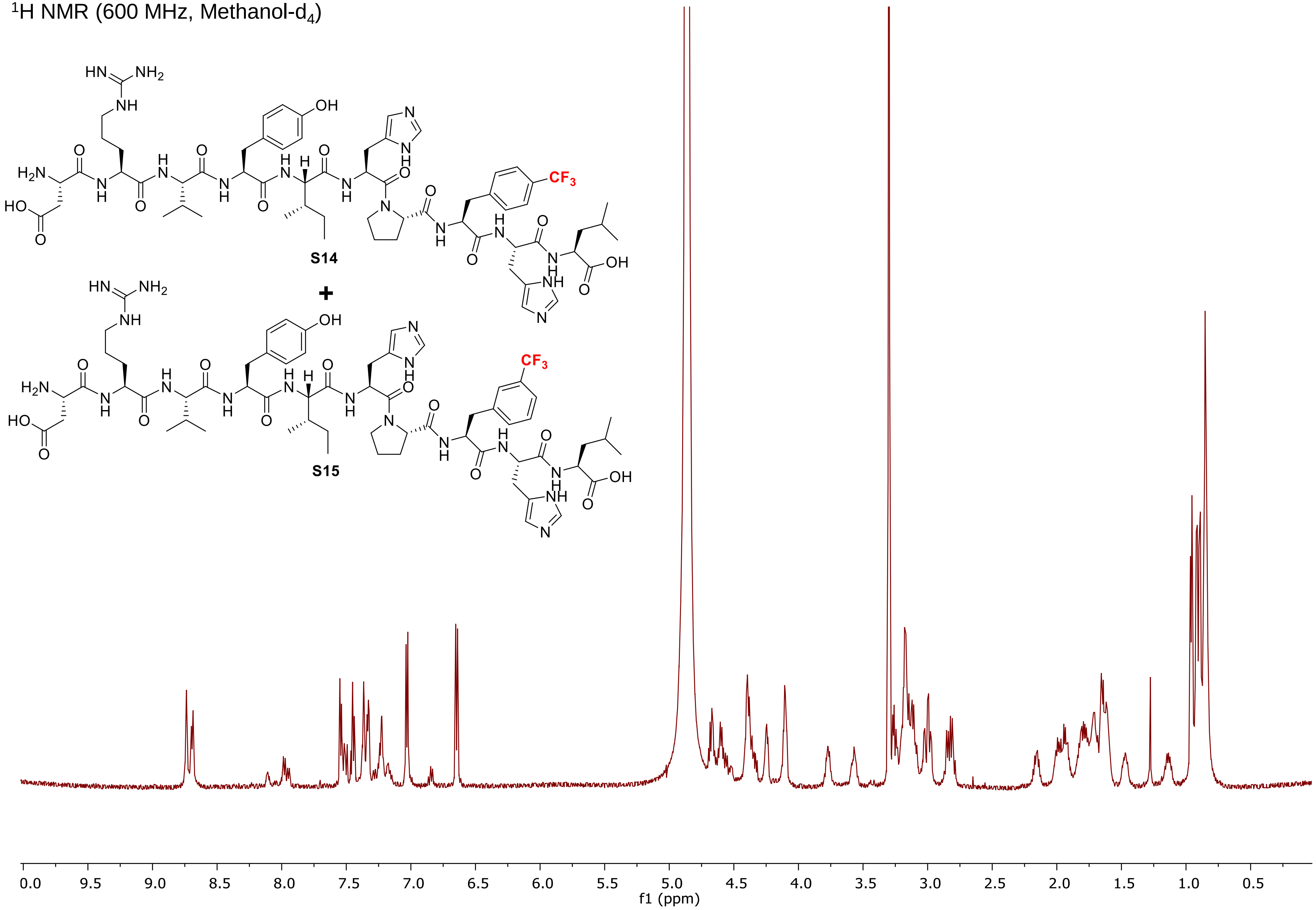

^{19}F NMR (471 MHz, Methanol-d_4)

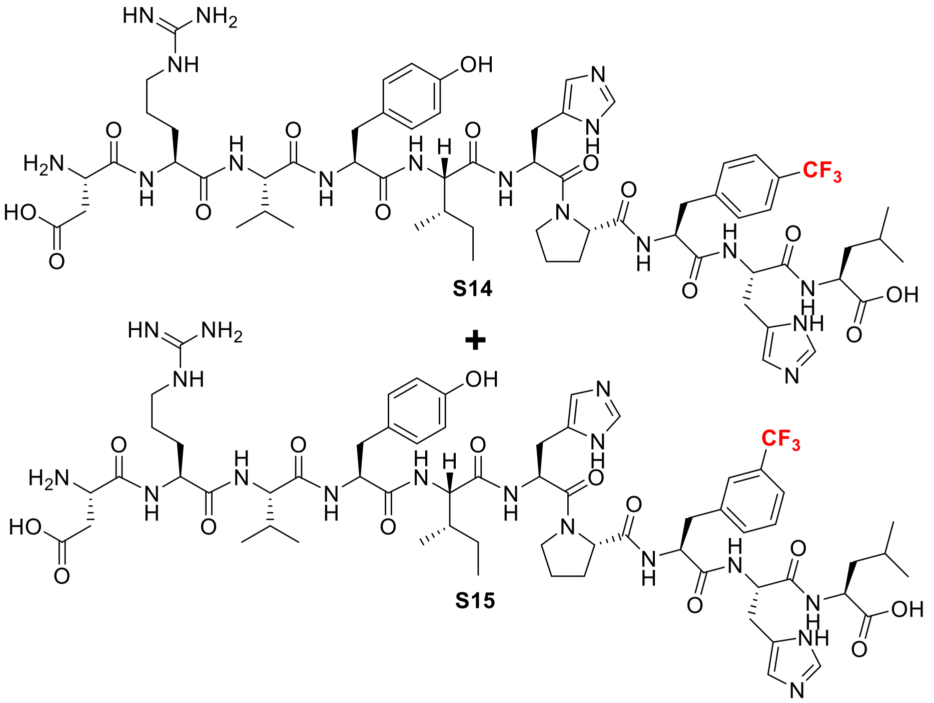

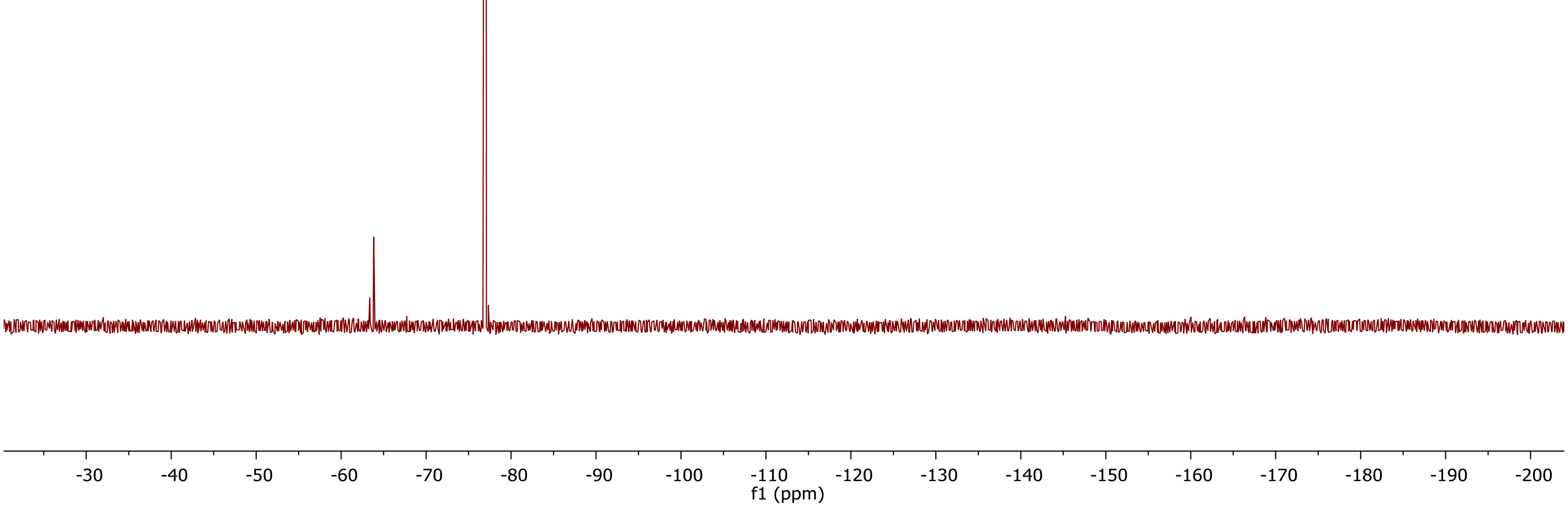

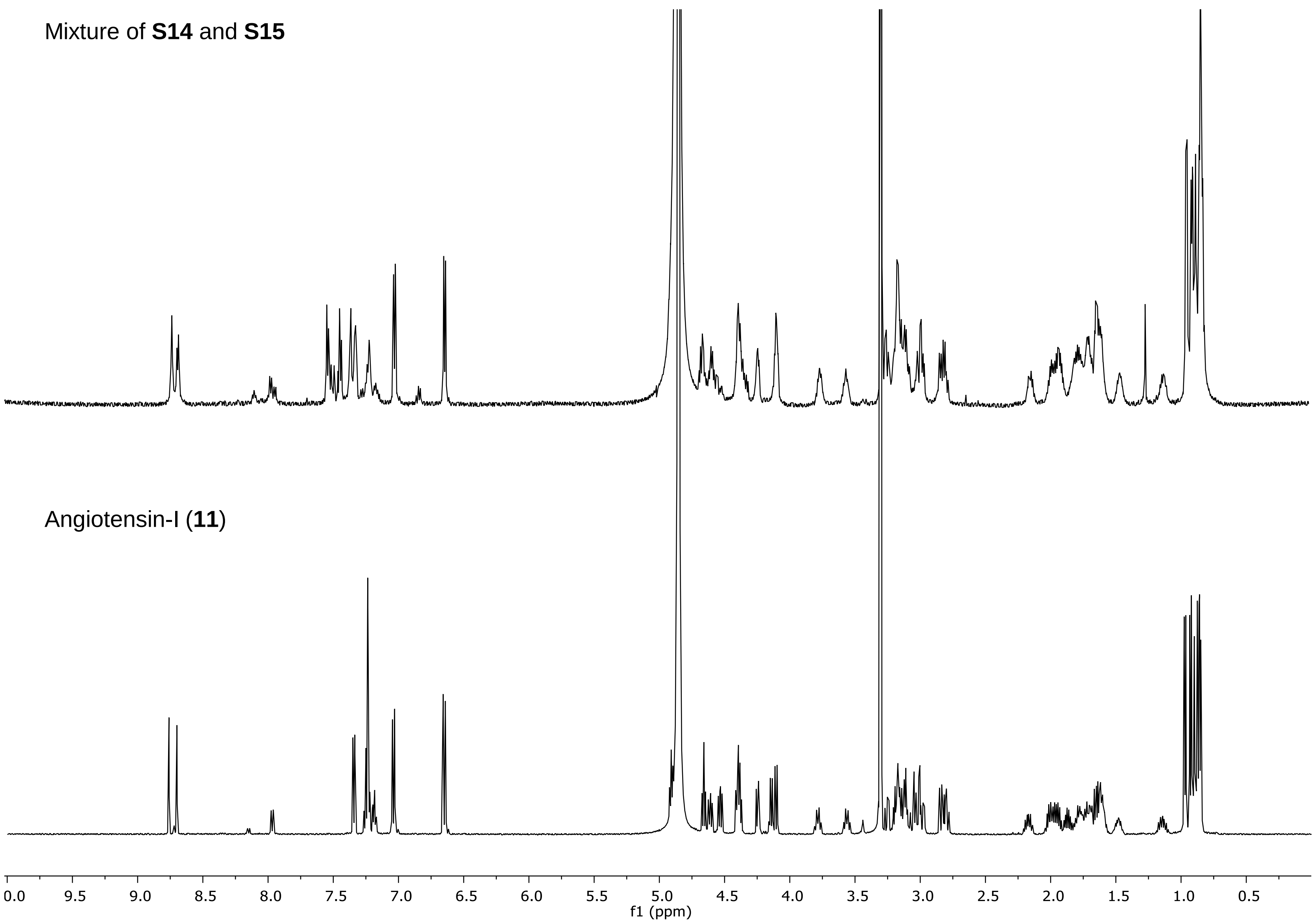

Mixture of **S14** and **S15**
Angiotensin-I (**11**)
0.0
9.5
9.0
8.5
8.0
7.5
7.0
6.5
6.0
5.5
5.0
4.5
4.0
3.5
3.0
2.5
2.0
1.5
1.0
0.5
f1 (ppm)

COSY (**S14** and **S15**)

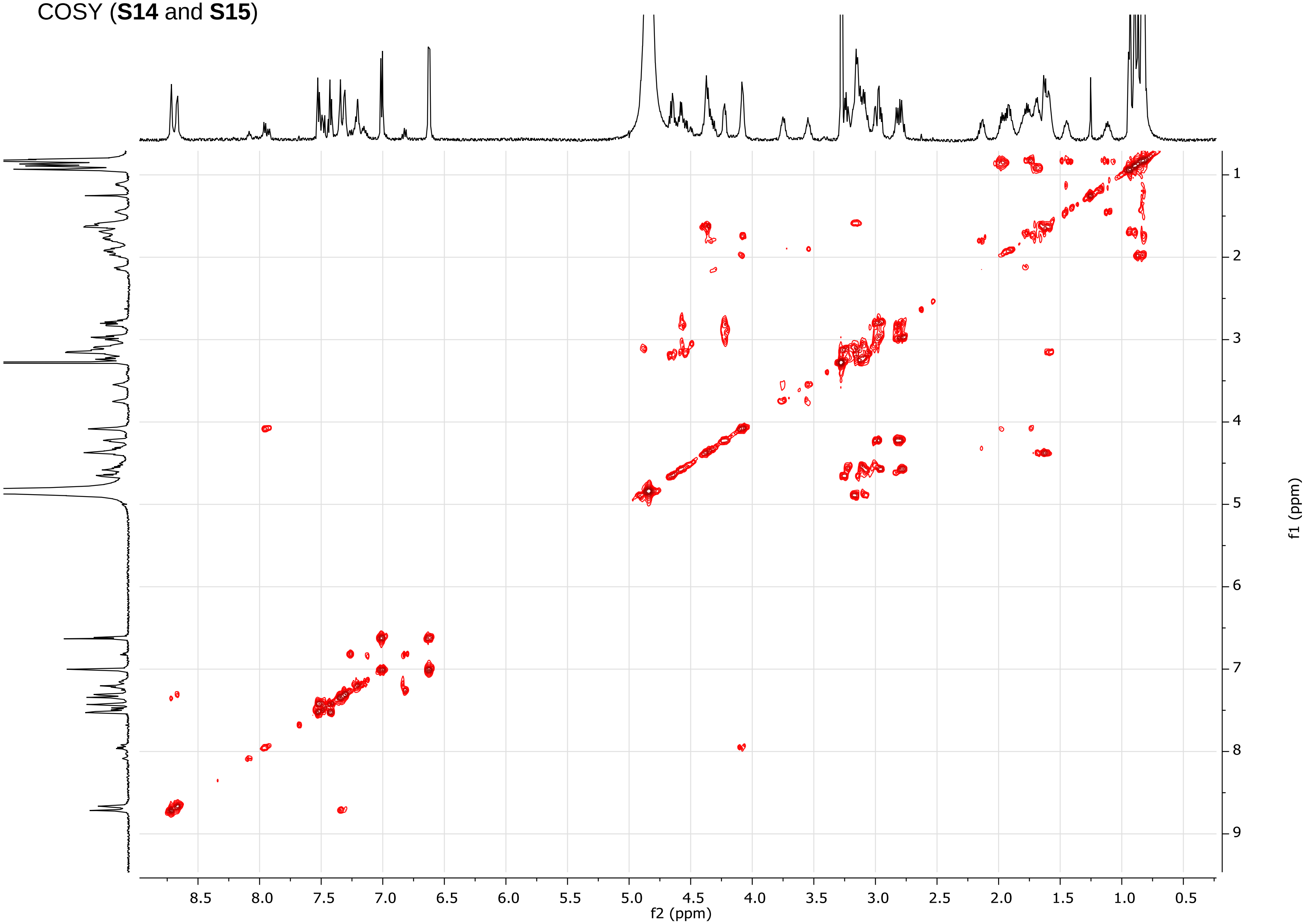

COSY aromatic region (**S14** and **S15**)

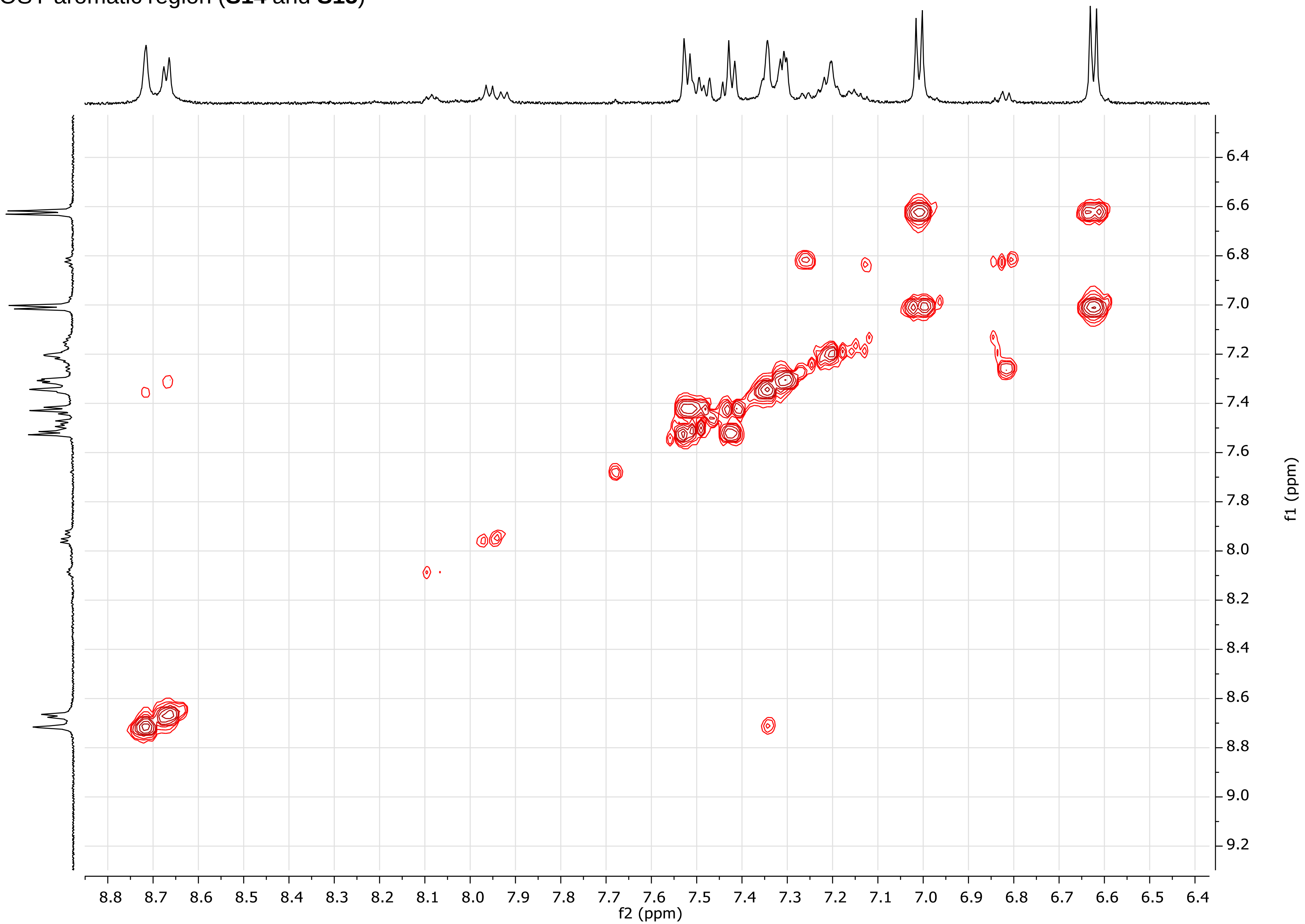

HSQC aromatic region (**S14** and **S15**)

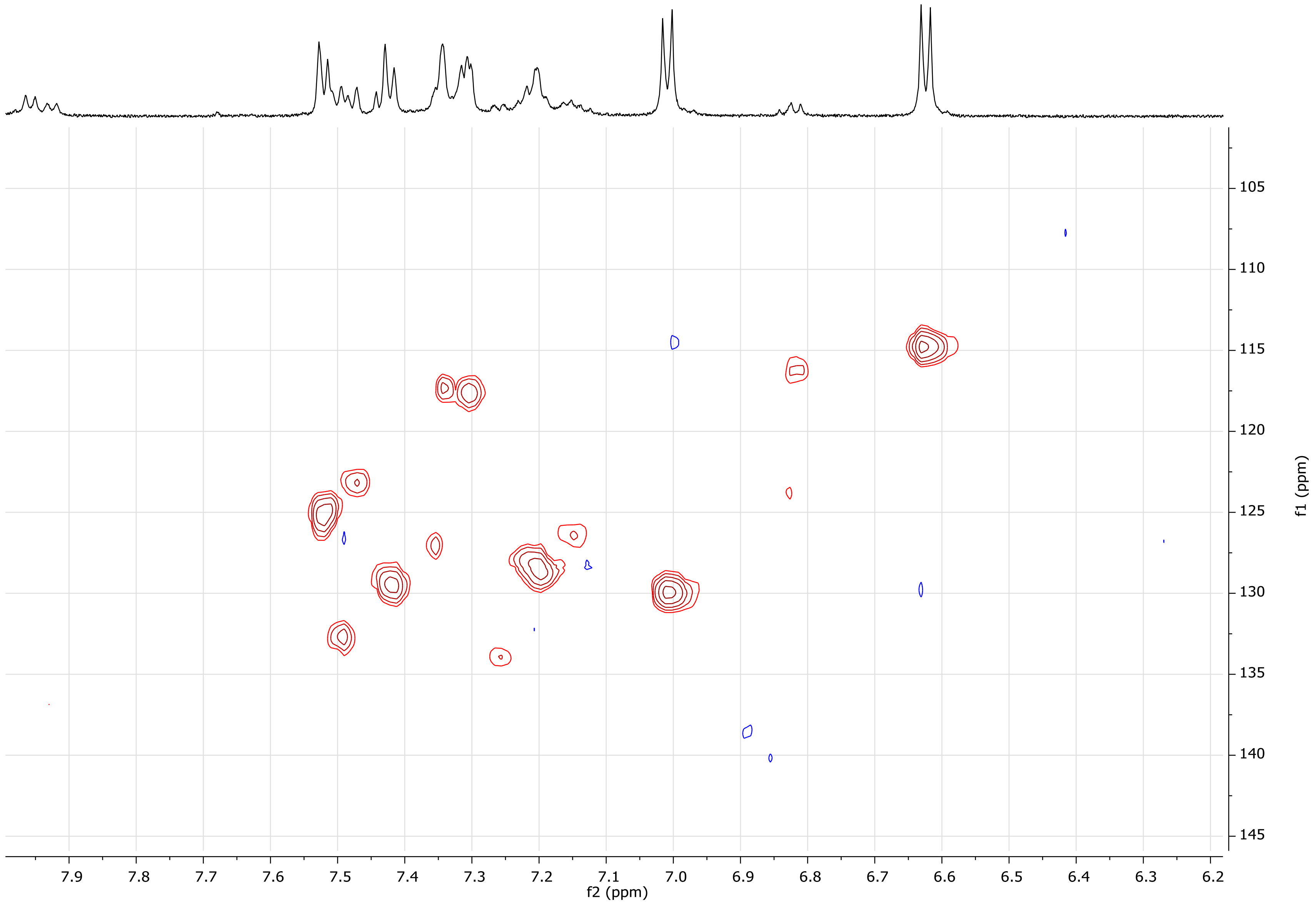

^{1}H NMR (600 MHz, Methanol-d_4)

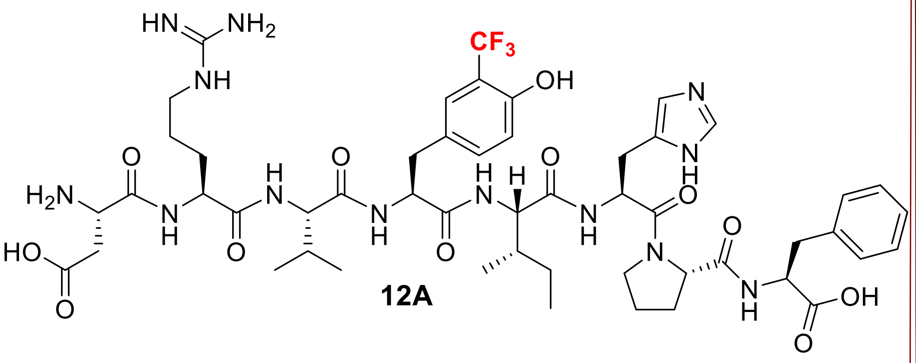

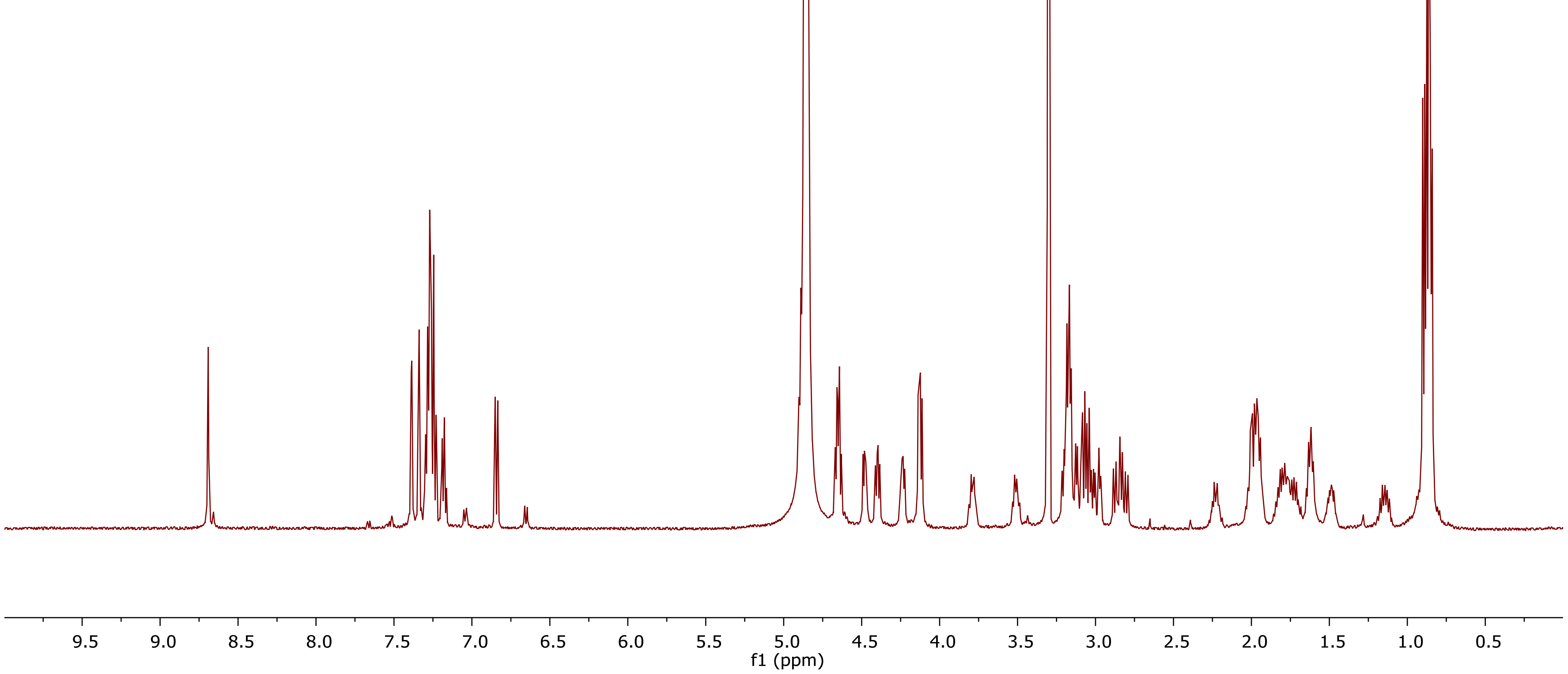

^{13}C NMR (150 MHz, Methanol-d_4)

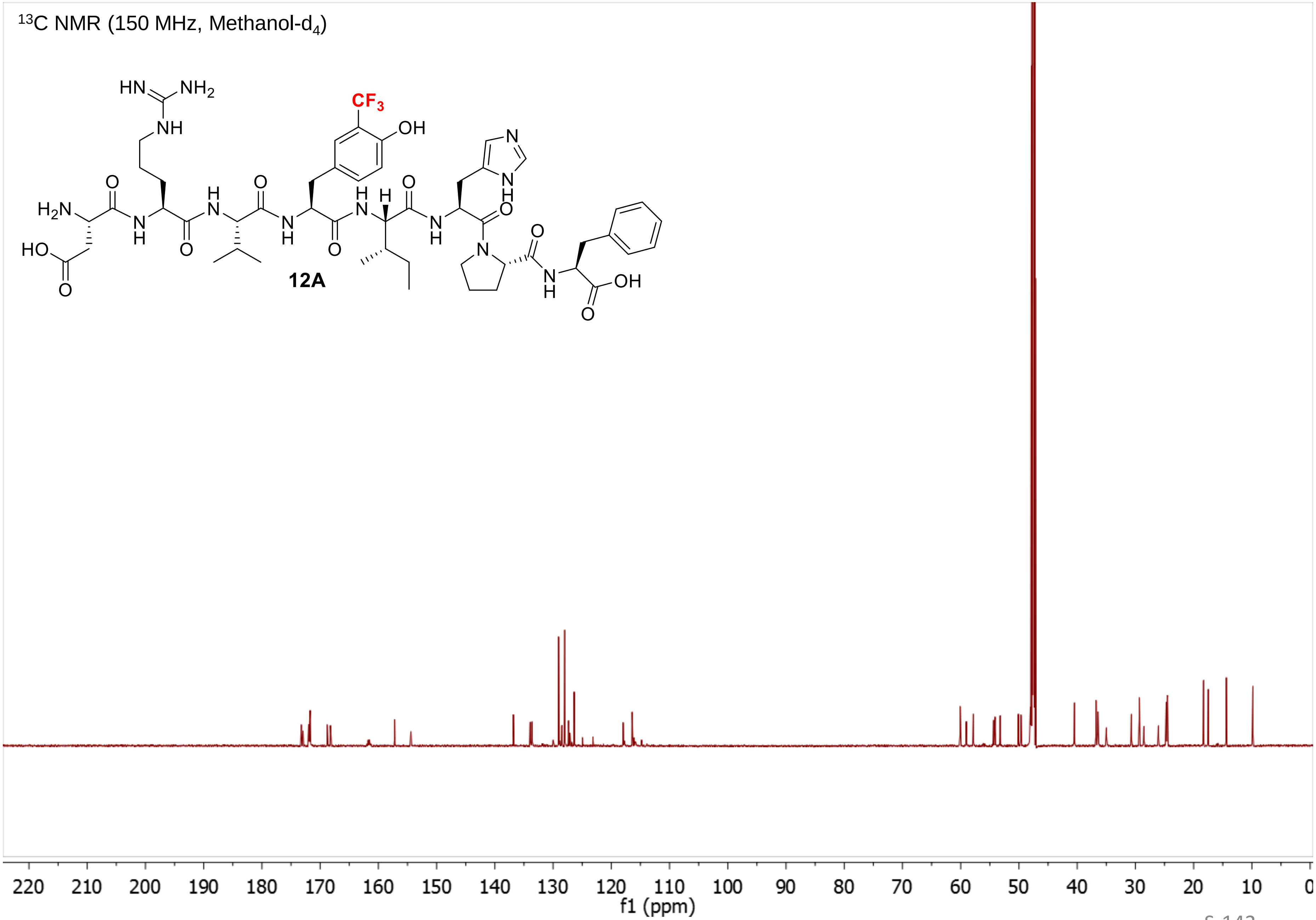

^{19}F NMR (471 MHz, Methanol-d_4)

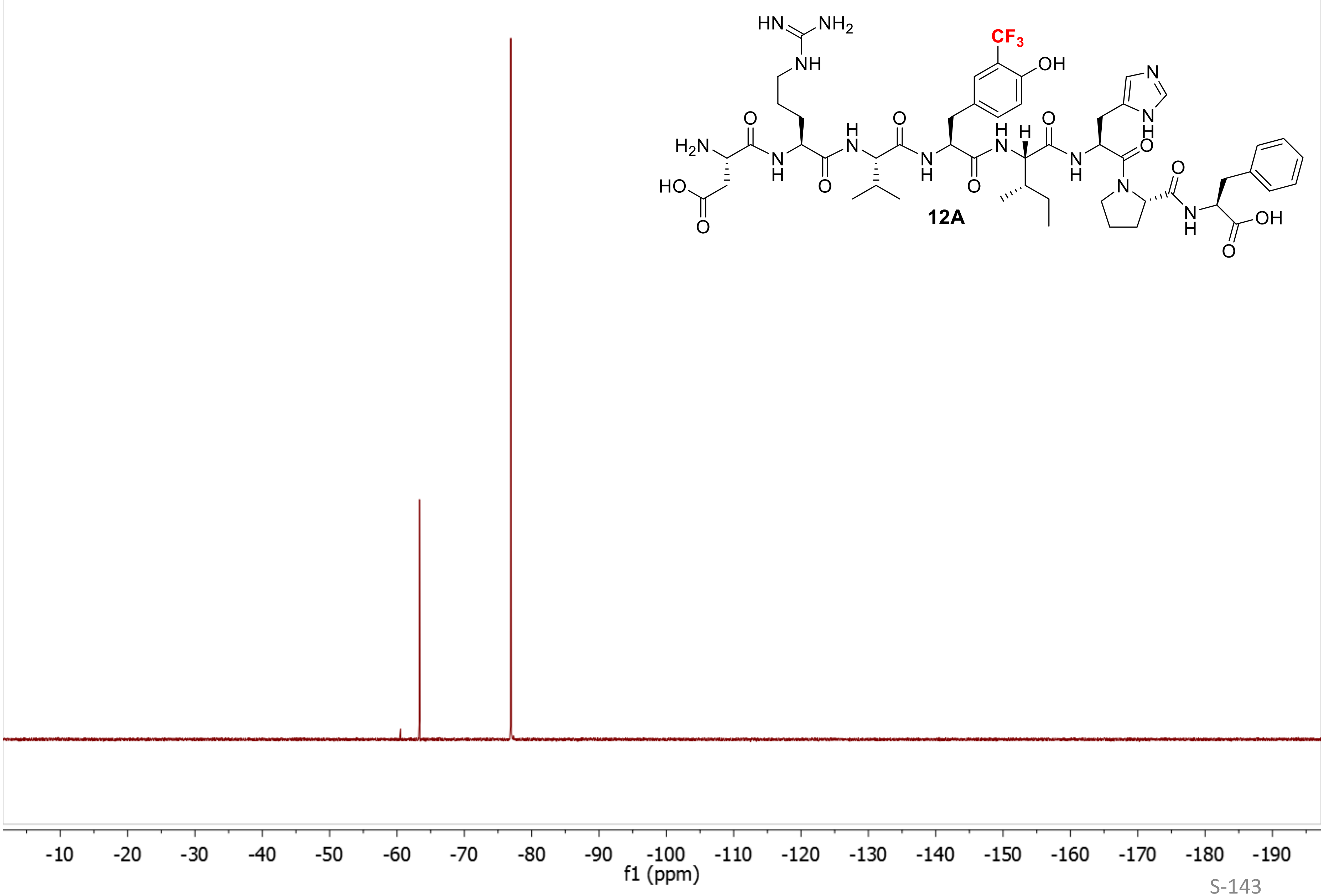

COSY aromatic region (**12A**)

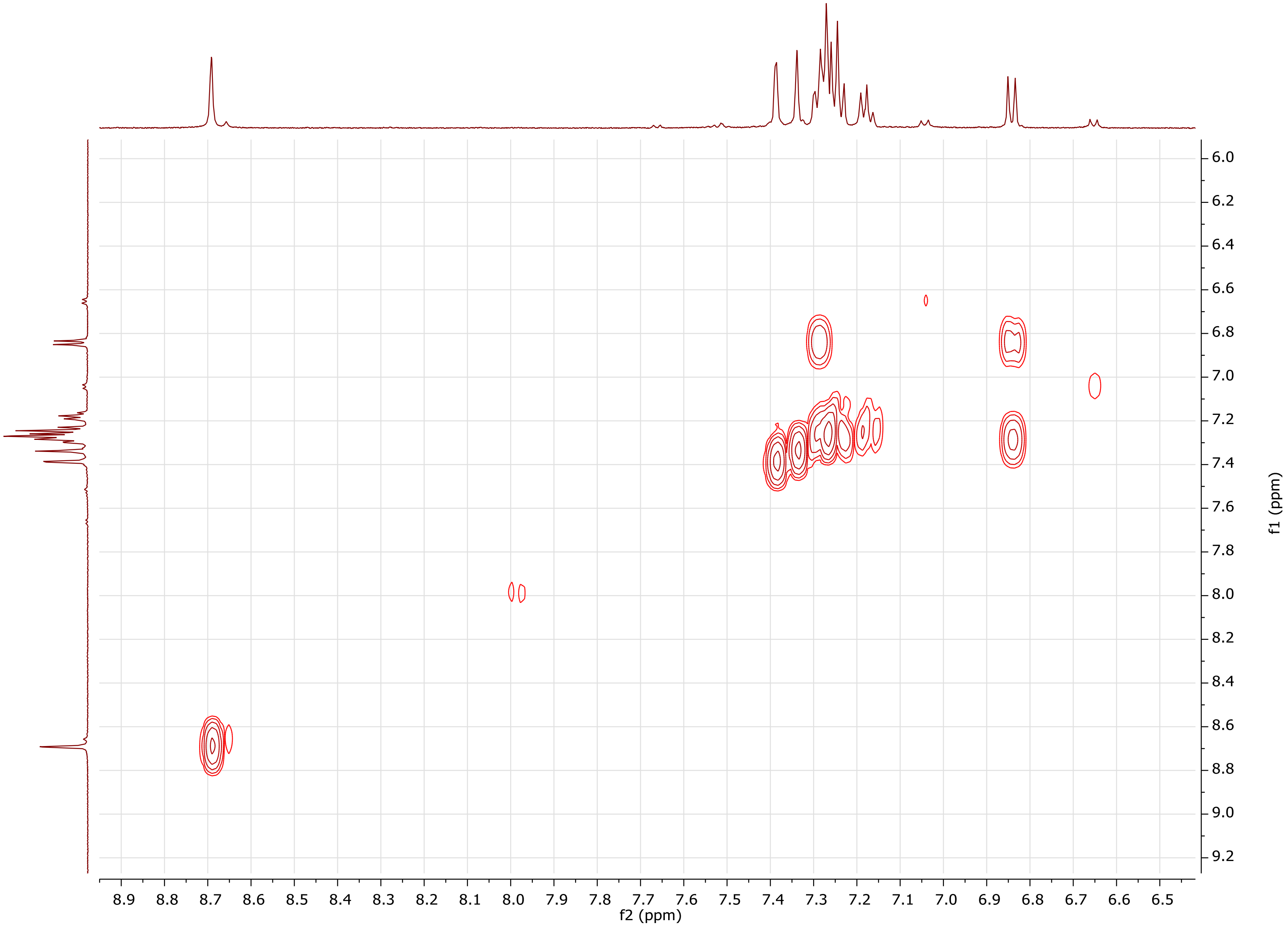

HSQC (**12A**)

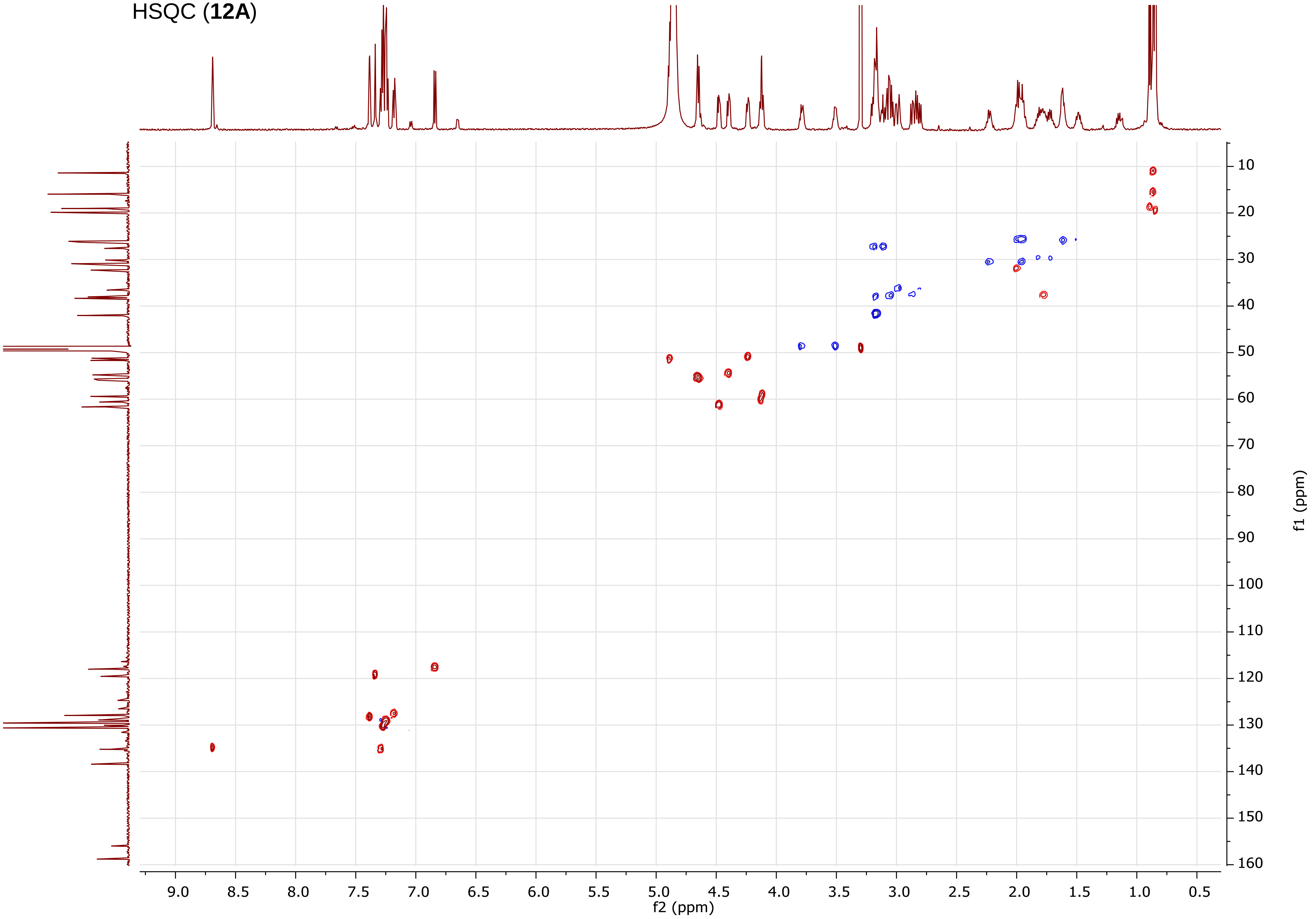

f2 (ppm)
f1 (ppm)
9.0
8.5
8.0
7.5
7.0
6.5
6.0
5.5
5.0
4.5
4.0
3.5
3.0
2.5
2.0
1.5
1.0
0.5
10
20
30
40
50
60
70
80
90
100
110
120
130
140
150
160

HSQC aromatic region (**12A**)

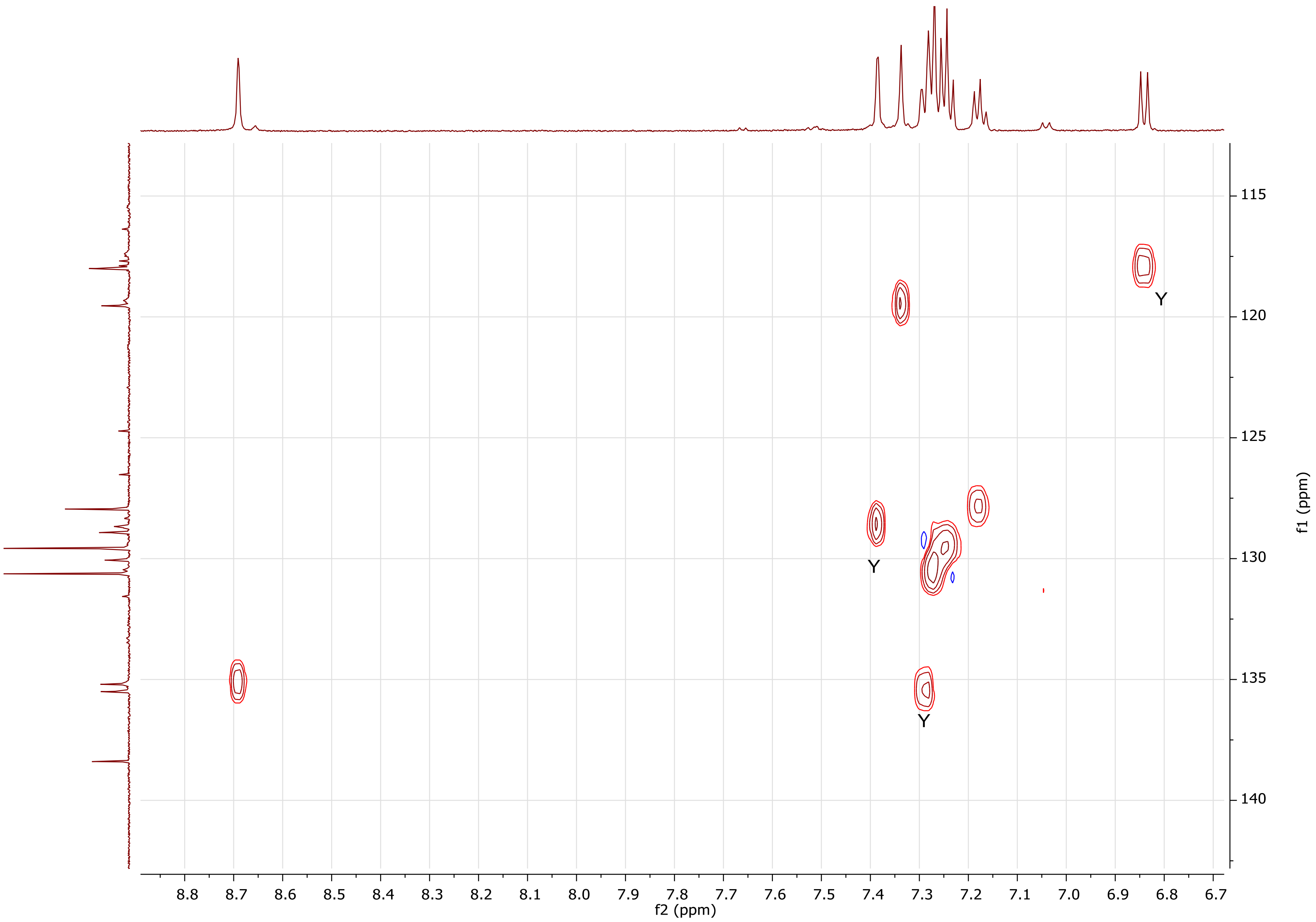

HMBC (**12A**)

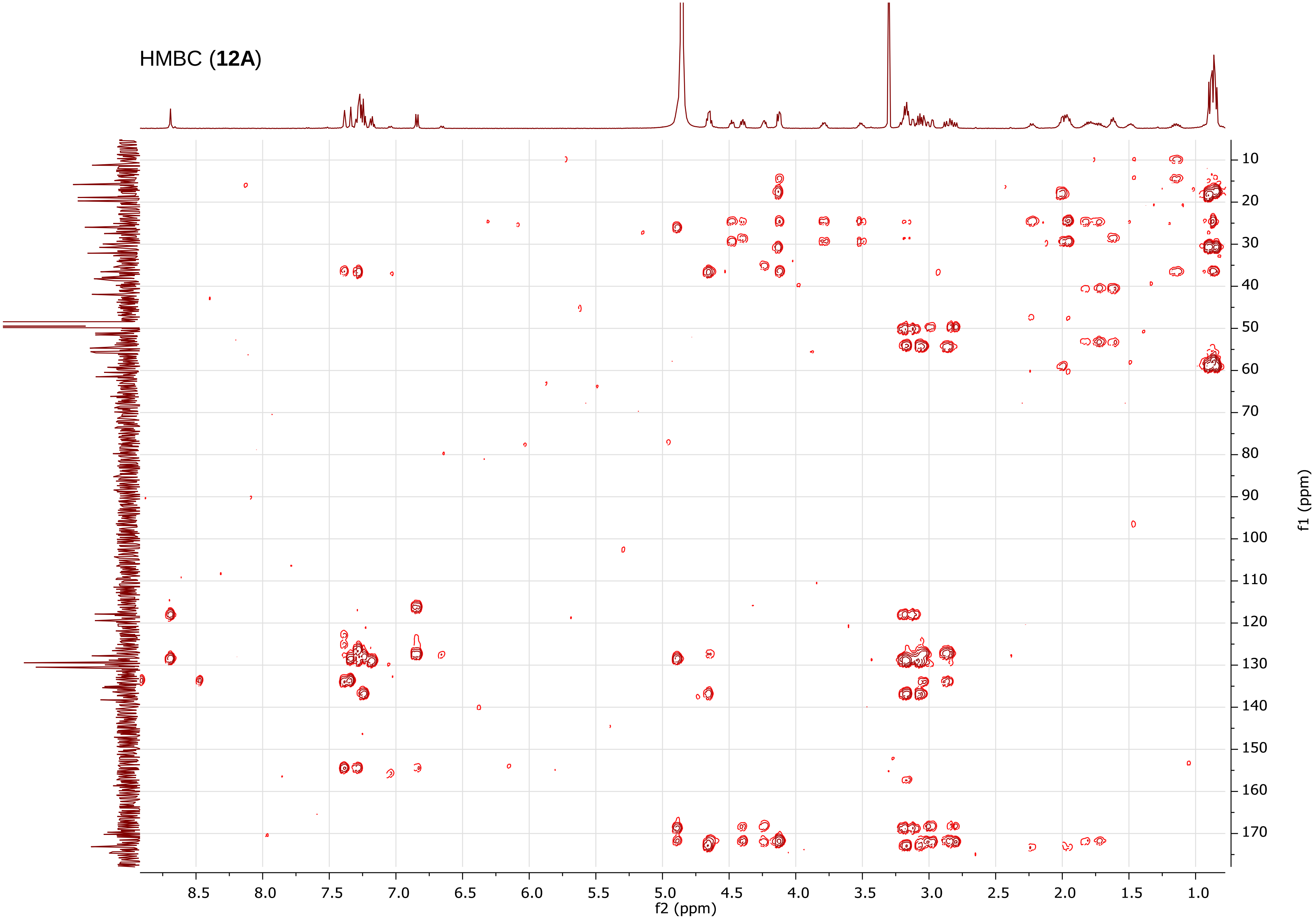

HMBC, aromatic region (**12A**)

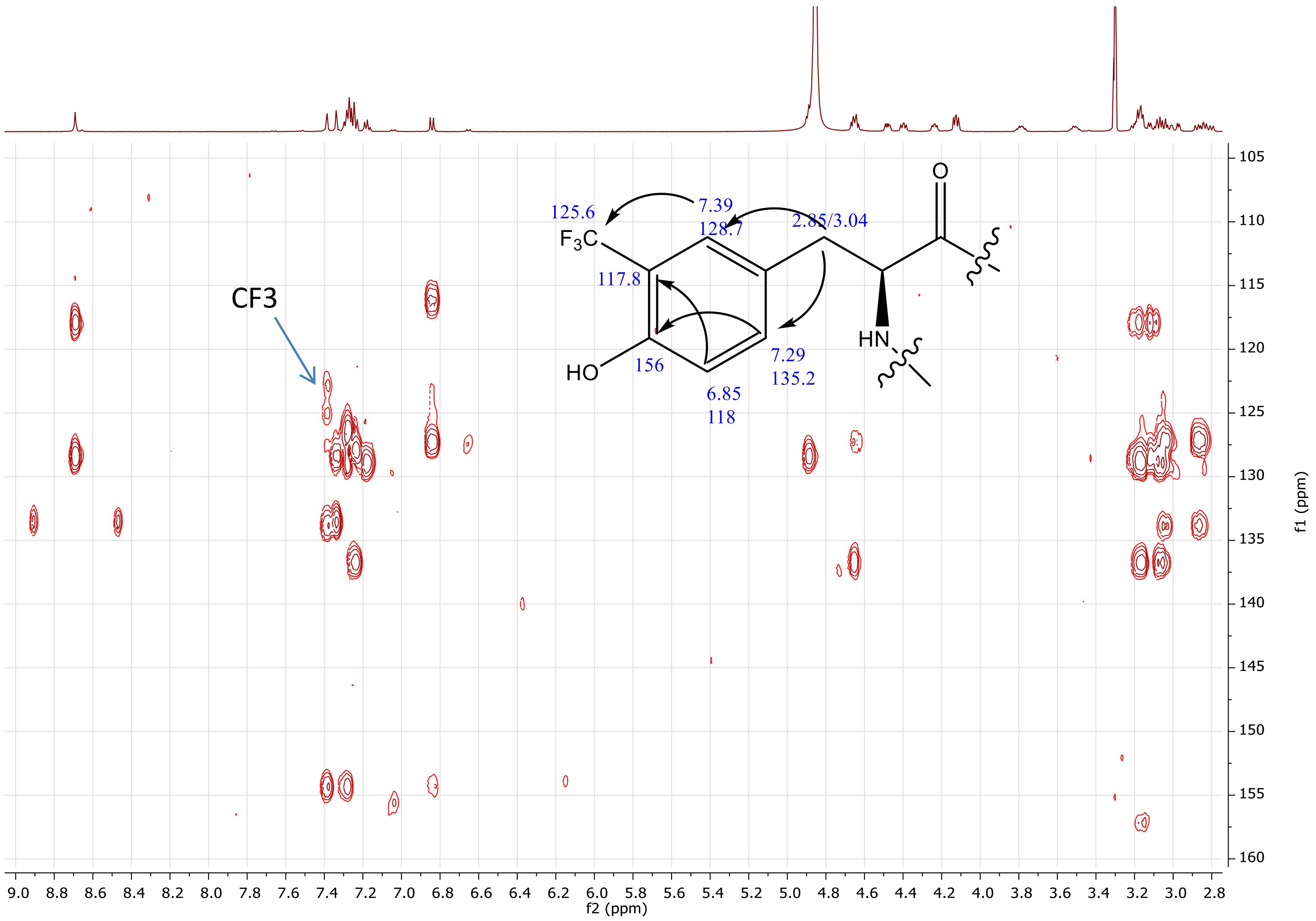

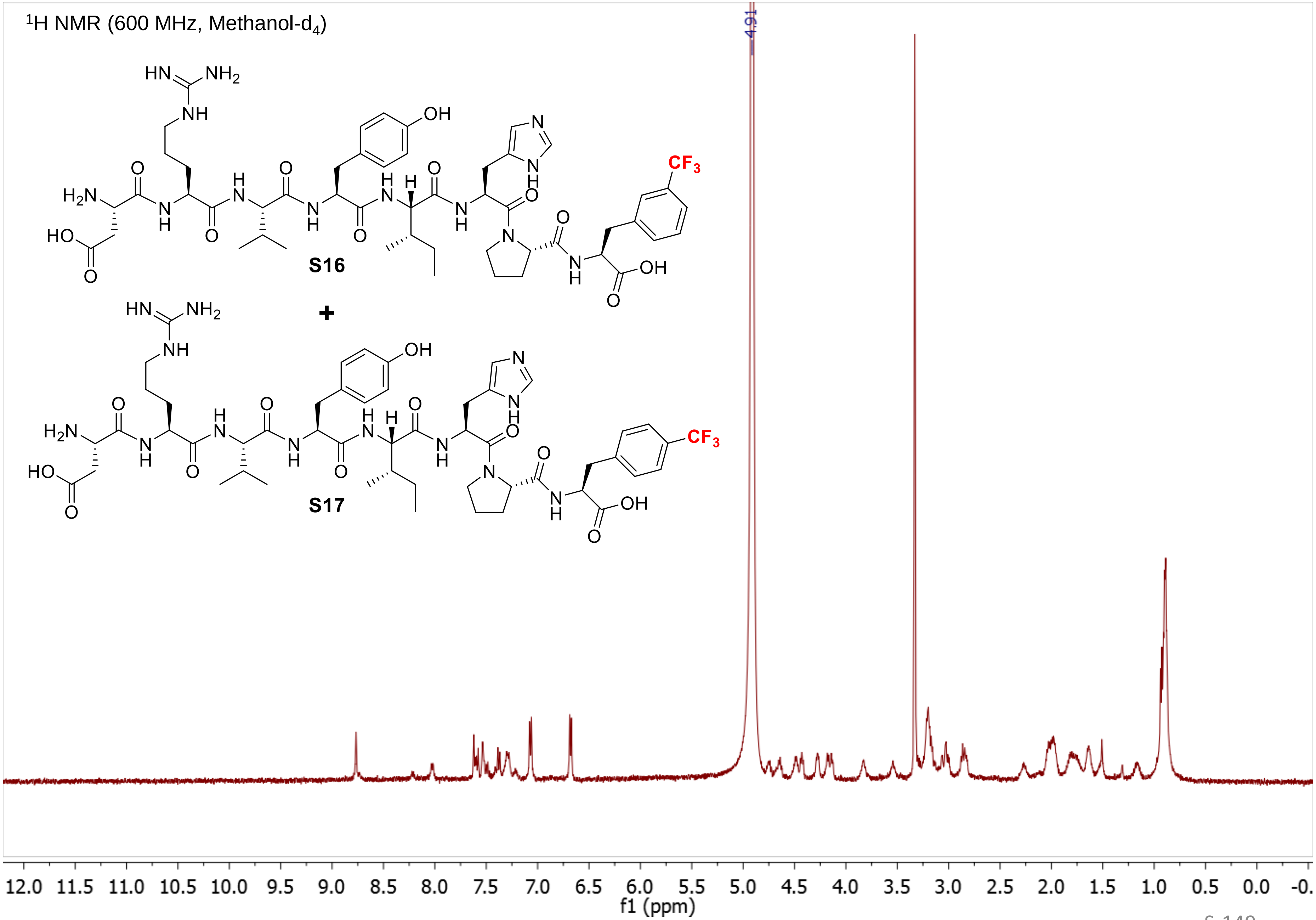

^{1}H NMR (600 MHz, Methanol-d$_4$)
S16
+
S17
CF$_3$
4.91
12.0 11.5 11.0 10.5 10.0 9.5 9.0 8.5 8.0 7.5 7.0 6.5 6.0 5.5 5.0 4.5 4.0 3.5 3.0 2.5 2.0 1.5 1.0 0.5 0.0
f1 (ppm)

^{19}F NMR (471 MHz, Methanol-d_4)

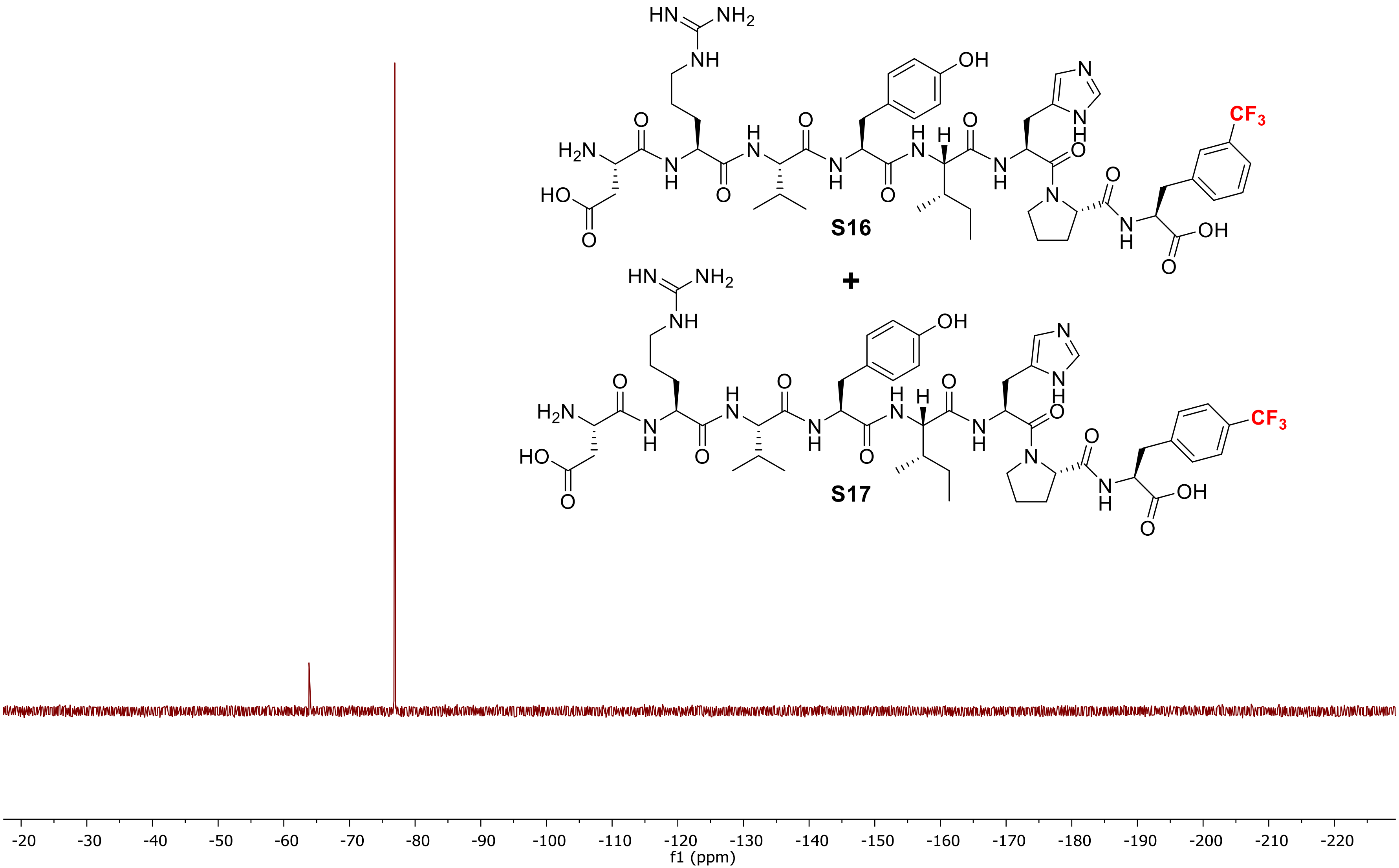

S16 + S17

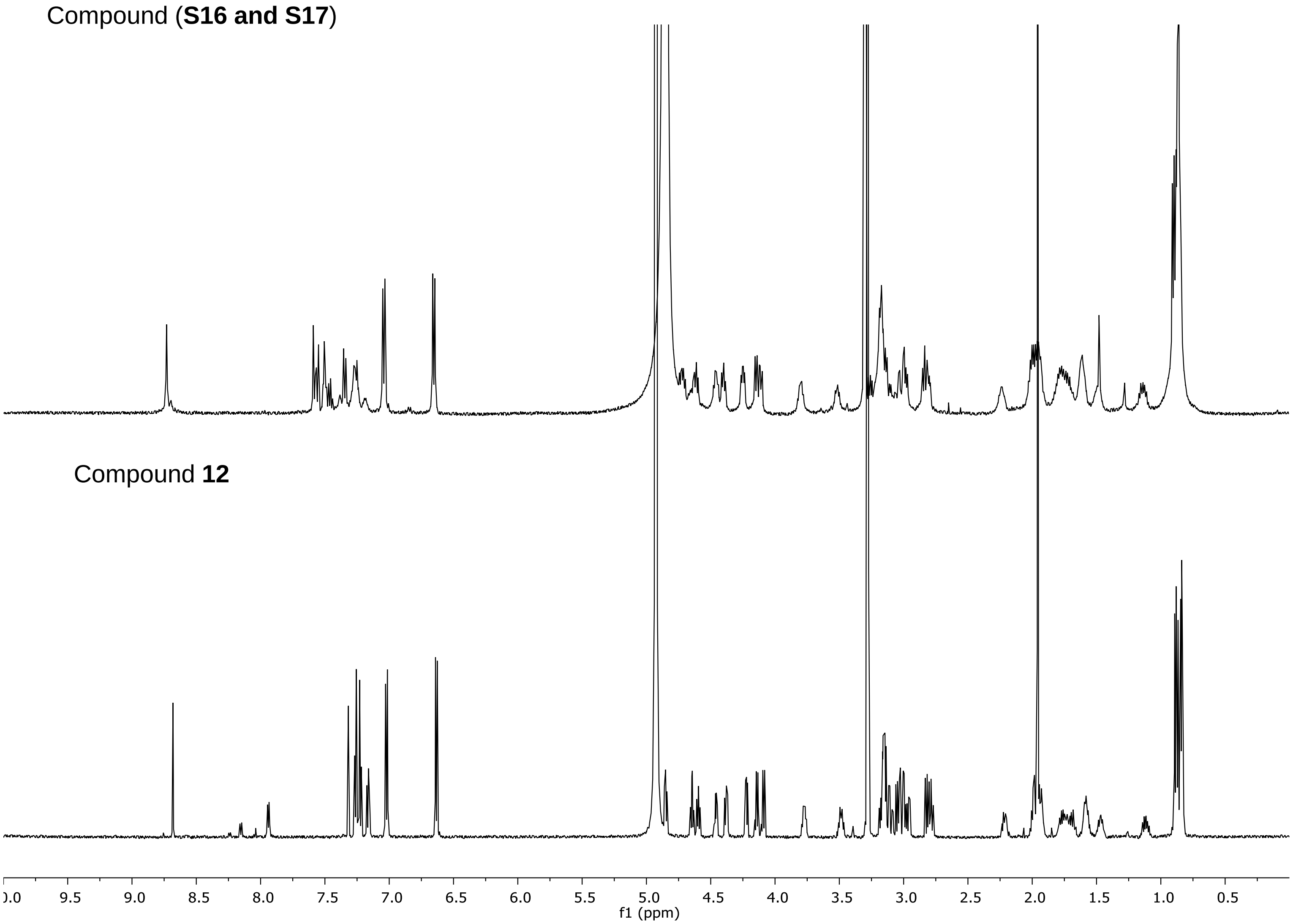
Compound (**S16 and S17**)
Compound **12**
f1 (ppm)

COSY (**S16 and S17**)

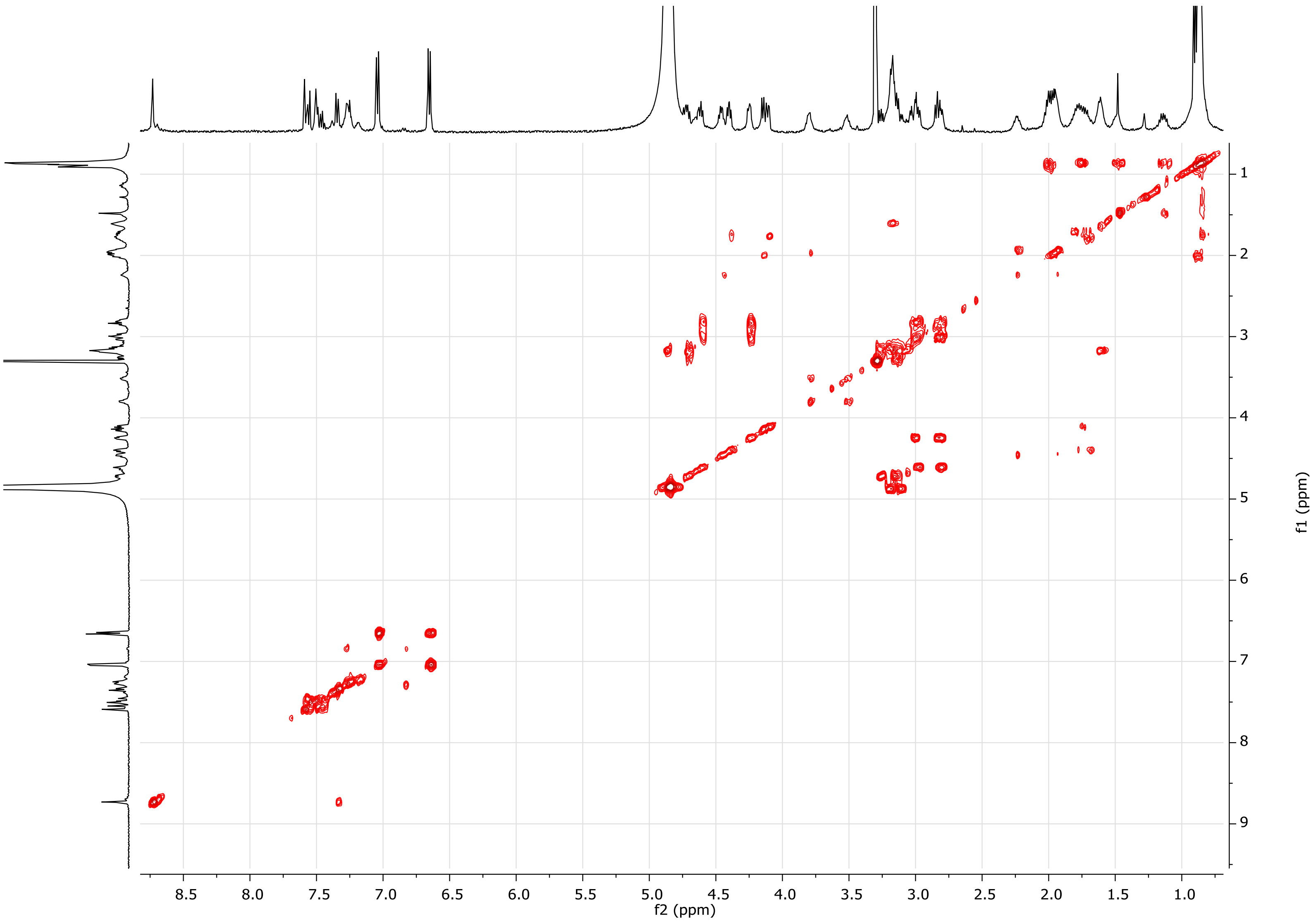

COSY aromatic region (**S16** and **S17**)

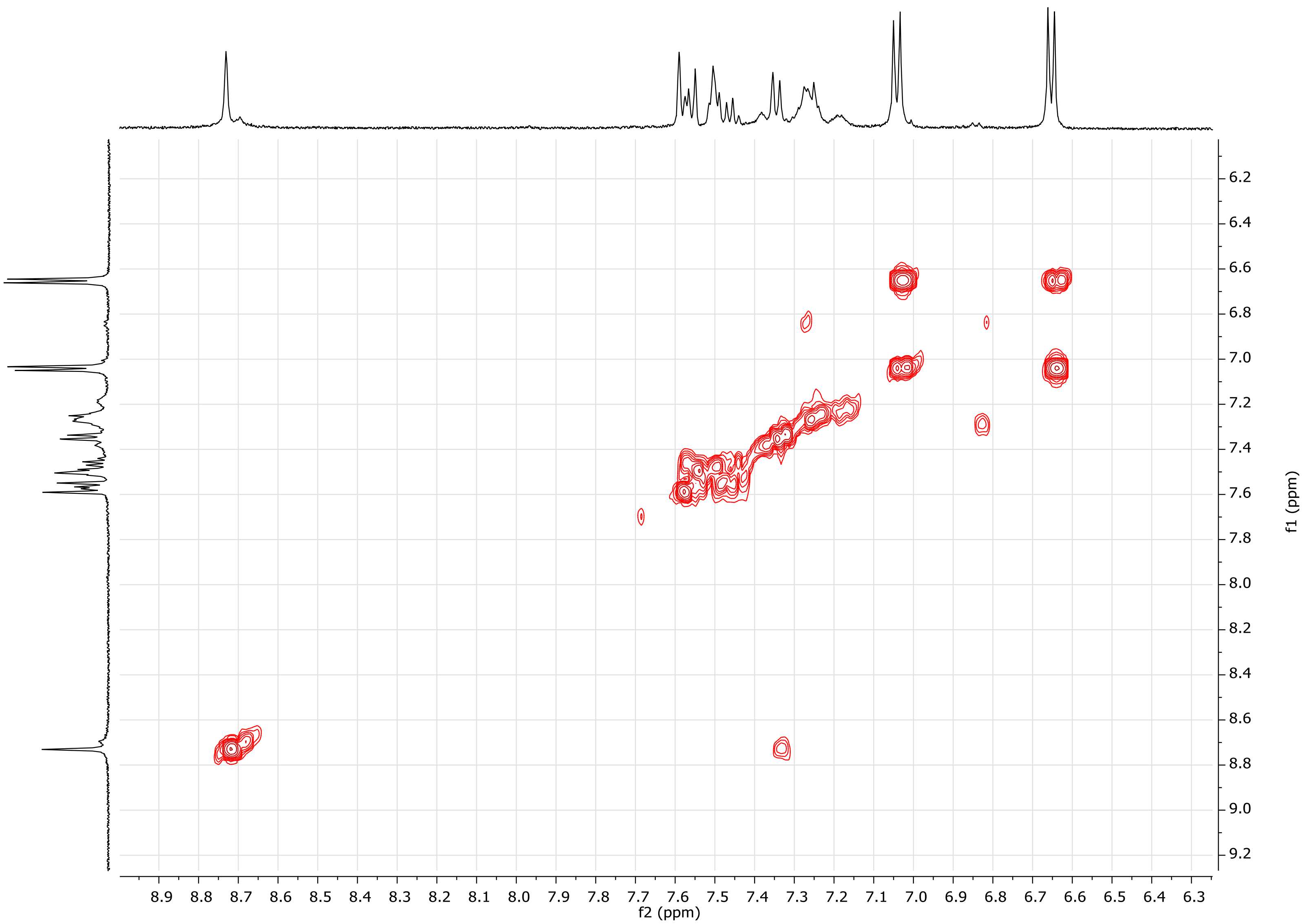

HSQC aromatic region (**S16** and **S17**)

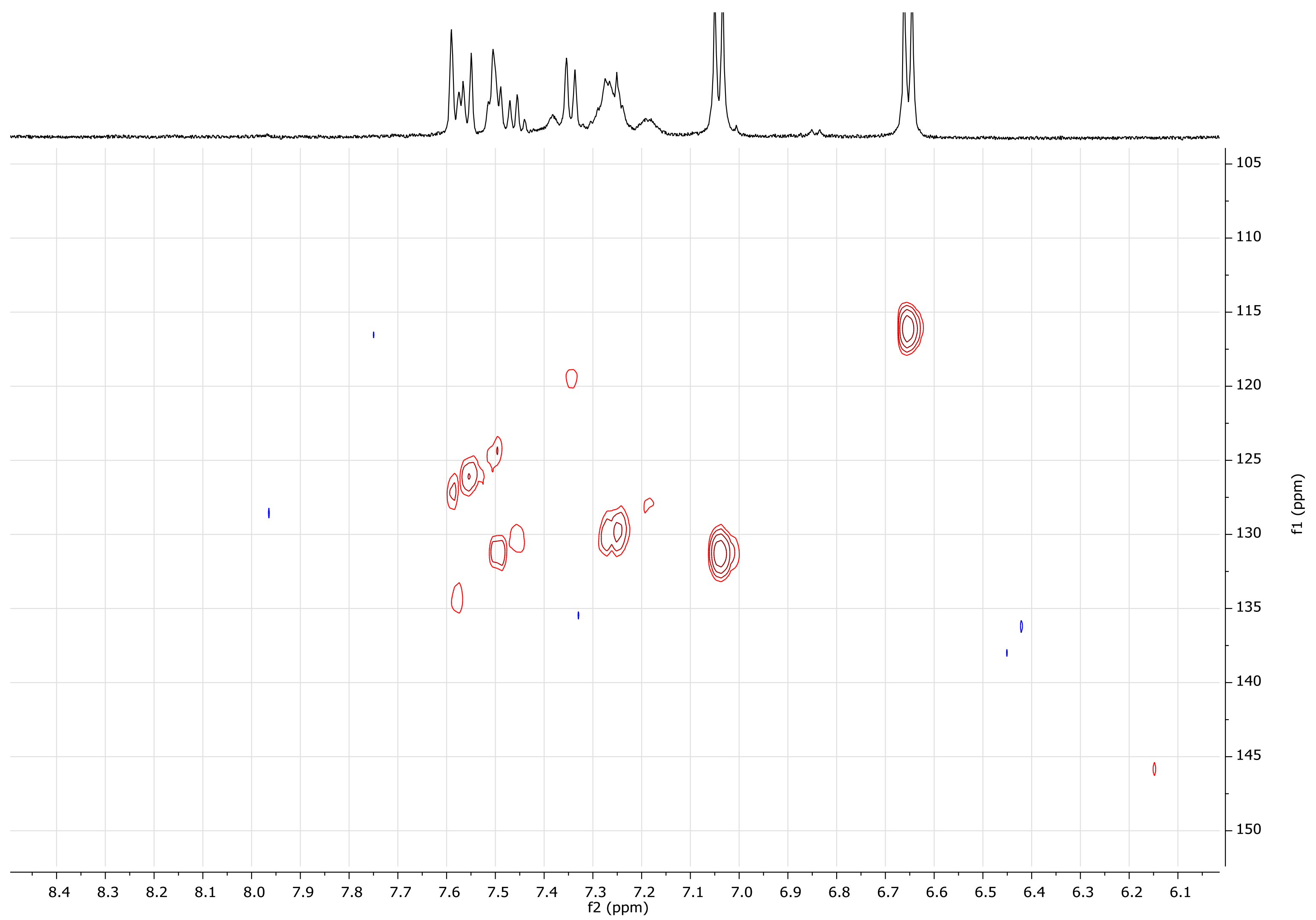

HMBC (**S16** and **S17**)

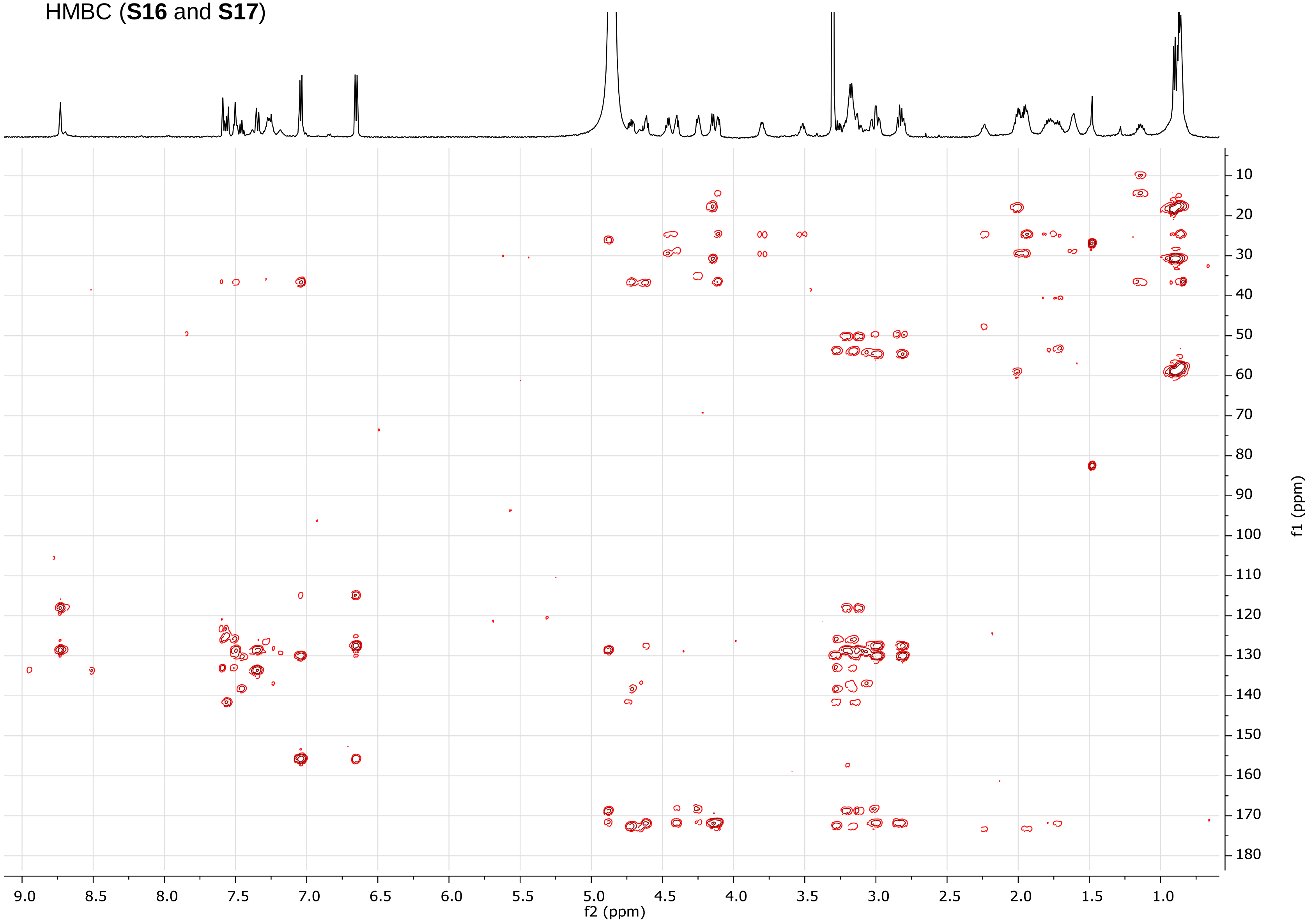

^{1}H NMR (500 MHz, Methanol-d$_4$)

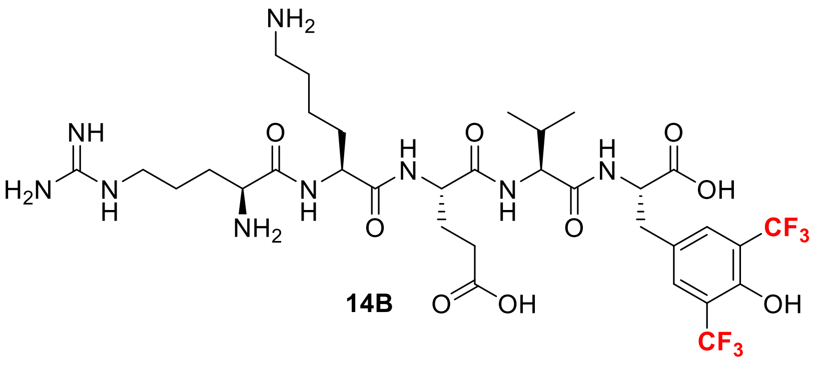

14B

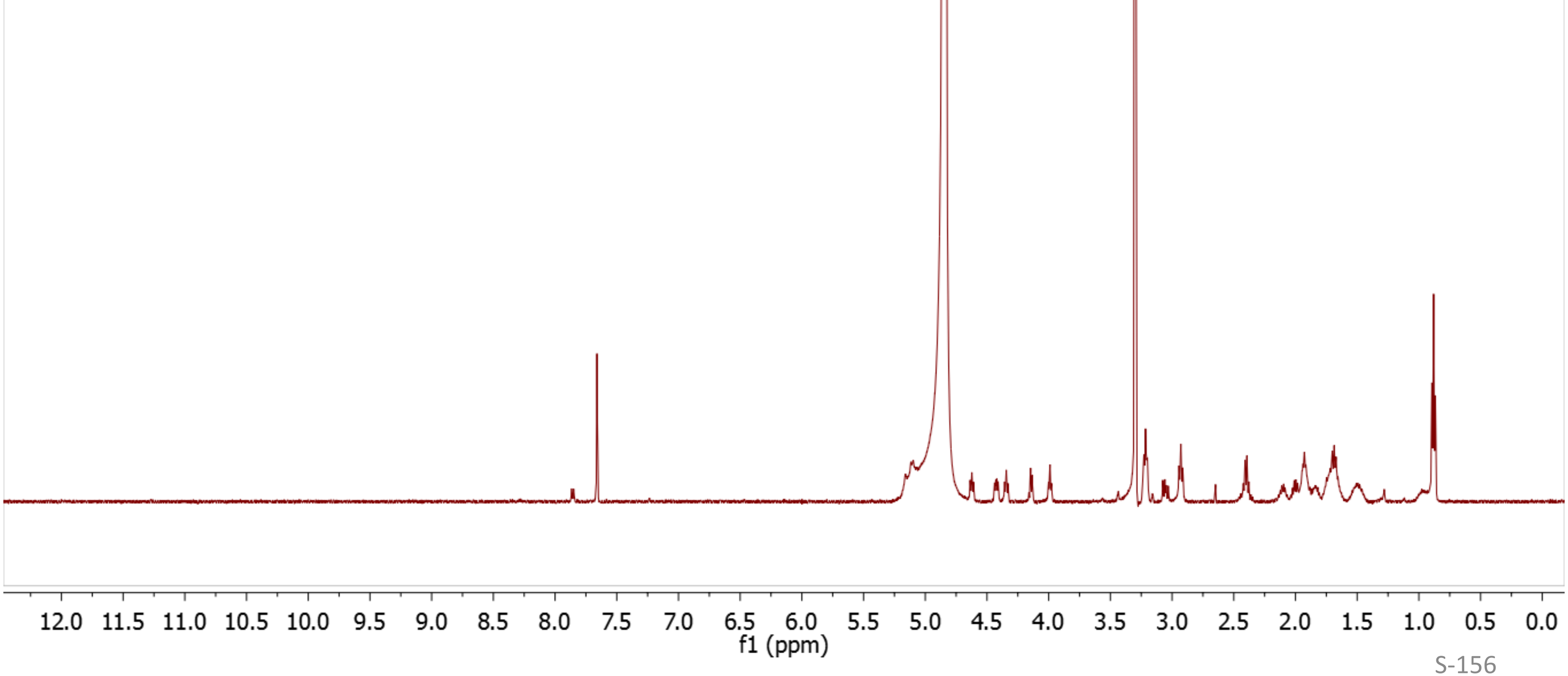

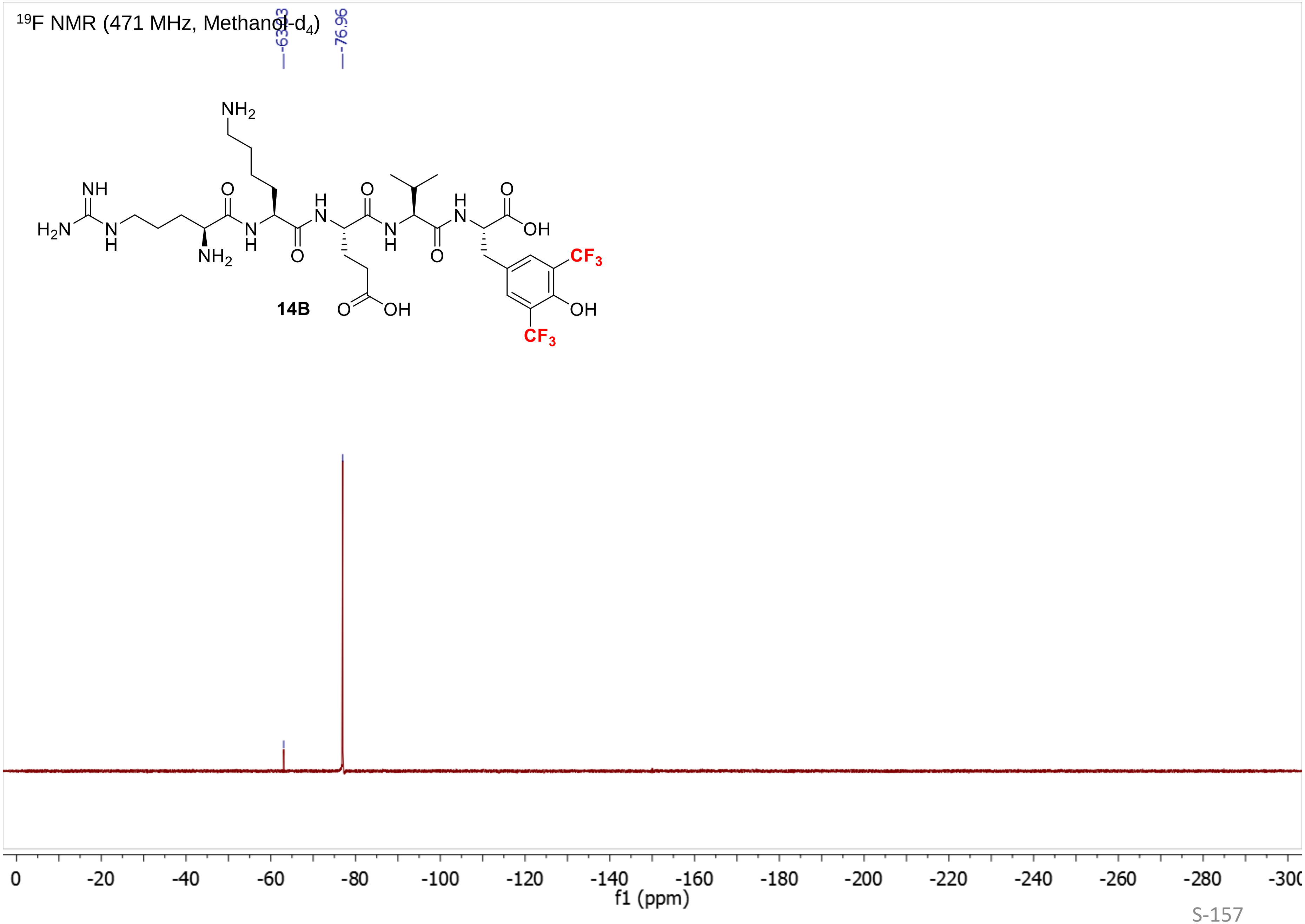

19F NMR (471 MHz, Methanol-d4)
-63.13
-76.96
NH2
NH
H2N
N
H
O
N
H
NH2
O
H
N
O
N
H
O
H
N
O
OH
CF3
OH
CF3
14B
O
OH
0
-20
-40
-60
-80
-100
-120
-140
-160
-180
-200
-220
-240
-260
-280
f1 (ppm)

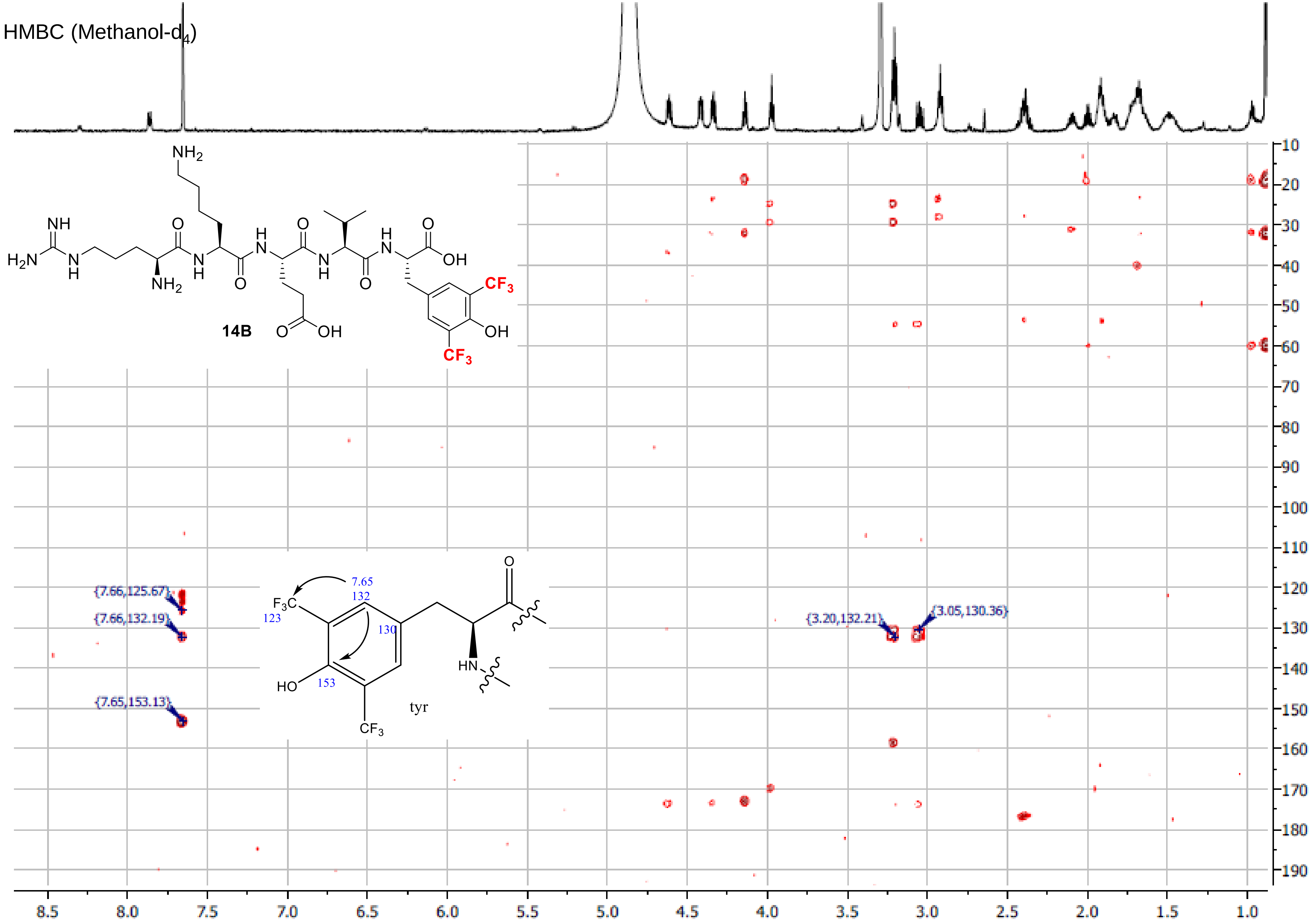

HMBC (Methanol-d_4)
14B
7.65
132
123
130
153
tyr
{7.66,125.67}
{7.66,132.19}
{7.65,153.13}
{3.20,132.21}
{3.05,130.36}

^{1}H NMR (500MHz, Methanol-d_4)

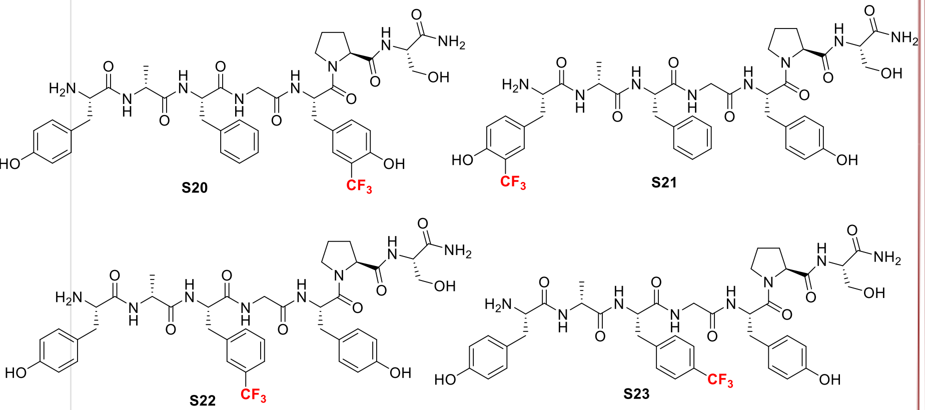

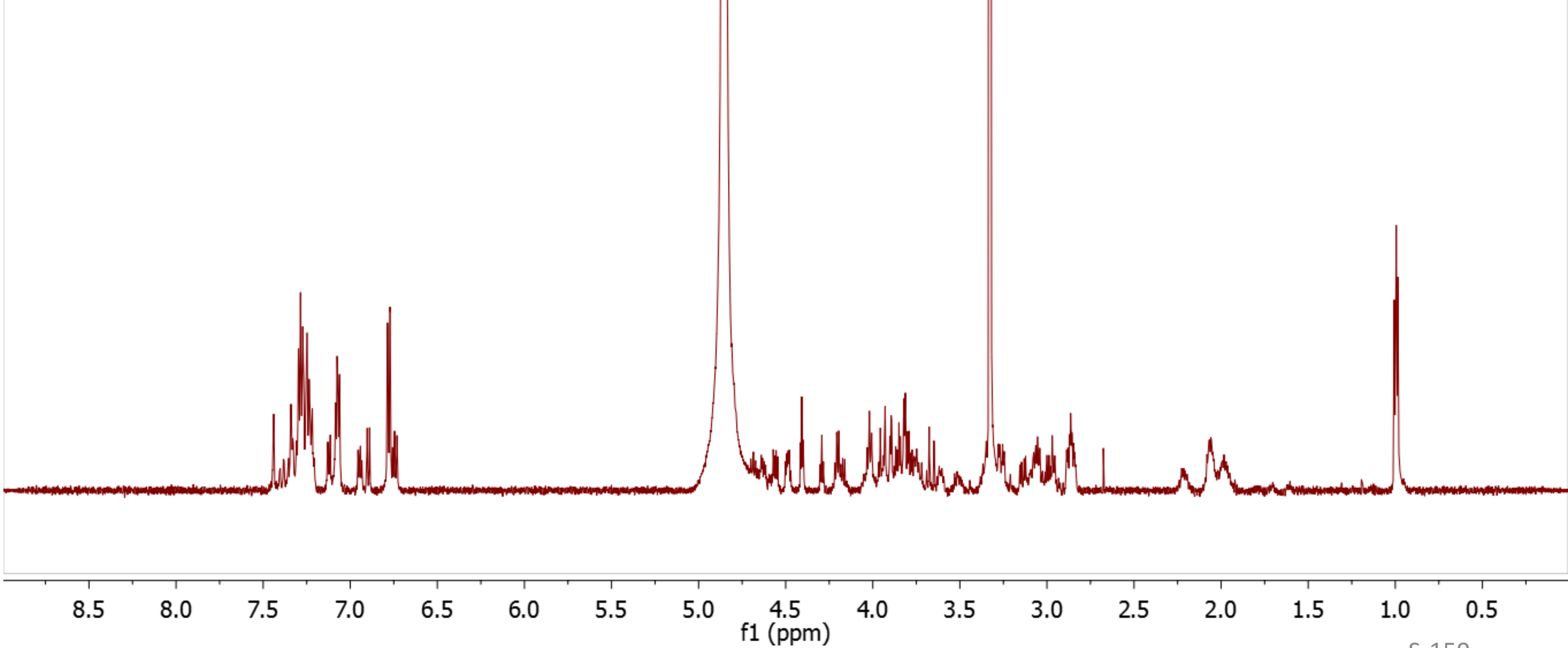

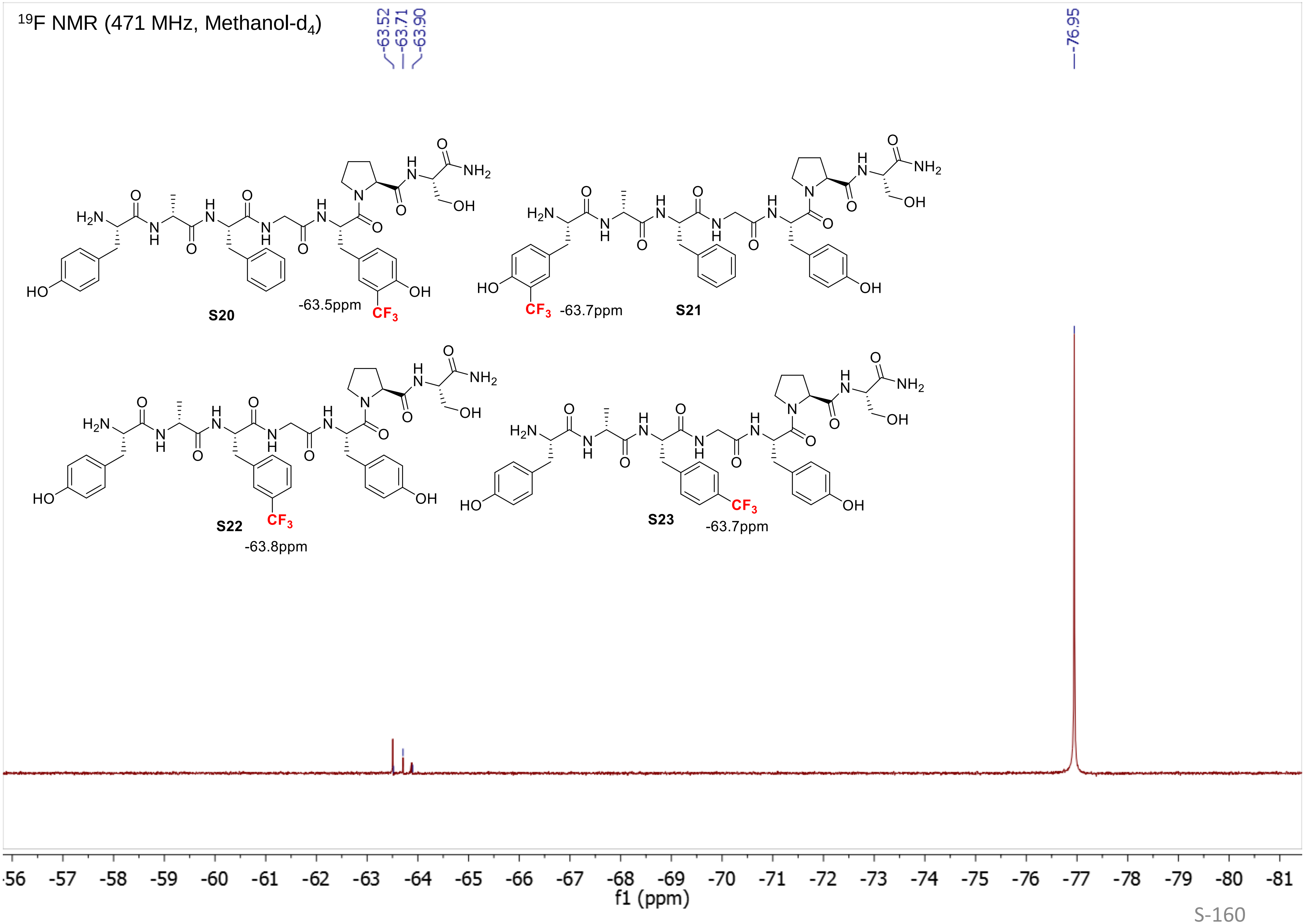

^{19}F NMR (471 MHz, Methanol-d$_4$)
-63.52
-63.71
-63.90
-76.95
S20
-63.5ppm
CF$_3$
S21
CF$_3$ -63.7ppm
S22
CF$_3$
-63.8ppm
S23
CF$_3$
-63.7ppm
f1 (ppm)

COSY (Methanol-d4)

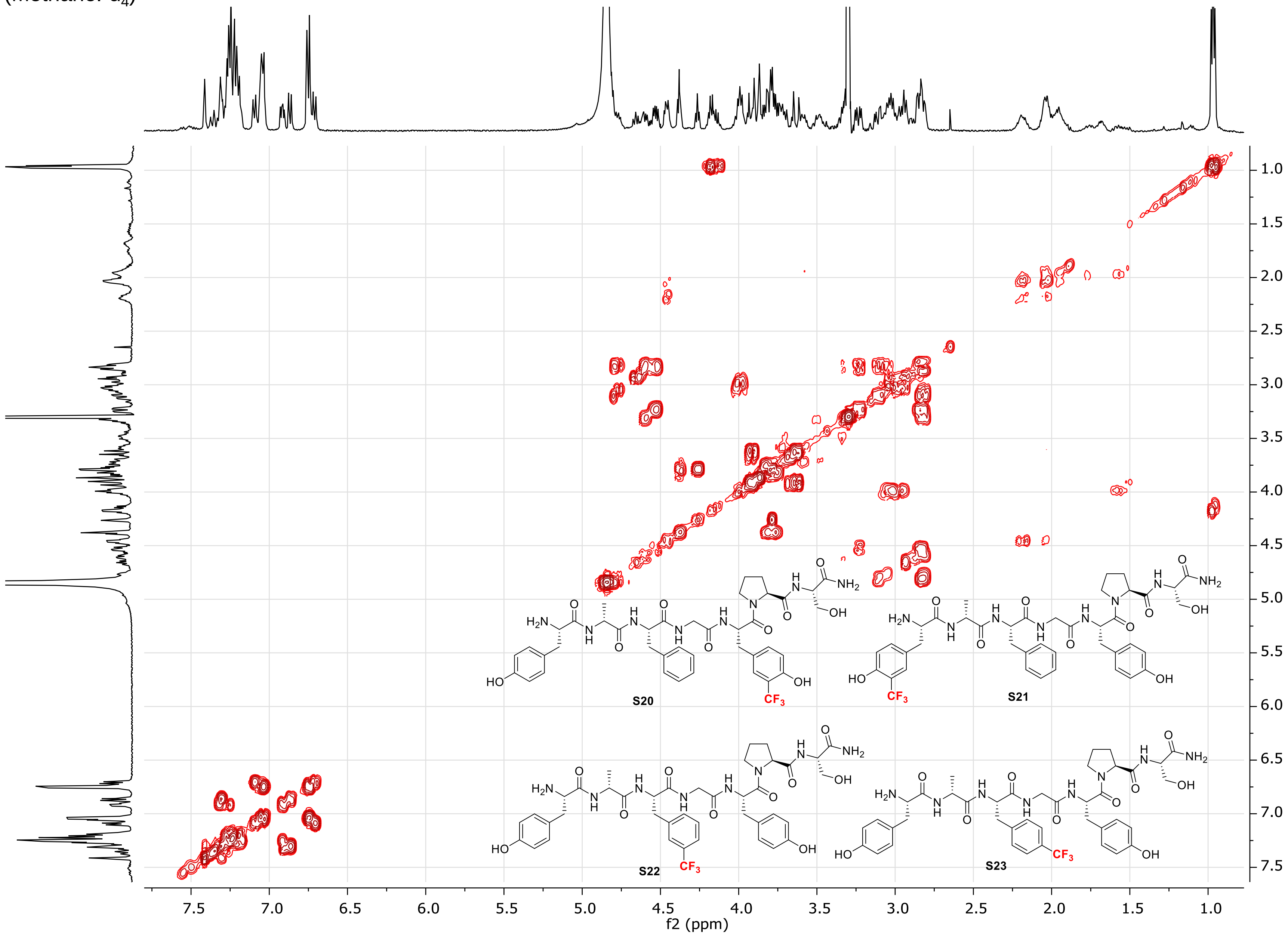

HSQC

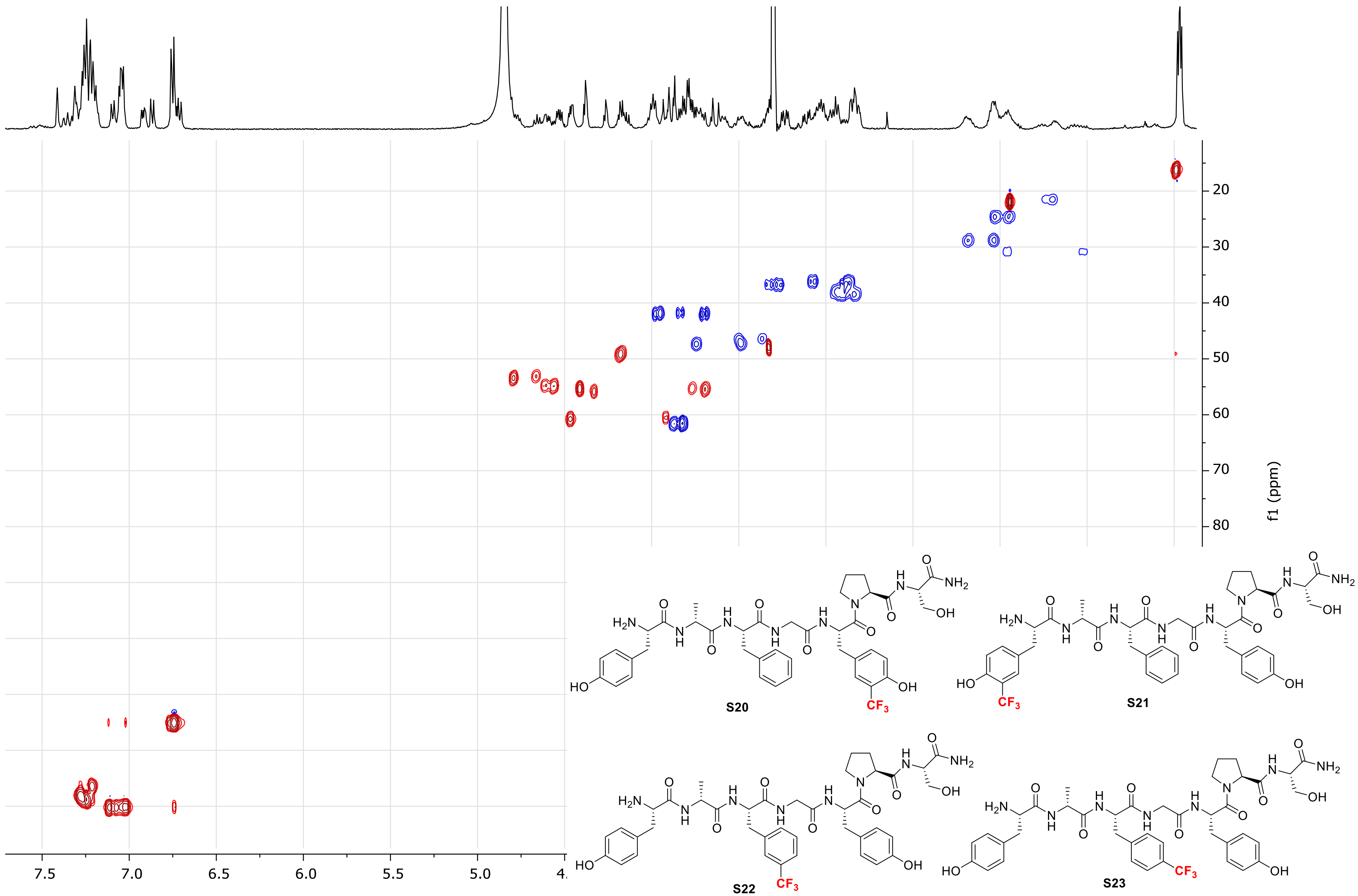

S20 S21 S22 S23

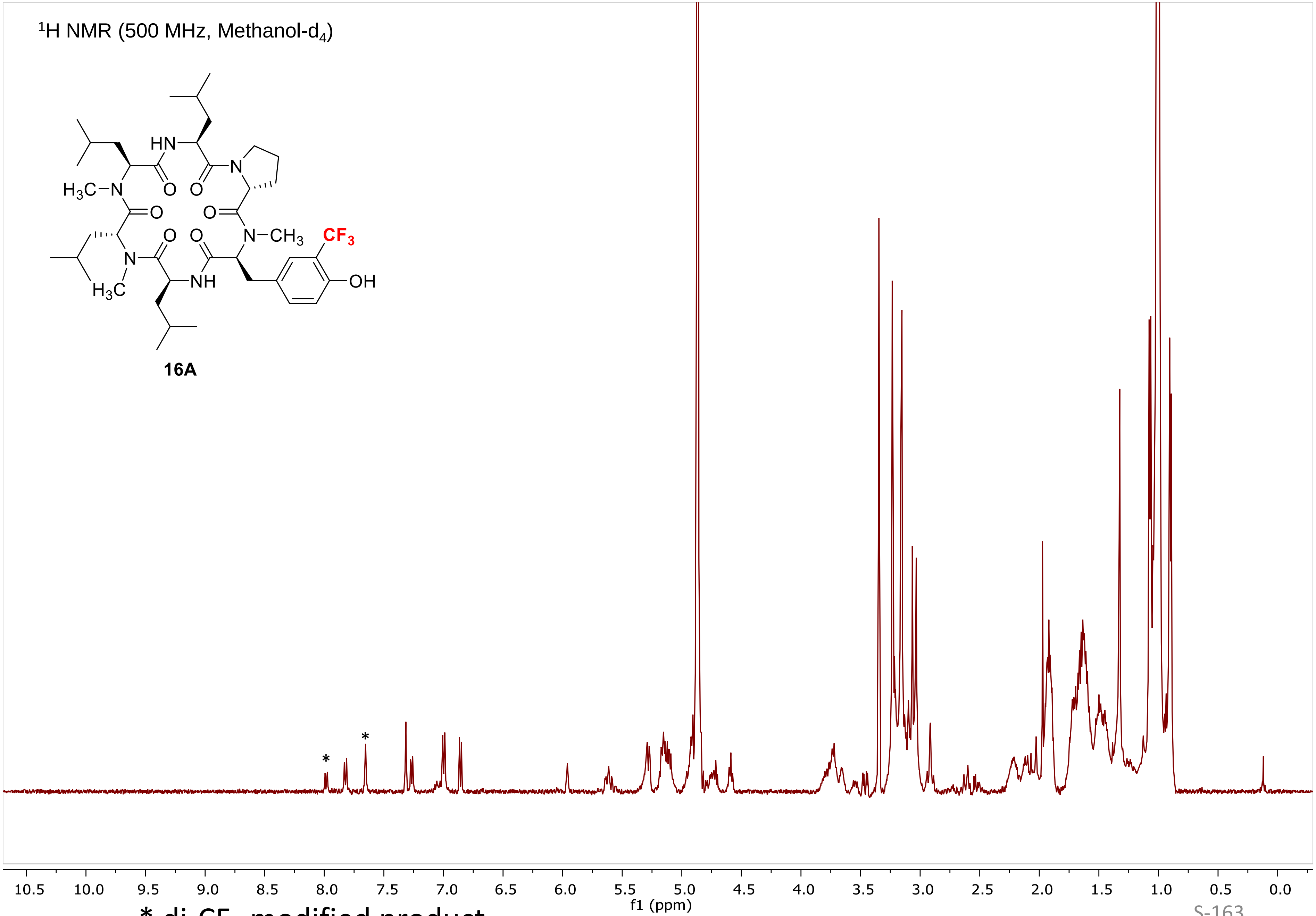

* di-CF_3 modified product

Overlay of ^{1}HNMR of **16A** and compound **16**

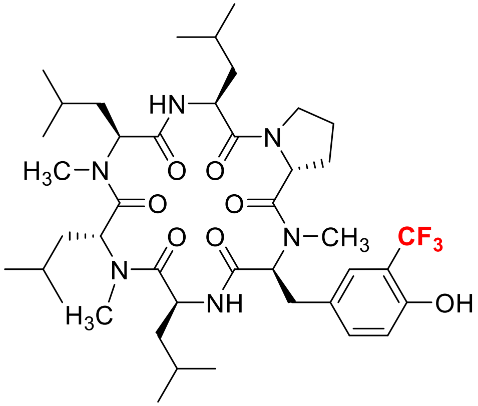

16A

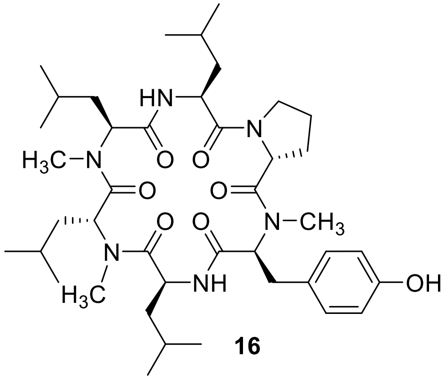

16

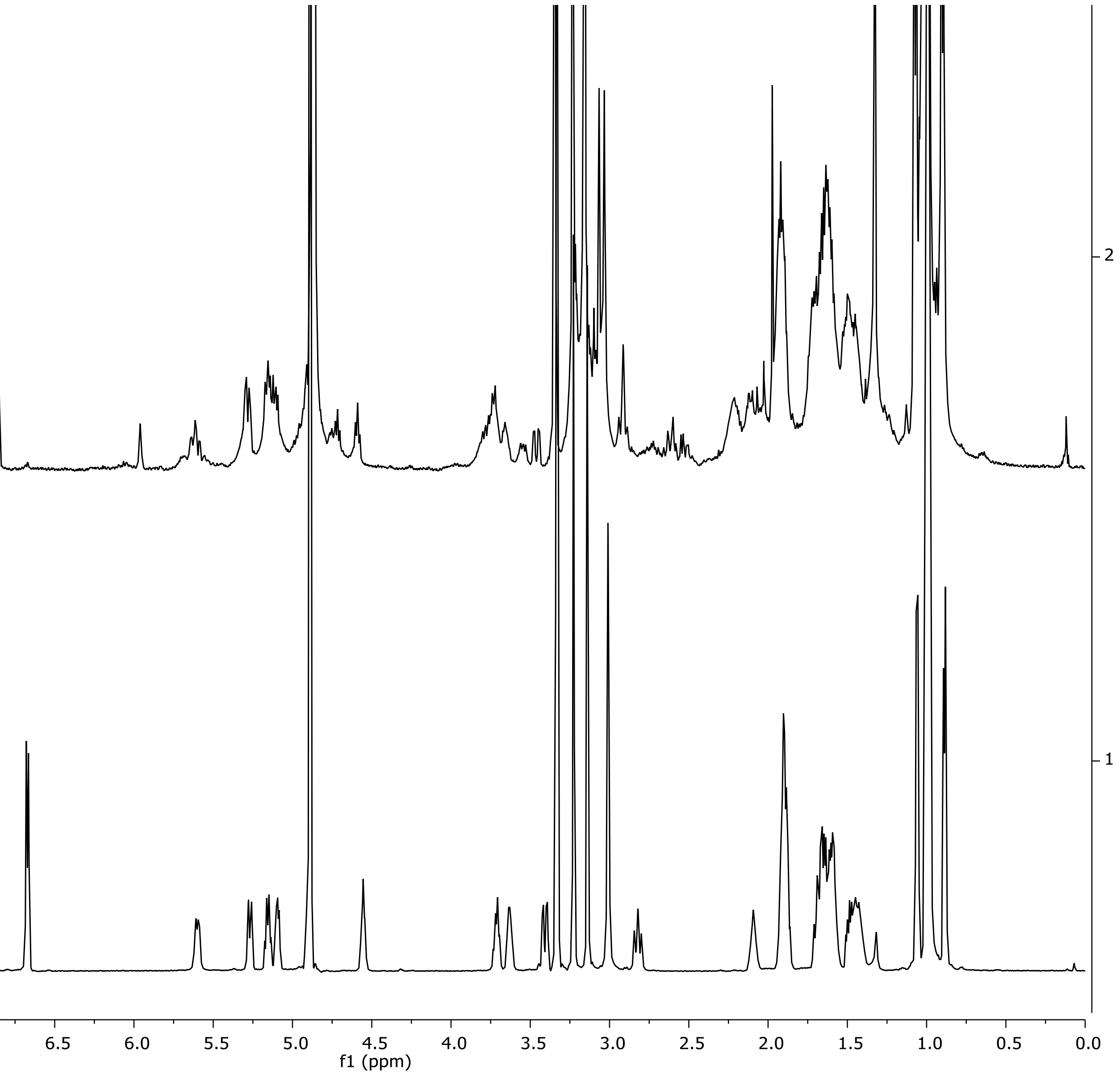

HSQC (**16A**)

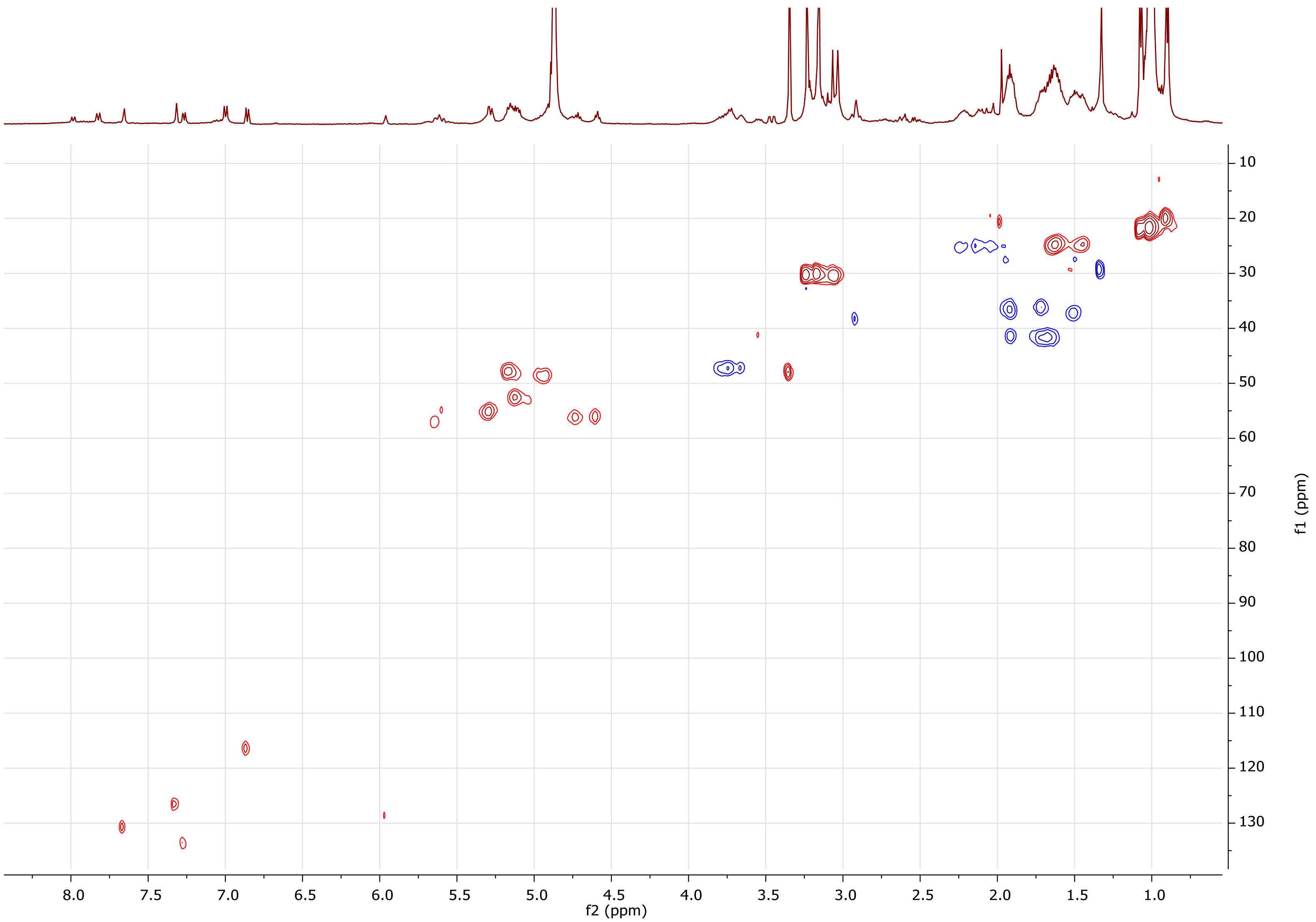
f2 (ppm)
f1 (ppm)
8.0
7.5
7.0
6.5
6.0
5.5
5.0
4.5
4.0
3.5
3.0
2.5
2.0
1.5
1.0
10
20
30
40
50
60
70
80
90
100
110
120
130

HSQC aromatic region

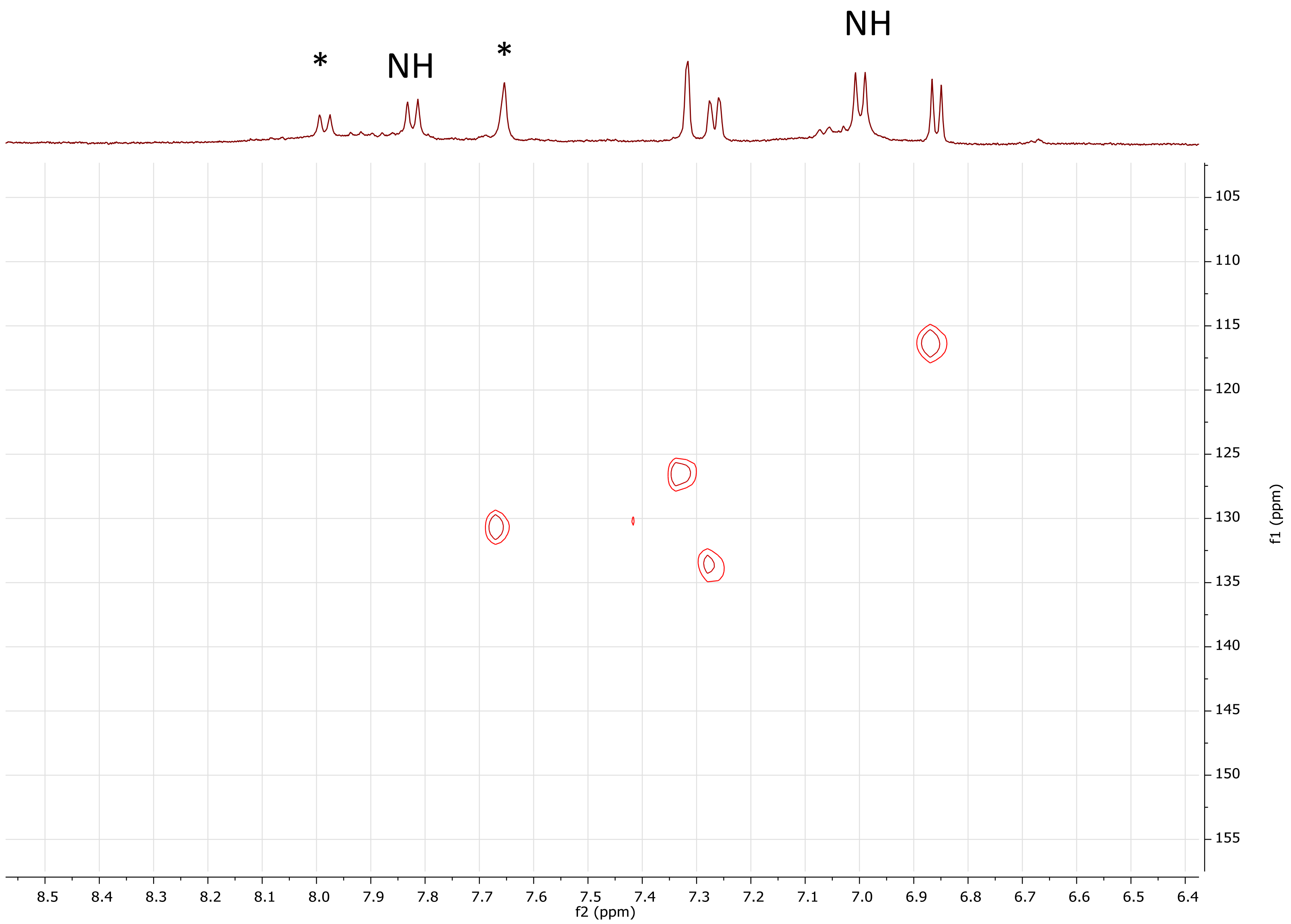

* di-CF_3 modified product

HSQC aromatic region

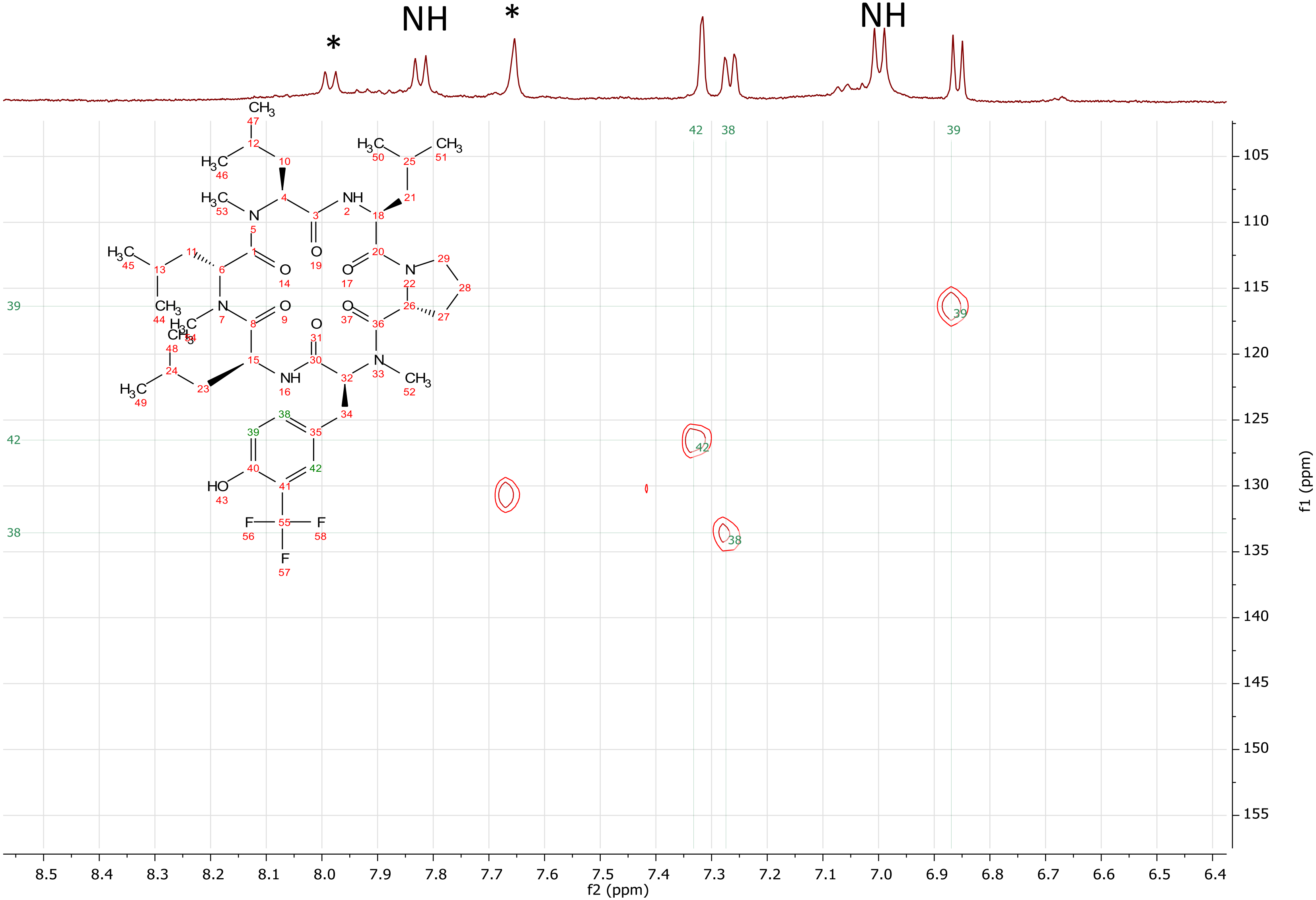

* di-CF_3 modified product

COSY aromatic region

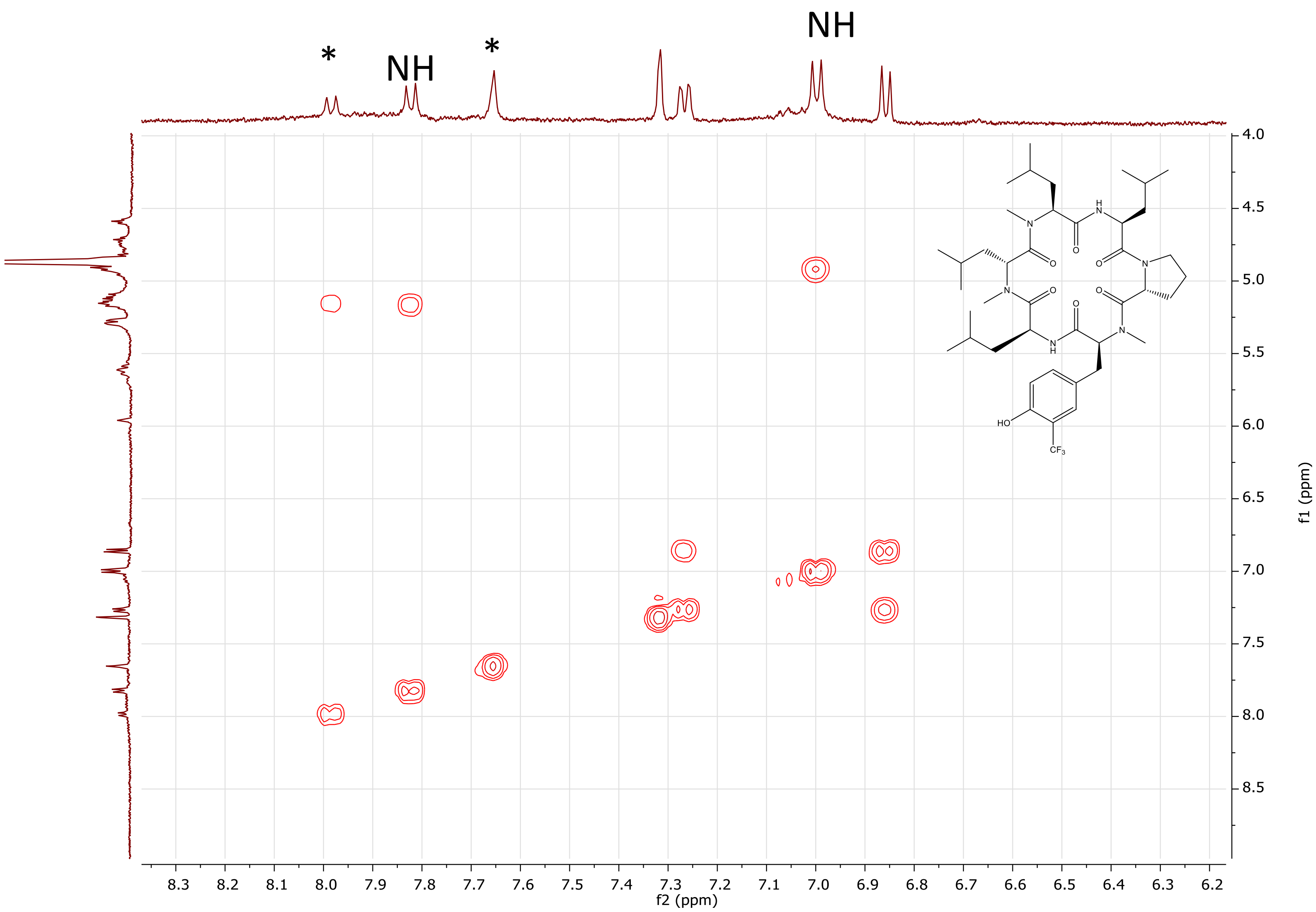

* di-CF_3 modified product

^{19}F NMR (471 MHz, Methanol-d_4)

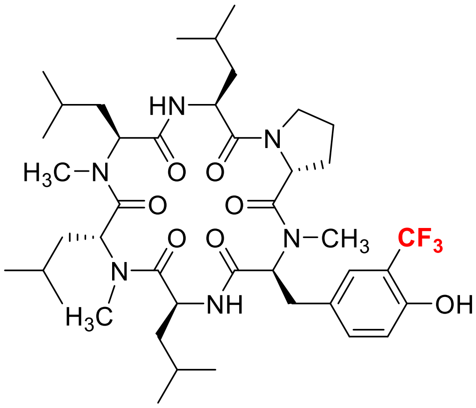

16A

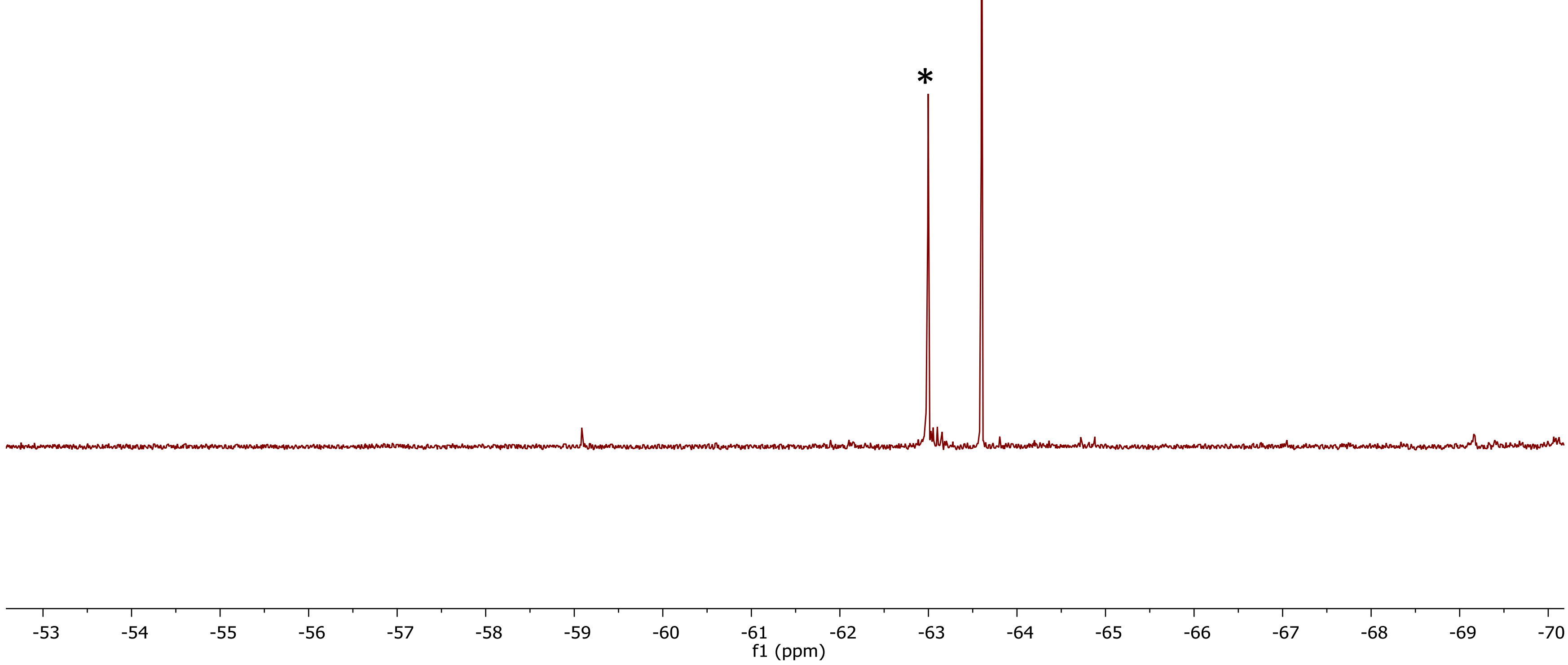

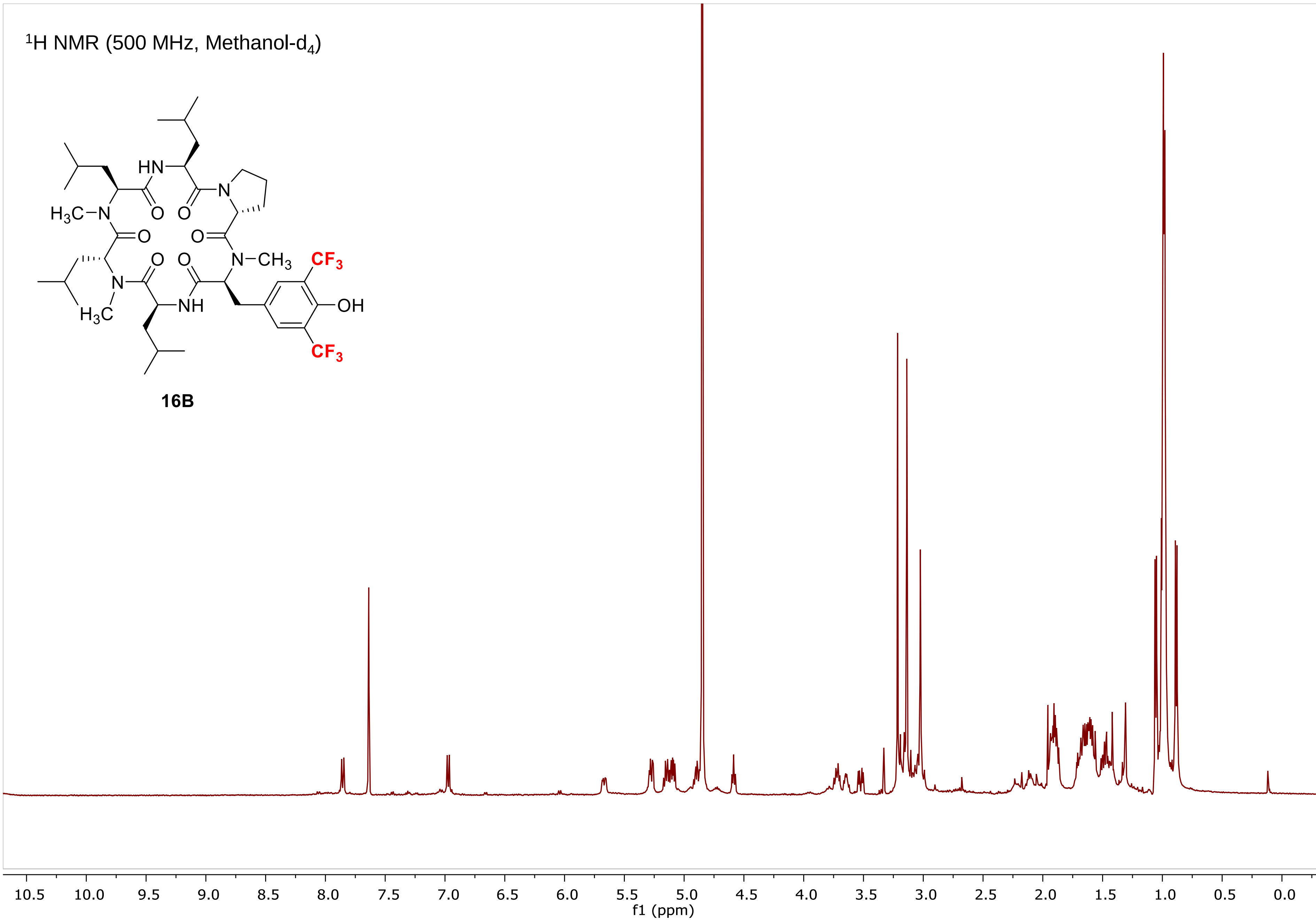
^1H NMR (500 MHz, Methanol-d$_4$)
16B
10.5 10.0 9.5 9.0 8.5 8.0 7.5 7.0 6.5 6.0 5.5 5.0 4.5 4.0 3.5 3.0 2.5 2.0 1.5 1.0 0.5 0.0
f1 (ppm)

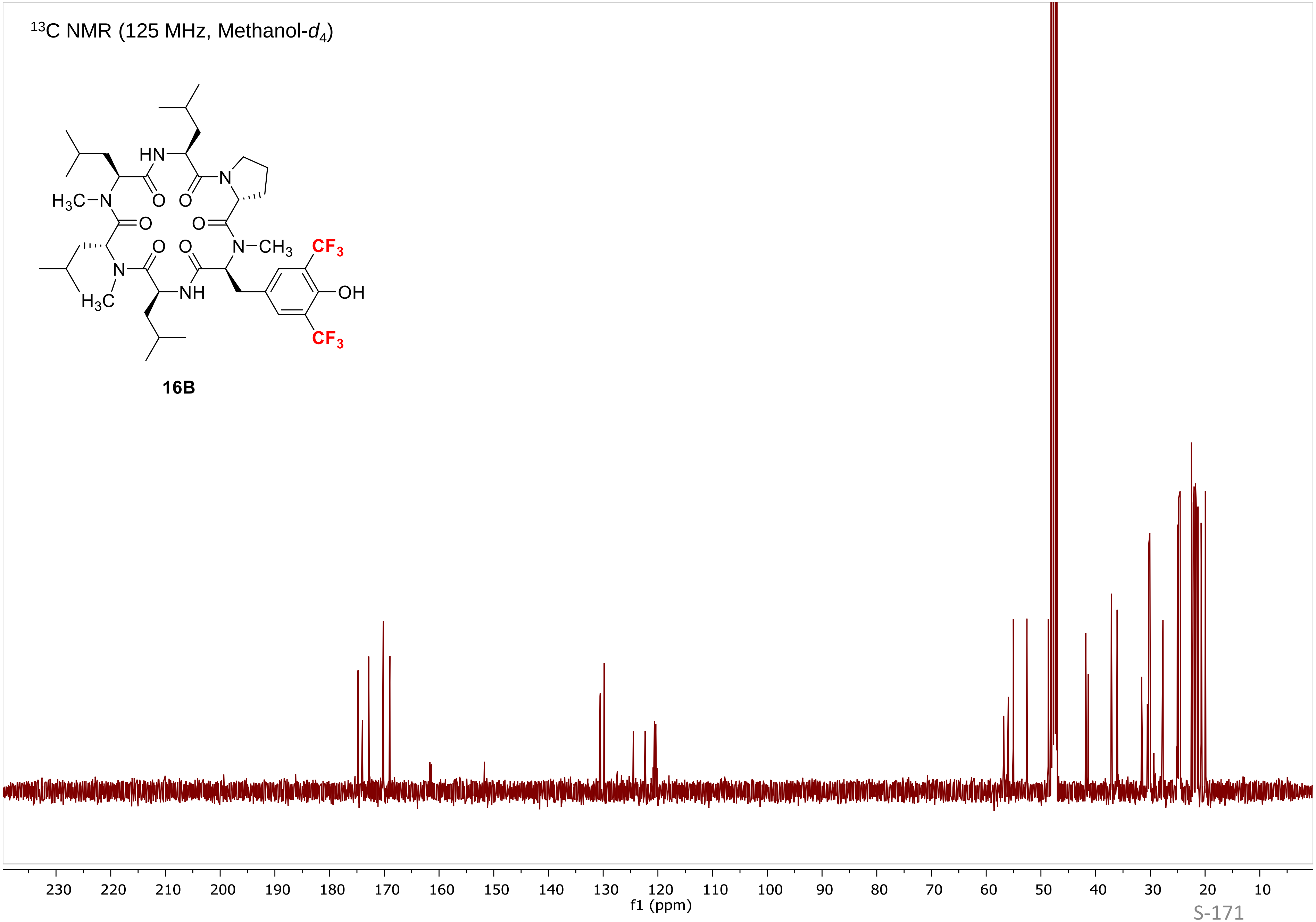

^{13}C NMR (125 MHz, Methanol-d_4)
HN
N
O
H3C–N
O
O
N–CH3
CF3
OH
CF3
N
NH
H3C
16B
230 220 210 200 190 180 170 160 150 140 130 120 110 100 90 80 70 60 50 40 30 20 10
f1 (ppm)

^{19}F NMR (471 MHz, Methanol-d_4)

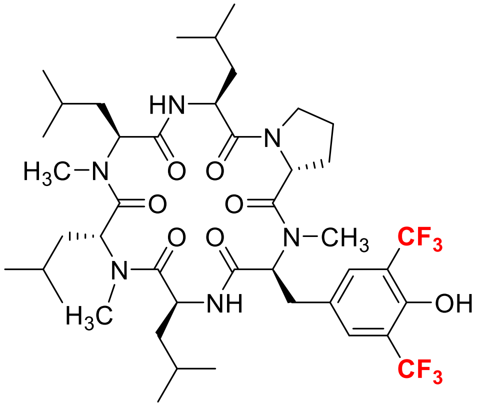

16B

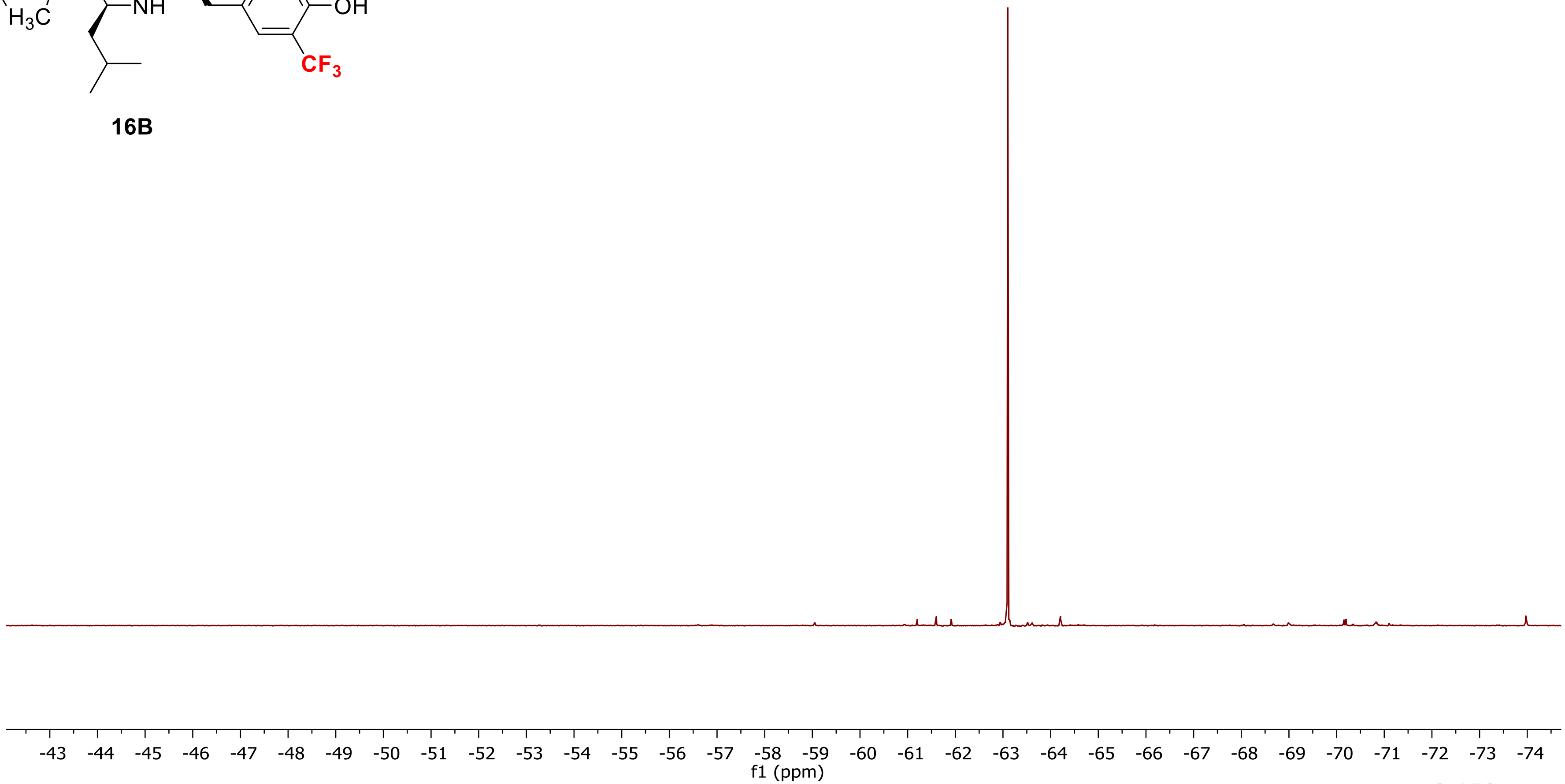

Overlay of ^{1}HNMR of **16B** and compound **16**

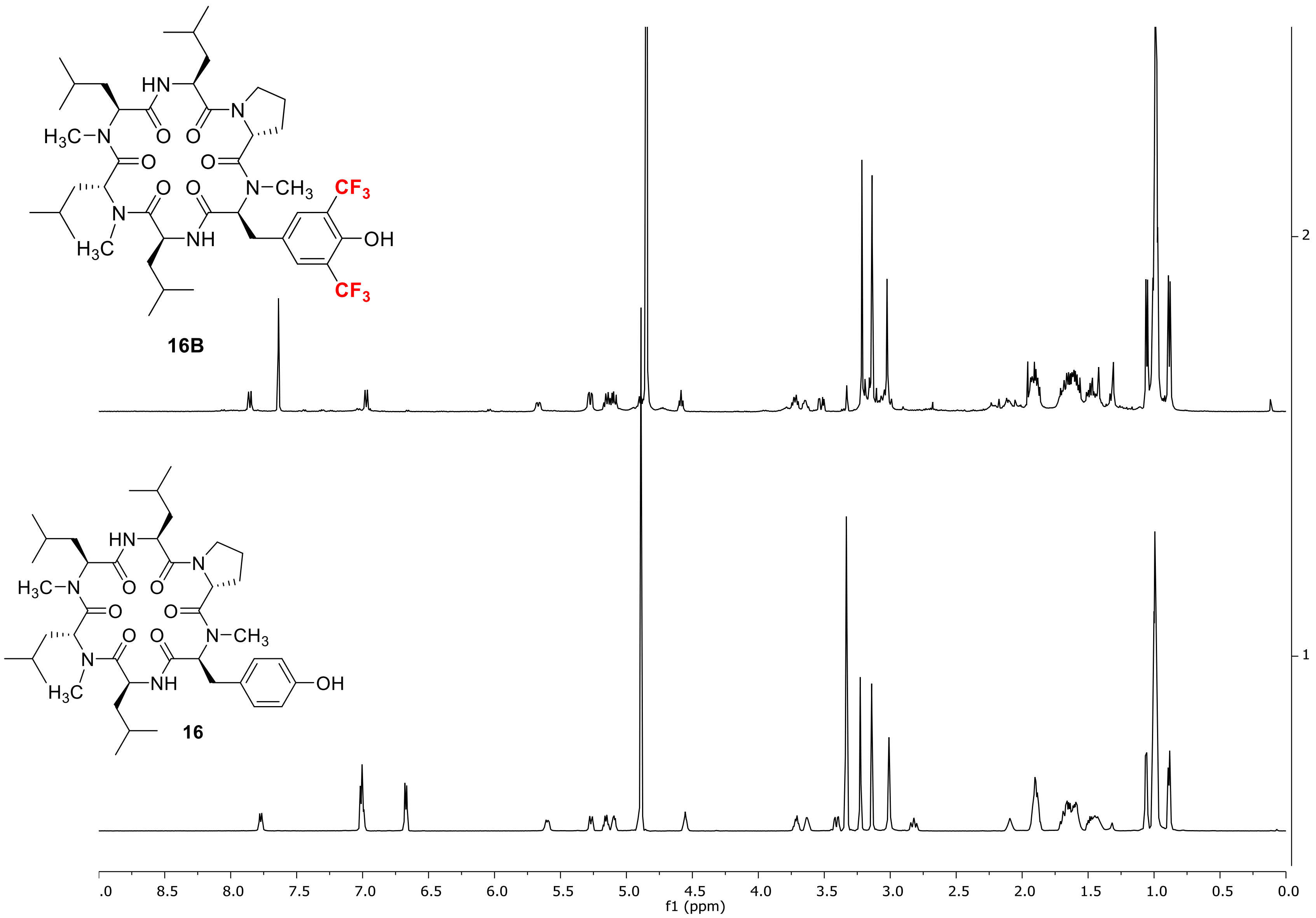

COSY (**16B**)

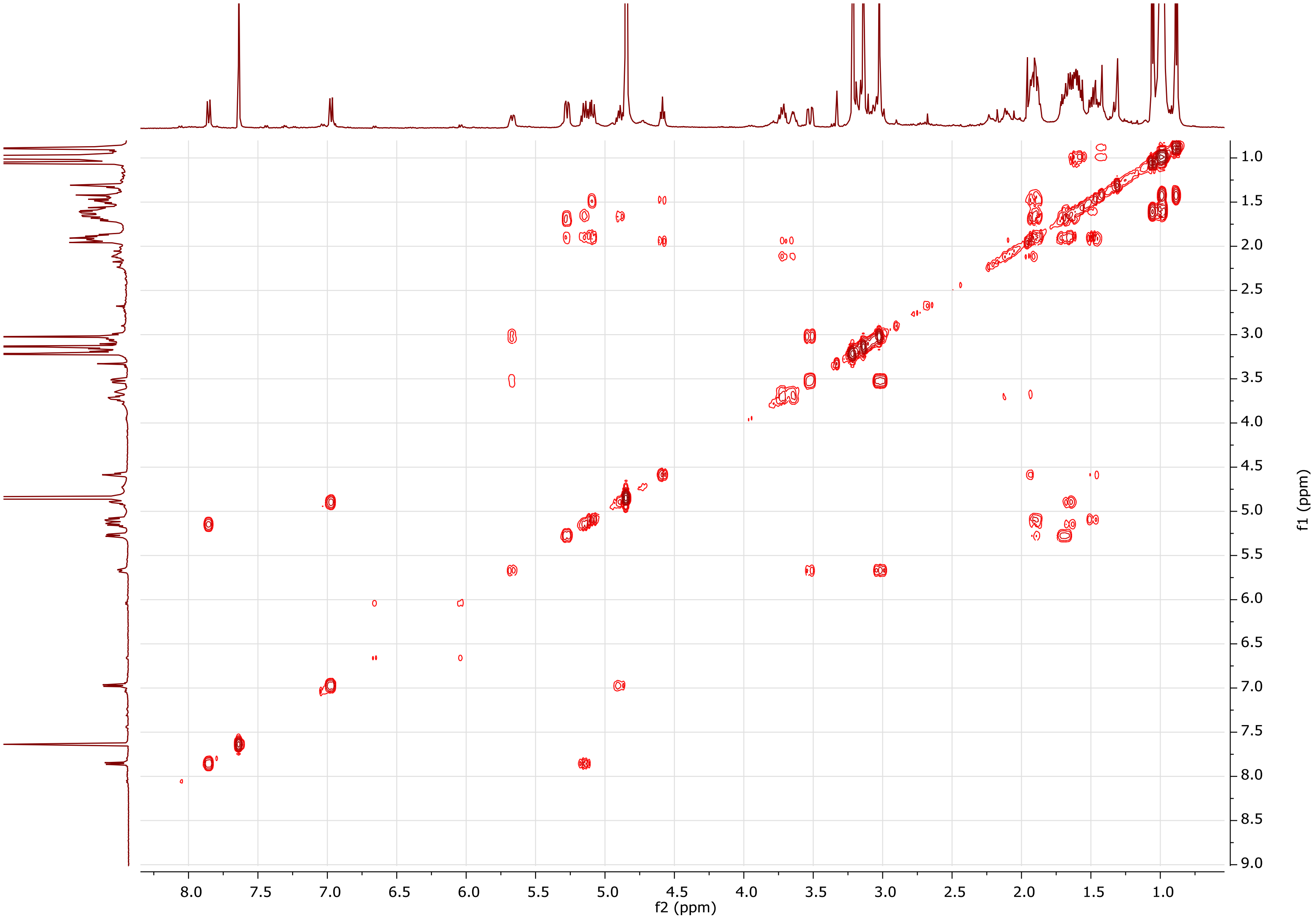

HSQC (**16B**)

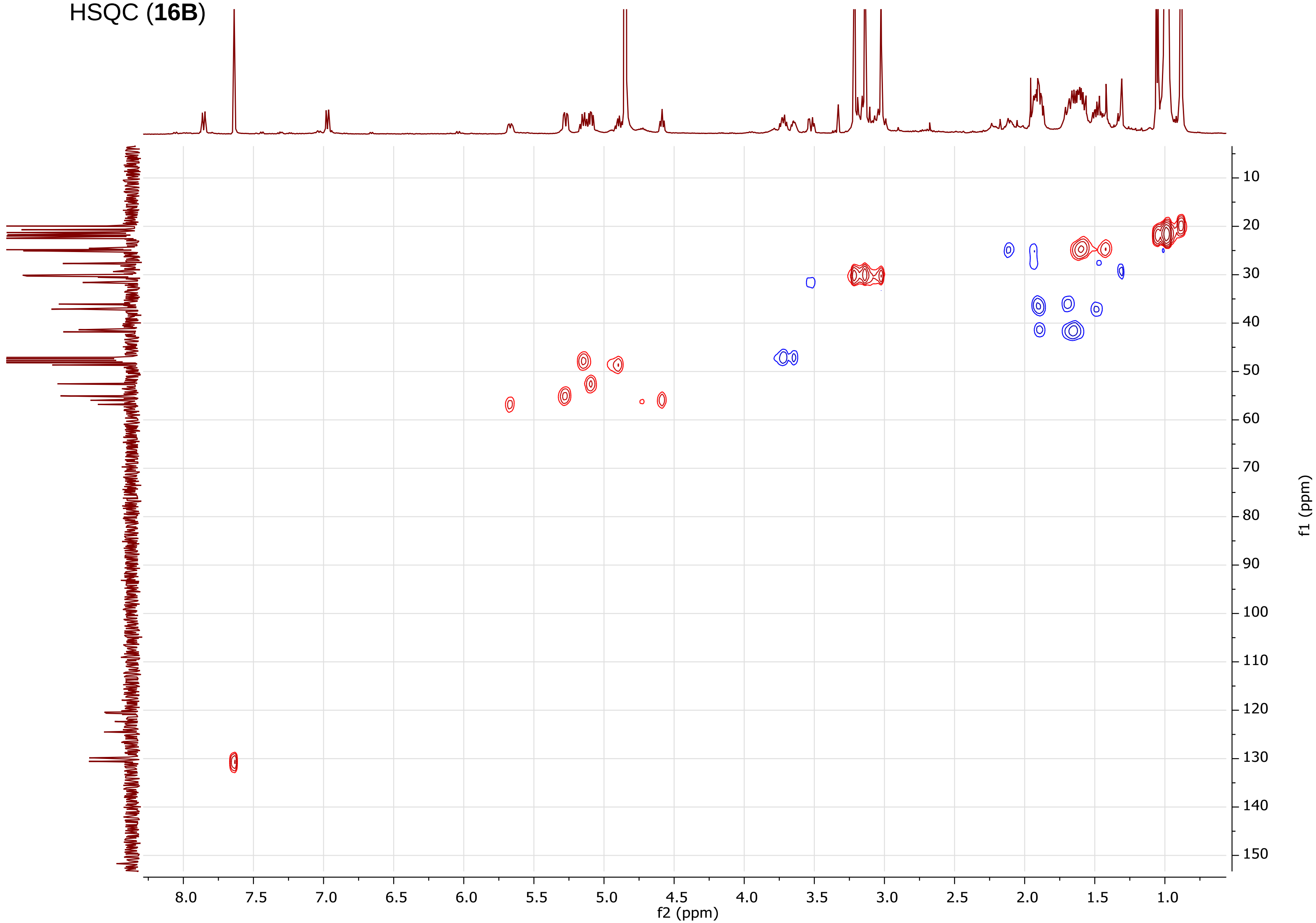

8.0
7.5
7.0
6.5
6.0
5.5
5.0
4.5
4.0
3.5
3.0
2.5
2.0
1.5
1.0
f2 (ppm)
10
20
30
40
50
60
70
80
90
100
110
120
130
140
150
f1 (ppm)

HMBC (**16B**)

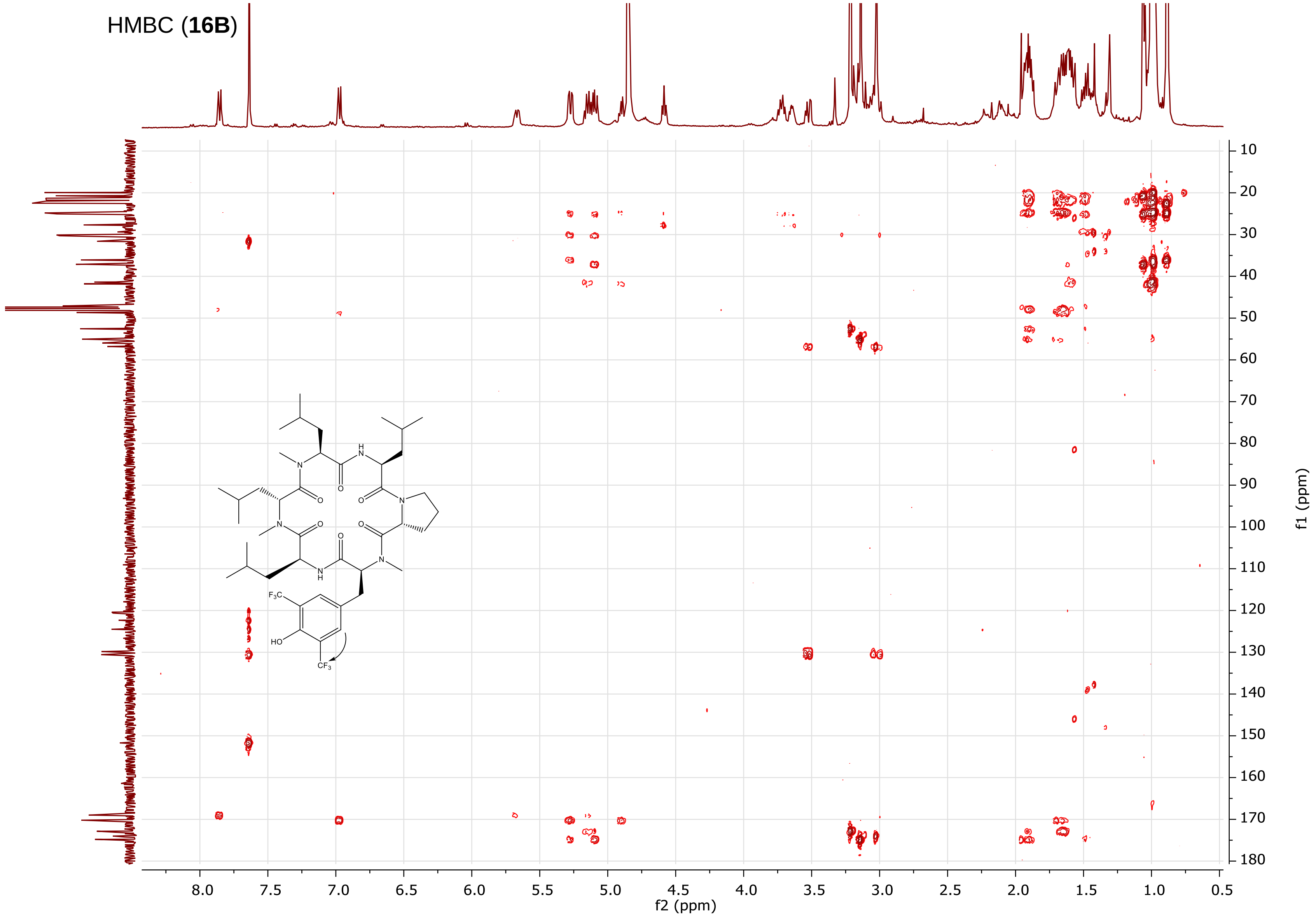

^{1}H NMR (500 MHz, Methanol-d_4)

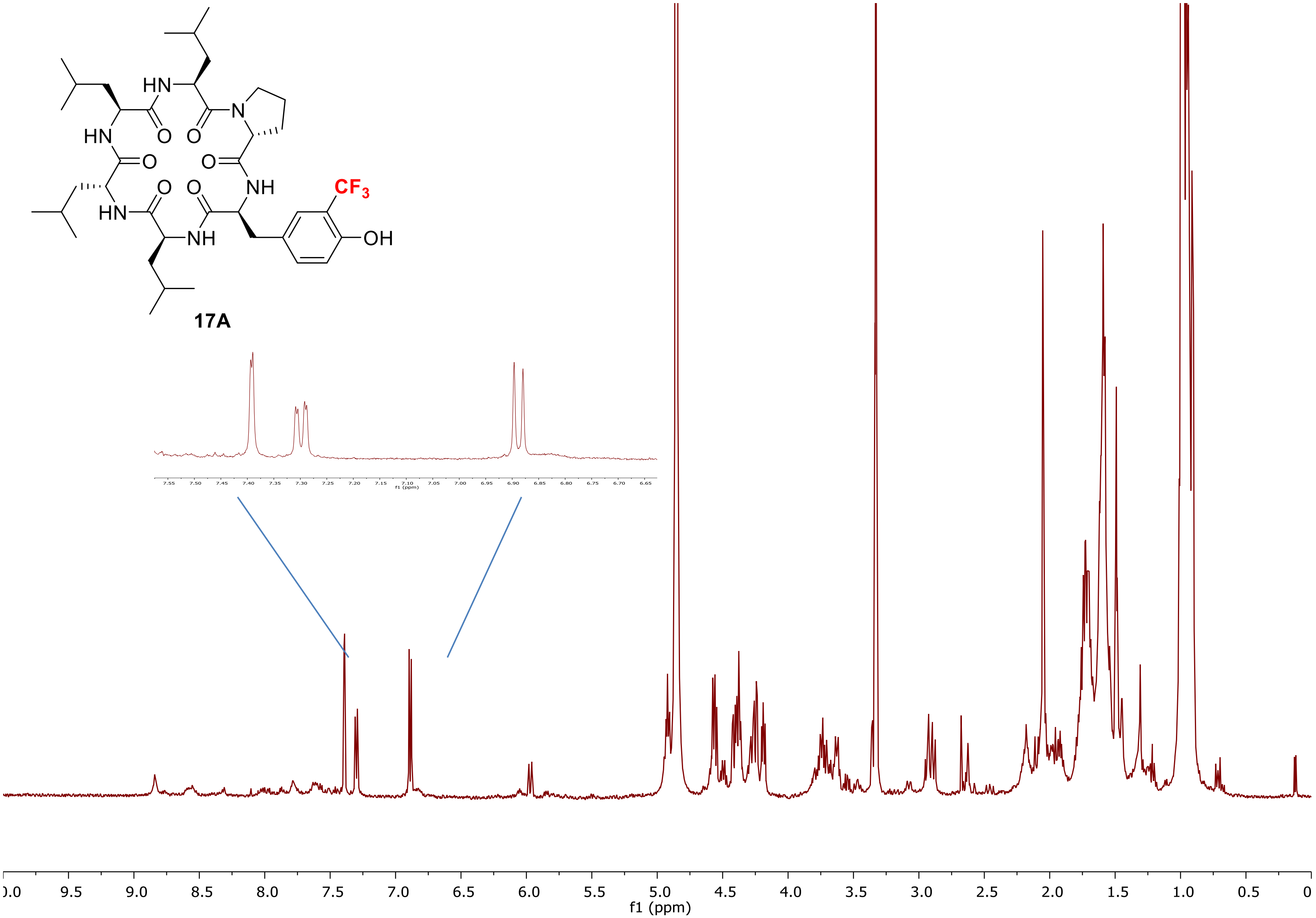

^{19}F NMR (471MHz, Methanol-d_4)

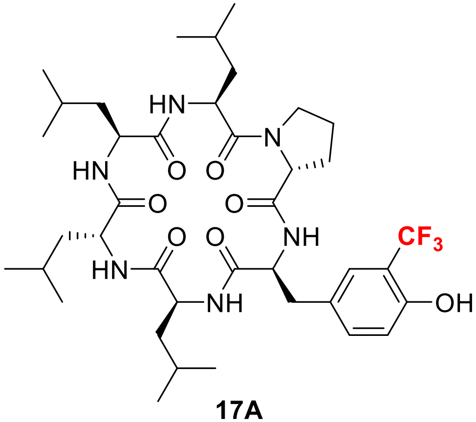

17A

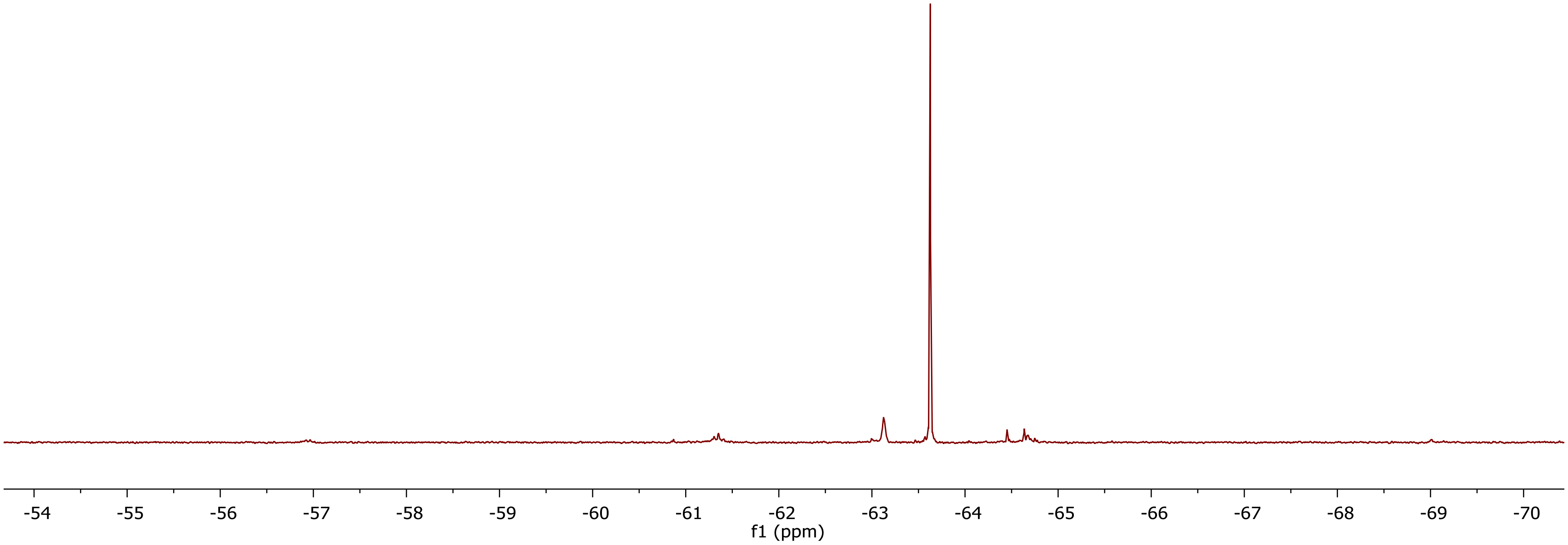

COSY (**17A)**

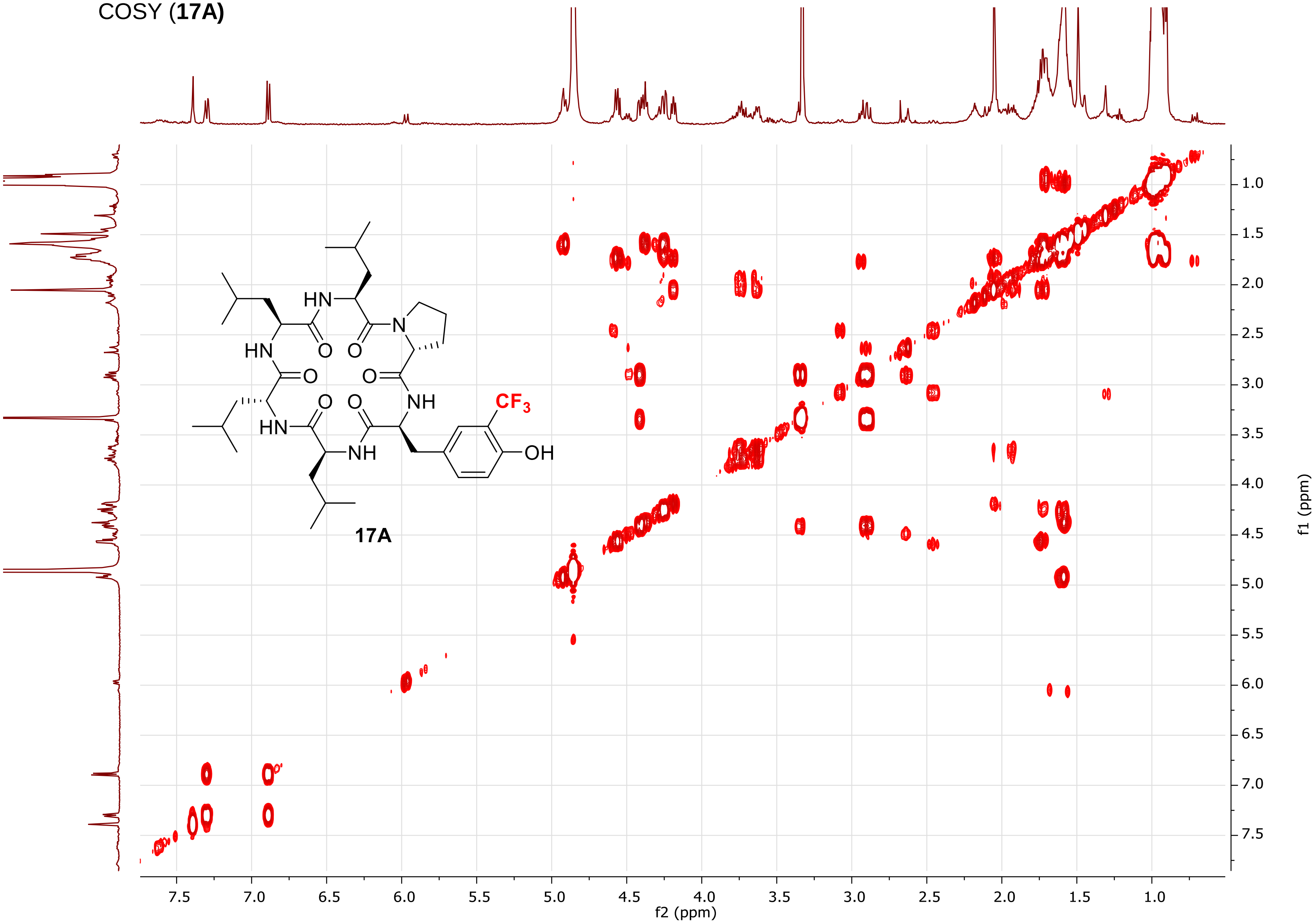

HSQC (**17A**)

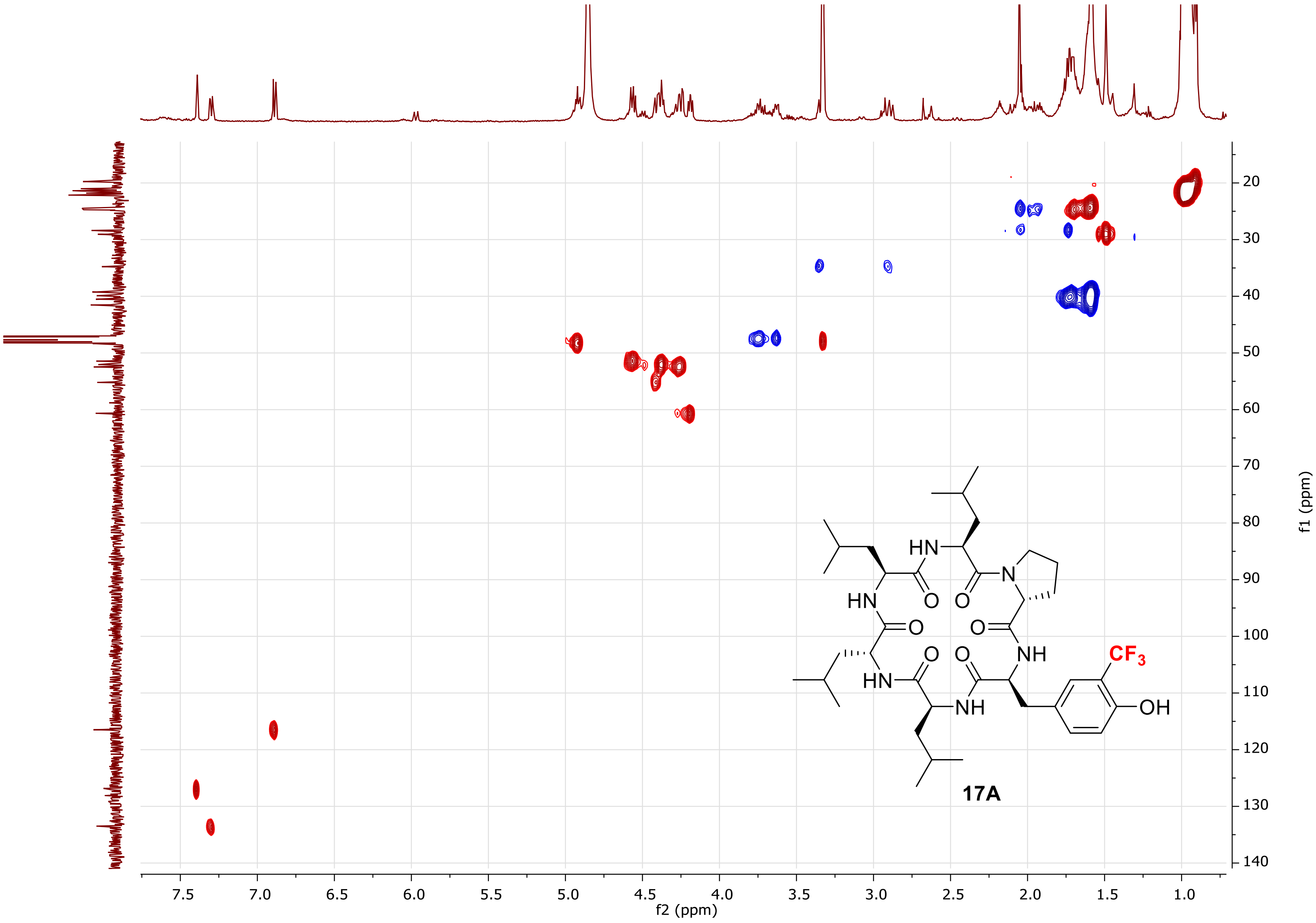

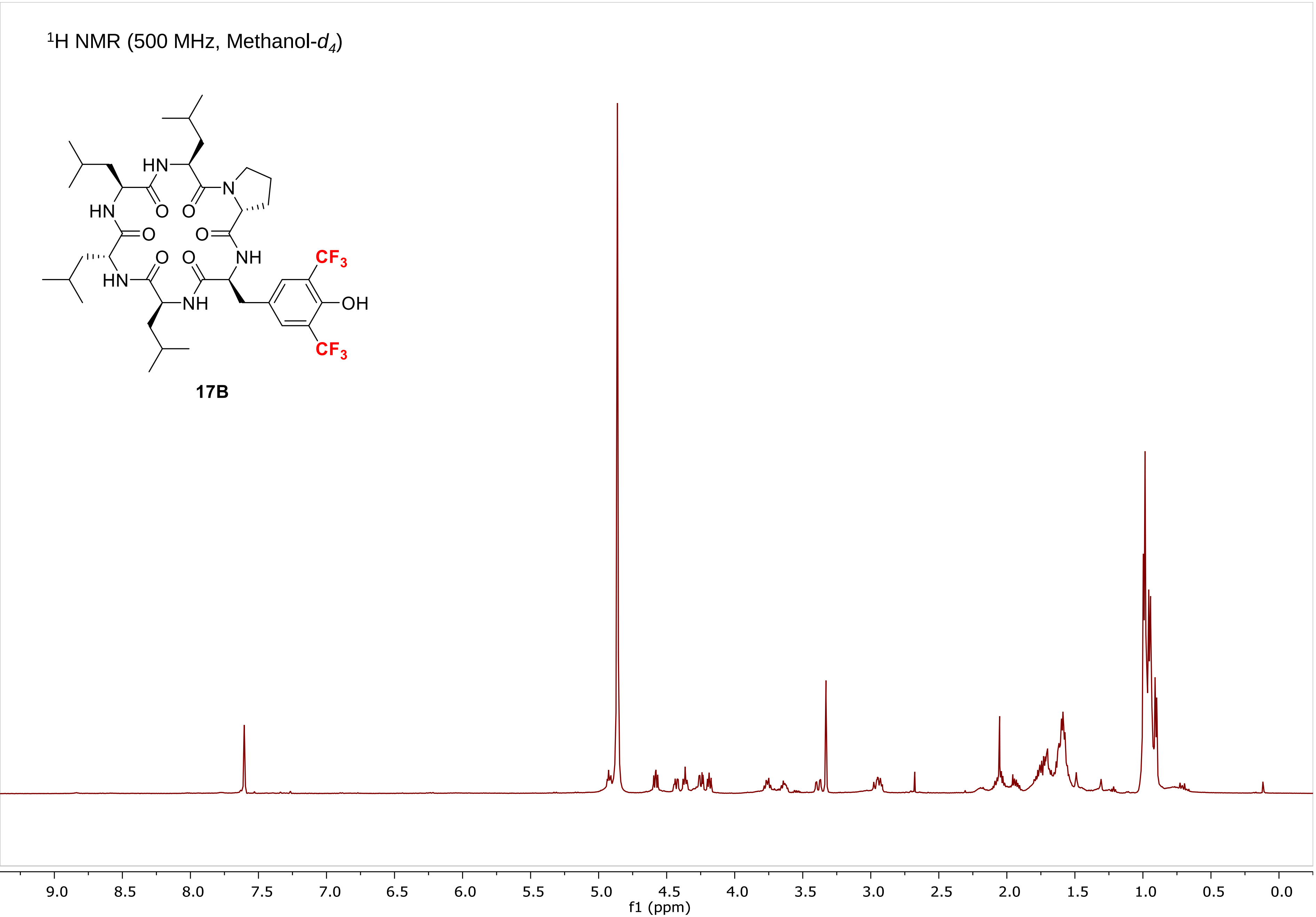
1H NMR (500 MHz, Methanol-d4)
17B
HN
NH
N
O
OH
CF3
9.0
8.5
8.0
7.5
7.0
6.5
6.0
5.5
5.0
4.5
4.0
3.5
3.0
2.5
2.0
1.5
1.0
0.5
0.0
f1 (ppm)

^{13}C NMR (125 MHz, Methanol-d_4)

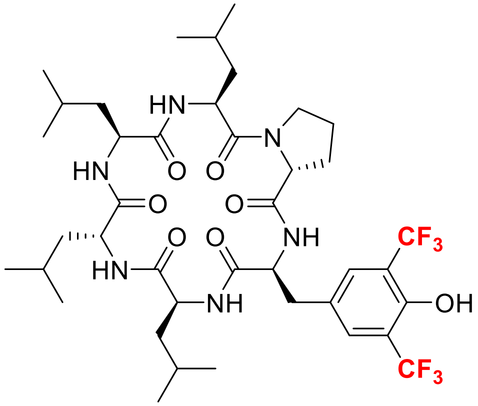

17B

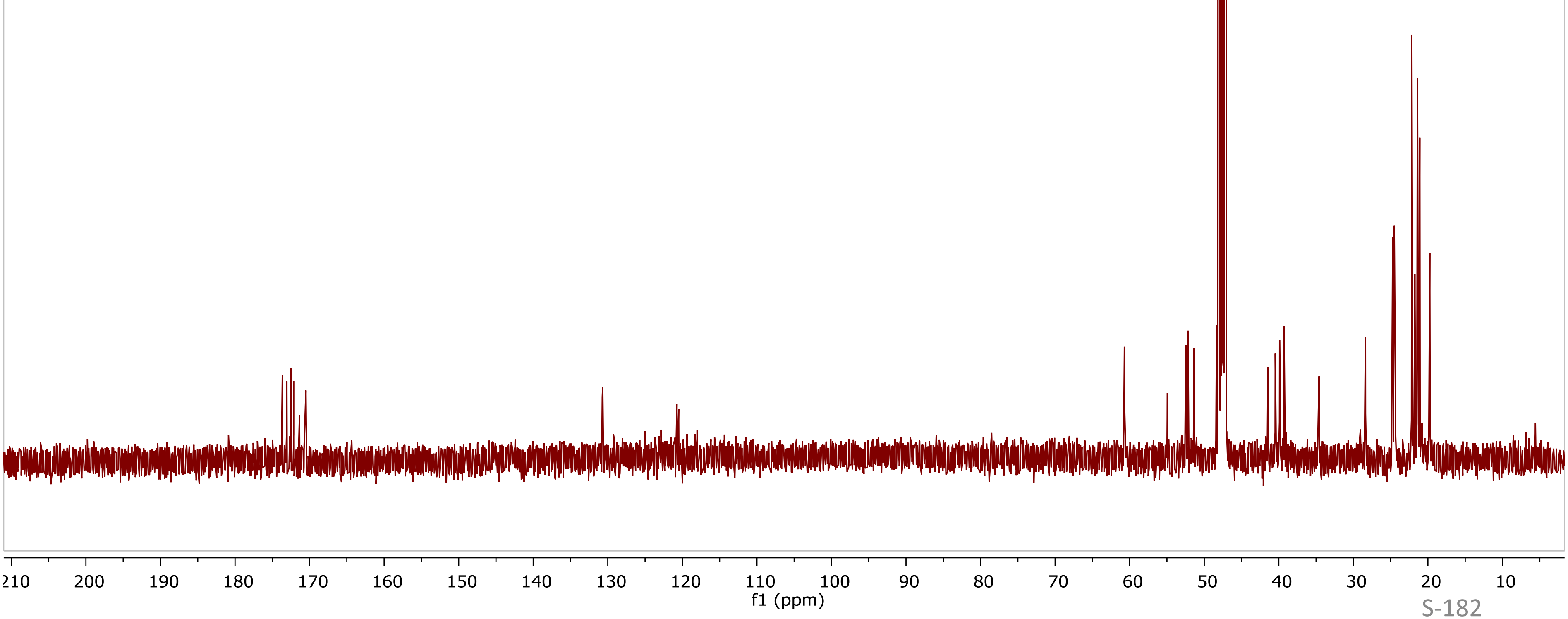

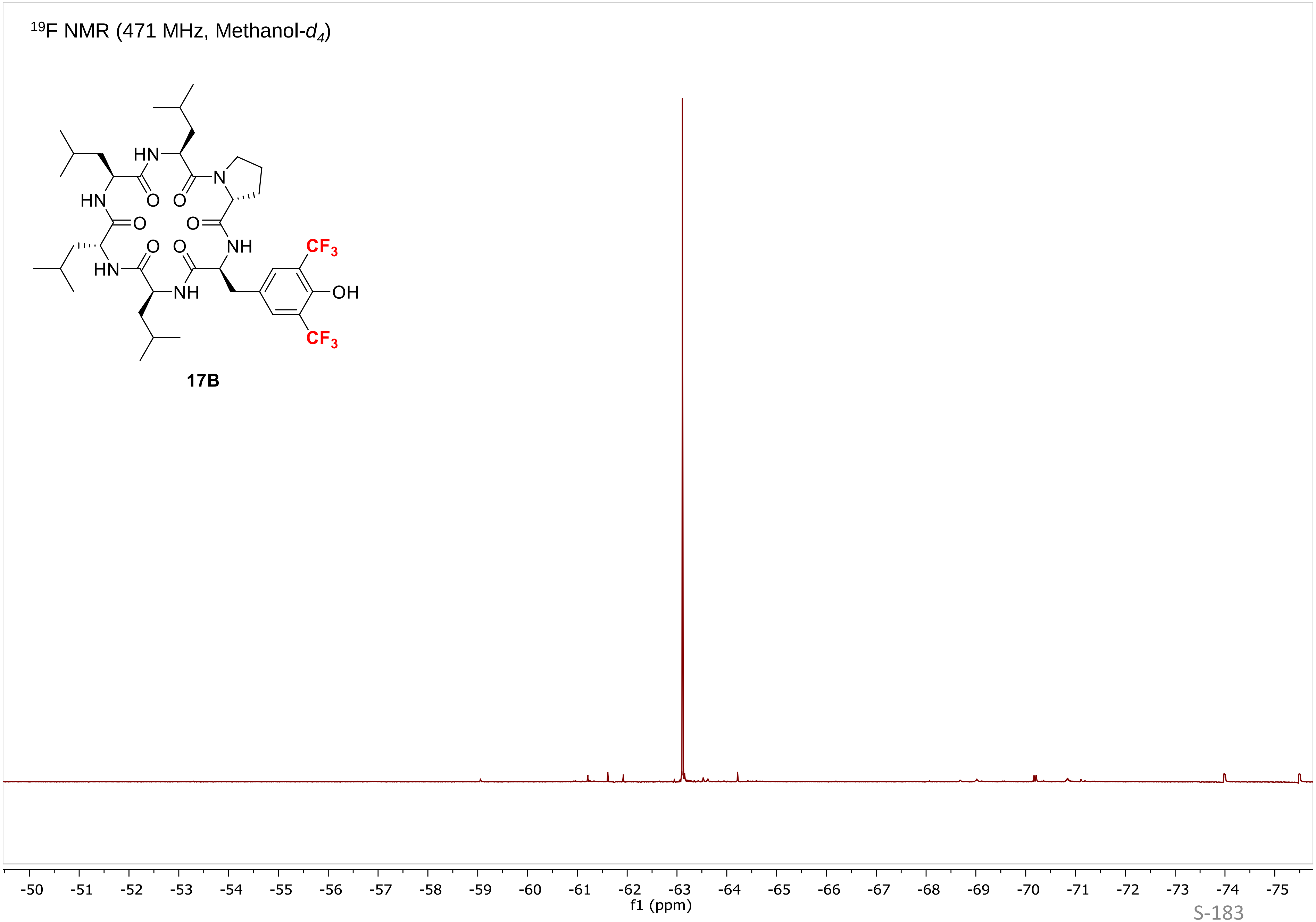

19F NMR (471 MHz, Methanol-d4)
HN
N
O
HN
O
O
NH
CF3
HN
O
O
NH
OH
CF3
17B
-50 -51 -52 -53 -54 -55 -56 -57 -58 -59 -60 -61 -62 -63 -64 -65 -66 -67 -68 -69 -70 -71 -72 -73 -74 -75
f1 (ppm)

HSQC (**17B**)

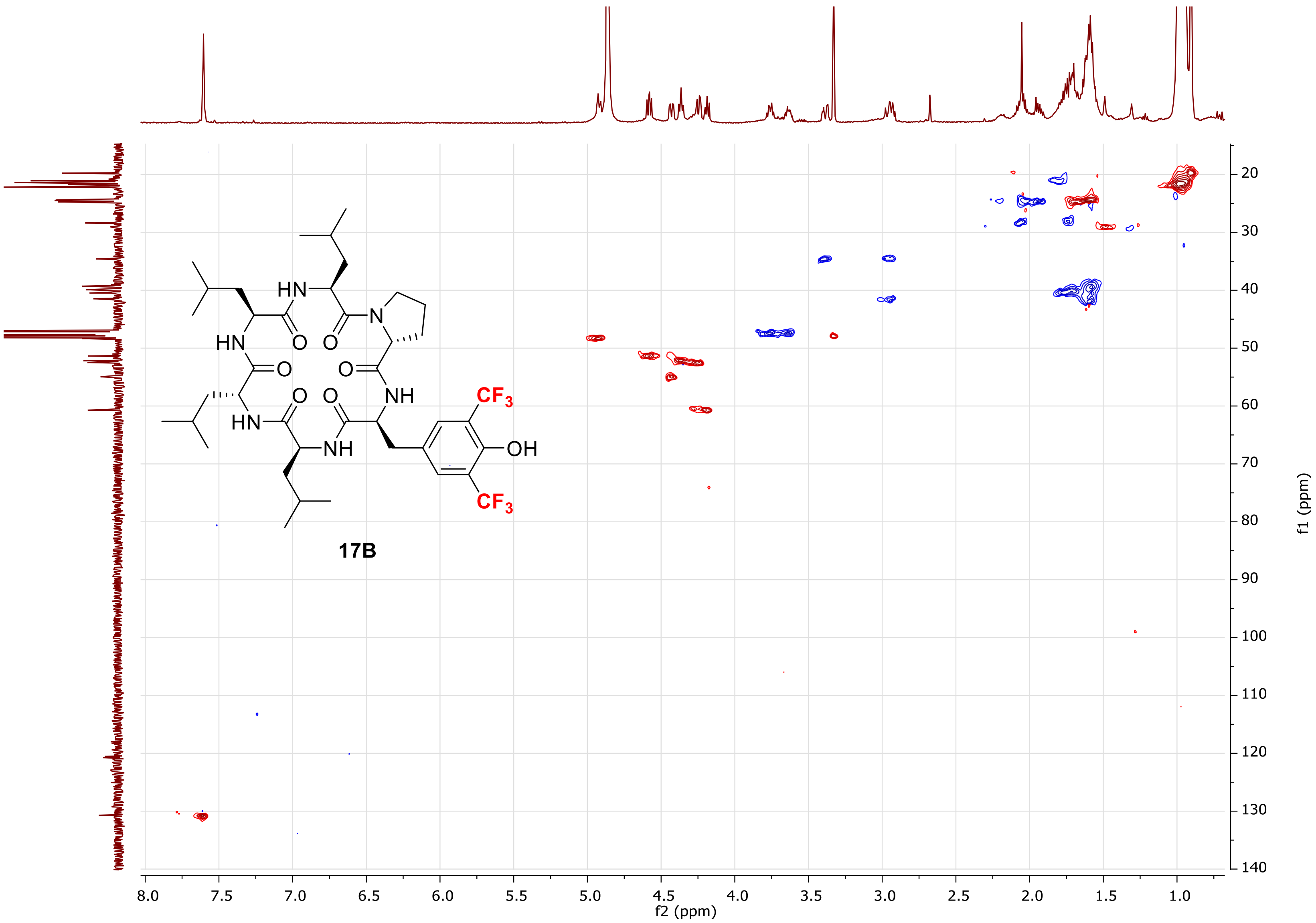

HMBC (**17B**)

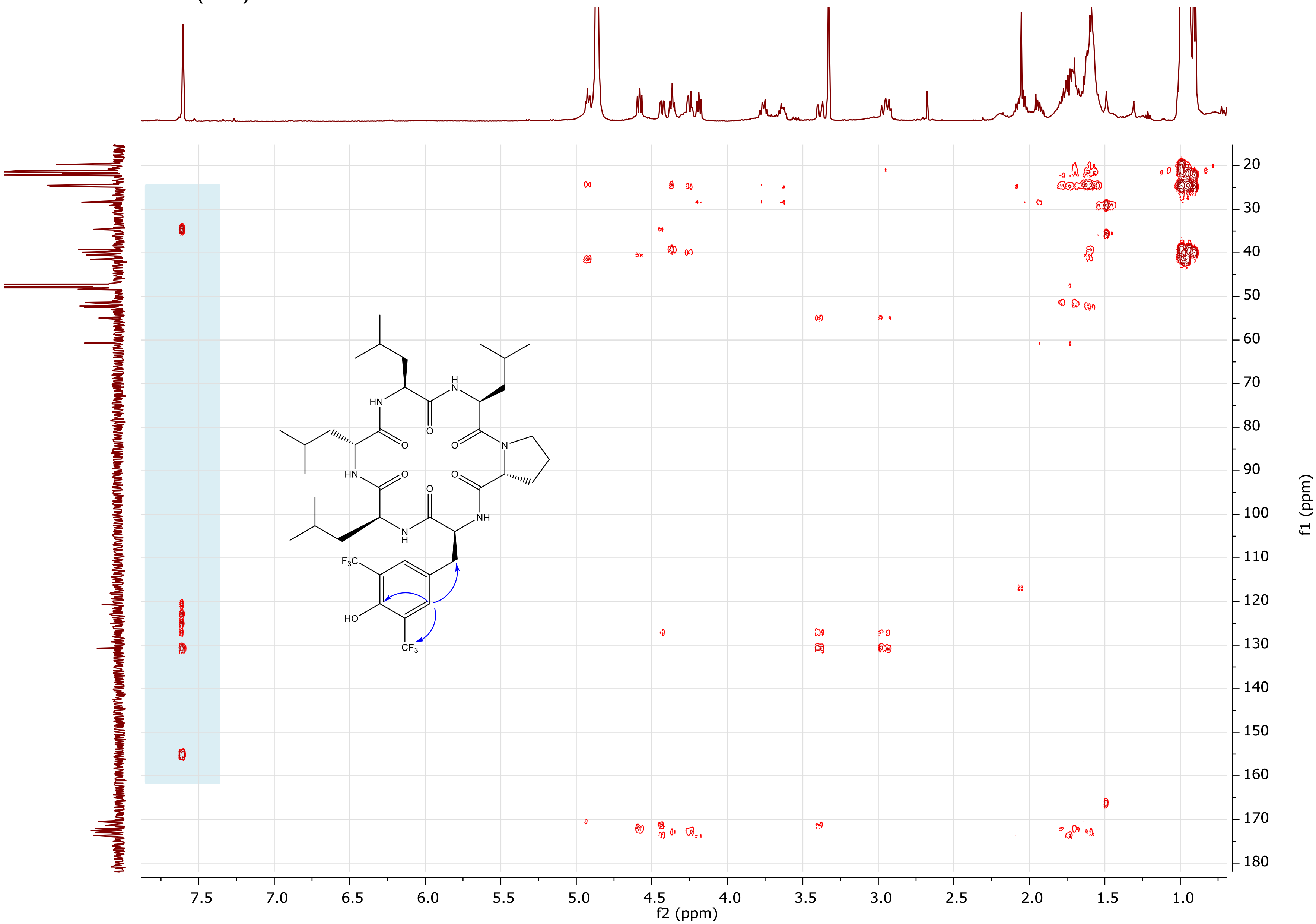

^{1}H NMR (600 MHz, Acetonitrile-d_3)

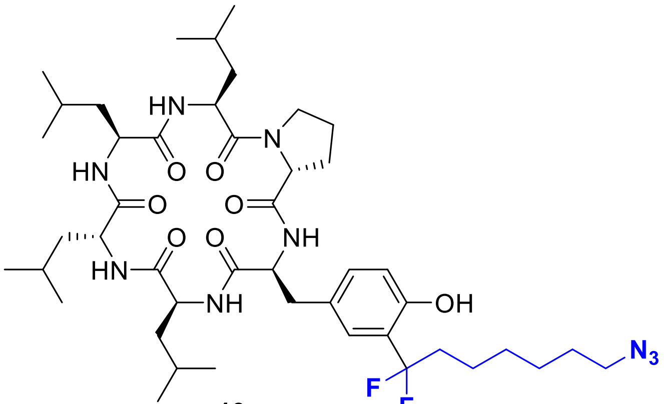

19

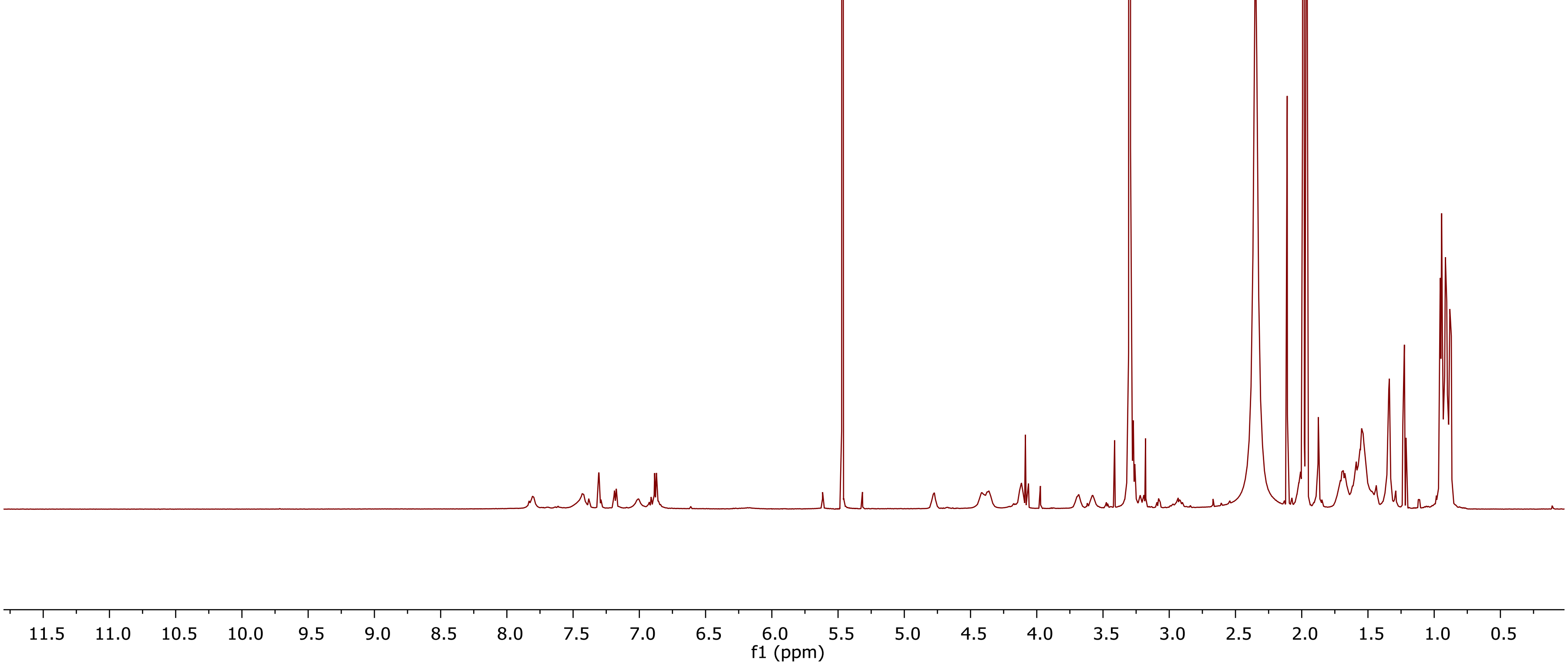

^{13}C NMR (150 MHz, Acetonitrile-d_3)

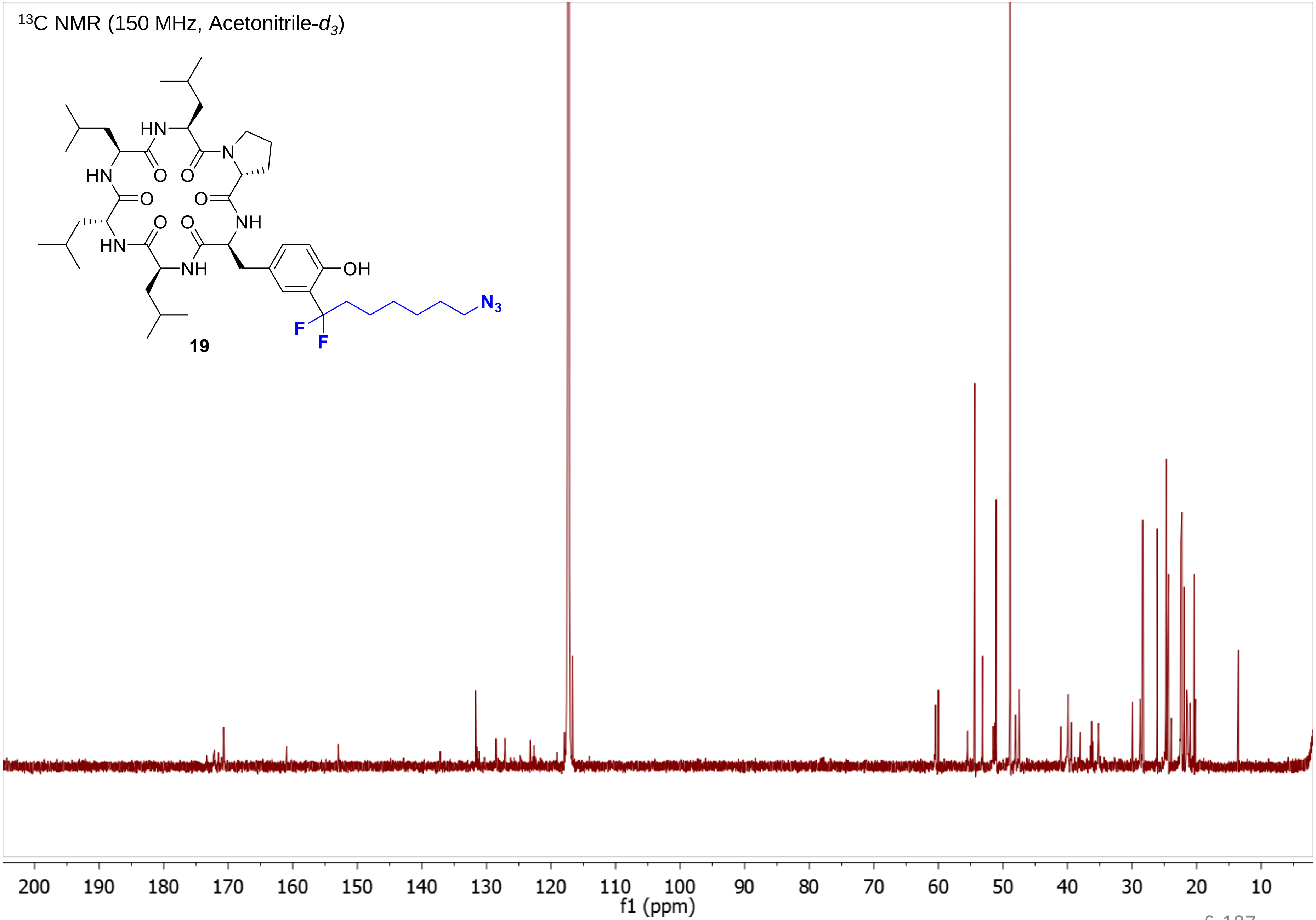

^{19}F NMR (471 MHz, Acetonitrile-d_3)

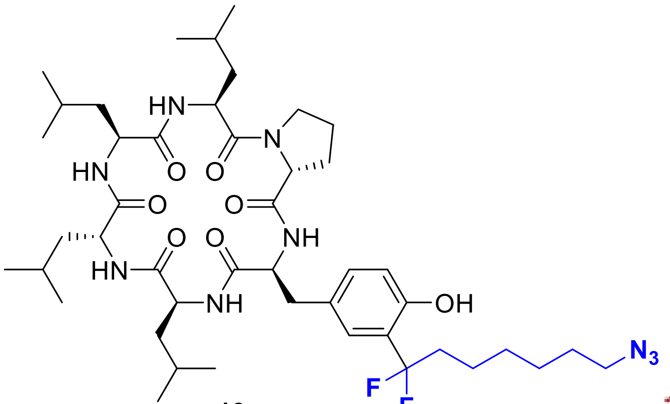

19

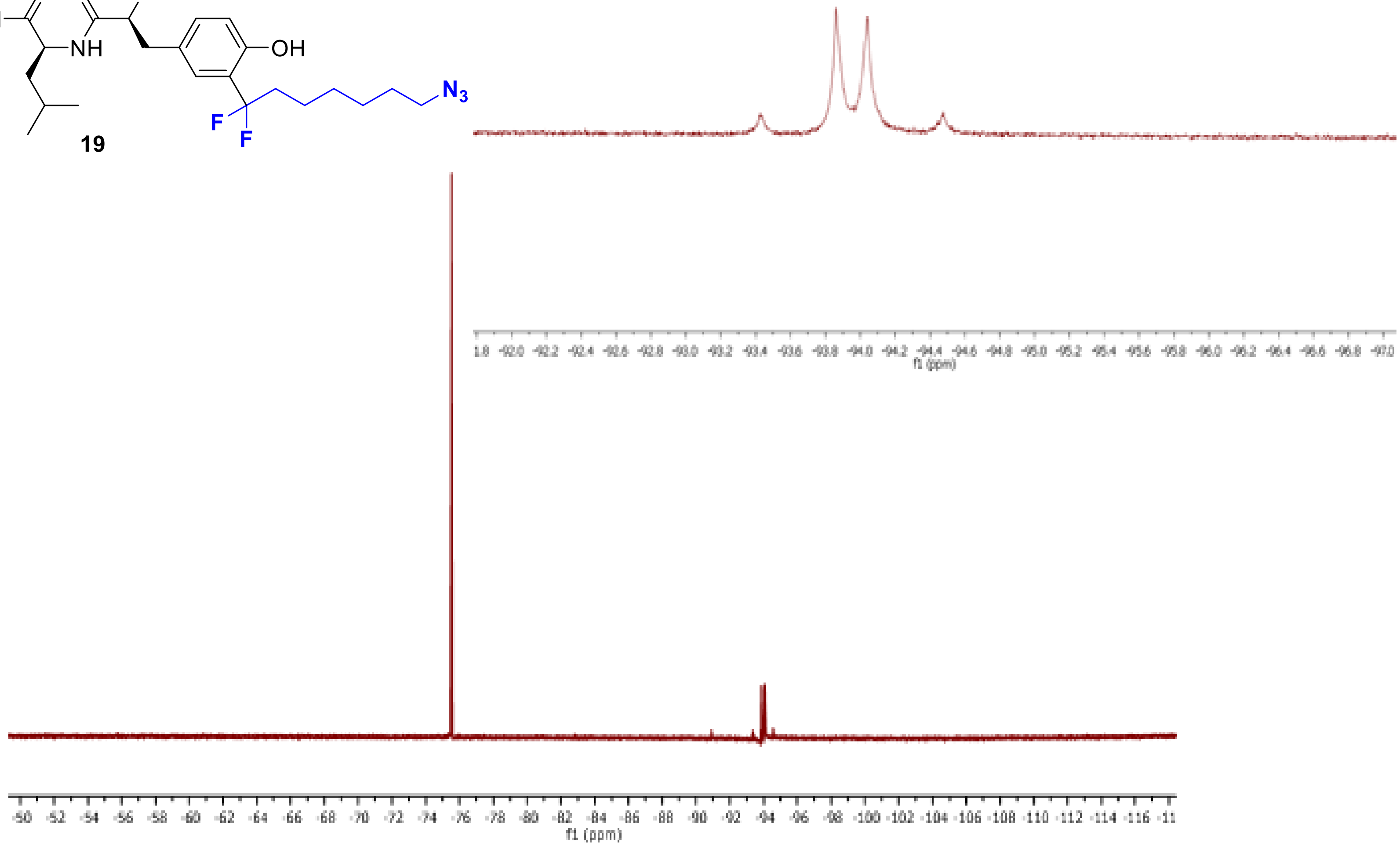

HSQC (**19**)

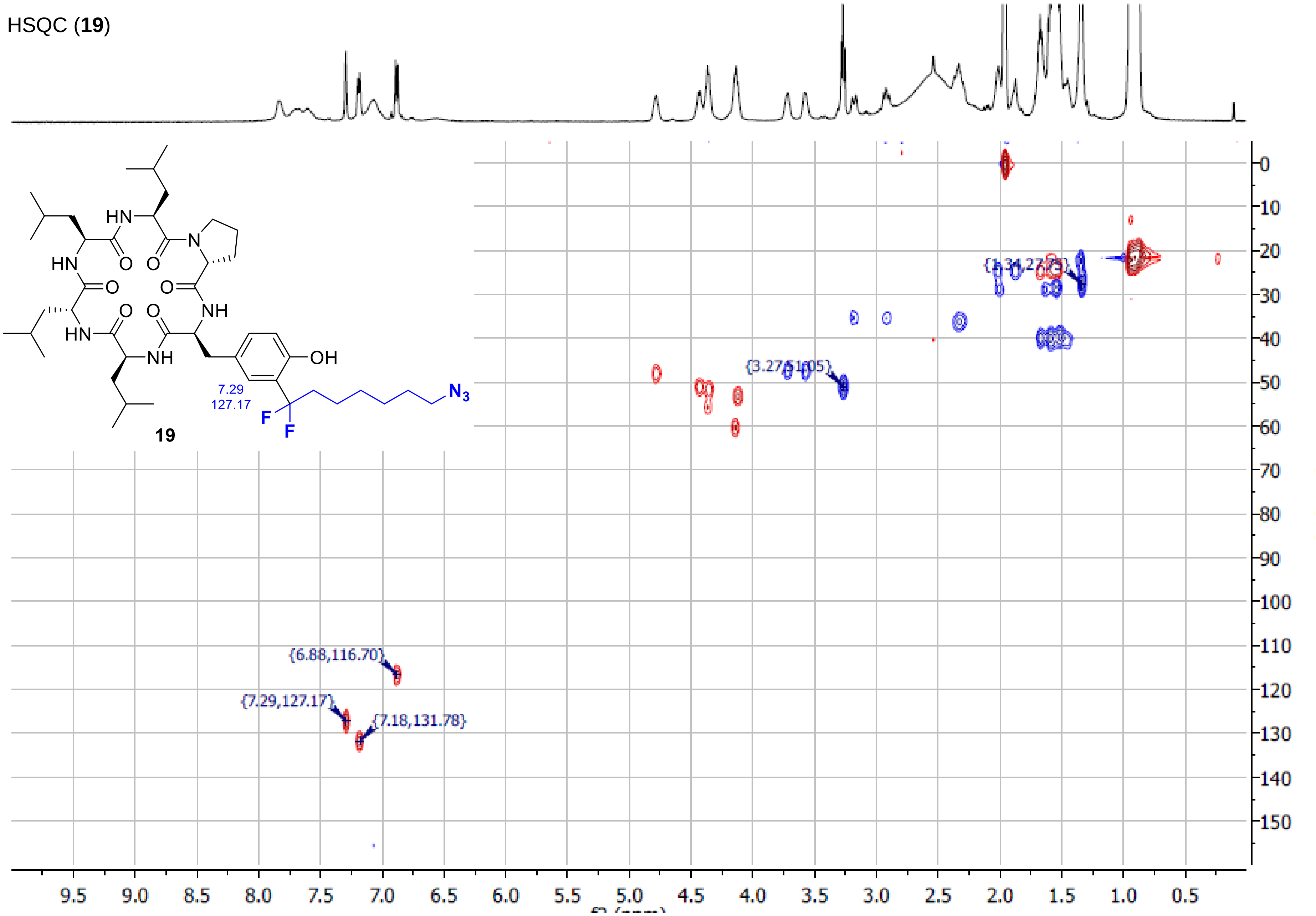

HN
O
N
HN
O
O
O
NH
O
O
HN
NH
OH
7.29
127.17
F
F
N3
19
{6.88,116.70}
{7.29,127.17}
{7.18,131.78}
{3.27,51.05}
0
10
20
30
40
50
60
70
80
90
100
110
120
130
140
150
9.5
9.0
8.5
8.0
7.5
7.0
6.5
6.0
5.5
5.0
4.5
4.0
3.5
3.0
2.5
2.0
1.5
1.0
0.5

Overlay of ^{1}HNMR of **19** and compound **17**

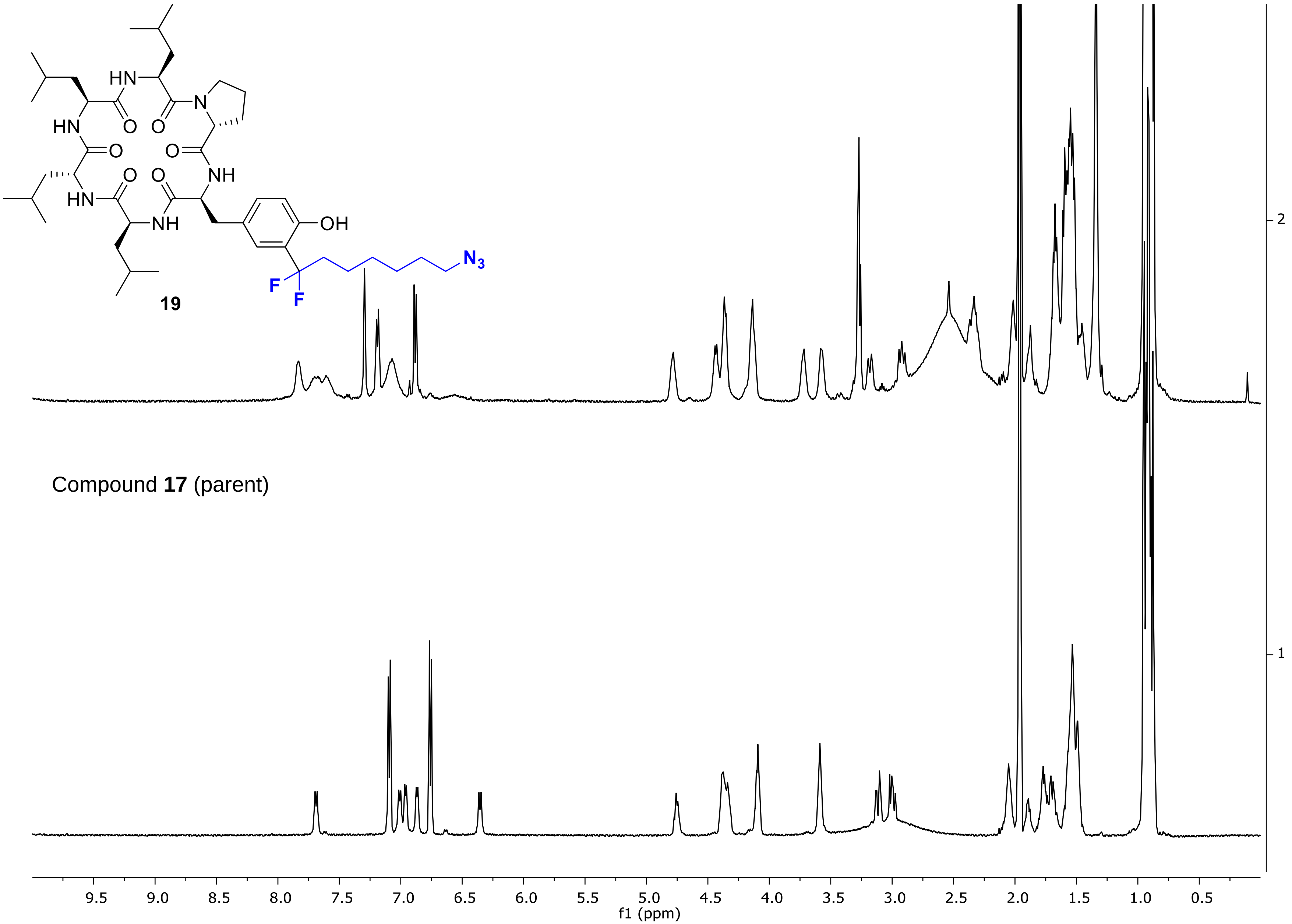

COSY (**19**)

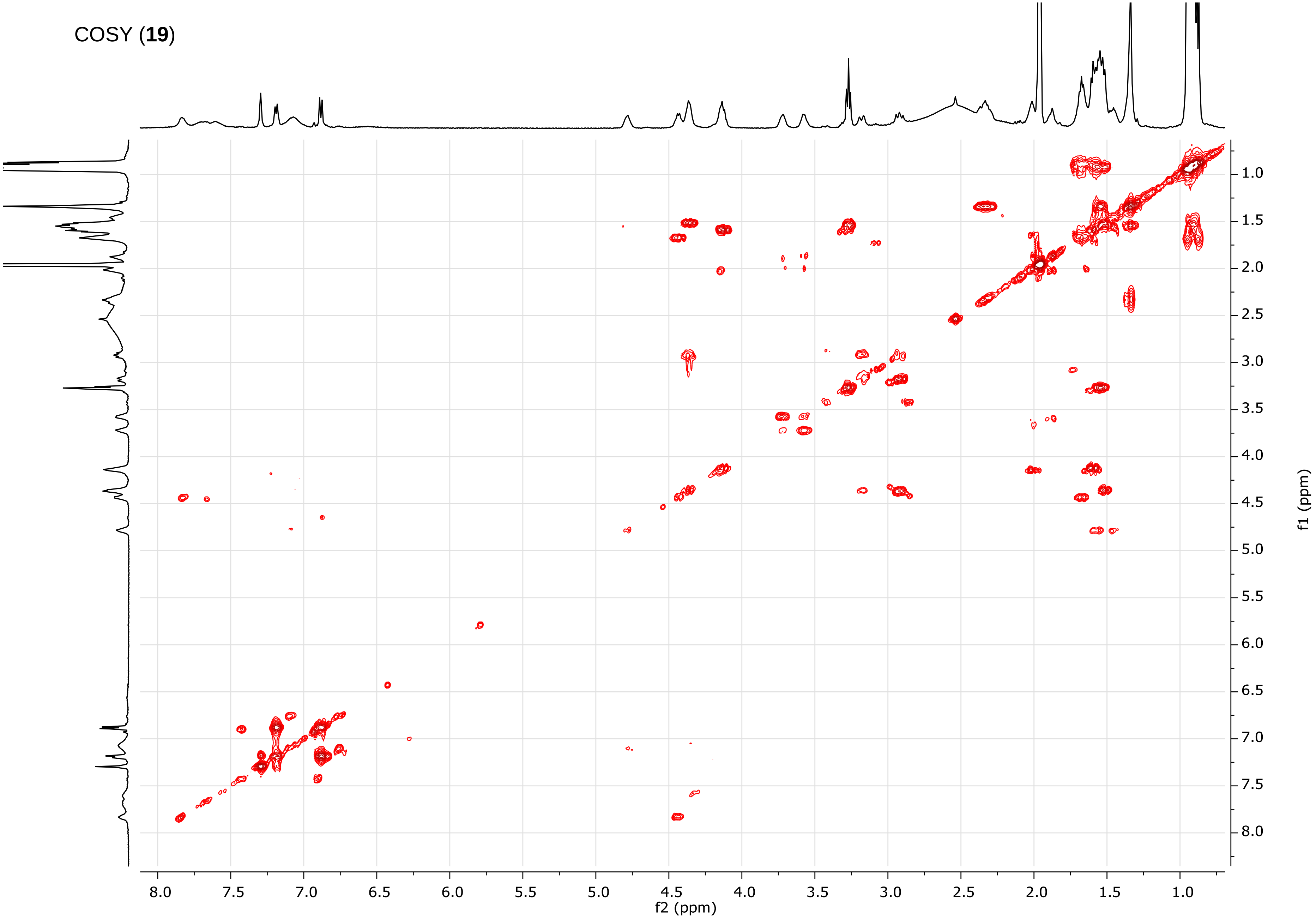

TOCSY (**19**)

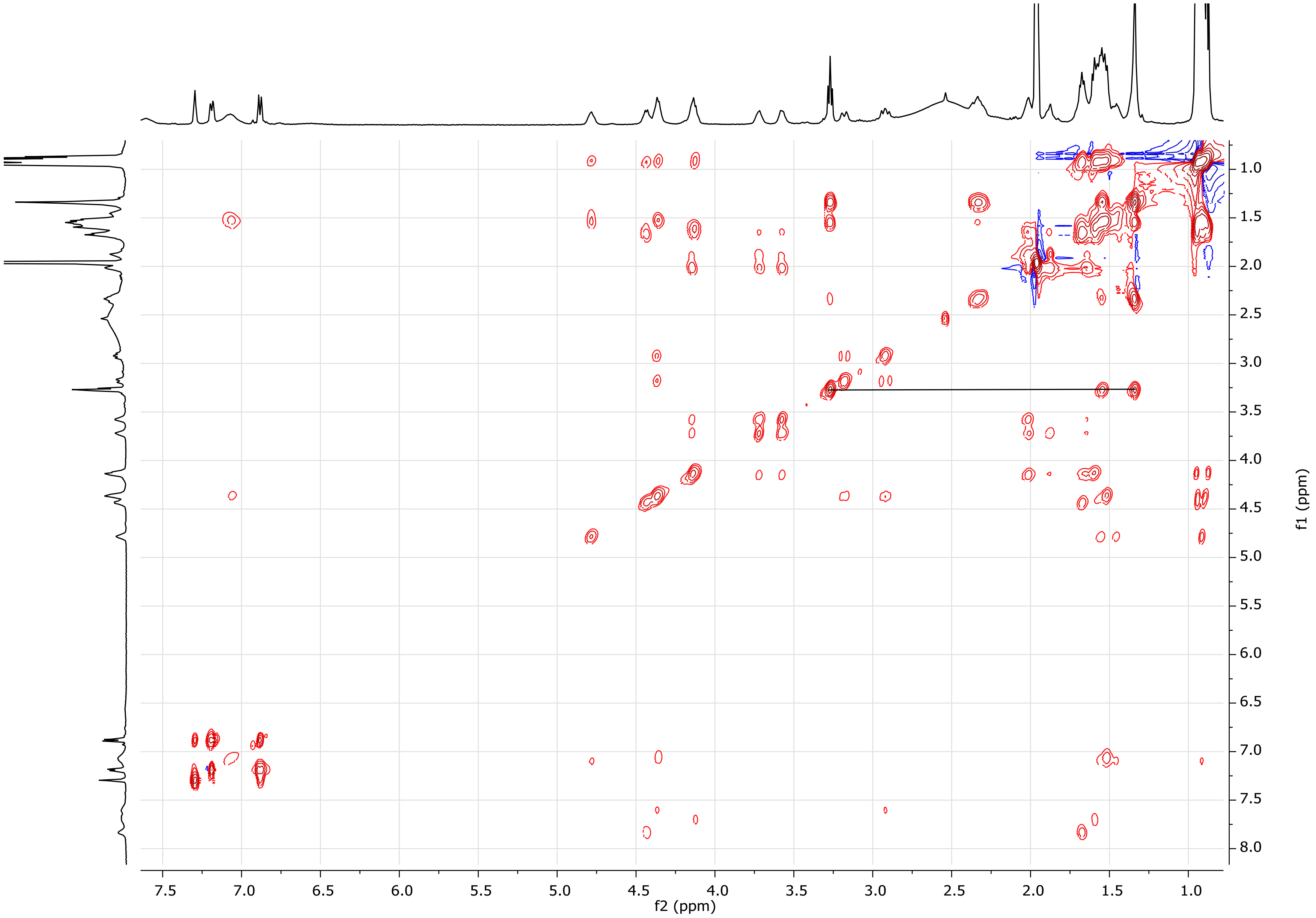

7.5
7.0
6.5
6.0
5.5
5.0
4.5
4.0
3.5
3.0
2.5
2.0
1.5
1.0
f2 (ppm)
1.0
1.5
2.0
2.5
3.0
3.5
4.0
4.5
5.0
5.5
6.0
6.5
7.0
7.5
8.0
f1 (ppm)

HSQC (**19**)

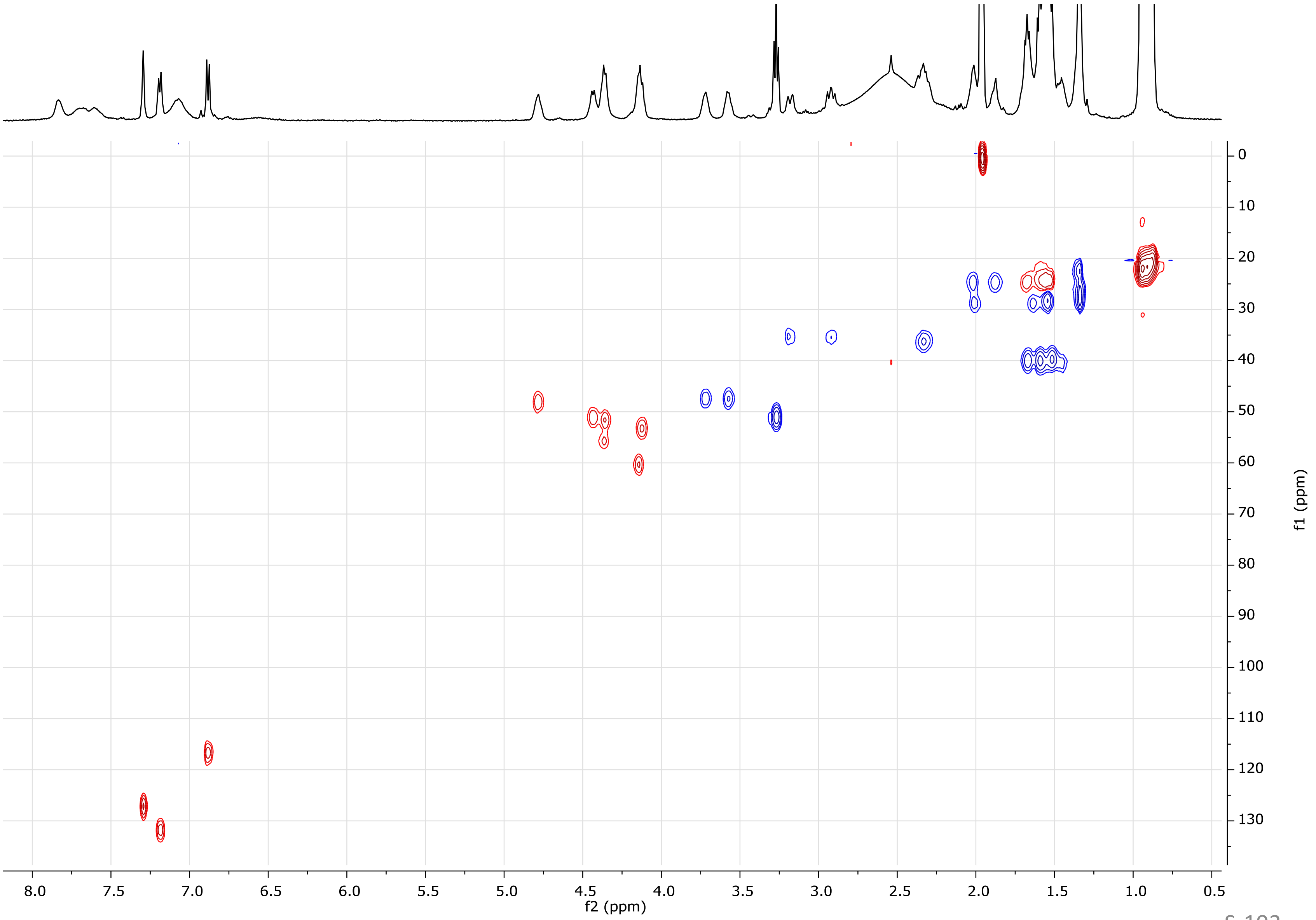

1D NOE (**19**)

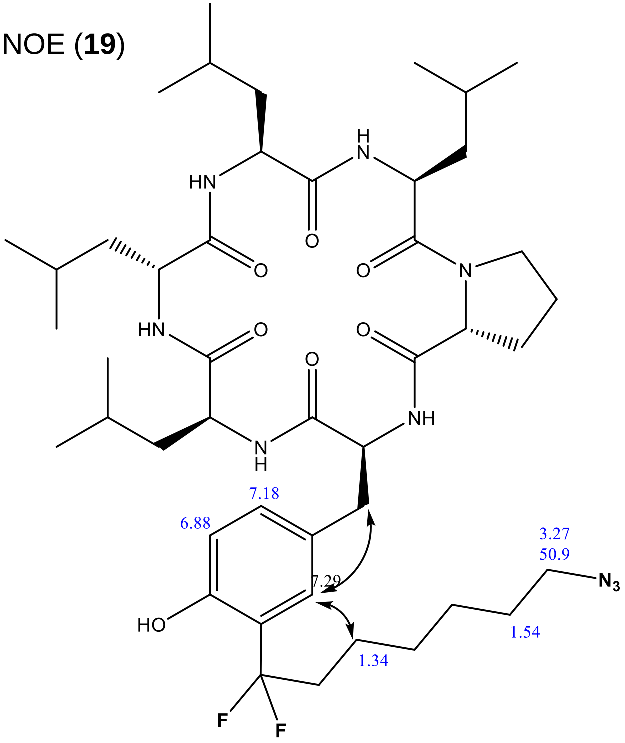

7.18
6.88
7.29
3.27
50.9
1.54
1.34
HO
F
F

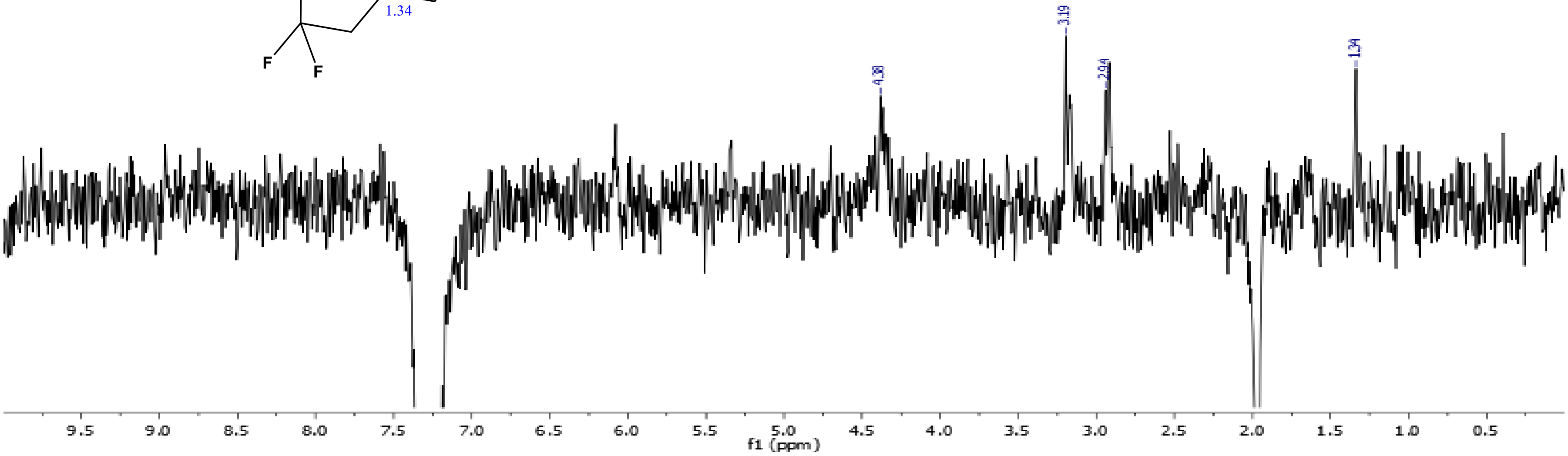

4.38
3.19
2.94
1.34
9.5
9.0
8.5
8.0
7.5
7.0
6.5
6.0
5.5
5.0
4.5
4.0
3.5
3.0
2.5
2.0
1.5
1.0
0.5
f1 (ppm)

ROESY (**19**)

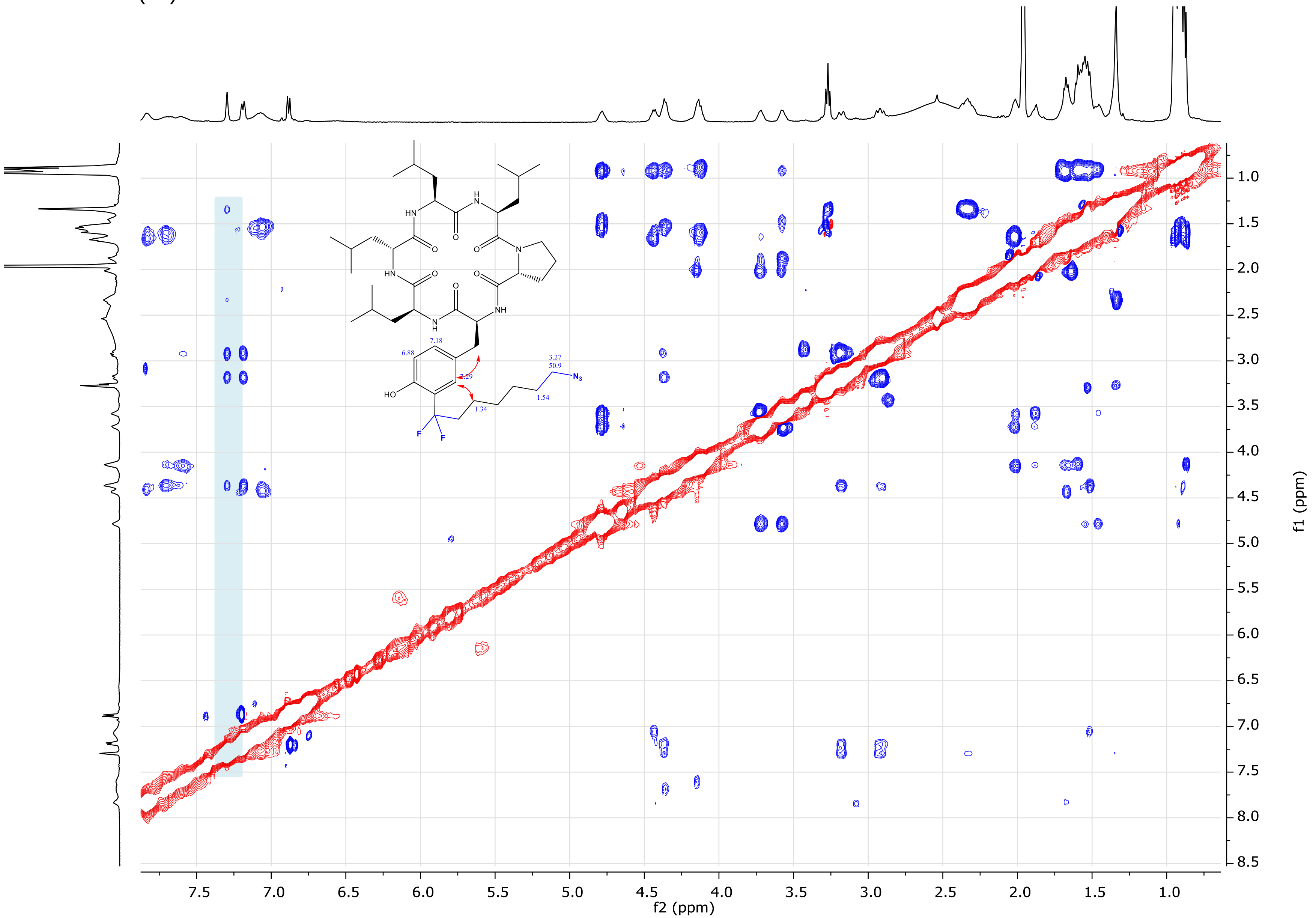

^{1}H NMR (600 MHz, Methylene Chloride-d_2)

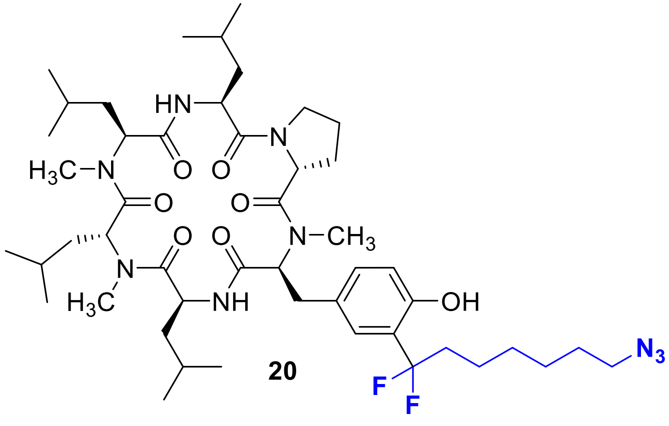

20

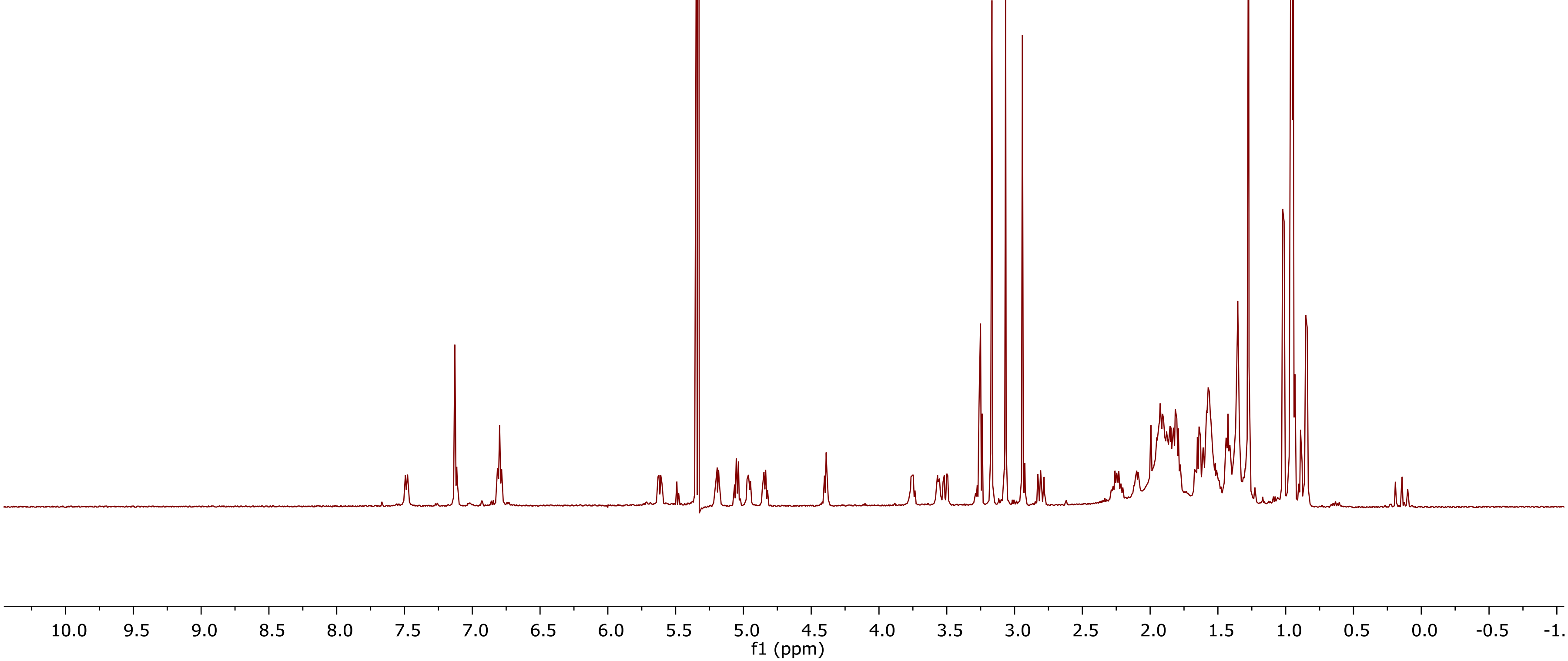

^{19}F NMR (471 MHz, Acetonitrile-d_3)

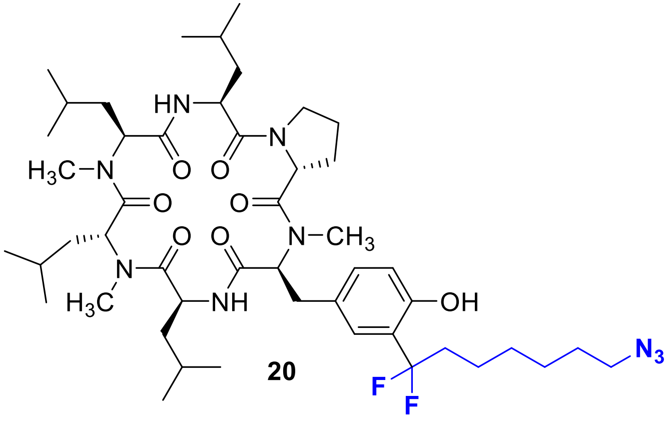

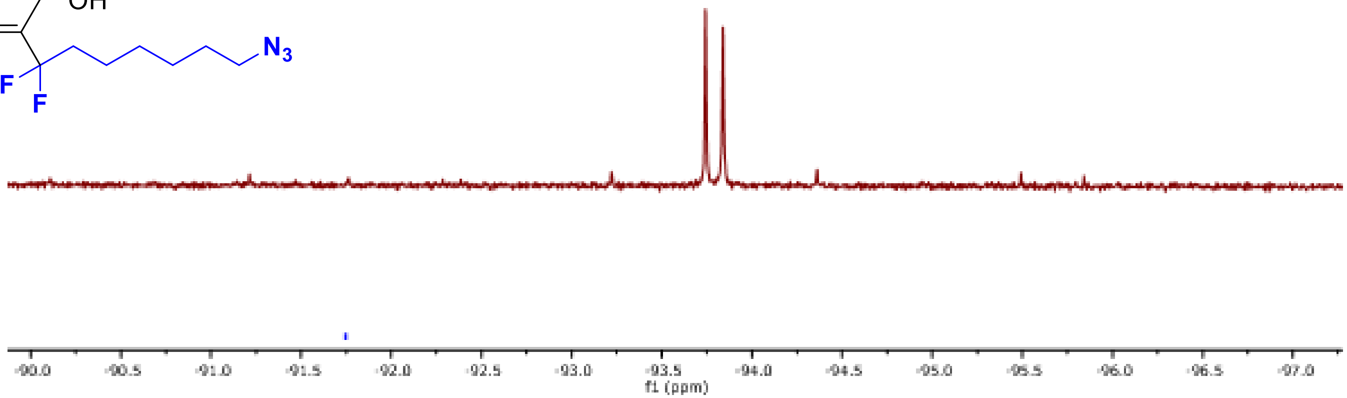

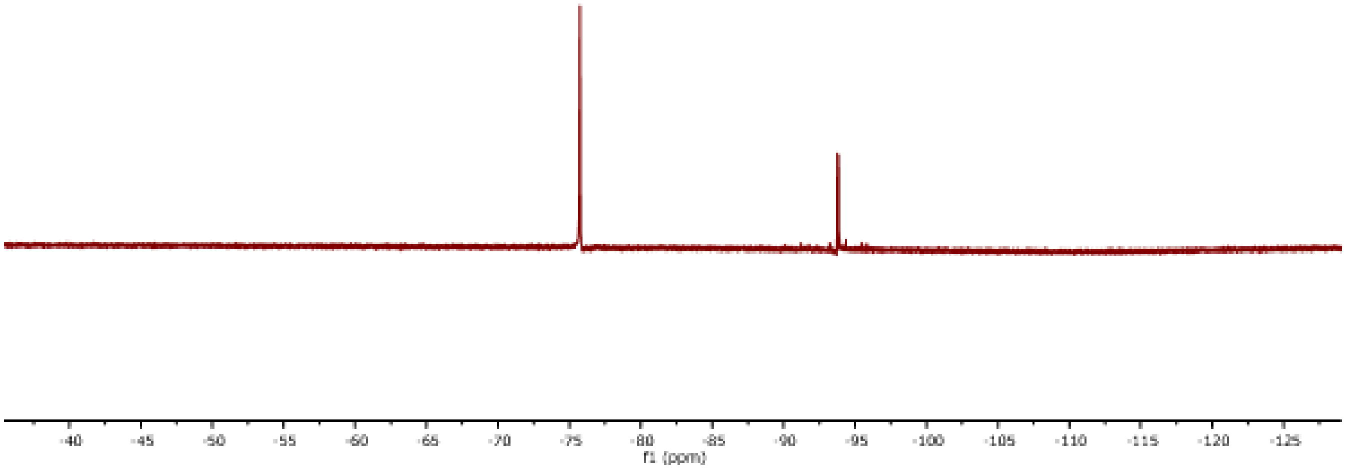

HSQC (Methylene Chloride-d_2)

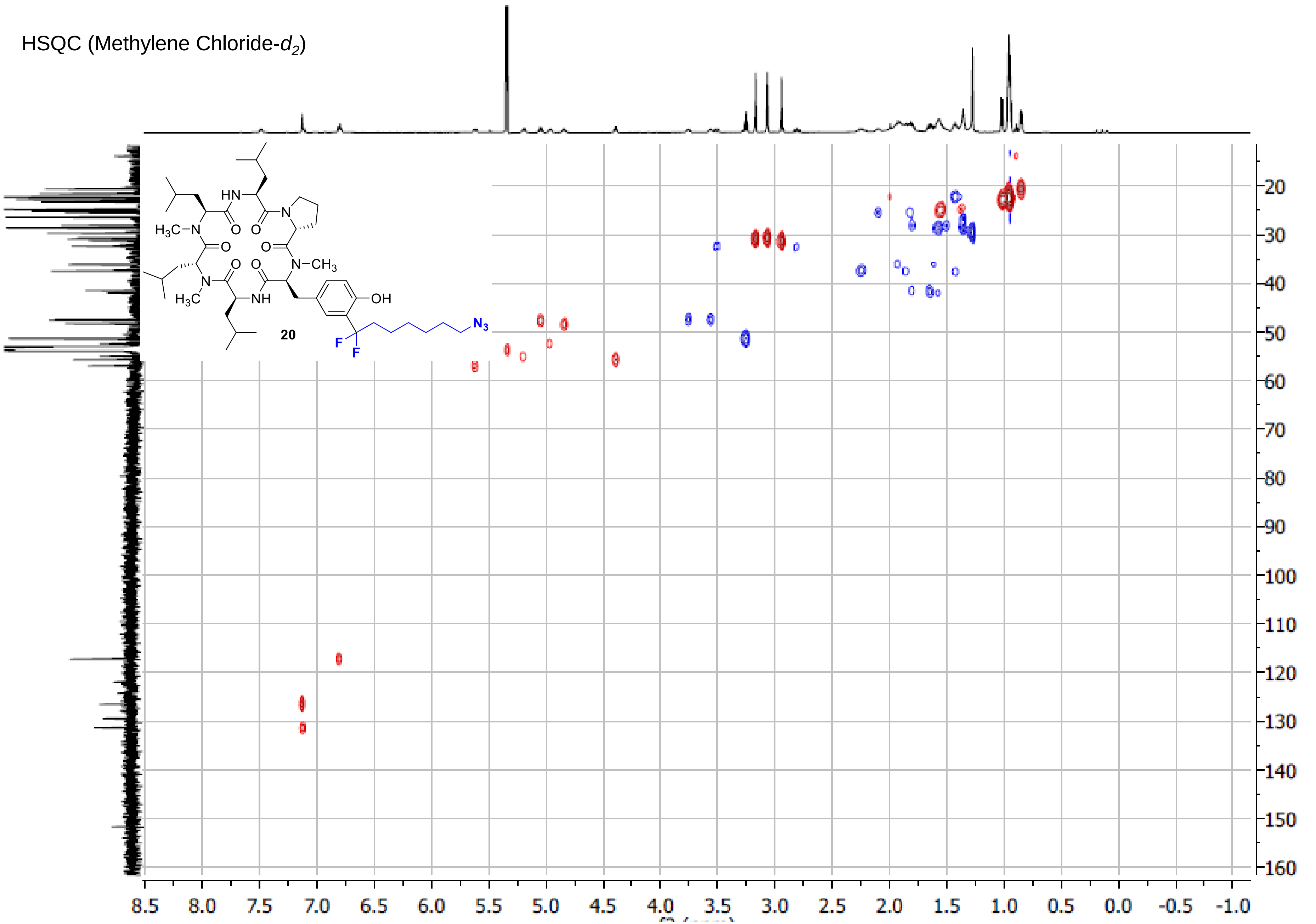

^{19}F NMR, CF_3-modified Insulin at pH = 2.9

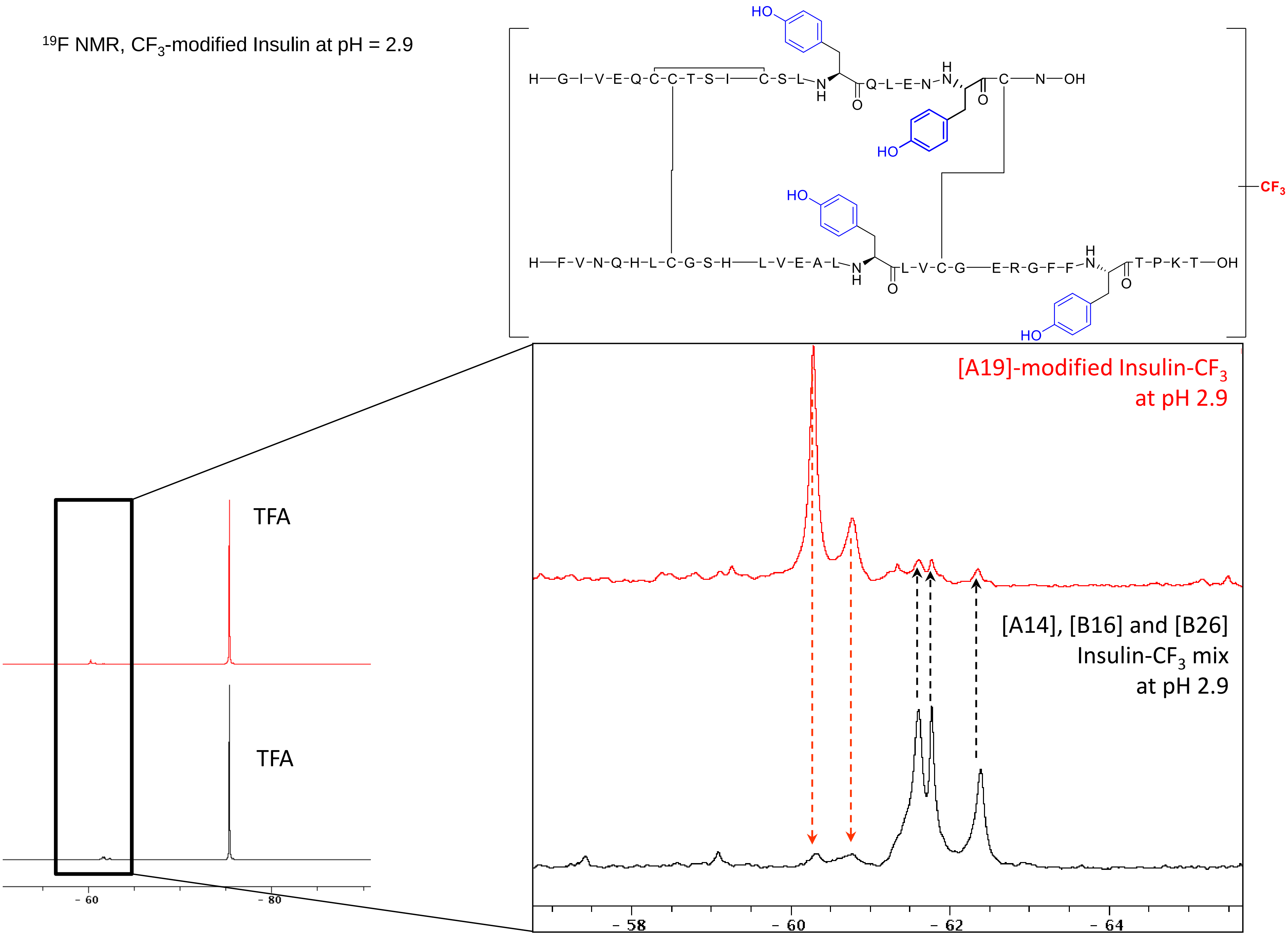

Supplement: Supplementary file 1 [file SC-009-C8SC00368H-s001.pdf]
